# Global burden and international disparities in NASH-associated liver Cancer: mortality trends (1990–2021) and future projections to 2045

**DOI:** 10.3389/fpubh.2025.1527328

**Published:** 2025-02-14

**Authors:** Qilong Nie, Yongwen Jiang, Mingyang Li, Qiuyan Liang, Xiaoai Mo, Tengyu Qiu, Qunfang Jiang, Kaizhou Huang, Youqing Xie, Ying Chen, Xiaojun Ma, Jianhong Li, Kaiping Jiang

**Affiliations:** ^1^The Eighth Clinical Medical College, Guangzhou University of Chinese Medicine, Foshan, Guangdong, China; ^2^Foshan Hospital of Traditional Chinese Medicine, Guangzhou University of Chinese Medicine, Foshan, Guangdong, China

**Keywords:** NASH-associated liver cancer, global burden of disease, joinpoint analysis, decomposition analysis, frontier analysis, prediction analysis

## Abstract

**Background:**

NASH-associated liver cancer (NALC) is a significant contributor to global cancer mortality, closely linked to the increasing prevalence of non-alcoholic fatty liver disease (NAFLD) and non-alcoholic steatohepatitis (NASH). This study comprehensively examines the global burden of NALC from 1990 to 2021.

**Methods:**

This study used data from the Global Burden of Disease (GBD) 2021 database to analyze NALC death and age-standardized death rates (ASDR) globally and regionally from 1990 to 2021. We applied Joinpoint regression analysis to assess temporal trends, calculating the annual percent change (APC) and average annual percent change (AAPC). Decomposition analysis was performed to break down mortality changes into contributions from population aging, growth, and epidemiological changes. A frontier analysis was used to evaluate the relationship between NALC burden and sociodemographic development using the Socio-Demographic Index (SDI). Prediction analysis of NALC deaths and ASDR from 2021 to 2045 were estimated using the Nordpred model.

**Results:**

From 1990 to 2021, the global burden of NALC deaths increased significantly, with the ASDR rising from 0.38 per 100,000 in 1990 to 0.48 per 100,000 in 2021. Age-specific data in 2021 revealed that NALC deaths peaked in the 65–69 age group for men and 70–74 age group for women. Decomposition analysis indicated that population growth was the most significant contributor to the global NALC death toll, followed by population aging and epidemiological changes. Frontier analysis showed that countries like Mongolia and Gambia were farthest from the disease burden frontier, while Morocco and Ukraine were closest. Prediction analysis suggest a significant increase in NALC deaths by 2045 compared to 2021, with a larger rise in deaths among women.

**Conclusion:**

Through this study, a data-driven approach is provided to reduce the global disease burden of NALC. Essential data support for public health prevention strategies is offered, helping guide the development of targeted government interventions. Trends across global regions, countries, age groups, and genders have been analyzed, providing valuable insights for the formulation of evidence-based policies aimed at mitigating the impact of NALC worldwide.

## Introduction

Non-alcoholic fatty liver disease (NAFLD) is a hepatic disorder characterized by the accumulation of fat in the liver, often closely linked to obesity, insulin resistance, type 2 diabetes mellitus (T2DM), and metabolic syndrome (1). NAFLD can progress from simple steatosis, known as non-alcoholic fatty liver (NAFL), to non-alcoholic steatohepatitis (NASH), a more severe condition that may lead to advanced liver diseases, such as cirrhosis and hepatocellular carcinoma (HCC) (2).

The global prevalence of NAFLD was 38.2% between 2016 and 2019 (3), and it has steadily increased over the past three decades. From 2005 to 2010, the prevalence rose from approximately 15–25%, with rates of NASH nearly doubling during the same period (4). Notably, NAFLD is responsible for a significant proportion of non-cirrhotic HCC cases, accounting for 26.3% of non-cirrhotic HCC, compared to 13.4% in cirrhotic HCC cases (5). Among patients with NASH, the incidence of HCC occurring without cirrhosis ranges from 20 to 50%, in contrast to <10% in other liver diseases such as hepatitis C virus (HCV) infection. This suggests that factors related to the pathogenesis of NAFLD may directly contribute to the development of HCC, independent of the progression to advanced fibrosis or cirrhosis (6–10).

A meta-analysis of 50 studies published by August 2020 found that the incidence of HCC in patients with NAFLD is 1.25 per 1,000 person-years, with a hazard ratio of 1.88 compared to individuals without NAFLD (11). Specifically, a meta-analysis by Petrelli et al. (12) reported that NAFLD is associated with more than twice the risk of dying from HCC compared to individuals without NAFLD (HR 2.16, 95% CI 0.85–5.5). These findings highlight the growing burden of NASH-associated liver cancer (NALC) and the substantial role NAFLD, particularly NASH, plays in its development.

The global burden of NALC has increased significantly over the past few decades, driven by the rising prevalence of metabolic diseases such as obesity, type 2 diabetes, and high fasting plasma glucose (HFPG). According to the Global Burden of Disease (GBD) Study, in 2019, approximately 7.6% of all Disability-Adjusted Life Year (DALY) and 8.8% of all deaths from NALC were attributed to HFPG (13). Furthermore, the overall burden of NALC has risen sharply, with a 43.5% increase in the proportion of liver cancer cases attributed to NASH from 1990 to 2019 (14). These trends highlight the growing significance of NASH as a cause of liver cancer, particularly in regions with higher socio-demographic indexes (SDI), and emphasize the need for public health interventions targeting metabolic risk factors such as HFPG.

Building on this understanding, the present study seeks to comprehensively analyze global trends in NALC-associated mortality across various demographic groups, including age, sex, region, and SDI levels, in 204 countries and territories. Using data from the GBD 2021, we applied a suite of advanced epidemiological models. These included Joinpoint regression analysis to detect significant shifts in temporal trends, decomposition analysis to determine the specific contributions of population aging, growth, and epidemiological changes to NALC mortality, frontier analysis to examine the relationship between sociodemographic development and disease burden, prediction analysis to forecast future trends. Through this multifaceted approach, we identified both short- and long-term trends, providing crucial insights for public health interventions and resource allocation, with a particular emphasis on addressing global health disparities related to NALC mortality.

## Methods

### Overview and data source

The GBD project remains one of the largest and most systematic epidemiological research efforts conducted globally. The GBD 2021 study, released in 2024, provided a detailed analysis of 371 diseases and injuries, alongside 88 risk factors worldwide (15). The data aggregation for GBD 2021 involved a thorough collection process from diverse sources, such as population censuses, household surveys, civil registration records, disease registries, health service utilization data, air quality monitoring, satellite imagery, and other relevant health-related databases (16). These data types are identified through a systematic review of published research, searches of governmental and international organization websites, official reports, primary data sources like the Demographic and Health Surveys, and the contributions of datasets from GBD collaborators (17). Newly identified and obtained data sources are uniquely tagged by a dedicated team of librarians and integrated into the Global Health Data Exchange (GHDx). GHDx ensures public access to the metadata of each source included in the GBD, along with the actual data, subject to approval from the data providers. The GHDx source tool allows users to identify the specific datasets used to estimate outcomes for various diseases or injuries in different settings (15).

Global mortality data and population estimates for NALC were extracted from the GBD 2021 database, with further disaggregation by age group, sex, year, country, and region. The GBD 2021 data can be accessed through the GHDx query.^1^ The global burden of NALC was assessed based on death and their rates, stratified by sex, region, and SDI (The regional division of SDI can be obtained from the Institute for Health Metrics and Evaluation).^2^ The SDI is categorized into quintiles based on the geometric mean of three key indicators: per capita income, fertility rate among individuals under 25 years of age, and average educational attainment for individuals aged 15 years and older. These indicators collectively represent a country’s level of social and economic development (15).

The GBD 2021 estimated mortality for 288 causes of death across age, sex, location, and year in 204 countries and territories, and 811 subnational locations from 1990 to 2021. The analysis utilized 56,604 data sources, including vital registration data, verbal autopsy, surveys, censuses, surveillance systems, and cancer registries, among others. For most causes of death, cause-specific death rates were estimated using the Cause of Death Ensemble model (CODEm), a statistical tool developed by GBD to assess the predictive validity of different models and covariate permutations. This model combines the results of various statistical approaches to produce cause-specific mortality estimates, with alternative strategies applied to model causes with insufficient data or substantial changes in reporting over time (18).

In the GBD, malignant neoplasms of the liver, specifically those attributed to NASH, are classified under the ICD-10 codes C22.0–C22.1, C22.3–C22.8, and a proportion of C22.9, which reflects primary liver cancer. These ICD-10 codes are used to capture liver cancers that are specifically linked to NASH-related liver disease. By using these classifications, GBD estimates the burden of liver cancer attributable to NASH, helping to better understand its contribution to liver cancer mortality across various populations (Supplementary Table S1).

### Statistical analysis

In this study, the primary indicators used to describe the burden of NALC were mortality and the corresponding mortality rates. The analysis was performed for specific age groups, including: 15–19 years, 20–24 years, 25–29 years, 30–34 years, 35–39 years, 40–44 years, 45–49 years, 50–54 years, 55–59 years, 60–64 years, 65–69 years, 70–74 years, 75–79 years, 80–84 years, 85–89 years, 90–94 years, and 95 plus years. Subgroup analyses were conducted based on sex (men and women), SDI categories (high, high-middle, middle, low-middle, and low SDI), and 21 GBD regions, including South Asia and East Asia, North Africa and the Middle East, Western Sub-Saharan Africa, Southeast Asia, Eastern Sub-Saharan Africa, Central Latin America, Tropical Latin America, Central Sub-Saharan Africa, Western Europe, High-income North America, Central Asia, Eastern Europe, Southern Sub-Saharan Africa, High-income Asia Pacific, Central Europe, Andean Latin America, Caribbean, Southern Latin America, Oceania, and Australasia, as well as 204 countries/territories. Each mortality rate was reported per 100,000 individuals, with a 95% uncertainty interval (UI), as calculated using the GBD methodology for modeling cause-specific mortality (18).

An explanatory table listing all the Country/Region groupings used in this study, including SDI countries and GBD regions, along with the terms and definitions related to GBD, has been added as Supplementary Table S2. The crude death rate is one of the most fundamental measures for assessing the epidemiological trends of disease; however, variations in the age structure of populations can result in differences in the burden of NALC. To ensure the comparability of these indicators, we adjusted the crude rate by applying weights based on the age distribution, resulting in the age-standardized rate (ASR). The calculation of ASR is detailed in the Supplementary methods. Ultimately, the age-standardized death rate (ASDR) was utilized to assess the burden of NALC.

### Joinpoint analysis

In this study, the Joinpoint regression analysis model was utilized, a statistical approach frequently applied in epidemiological research to examine temporal trends in disease prevalence or mortality (19). This model is particularly effective in identifying significant change points within time-series data, which, in this case, were used to analyze trends in NALC prevalence on both global and national levels. The Joinpoint model enabled the calculation of the annual percent change (APC) and its corresponding 95% confidence interval (CI) to describe trends over specific time periods. Additionally, the average annual percent change (AAPC) was computed to provide a comprehensive overview of the trend across the entire study period from 1990 to 2021.

From a statistical perspective, an APC or AAPC with a 95% CI lower bound above zero indicates an increasing trend, while a 95% CI upper bound below zero suggests a decreasing trend. If the 95% CI includes zero, it signifies that the trend has remained stable. The analysis employed a Poisson distribution-based Joinpoint model to explore both short-term and long-term shifts in the disease burden of NALC. Short-term trends in the ASDR were quantified using the APC, while long-term trends were represented by the AAPC. Trends were deemed increasing or decreasing if the APC or AAPC was statistically significant (*p* < 0.05); otherwise, they were considered stable.

### Decomposition analysis

We implemented the decomposition analysis method originally proposed by Das Gupta (20), combined with the improved approach developed by Cheng and colleagues (21) in 2020, to disaggregate changes in NALC mortality. The essence of decomposition analysis lies in its ability to break down the overall variation in a given outcome into the contributions of distinct factors. In this case, we decomposed the observed shifts in NALC deaths into three key group-level determinants: population aging, population growth, and epidemiological change. This approach allowed us to quantify the specific contribution of each factor to the total change in mortality, providing a more comprehensive understanding of how demographic and epidemiological dynamics interact to influence mortality trends. Through this method, we were able to assess not only the magnitude of each determinant’s impact but also its relative importance in shaping the overall trend. The methodology of the decomposition analysis is further described in Supplementary methods.

### Frontier analysis

To evaluate the relationship between the burden of NALC and sociodemographic development, we employed frontier analysis as a quantitative methodology. This approach was used to identify the lowest potentially achievable ASDR based on a country’s SDI. The SDI serves as a composite measure of development, incorporating indicators such as income per capita, educational attainment, and fertility rates. The methodology of the frontier analysis is further described in Supplementary methods.

### Prediction analysis

To project the future burden of NALC deaths and ASDR from 2021 to 2045, we employed the Nordpred model, which uses an age-period-cohort regression framework. The model applies a Poisson log-link function to estimate future mortality trends based on historical data, adjusting for the effects of age, period, and birth cohort (22, 23). This approach assumes that the number of deaths follows a Poisson distribution and incorporates non-linear trends through the log-link function. Using country-specific historical data on NALC deaths and ASDR, we predicted both the number of deaths and ASDR from 2021 to 2045. The model adjusts for changes in population size, age structure, and other demographic factors over time, providing robust projections of disease burden. Percentage changes in predicted deaths and ASDR were calculated to assess future trends and inform public health planning (22).

All data analyses were conducted using R (version 4.3.1), Stata (version 16.0), and several R packages were utilized to analyze and visualize the GBD data. Key packages included ggplot2 (for data visualization), easyGBDR (for working with GBD data), and ggsci (for scientific color palettes). We also used data. Table and tidytable for efficient data handling, dplyr and tidyr for data manipulation and reshaping, splines for fitting non-linear regression models, and patchwork for combining multiple plots into composite figures. These packages facilitated efficient and effective data processing and visualization throughout the study. RStata was used to integrate Stata commands within the R environment.

## Results

### Trends in deaths attributable to NALC in 204 countries and territories from 1990 to 2021

**Figure 1:**
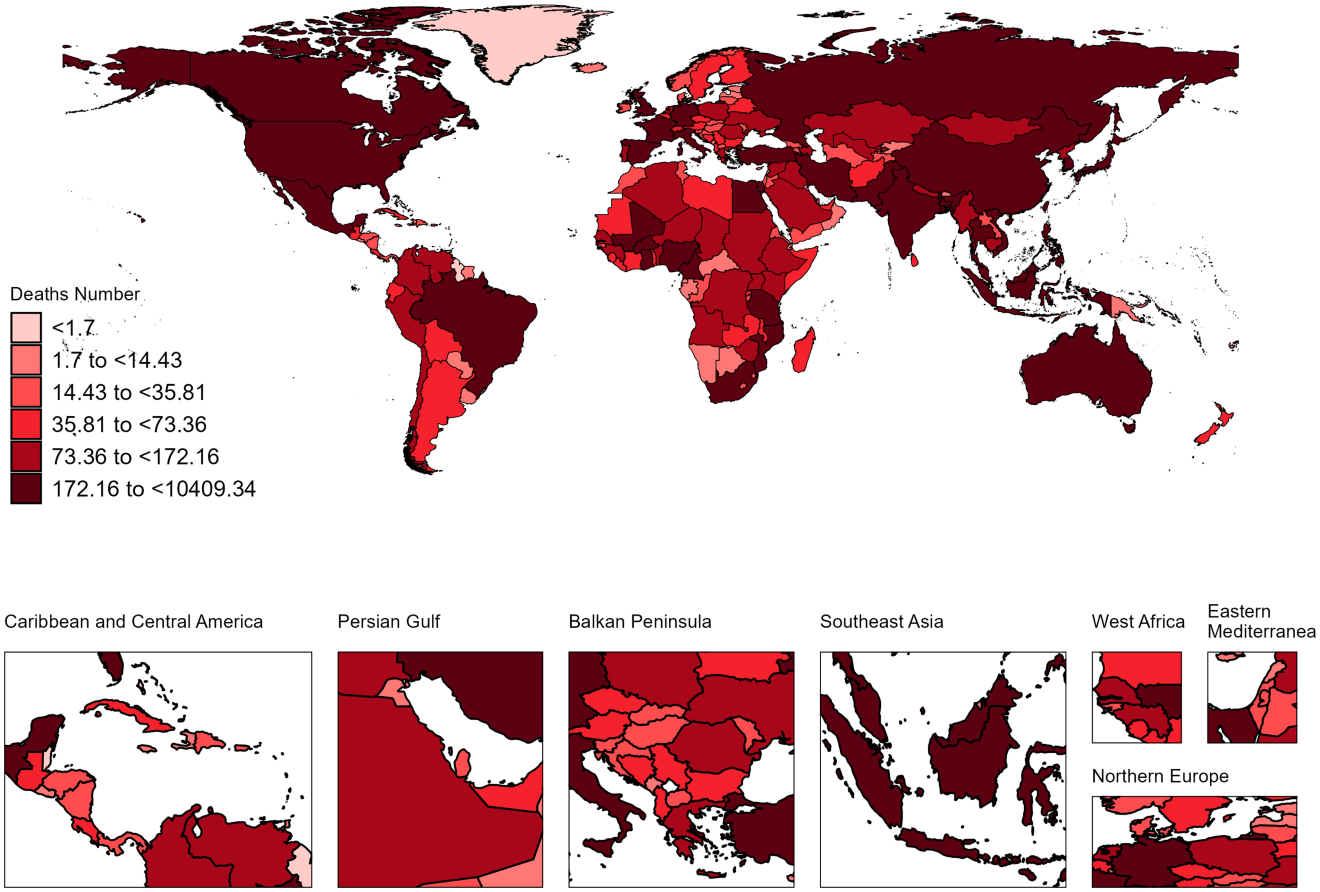
The absolute deaths of NALC in 204 countries and territories in 2021.

**Figure 2:**
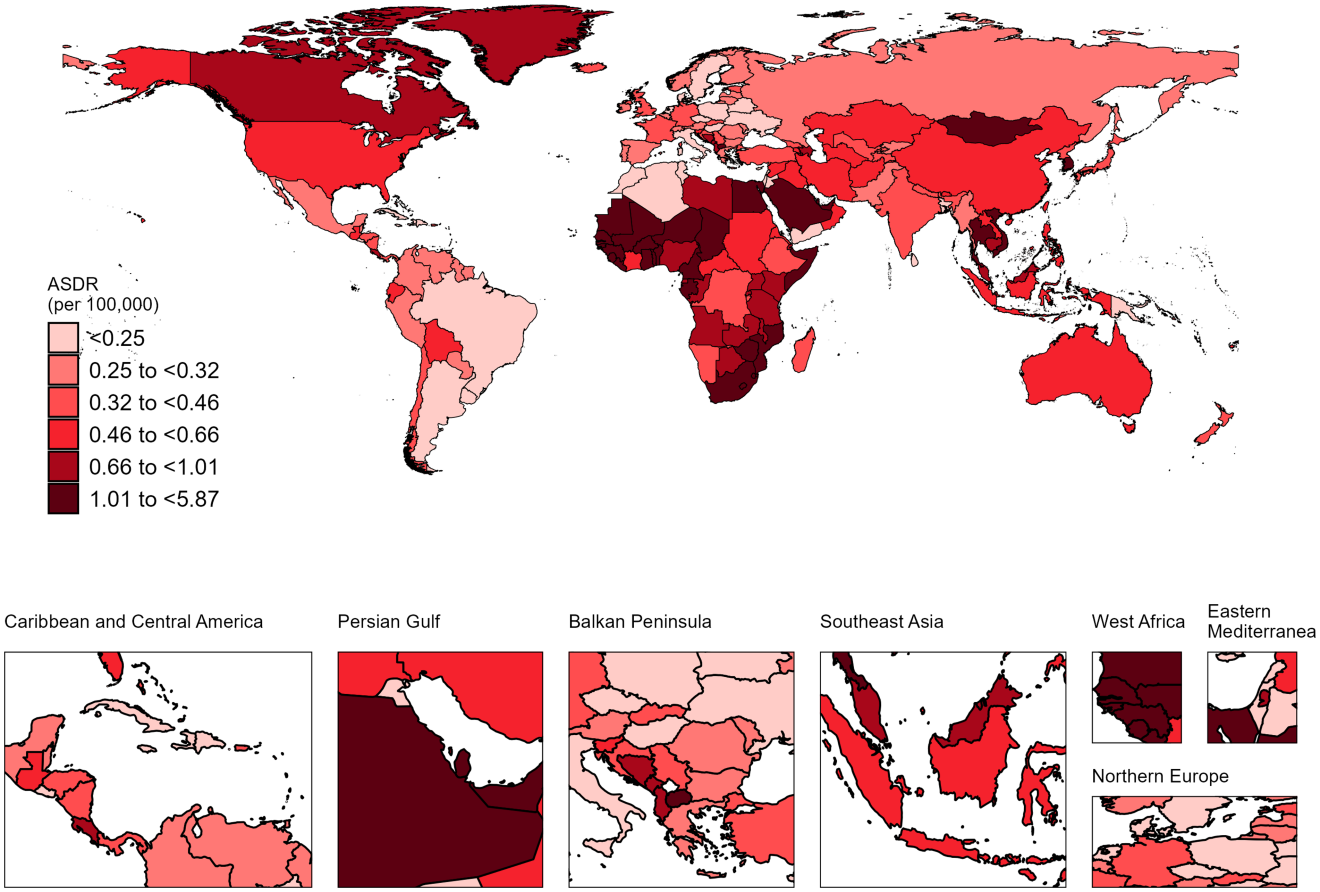
The ASDR of NALC in 204 countries and territories in 2021.

In 2021, the countries with the highest number of NALC cases were China with 10,409 cases (95% UI: 8,036 to 13,180) and an ASDR of 0.51 (95% UI: 0.39–0.64), followed by India with 4,735 cases (95% UI: 3,969 to 5,678) and an ASDR of 0.41 per 100,000 (95% UI: 0.34–0.49). In terms of ASDR, Mongolia had the highest ASDR at 5.87 per 100,000 (95% UI: 3.61–9.12) with 118 cases (95% UI: 73–183), followed by Gambia, which had an ASDR of 3.99 per 100,000 (95% UI: 2.18 to 6.40) and 38 cases (95% UI: 22–60). On the other hand, Morocco had the lowest ASDR at 0.06 per 100,000 (95% UI: 0.04–0.09), with 21 cases (95% UI: 13 to 31) (Figures 1, 2; Supplementary Table S3).

**Figure 3:**
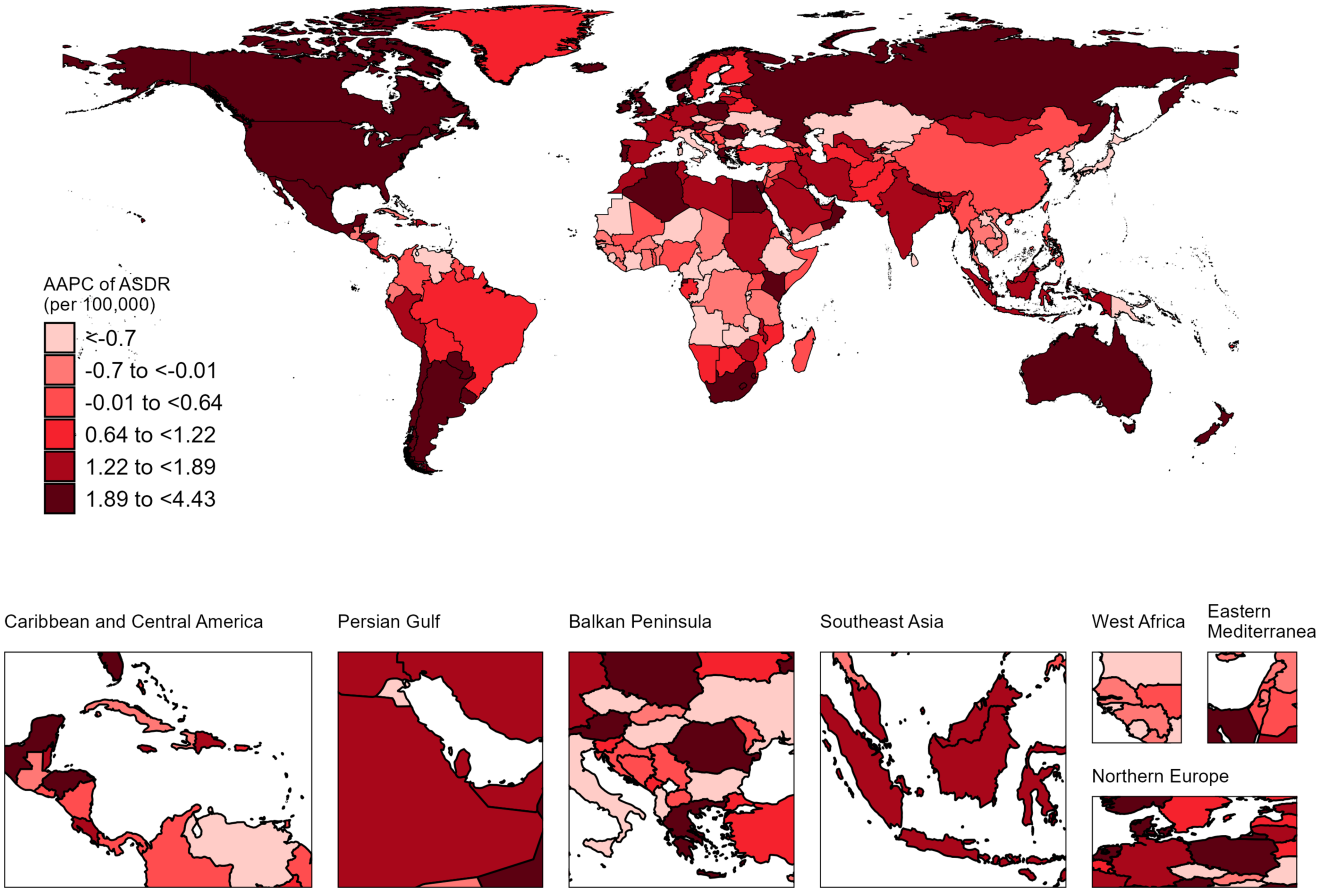
The AAPC of NALC ASDR in 204 countries and territories, 1990–2021.

Australia exhibited the largest increase in ASDR for NALC, with an AAPC of 4.43% (95% CI: 3.94–4.92) from 1990 to 2021. Conversely, Mauritius recorded the most substantial decline, with an AAPC of −4.52% (95% CI: −7.49 to −1.46) over the same time frame (Figure 3; Supplementary Table S3).

### Trends in global deaths attributable to NALC from 1990 to 2021

**Figure 4:**
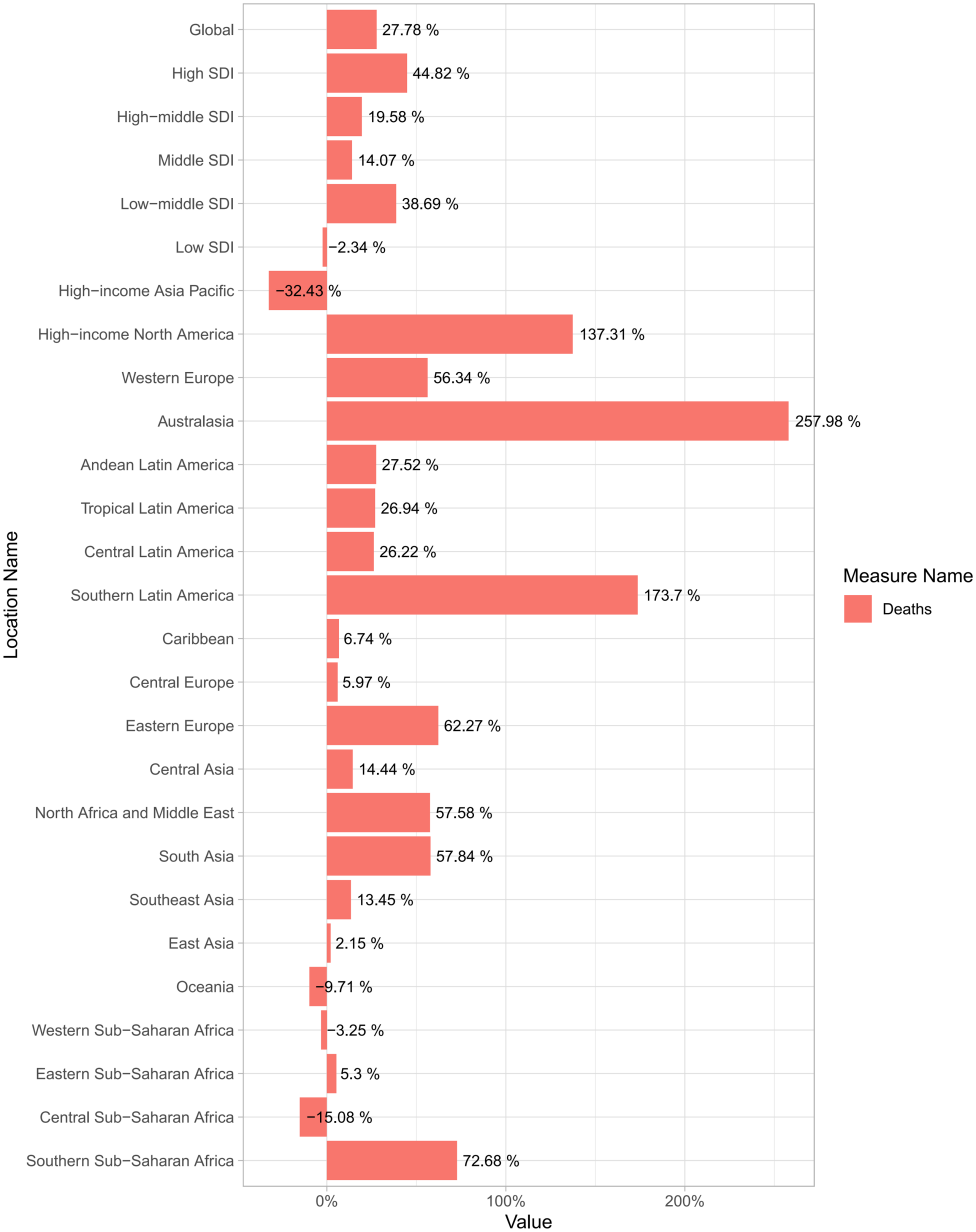
Percentage change in NALC deaths from 1990 to 2021: global, across five SDI regions, and 21 GBD regions. SDI, Socio-demographic index.

From 1990 to 2021, the global burden of deaths associated with NALC showed a notable increase, with a percentage change of approximately 27.78% (Figure 4). In 1990, the total number of deaths was 14,675 (95% UI: 11,621–18,159), rising to 39,074 (95% UI: 31,718–47,007) by 2019, and further increasing to 40,925 (95% UI: 32,961–49,610) by 2021. Similarly, the ASDR demonstrated an upward trend over the same period. In 1990, the global ASDR was 0.38 per 100,000 population (95% UI: 0.30–0.47), which increased to 0.48 per 100,000 (95% UI: 0.39–0.58) by 2019 and remained at the same level in 2021 (Table 1; Supplementary Table S4).

**Table 1:** Global and regional NALC deaths and age-standardized death rates (ASDR) in 1990 and 2021, with average annual percentage changes (AAPCs) from 1990 to 2021 across 21 GBD and 5 SDI regions.

Location	1990 ASDR (95% UI)			2019 ASDR (95% UI)			2021 ASDR (95% UI)			1990–2021 AAPC (95% CI)		
	Both	Female	Male	Both	Female	Male	Both	Female	Male	Both	Female	Male
Global	0.38 (0.3–0.47)	0.36 (0.28–0.46)	0.39 (0.31–0.49)	0.48 (0.39–0.58)	0.45 (0.36–0.54)	0.52 (0.41–0.64)	0.48 (0.39–0.58)	0.44 (0.36–0.54)	0.52 (0.41–0.64)	0.82 (0.72–0.92)	0.74 (0.63–0.84)	0.92 (0.82–1.02)
Andean Latin America	0.29 (0.2–0.41)	0.43 (0.29–0.59)	0.14 (0.09–0.21)	0.36 (0.24–0.51)	0.50 (0.34–0.71)	0.20 (0.13–0.30)	0.37 (0.24–0.53)	0.52 (0.34–0.75)	0.2 (0.13–0.3)	0.75 (0.22–1.28)	0.59 (−0.06–1.25)	1.2 (0.73–1.67)
Australasia	0.15 (0.11–0.2)	0.13 (0.09–0.17)	0.18 (0.13–0.26)	0.53 (0.39–0.73)	0.47 (0.34–0.65)	0.60 (0.42–0.83)	0.55 (0.39–0.74)	0.48 (0.34–0.66)	0.62 (0.42–0.84)	4.22 (3.89–4.56)	4.36 (3.77–4.94)	4.04 (3.46–4.62)
Caribbean	0.14 (0.12–0.16)	0.12 (0.1–0.15)	0.17 (0.14–0.21)	0.22 (0.15–0.30)	0.22 (0.15–0.31)	0.21 (0.15–0.30)	0.22 (0.15–0.31)	0.23 (0.15–0.32)	0.22 (0.15–0.31)	0.18 (−0.43–0.79)	−0.3 (−0.8–0.21)	0.94 (0.53–1.36)
Central Asia	0.76 (0.52–1.1)	0.97 (0.68–1.37)	0.55 (0.33–0.88)	0.61 (0.41–0.86)	0.62 (0.42–0.87)	0.58 (0.39–0.83)	0.61 (0.41–0.87)	0.63 (0.42–0.9)	0.59 (0.39–0.86)	0.43 (0.2–0.65)	0.68 (0.42–0.94)	−0.04 (−0.43–0.35)
Central Europe	0.71 (0.56–0.91)	0.59 (0.45–0.77)	0.84 (0.67–1.06)	0.29 (0.21–0.38)	0.27 (0.20–0.36)	0.31 (0.22–0.42)	0.29 (0.21–0.39)	0.28 (0.2–0.37)	0.31 (0.22–0.43)	0.16 (−0.22–0.53)	−0.26 (−0.67–0.14)	0.79 (0.38–1.2)
Central Latin America	0.26 (0.2–0.34)	0.32 (0.24–0.41)	0.2 (0.15–0.27)	0.31 (0.25–0.39)	0.34 (0.26–0.42)	0.29 (0.23–0.36)	0.33 (0.26–0.42)	0.35 (0.27–0.45)	0.3 (0.23–0.39)	0.8 (0.29–1.32)	0.29 (−0.16–0.74)	1.28 (0.4–2.17)
Central Sub-Saharan Africa	0.54 (0.21–1.23)	0.57 (0.21–1.42)	0.49 (0.18–1.2)	0.44 (0.17–1.05)	0.48 (0.17–1.24)	0.38 (0.15–0.94)	0.46 (0.18–1.12)	0.49 (0.18–1.26)	0.4 (0.15–0.98)	−0.55 (−0.63 to −0.47)	−0.5 (−0.56–−0.44)	−0.73 (−0.92–−0.53)
East Asia	0.5 (0.4–0.61)	0.47 (0.36–0.59)	0.53 (0.42–0.67)	0.55 (0.43–0.68)	0.51 (0.38–0.65)	0.60 (0.44–0.79)	0.51 (0.4–0.64)	0.46 (0.35–0.6)	0.58 (0.42–0.78)	0.16 (−0.34–0.66)	−0.02 (−0.5–0.45)	0.34 (−0.08–0.77)
Eastern Europe	0.28 (0.19–0.38)	0.29 (0.2–0.4)	0.24 (0.17–0.35)	0.22 (0.18–0.25)	0.18 (0.15–0.21)	0.27 (0.22–0.31)	0.23 (0.19–0.27)	0.19 (0.16–0.23)	0.28 (0.24–0.34)	1.62 (0.86–2.38)	1.49 (0.78–2.2)	1.56 (1.09–2.03)
Eastern Sub-Saharan Africa	0.21 (0.14–0.29)	0.26 (0.17–0.35)	0.16 (0.11–0.23)	0.79 (0.54–1.08)	0.97 (0.69–1.32)	0.57 (0.34–0.92)	0.8 (0.55–1.11)	0.99 (0.69–1.34)	0.58 (0.35–0.94)	0.16 (0.08–0.24)	0.06 (−0.01–0.13)	0.12 (−0.08–0.32)
High SDI	0.3 (0.24–0.38)	0.26 (0.2–0.32)	0.35 (0.28–0.45)	0.43 (0.34–0.53)	0.37 (0.29–0.46)	0.50 (0.40–0.62)	0.44 (0.35–0.55)	0.38 (0.29–0.47)	0.51 (0.4–0.64)	1.15 (0.86–1.44)	1.17 (0.93–1.42)	1.16 (0.79–1.53)
High-income Asia Pacific	0.54 (0.37–0.76)	0.5 (0.34–0.69)	0.59 (0.41–0.85)	0.46 (0.36–0.59)	0.39 (0.29–0.51)	0.54 (0.42–0.70)	0.48 (0.37–0.62)	0.4 (0.29–0.54)	0.56 (0.44–0.75)	−1.27 (−1.54–−0.99)	−1.27 (−1.59–−0.94)	−1.27 (−1.61–−0.93)
High-income North America	0.21 (0.18–0.24)	0.18 (0.15–0.2)	0.25 (0.21–0.29)	0.49 (0.40–0.58)	0.40 (0.34–0.47)	0.58 (0.49–0.69)	0.49 (0.41–0.58)	0.4 (0.33–0.48)	0.59 (0.49–0.7)	2.81 (2.64–2.97)	2.7 (2.53–2.87)	2.84 (2.6–3.09)
Low SDI	0.7 (0.44–1.09)	0.8 (0.49–1.32)	0.6 (0.37–0.9)	0.67 (0.47–0.92)	0.79 (0.56–1.10)	0.54 (0.36–0.76)	0.68 (0.48–0.94)	0.81 (0.57–1.11)	0.54 (0.37–0.76)	−0.09 (−0.17–−0.01)	0.02 (to0.1–0.14)	−0.32 (−0.5–−0.15)
Low-middle SDI	0.36 (0.26 to0.52)	0.4 (0.28–0.6)	0.32 (0.24–0.44)	0.49 (0.39–0.60)	0.50 (0.40–0.62)	0.48 (0.37–0.60)	0.5 (0.4–0.62)	0.51 (0.4–0.64)	0.49 (0.37–0.62)	1.06 (0.92–1.2)	0.79 (0.62–0.96)	1.35 (1.19–1.51)
Middle SDI	0.46 (0.37–0.56)	0.45 (0.35–0.56)	0.46 (0.36–0.57)	0.53 (0.43–0.64)	0.49 (0.40–0.60)	0.58 (0.45–0.73)	0.52 (0.42–0.64)	0.47 (0.38–0.58)	0.58 (0.45–0.73)	0.44 (0.24–0.64)	0.14 (−0.03–0.32)	0.79 (0.58–0.99)
North Africa and Middle East	0.44 (0.27–0.74)	0.49 (0.28–0.92)	0.38 (0.25–0.6)	0.67 (0.48–0.91)	0.65 (0.48–0.87)	0.70 (0.48–0.96)	0.69 (0.47–0.94)	0.65 (0.46to0.88)	0.72 (0.49–1.02)	1.49 (1.24–1.74)	0.92 (0.7–1.15)	2.09 (1.82–2.37)
Oceania	0.39 (0.21–0.8)	0.4 (0.2–0.8)	0.4 (0.22–0.86)	0.35 (0.20–0.60)	0.36 (0.21–0.60)	0.35 (0.19–0.68)	0.36 (0.2–0.62)	0.36 (0.21–0.6)	0.36 (0.19–0.7)	−0.37 (−0.48–−0.26)	−0.34 (−0.46–−0.22)	−0.38 (−0.51–−0.25)
South Asia	0.24 (0.19–0.29)	0.23 (0.18–0.28)	0.25 (0.2–0.31)	0.37 (0.31–0.43)	0.36 (0.30–0.42)	0.38 (0.31–0.45)	0.38 (0.32–0.45)	0.37 (0.3–0.44)	0.39 (0.32–0.49)	1.48 (1.38–1.58)	1.57 (1.44–1.69)	1.43 (1.32–1.53)
Southeast Asia	0.6 (0.44–0.82)	0.55 (0.39–0.74)	0.66 (0.47–0.94)	0.68 (0.49–0.93)	0.58 (0.37–0.81)	0.80 (0.57–1.16)	0.69 (0.48–0.94)	0.57 (0.36–0.83)	0.81 (0.56–1.19)	0.42 (0.3–0.55)	0.13 (0.09–0.18)	0.62 (0.51–0.74)
Southern Latin America	0.07 (0.05–0.1)	0.07 (0.04–0.09)	0.07 (0.05–0.11)	0.18 (0.13–0.26)	0.17 (0.11–0.23)	0.21 (0.14–0.30)	0.19 (0.13–0.27)	0.17 (0.12–0.24)	0.22 (0.14–0.32)	3.3 (2.59–4.01)	3.09 (2.19–3.99)	3.65 (3.03–4.28)
Southern Sub-Saharan Africa	0.67 (0.4–1.01)	0.69 (0.46–1.04)	0.63 (0.26–1.2)	1.11 (0.88–1.36)	0.93 (0.73–1.20)	1.39 (1.05–1.81)	1.15 (0.92–1.42)	0.96 (0.76–1.25)	1.46 (1.09–1.92)	1.75 (1.42–2.08)	1.02 (0.67–1.37)	2.73 (2.56–2.89)
Tropical Latin America	0.13 (0.11–0.15)	0.15 (0.13–0.18)	0.11 (0.09–0.12)	0.16 (0.14–0.19)	0.16 (0.14–0.19)	0.16 (0.14–0.19)	0.17 (0.14–0.19)	0.17 (0.14–0.2)	0.16 (0.14–0.19)	0.74 (0.52–0.96)	0.34 (to0.25–0.93)	1.42 (1.02–1.82)
Western Europe	0.2 (0.15–0.27)	0.18 (0.13–0.23)	0.23 (0.18–0.32)	0.32 (0.23–0.42)	0.28 (0.20–0.36)	0.37 (0.27–0.49)	0.32 (0.23–0.42)	0.28 (0.2–0.36)	0.37 (0.27–0.5)	1.44 (1.18–1.71)	1.44 (1.23–1.65)	1.42 (1.13–1.72)
Western Sub-Saharan Africa	1.27 (0.72–2.16)	1.46 (0.8–2.68)	1.07 (0.61–1.71)	1.20 (0.88–1.60)	1.42 (1.05–1.89)	0.95 (0.68–1.33)	1.23 (0.91–1.65)	1.46 (1.06–1.94)	0.97 (0.7–1.35)	−0.12 (−0.19–−0.05)	−0.01 (−0.1–0.07)	−0.33 (−0.43–−0.23)

From 1990 to 2021, deaths from NALC have increased significantly for both men and women globally. While the rise in deaths is more pronounced among women, men also show a substantial upward trend over this period (Supplementary Table S5). Correspondingly, the AAPC in the ASDR for NALC has shown a significant increase for both genders. Specifically, for women, the AAPC is 0.74 (95% CI: 0.63–0.84), indicating a steady annual rise, whereas for men, the AAPC is 0.92 (95% CI: 0.82–1.02) (Table 1). The ASDR for NALC exhibited distinct trends over different time periods. From 1990 to 2000, the ASDR increased significantly [APC 1.54% (95% CI: 1.42–1.66, *p* < 0.001)]. From 2000 to 2006, there was a slight decline in the ASDR [APC −0.42% (95% CI: −0.76 to −0.08, *p* = 0.018)]. From 2006 to 2016, the ASDR rose again [APC 1.36% (95% CI: 1.22–1.5, *p* < 0.001)]. In contrast, from 2016 to 2021, the ASDR decreased marginally [APC −0.21% (95% CI: −0.55–0.13, *p* = 0.21)], though this change was not statistically significant (Supplementary Table S6).

### Trends in global deaths attributable to NALC in 2021 across different age groups

**Figure 5:**
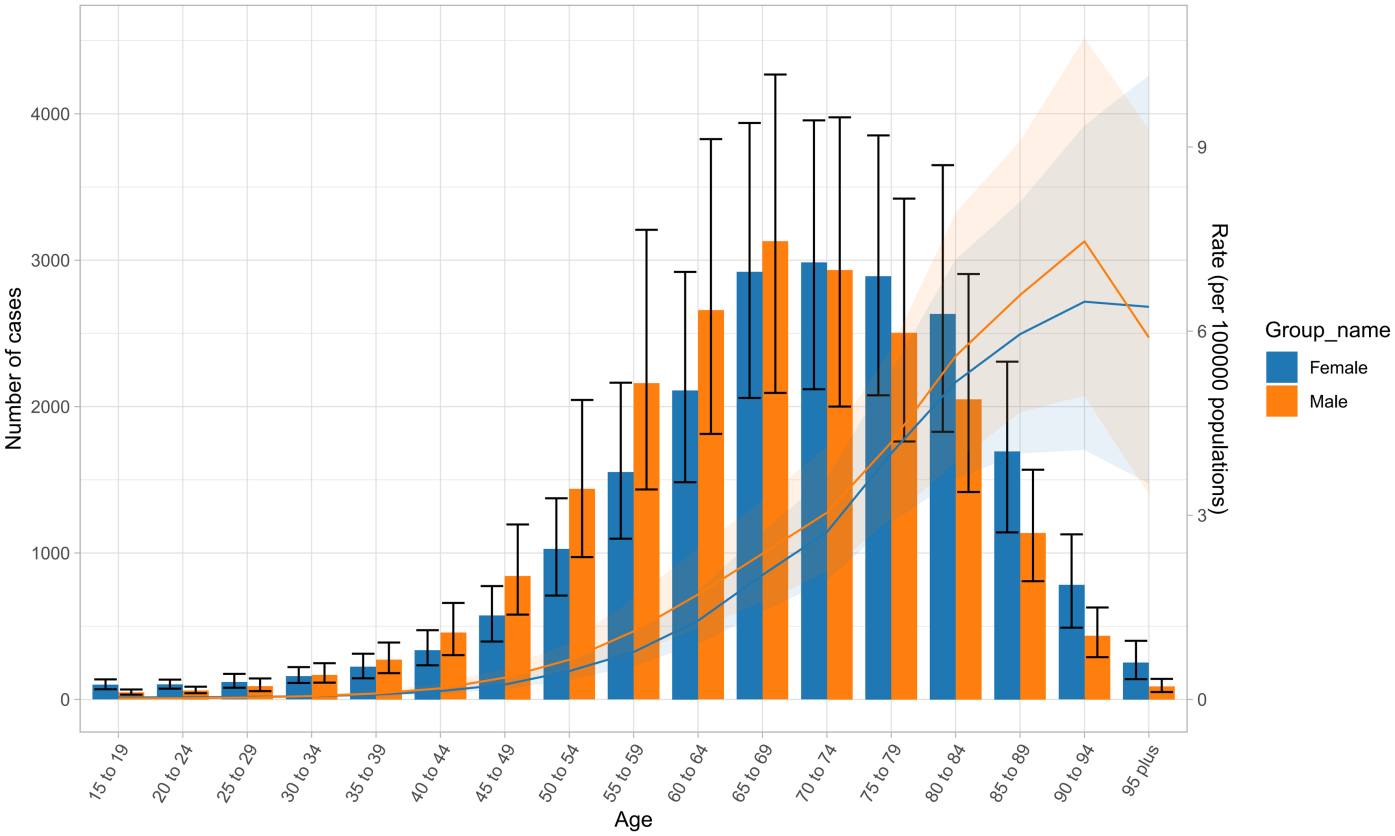
Trends in the number of NALC cases and ASDR across different age groups and genders in 2021.

The number of NALC cases in 2021 showed distinct age and gender-specific trends. For men, the peak occurred in the 65–69 age group, while for women, it was in the 70–74 age group. Before the age of 70, men consistently had more cases than women; however, this trend reversed after 70, with women surpassing men as age increased. Similarly, the ASDR steadily rose with age, with minimal gender differences overall. Notably, in the 90–94 age group, the ASDR for men was significantly higher than for women, whereas in the 95 plus age group, women exhibited a markedly higher ASDR than men (Figure 5).

### Trends in deaths attributable to NALC in 21 GBD regions from 1990 to 2021

In 2021, among the 21 regions analyzed for NALC, East Asia had the highest number of cases, with 10,936 cases (95% UI: 8,458–13,798), while Oceania experienced the lowest burden, with 25 cases (95% UI: 14–46). In terms of the ASDR, Western Sub-Saharan Africa recorded the highest ASDR at 1.23 (95% UI: 0.91–1.65), in contrast, Tropical Latin America had the lowest ASDR at 0.17 (95% UI: 0.14–0.19) in 2021 (Table 1).

From 1990 to 2021, the global burden of deaths associated with NALC across the 21 GBD regions exhibited significant regional variations. For example, Australasia, Southern Latin America, and High-income North America were the top three regions with the highest positive growth in deaths. In contrast, only four regions—High-income Asia Pacific, Central Sub-Saharan Africa, Oceania, and Western Sub-Saharan Africa—experienced negative growth during this period (Figure 4).

Among the 21 regions analyzed, Australasia exhibited the most rapid increase in the ASDR related to NALC, with an AAPC of 4.22% (95% CI: 3.89–4.56) from1990 to 2021. In contrast, High-income Asia Pacific demonstrated the greatest reduction in ASDR during the same period, with an AAPC of −1.27% (95% CI: −1.68 to −0.86) (Table 1).

### Trends in deaths attributable to NALC in 5 SDI regions from 1990 to 2021

**Figure 6:**
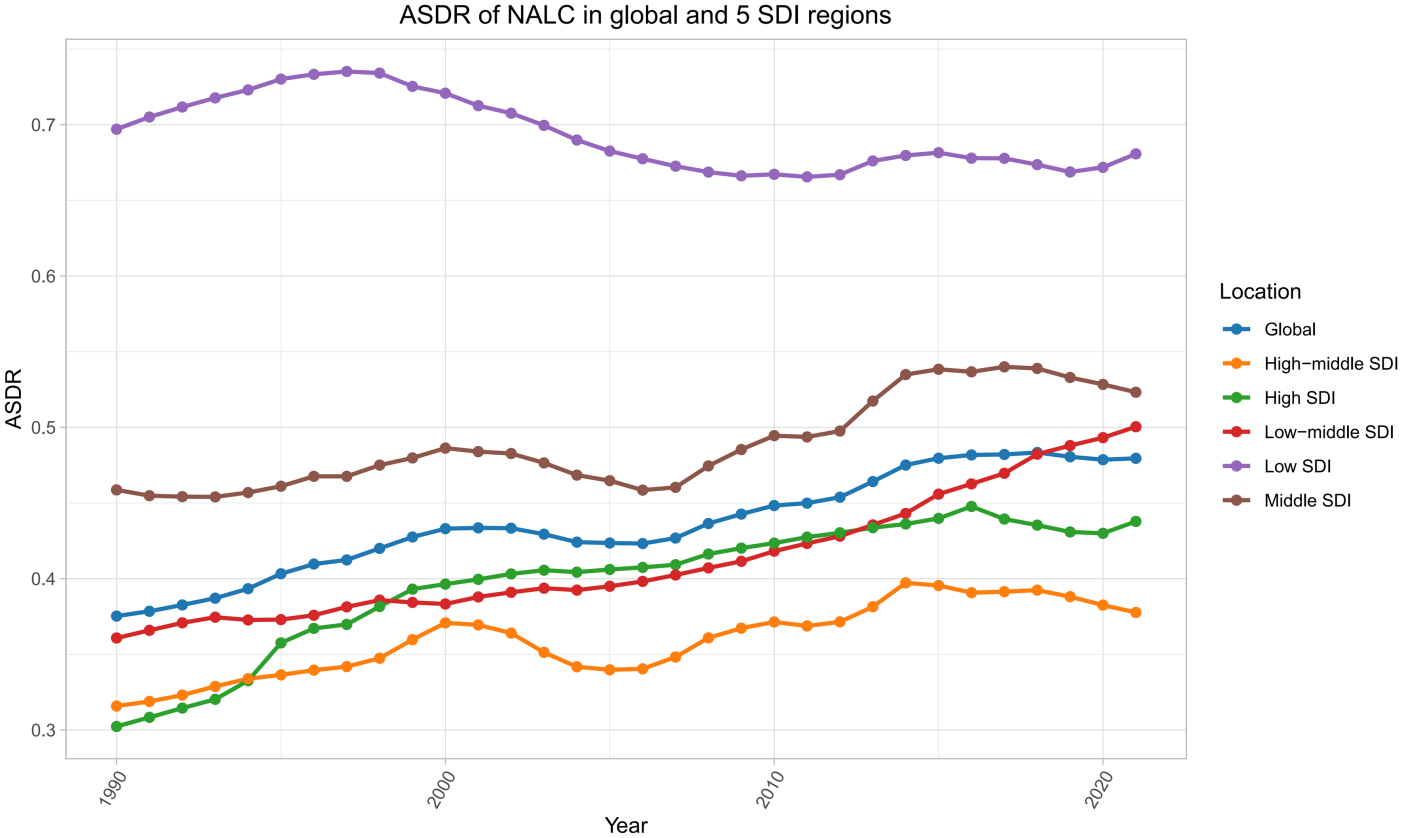
Trends in the ASDR of NALC across global and five SDI regions from 1990 to 2021. SDI, Socio-demographic index.

Among the five SDI regions, the ASDR of NALC shows a gradual increase from 1990 to 2021, with the Low SDI region consistently maintaining the highest ASDR throughout the period (Figure 6). In 2021, Middle SDI had the highest number of cases, with 13,613 cases (95% UI: 10,818–16,683), while Low SDI had the lowest number of cases, with 3,222 cases (95% UI: 2,283–4,455). In terms of ASDR, Low SDI had the highest ASDR at 0.68 per 100,000 (95% UI: 0.48–0.94), whereas High-middle SDI recorded the lowest ASDR at 0.38 per 100,000 (95% UI: 0.30–0.46) (Table 1).

Among the five SDI regions, High SDI experienced the fastest growth in the ASDR related to NALC, with an AAPC of 1.15% (95% CI: 0.86–1.44) from 1990 to 2021. In contrast, Low SDI was the only region to show a decline, with an AAPC of −0.09% (95% CI: −0.17 to −0.01) over the same period (Table 1).

### Decomposition analysis of NALC burden

**Figure 7:**
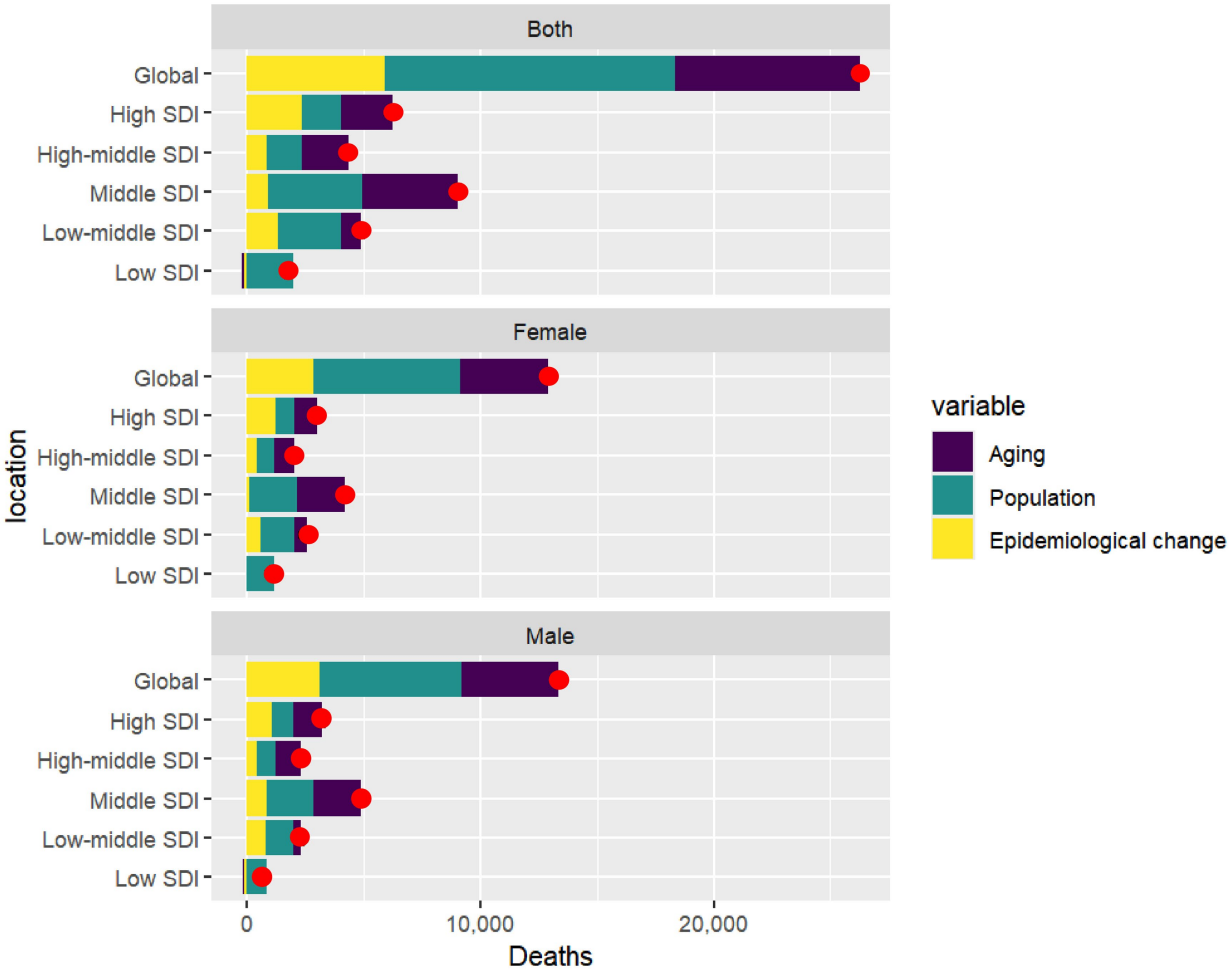
Gender-based decomposition analysis of NALC deaths across global and five SDI regions from 1990 to 2021. The red dot represents the overall change value of population aging, population growth, and epidemiological change. SDI, socio-demographic index.

The global burden of deaths from NALC is influenced by three major factors: population aging, population growth, and epidemiological change, with variations between men and women. Population aging contributes to 30.14% of the global deaths, with 29.04% in women and 31.24% in men. Population growth has the most significant impact, contributing 47.38% globally, with 49.16% in women and 45.65% in men. Epidemiological change accounts for 22.49% of the global death toll, with 21.81% in women and 23.12% in men (Figure 7, Supplementary Table S7).

From 1990 to 2021, the overall difference in deaths due to NALC in the Middle SDI region was 9,020, representing the largest change among the five SDI regions, with women accounting for 4,166 deaths (46.19%) and men contributing 4,853 deaths (53.81%). Population aging had a significant impact in the High-middle SDI region, contributing 45.37% to the increase in deaths, with women contributing 44.02% and men 48.00%. In contrast, in the Low SDI region, population aging had a negative contribution, with an overall reduction of −6.24%, where women contributed −2.10% and men −11.81%. Population growth played a particularly important role in the Low SDI region, leading to a 112.43% increase in deaths, with women accounting for 103.55% and men for 128.56%. Epidemiological change contributed to a 37.82% increase in NALC deaths in the High SDI region, with women contributing 40.72% and men 33.44%. Conversely, in the Low SDI region, epidemiological change led to a −6.19% overall decrease in deaths, with a reduction of −1.45% among women and a more substantial decline of −16.75% among men (Figure 7, Supplementary Table S7).

From 1990 to 2021, the overall difference in deaths due to NALC in East Asia was 6,644, with women accounting for 3,098 deaths (46.63%) and men contributing 3,546 deaths (53.37%), making it the region with the largest change among the 21 GBD regions. Regarding the impact of population aging, the High-income Asia Pacific region, which experienced the greatest increase in deaths, saw an overall contribution of 125.16%, with women contributing 115.04% and men 142.46%. In Central Europe, which had the second-largest increase, population aging contributed 83.00% overall, with women contributing 108.40% and men 61.06%. In contrast, three regions in Sub-Saharan Africa experienced negative contributions from population aging. In Western Sub-Saharan Africa, aging reduced deaths by −22.64%, with women contributing −21.35% and men −26.01%. In Central Sub-Saharan Africa, there was a reduction of −7.57%, with women contributing −0.78% and men −16.64%. Lastly, in Eastern Sub-Saharan Africa, aging contributed a decline of −8.60%, with women contributing −1.93% and men −15.81%. In Central Sub-Saharan Africa, there was a reduction of −7.57%, with women contributing −0.78% and men −16.64%. Population growth had a significant impact on NALC, particularly in Sub-Saharan Africa. The region most affected was Central Sub-Saharan Africa, where population growth contributed 139.83% overall, with women contributing 123.97% and men 167.55%. The second most impacted region was Western Sub-Saharan Africa, where population growth accounted for an increase of 132.37%, with women contributing 126.23% and men 147.31%. In third place, Eastern Sub-Saharan Africa saw an overall contribution of 105.07%, with women contributing 103.41% and men 107.74%. In contrast, Eastern Europe experienced a negative contribution from population growth, with an overall impact of −3.04%, as women contributed −3.30% and men −2.76%. Epidemiological change also played a critical role, contributing negatively to NALC in several regions. In High-income Asia Pacific, epidemiological change led to a reduction of −55.58%, with women contributing −41.63% and men −78.05%. In Central Sub-Saharan Africa, the overall reduction was −32.26%, with women contributing −23.19% and men −50.91%. For Oceania, epidemiological change resulted in an overall reduction of −14.42%, with women contributing −14.96% and men −14.11%. Lastly, in Western Sub-Saharan Africa, the overall reduction was −9.73%, with women contributing −4.88% and men −21.30%, In East Asia, the overall contribution from epidemiological change was smaller at −1.46%, with women contributing −6.51%, while men saw a slight increase of 3.20% (Supplementary Table S7).

From 1990 to 2021, among 203 countries analyzed for NALC deaths, China exhibited the largest overall NALC deaths with 6,281.15 deaths—2,899.84 (46.17%) among women and 3,381.31 (53.83%) among men. India followed with an increase of 3,571.74 deaths, comprising 1,825.22 deaths (51.10%) among women and 1,746.52 deaths (48.90%) among men. The United States ranked third with an increase of 2,138.76 deaths, where women accounted for 901.51 deaths (42.15%) and men for 1,237.25 deaths (57.85%). Egypt was fourth with an increase of 1,115.93 deaths, including 465.10 deaths (41.68%) among women and 650.83 deaths (58.32%) among men. Thailand recorded the smallest increase among the top five, with 812.20 deaths—326.12 (40.15%) among women and 486.08 (59.85%) among men. In the above five countries, both population aging and population growth contributed positively to the increase in NALC deaths. However, epidemiological changes had a negative impact on such deaths in Thailand and China. In Thailand, these changes led to an overall reduction of 11.6%, with a significant decline of 35.44% among women and a slight increase of 4.25% among men. Similarly, in China, epidemiological changes resulted in an overall reduction of 2.97%, with women experiencing a decline of 8.89% and men seeing a marginal increase of 2.37%. These figures highlight a notable gender disparity in the impact of epidemiological changes in both countries (Supplementary Table S8).

### Frontier analysis of NALC burden

**Figure 8:**
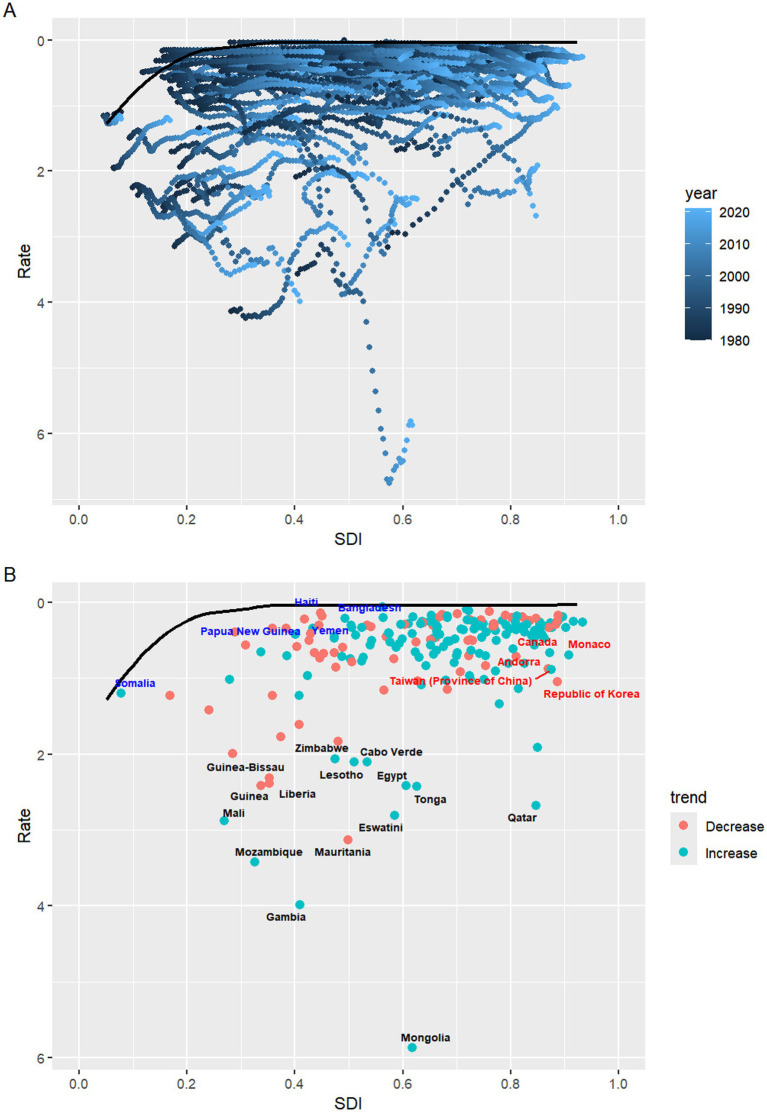
**(A)** Frontier analysis of SDI and NALC-related death rates from 1990 to 2021. **(B)** Frontier analysis of SDI and NALC-related death rates for the year 2021. SDI, socio-demographic index.

The frontier analysis highlights the leading countries or regions that represent the lowest disease burden, defined by their SDI, essentially “pushing the envelope” at the frontier. The effective difference, which indicates the distance from this frontier, reflects the gap between the observed disease burden in a country or region, based on its SDI, and the potential disease burden that could be achieved (24). By comparing actual health outcomes, such as mortality rates, to an optimal “frontier”—representing the best possible outcomes given available resources—this method highlights inefficiencies and identifies areas for improvement. In this analysis, frontier analysis was applied to evaluate deaths related to NALC across various countries. Countries with the largest Effective Difference, such as Mongolia (5.84), Gambia (3.95), Mozambique (3.36), Mauritania (3.09), and Eswatini (2.77), are significantly farther from the frontier. On the other end of the spectrum, countries like Morocco (0.03), Somalia (0.04), Mauritius (0.06), Argentina (0.08), and Ukraine (0.08) exhibit the smallest Effective Difference (Figure 8; Supplementary Table S9).

### Prediction analysis of NALC burden

The predictive analysis provides insights into future trends in mortality and age-standardized death rates based on current data. By projecting these trends, we can better understand how mortality patterns are likely to evolve over time, taking into account factors such as population aging and changes in disease burden. This analysis focuses on the expected changes in both the total number of deaths and the ASDR between 2021 and 2045, highlighting gender-specific trends and the variations across different age groups.

**Figure 9:**
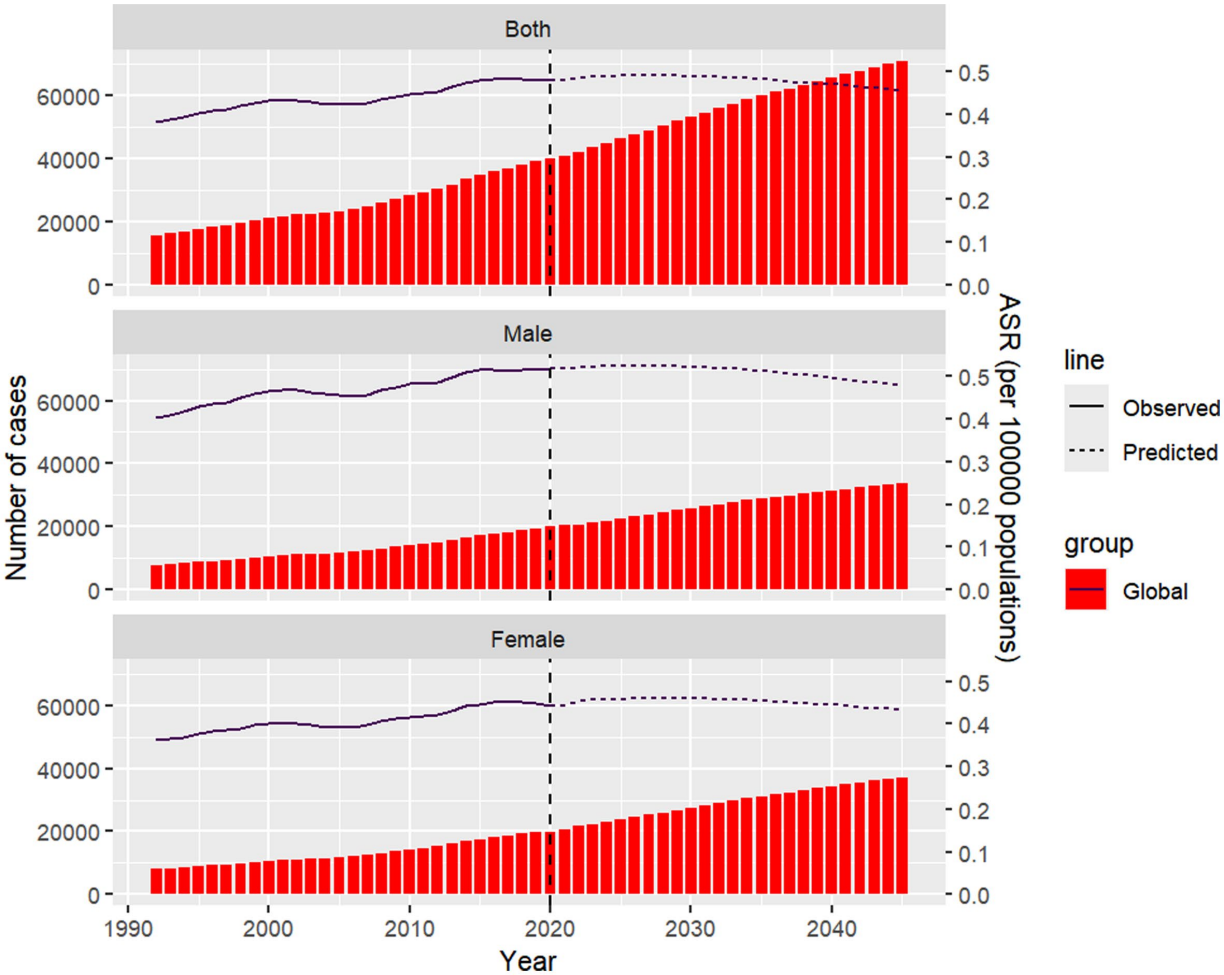
Projected number of death cases and ASDR of NALC by gender globally in 2045. The Nordpred prediction model was used to evaluate the mortality trends of NALC patients in different gender subgroups up to 2045. ASDR, age-standardized death rate; Nordpred, Nordic Cancer Prediction Model.

Based on the prediction analysis, the total number of deaths is projected to significantly increase by 2045 compared to 2021, but the ASDR is expected to decrease. Notably, the increase in deaths among women is expected to surpass that of men. While the ASDR is projected to rise initially, a decline is anticipated by 2045 (Figure 9; Supplementary Table S10). For women, the number of deaths across all age groups is expected to increase by 2045, with a particularly sharp rise in the 80–84 age group. In terms of ASDR, the age group showing an increase is women aged 60 and above (Supplementary Tables S11, 12). For men, death counts are also projected to rise across all age groups by 2045, with a notable increase in the 75–79 age group. ASDR for men is expected to show a slight decline across the 30–94 age range, but the decline is minimal, with the only significant increase occurring in the 95 plus age group (Supplementary Tables S13, 14; Figure 9).

## Discussion

Our comprehensive analysis of global trends in NALC reveals several key observations. First, from 1990 to 2021, NALC-related mortality has increased significantly, both in absolute numbers and ASDR. This upward trend is consistent across both genders, although the increase is slightly more pronounced in men vs. women (0.92 vs. 0.74). Regional disparities in NALC mortality were also significant. East Asia exhibited the highest number of cases, while Western Sub-Saharan Africa had the highest ASDR. Conversely, Oceania reported the lowest number of cases, and Tropical Latin America had the lowest ASDR, underscoring the considerable geographical variation in the burden of NALC. In terms of socioeconomic development, countries classified in the Low SDI region exhibited the highest ASDR, while the High-middle SDI region reported the lowest. The decomposition analysis further highlighted that population growth was the most significant contributor to the rise in NALC deaths globally, accounting for 47.38% of the increase, while epidemiological changes contributed 22.49%. Importantly, population aging played a substantial role in High-middle and High SDI regions but had a negative contribution in Low SDI regions, where population growth dominated the increase in mortality. Finally, our projection analysis suggests that the global burden of NALC-related deaths will continue to rise by 2045, particularly in older age groups, with a more substantial increase in women compared to men. This study provides crucial insights into the regional and demographic variations in NALC mortality, offering a valuable foundation for targeted public health interventions and resource allocation.

In recent years, there has been a growing consensus within the medical community regarding the need to redefine NAFLD to better reflect its underlying pathophysiology. As a result, in 2020, a significant shift in terminology occurred with the proposal of renaming NAFLD to Metabolic Associated Fatty Liver Disease (MAFLD). This new classification was driven by the recognition that NAFLD, as a term, was somewhat limited in its scope, as it emphasized the exclusion of alcohol consumption rather than focusing on the metabolic origins of the disease (25). MAFLD is now seen as a more inclusive term that acknowledges the metabolic risk factors associated with fatty liver disease, such as obesity, type 2 diabetes, and dyslipidemia. This redefinition aligns more closely with the growing body of research that links fatty liver disease to the global rise in metabolic disorders, making the condition more relevant in today’s healthcare landscape. Additionally, the MAFLD criteria emphasize the importance of diagnosing the disease in individuals who exhibit clinical features of metabolic syndrome, broadening the diagnostic scope beyond alcohol-related and viral etiologies (26).

According to a meta-analysis that systematically compared the epidemiological features of MAFLD and NAFLD, the prevalence of MAFLD in the general population was 33.0%, while NAFLD was found to have a prevalence of 29.1% (27). Based on a recent study analyzing the prevalence and characteristics of NAFLD and MAFLD in an Asian population, it was found that the prevalence rates for these conditions were 36.26% for NAFLD and 53.03% for MAFLD. The study showed that the prevalence of MAFLD was significantly higher than NAFLD. Additionally, patients with MAFLD exhibited distinct differences in body composition, such as older age, higher body mass index, and higher levels of alanine aminotransferase (ALT), triglycerides, and waist circumference compared to NAFLD patients (28).

The global epidemiology of HCC is shifting, with an anticipated rise in incidence over the next 30 years. While cases linked to hepatitis B and C are declining, the prevalence of HCC related to NAFLD/MAFLD is increasing significantly. This surge in MAFLD cases is driving the overall increase in HCC incidence, despite the reduction in hepatitis-related HCC (29). According to a meta-analysis of 22 studies, the global prevalence of HCC attributable to MAFLD is significant. The total prevalence of MAFLD in HCC cases was found to be 48.7% (95% CI: 34.5–63.0%), with 12.4% (95% CI: 8.3–17.3%) of cases having MAFLD as the sole liver disease (30).

Initially, due to unclear diagnostic criteria for NASH, a significant portion of NAFLD cases may have been misclassified as NASH, leading to an overestimation of the disease burden associated with NASH. However, since the early 20th century, the definition and diagnosis of NASH have been significantly improved with the establishment of standardized criteria for histological changes, especially in clinical trials of NAFLD. In 2005, the NASH Clinical Research Network (NIH NASH CRN) introduced the NAFLD Activity Score (NAS) system, which provided a more precise and standardized method for evaluating NAFLD in clinical studies (31). At the same time, advancements in medical imaging technologies, such as transient elastography (TE) and magnetic resonance elastography (MRE), have allowed for more accurate assessment of liver stiffness, a potential surrogate marker of liver fibrosis (32). The continuous progress in these diagnostic tools may explain the sudden decline in NALC mortality rates and ASDR observed in some middle-income countries after 2000, as early diagnosis and monitoring of the disease improved.

In 2021, China had the highest number of deaths from NALC, although its ASDR was not the highest. This may be attributed to obesity and metabolic factors being the primary risk factors (33, 34). With China’s economic growth, the dietary structure in more developed regions has gradually transitioned from a plant-based Eastern diet to a Western, animal-based diet. However, improvements in health awareness have not kept up with these dietary changes (35, 36). China now has the highest number of individuals affected by overweight or obesity worldwide (37). Over the past century, the prevalence of these conditions has followed an increasing-decreasing-increasing trend. Between 1993 and 2021, the overweight rate surged by 2.68 times, from 20.65 to 55.33% (38).

Our decomposition analysis identified population aging, population growth, and epidemiological changes as key contributors, with varying effects across regions and genders. Population aging contributed 30.14% globally to NALC mortality, particularly in high-middle SDI regions (45.37%). In areas like Central Europe and the High-Income Asia Pacific, aging increases susceptibility to NASH and related liver complications, underscoring the need for targeted interventions. In contrast, in low SDI regions, aging contributed negatively (−6.24%) due to insufficient healthcare resources and under diagnosis, highlighting the need for improved healthcare infrastructure and awareness programs. Population growth was the largest contributor, accounting for 47.38% of the global NALC burden, especially in low SDI regions, where it caused a 112.43% increase in deaths. Rapid population growth in regions like Central Sub-Saharan Africa has exacerbated the burden, particularly among women (103.55%).

In a multicenter study in Australia, MAFLD was found to be the most common underlying liver disease in HCC patients, particularly in older adults (73 vs. 67 years in non-MAFLD). The study highlighted that metabolic syndrome was more prevalent in the MAFLD group, emphasizing the need for targeted HCC prevention and screening strategies for older populations (39). In a nationwide cohort study of HCC patients in the United States (2003–2021), it was found that the mean age of HCC patients was 68.3 years, with those having NAFLD being the oldest (71.1 years). The study also revealed that NAFLD patients had the highest Charlson Comorbidity Index (CCI), indicating a greater burden of comorbid conditions. Furthermore, HCC patients in the more recent 2014–2021 cohort were significantly older, and the survival rate during this period was lower compared to the earlier 2003–2013 cohort, highlighting the impact of aging on the prognosis of HCC patients (40).

While the large population sizes in India contribute significantly to the high absolute number of NALC cases in these countries, it is essential to interpret these figures within the context of ASDR. Despite the substantial number of cases in both nations, their ASDR are not among the highest globally. This suggests that, although the total burden of NALC is considerable in these countries, the relative mortality risk, adjusted for age distribution, is not as elevated as in regions with smaller populations but higher ASDR.

According to our frontier analysis of NALC burden, the large Effective Differences highlight significant untapped opportunities for improving health outcomes through targeted interventions and policy reforms. Even small changes in the allocation of resources or enhancements in healthcare system management could lead to considerable progress, especially in countries where healthcare inefficiencies are more pronounced. In contrast, countries with the smallest Effective Differences, despite facing various socioeconomic challenges, have achieved disease burdens relatively close to the frontier. Consequently, their potential for further reducing the disease burden is more limited compared to countries with larger disparities. Nonetheless, sustained attention to healthcare policies and efforts to maintain existing efficiencies will be crucial for preserving their favorable health outcomes.

In parallel, epidemiological research indicates that the global incidence of NALC is expected to increase significantly in the coming decade. This trend is primarily driven by the rising prevalence of obesity and metabolic syndrome. In regions such as the United States, Europe, and East Asia, NAFLD is anticipated to become a leading cause of HCC as populations age and rates of obesity and insulin resistance continue to climb. By 2030, NALC is projected to account for a substantial proportion of all HCC cases, especially in developed countries. These findings highlight the urgent need for public health interventions that target metabolic risk factors like obesity and insulin resistance to reduce the future global burden of NAFLD and HCC (41).

This study has several limitations. First, the accuracy of mortality data from countries with inadequate vital registration systems, particularly in low-and low-middle SDI regions, may be compromised due to underreporting and misclassification. These issues could introduce bias into our mortality estimates, affecting the reliability of our findings. Despite using advanced statistical methods, the data quality in these regions remains a potential source of bias. Second, our analysis does not distinguish between histopathological subtypes of NALC, such as hepatocellular carcinoma and cholangiocarcinoma, due to the lack of granularity in the GBD 2021 dataset. This limits our understanding of subtype-specific trends. Third, while we attribute the increase in NALC deaths to population growth and aging, we did not account for other factors such as environmental exposures or lifestyle changes, which could also influence mortality trends.

## Data Availability

The original contributions presented in the study are included in the article/Supplementary material, further inquiries can be directed to the corresponding authors.

## Footnotes

^1^ https://vizhub.healthdata.org/gbd-results/

^2^ https://ghdx.healthdata.org/search/site/SDI

## Glossary

NAFLD: Non-alcoholic fatty liver disease
T2DM: type 2 diabetes mellitus
NAFL: non-alcoholic fatty liver
NASH: non-alcoholic steatohepatitis
HCC: hepatocellular carcinoma
HCV: hepatitis C virus
NALC: NASH-associated liver cancer
HFPG: high fasting plasma glucose
GBD: Global burden of disease
SDI: sociodemographic index
GHDx: Global health data exchange
CODEm: Cause of death ensemble model
95%UI: 95% uncertainty interval
ASR: age-standardized rate
ASDR: age-standardized death rate
APC: annual percent change
95%CI: 95% confidence interval
AAPC: average annual percent change
WHO: World Health Organization
MAFLD: Metabolic associated fatty liver disease
NIH NASH CRN: NASH clinical research network
NAS: NAFLD activity score
TE: transient elastography
MRE: magnetic resonance elastography
CCI: Charlson comorbidity index
