# Supplementary material for: Global burden and international disparities in NASH-associated liver Cancer: mortality trends (1990–2021) and future projections to 2045
Source: Front Public Health. 2025 Feb 14;13:1527328. doi: 10.3389/fpubh.2025.1527328 (PMC11868124; doi:10.3389/fpubh.2025.1527328)
Supplement: Supplementary file 1 [file Data_Sheet_1.pdf]

# Global Burden and International Disparities in NASH-Associated Liver Cancer: Mortality Trends (1990 – 2021) and Future Projections to 2045

## Supplementary material catalogue

Qilong Nie, Yongwen Jiang, Mingyang Li, Qiuyan Liang, Xiaoai Mo, Tengyu Qiu, Qunfang Jiang, Kaizhou Huang, Youqing Xie, Ying Chen, Xiaojun Ma, Jianhong Li, Kaiping Jiang

**Supplementary Methods**

**Table S1. List of International Classification of Diseases (ICD) codes mapped to the Global Burden of Disease cause list for liver cancer mortality data.**

**Table S2 Terms and definitions to Country/Region Groupings Used in This Study to Including SDI Countries and GBD Regions to 21GBD region and 5 SDI region specific distribution**

**Table S3 Absolute deaths and age-standardized death rate (ASDR) of NALC globally and regionally in 1990 and 2021, and their average annual percentage change (AAPC) from 1990 to 2021 across 204 countries and regions.**

**Table S4 Absolute deaths and age-standardized death rate (ASDR) of NALC globally and regionally in 1990 and 2021 across 21 GBD regions and 5 SDI regions.**

**Table S5 Absolute and age-standardized death rate (ASDR) for NALC in men and women globally over 30 years from 1990 to 2021**

**Table S6 The annual percentage change (APC) of ASDR of NALC in 21 GBD regions and global, from 1990 to 2021**

**Table S7 Changes in NALC deaths in men and women at global and regional levels from 1990 to 2021, decomposed by three population-level determinants: population aging, population growth, and epidemiological changes.**

**Table S8 Changes in NALC deaths according to population-level determinants of population growth, aging, and epidemiological change from 1990 to 2021 in 203 countries**

**Table S9 Frontier deaths, and effective difference by country or territory in 2021**

**Table S10 Projected Absolute and Age-Standardized Death Rates (ASDR) for NALC in men and women Globally through 2045**

**Table S11 Projected Absolute Death Numbers for NALC in women Globally by Age Group through 2045**

**Table S12 Projected age-standardized death rate (ASDR) for NALC in women Globally by Age Group through 2045**

**Table S13 Projected Absolute Death Numbers for NALC in men Globally by Age Group through 2045**

**Table S14 Projected age-standardized death rate (ASDR) for NALC in men Globally by Age Group through 2045**

**Supplementary Methods**

**Calculation of ASR**

$$\text{ASR} = \frac{\left(\sum_{i=1}^{A} a_i w_i\right)}{\left(\sum_{i=1}^{A} w_i\right)} / \times 100{,}000$$

In this formula, $i$ represents the $i$-th age group. The term $a_i$ denotes the crude rate of the specific disease within the $i$-th age group, while $w_i$ refers to the weight assigned to the $i$-th age group based on a reference standard population. The weight $w_i$ reflects the proportion of individuals in that age group within the reference population, ensuring that the age distribution is standardized across different populations.

**Joinpoint analysis**

Time trend analysis is an important component of epidemiological research. Traditional regression models primarily fit and evaluate the overall trend of disease distribution within the study period from a global perspective, failing to capture local ariation characteristics. In 1998, Kim et al. first proposed the Joinpoint regression model. The core idea of this model is to establish segmented regression based on the temporal characteristics of disease distribution. By dividing the study time into different intervals through several Joinpoints, the trend in each interval is fitted and optimized, allowing for a more detailed assessment of the specific disease change characteristics within different intervals of the overall time range[1]. The Joinpoint regression model, developed by the Division of Cancer Control and Population Sciences at the National Cancer Institute of the United States, has been widely applied in the field of trend studies on disease incidence and mortality rates.

(I) Model Introduction

The Joinpoint regression model includes two types: the linear model (y = xb) and the logarithmic linear model (ln y = xb). If the dependent variable follows a normal distribution (or approximately normal distribution) and the sample size is large (usually greater than 100), the linear model is preferred. For example, when the dependent variable is continuous variables like height, weight, etc. If the dependent variable follows an exponential distribution or a Poisson distribution, the logarithmic linear model is more suitable. For instance, when the dependent variable represents epidemiological data based on populations such as incidence rates, number of cases, etc. When analyzing trends in the incidence, prevalence, mortality rates, and DALYs rates of thalassemia based on population data, the logarithmic linear model is generally chosen.

(II) Modeling Method

The grid search method (GSM) is the default modeling approach used by Joinpoint. GSM divides the study data into a grid, with each grid intersection corresponding to a planned scenario[1]. Then, within the specified intervals, it computes performance metrics for the corresponding equations at each point using a fixed step size to determine the optimal function. In essence, the Joinpoint model uses the GSM to establish all possible segment function Joinpoints (i.e., Joinpoints) and calculates the sum of squares errors (SSE) and mean squared errors (MSE) for each possible scenario. It selects the grid point with the smallest MSE as the Joinpoint for the segment function and fits the equation parameters such as $\beta_0$, $\beta_1$, $\delta_1$, ..., $\delta_k$ based on the selected Joinpoints and interval functions[2].

(III) Model Optimization

Monte Carlo permutation test is the default model optimization method in Joinpoint software. Before modeling, it is necessary to set the range of the number of Joinpoints k as $k \in$ (MIN，MAX) , where MIN represents the minimum number of Joinpoints, which is usually set to 0;

MAX represents the maximum number of Joinpoints. Each permutation test checks the null hypothesis $H_0$: the number of Joinpoints is $k = k_a$, and the alternative hypothesis $H_1$: the number of Joinpoints is $k = k_b$. The permutation test starts from $k_a$=MIN and $k_b$=MAX. If $H_0$ is rejected, k is set to $k_a + 1$ for further testing; if $H_0$ is not rejected, k is set to $k_b - 1$ for another test, until $k_a = k_b$, which means $k = k_a = k_b$ is the preferred number of Joinpoints selected by the permutation test, and the corresponding model is the optimal model IV) Index Calculation

Annual percent change (APC) and average annual percent change (AAPC) along with their 95% confidence intervals (CI) are the primary outcome indicators of the Joinpoint model. As the name suggests, APC represents the average annual percentage change of the dependent variable. For example, in a logarithmic linear model $\ln(y) = \beta_0 + \beta_1 x$, where y represents the incidence rate and x represents the year of incidence, the formula for calculating APC in the fitted model can be derived as:

$$\text{APC} = \left(\frac{y_{x+1} - y_x}{y_x}\right) \times 100 = \left(e^{\beta_1} - 1\right) \times 100$$

The lower and upper limits of the 100(1-α) % confidence interval are respectively:

$$APC_{L(\alpha)} = 100\left(e^{\beta_1 - s\times t_d^{-1}(1-\alpha/2)} - 1\right)$$

$$APC_{U(\alpha)} = 100\left(e^{\beta_1 + s\times t_d^{-1}(1-\alpha/2)} - 1\right)$$

In the above formula, $\beta_1$ represents the regression coefficient, s represents the standard error of $\beta_1$, d represents the degrees of freedom, and $t_d(q)$ is the value corresponding to the qth percentile of the t-distribution with d degrees of freedom (such as 95%).

The APC is used to evaluate the internal trend of each independent interval of a segmented function or the overall trend with no connecting points. When it comes to assessing the overall average change trend encompassing multiple intervals, the AAPC is required. The parameter

calculation method of AAPC involves weighted calculation of the regression coefficients of each interval based on the width w of the segment intervals. Its formula is as follows:

$$AAPC = \left( e^{\frac{\sum w_i \beta_i}{\sum w_i}} - 1 \right) \times 100$$

The lower and upper limits of the 100(1-α) % confidence interval are respectively:

$$AAPC_{L(\alpha)} = \left\{ \exp\left[ \ln\left( \frac{AAPC}{100} + 1 \right) - Z_{1-\alpha/2} \sqrt{\sum \widetilde{w_i^2}\, \widetilde{\sigma_i^2}} \right] - 1 \right\}$$

$$AAPC_{U(\alpha)} = \left\{ \exp\left[ \ln\left( \frac{AAPC}{100} + 1 \right) + Z_{1-\alpha/2} \sqrt{\sum \widetilde{w_i^2}\, \widetilde{\sigma_i^2}} \right] - 1 \right\}$$

In the above formula, $w_i$ represents the width of each segment function interval (i.e, the number of years included in the interval), $\beta_i$ denotes the regression coefficient corresponding to each interval, $\sigma^2$i is the variance of $\beta_i$, and $Z_\alpha$ represents the corresponding value of the α percentile in the normal distribution.

**Decomposition analysis**

We first used the decomposition methodology of Das Gupta to decompose NASH-associated liver cancer deaths by population age structure, population growth, and epidemiologic changes[3-5]. The number of deaths at each location was obtained from the following formula:

$$\text{DALY}_{ay,\,py,\,ey} = \sum_{i=1}^{17} (a_{i,\,y} * p_{y} * e_{i,\,y})$$

Where $DALY_{ay, py, ey}$ represented DALYs based on the factors of age structure, population, and DALYs rate for specific year y; $a_{i\ y}$ represents the proportion of population for the age category i of the 17 age categories in given year y; $p_y$ represents the total population in given year y; and $e_{i, y}$ represents DALYs rate given age category i in year y. The contribution of each factor to the change in DALYs from 1990 to 2021 was defined by the effect of one factor changing while the other factors were held constant. For example, the effect of age structure was calculated as:

$(DALY_{a2021, p1990, e1990} + DALY_{a2021, p2021, e2021})/3 + (DALY_{a2021, p1990, e2019} + DALY_{a2021, p2021, e1990})/6] - [(DALY_{a1990, p2021, e2021} + DALY_{a1990, p1990, e1990})/3 + (DALY_{a1990, p2021b, e1990} + DALY_{a1990, p1990, e2021})/6$

## Frontier analysis

In order to evaluate the relationship between the burden of NASH-associated liver cancer and socio-demographic development, we applied a frontier analysis as a quantitative methodology to identify the lowest potentially achievable age-standardized death rate based on development status, as measured by the SDI. The death rate frontier pinpoints the minimum number of deaths that could be attained for every country or territory given its SDI. Distance from the frontier is termed the effective difference; a large effective difference from the frontier suggests there may be unrealized opportunities for gains or improvement (reduction in NASH-associated liver cancer deaths) that should be possible based on the country or territory's place on the development spectrum. A data envelopment analysis, which allows for the delineation of non-linear frontiers, utilizing the free disposal hull method, was developed to produce a frontier for age-adjusted NASH-associated liver cancer deaths by SDI, using data from 1990-2021. In order to account for uncertainty, we used 1,000 bootstrapped samples of the data, randomly sampling with replacement from all countries and territories across all years. The mean death rate for NASH-associated liver cancer at each SDI value from the

bootstrapped samples was computed. A LOESS regression with local polynomial degree of 1 and span of 0.2 was then developed to generate a smoothed frontier. To exclude the influence of outliers, super-efficient countries were excluded from the generation of the frontier. To understand the relationship between age-standardized NASH-associated liver cancer death rates relative to the frontier in 2021, we calculated the effective difference (the absolute distance from the frontier) using 2021 SDI and age-standardized death rate data points for each country or territory. Countries or territories with lower death rates than the frontier were assigned a zero distance.

**References**

1. Kim HJ, Fay MP, Feuer EJ, Midthune DN: **Permutation tests for joinpoint regression with applications to cancer rates**. *Stat Med* 2000, **19**(3):335-351.

2. Kim S, Lee S, Choi JI, Cho H: **Binary genetic algorithm for optimal joinpoint detection: Application to cancer trend analysis**. *Stat Med* 2021, **40**(3):799-822.

3. Das Gupta P: **Standardization and decomposition of rates from cross-classified data**. *Genus* 1994, **50**(3-4):171-196.

4. Chevan A, Sutherland M: **Revisiting Das Gupta: refinement and extension of standardization and decomposition**. *Demography* 2009, **46**(3):429-449.

5. Gupta PD: **Standardization and decomposition of rates: a user's manual**; 1993.

**Table S1. List of International Classification of Diseases (ICD) codes mapped to the Global Burden of Disease cause list for liver cancer mortality data.**

| Disease | ICD10 | ICD9 |
|---|---|---|
| **Liver cancer** | C22 to C22.0 to C22.1 to C22.3 to C22.4 to C22.5 to C22.7 to C22.8 to D13.4 | 155 to 155.0 to 155.1 to 155.3 to 155.5 to 155.9 to 211.5 |

## Table S2 Terms and definitions to Country/Region Groupings Used in This Study to Including SDI Countries and GBD Regions to 21GBD region and 5 SDI region specific distribution

**The Global Burden of Disease (GBD):** GBD is a comprehensive epidemiological database spearheaded by the Institute for Health Metrics and Evaluation (IHME) at the University of Washing ton. It covers 288 causes of death to 71 diseases and injuries to and 88 risk fac to rs across 204 countries and terri to ries to including subnational estimates for 21 regions to spanning the years 1990 to 2021. The GBD provides detailed insights in to global health trends and emerging challenges. With its vast array of data sources and advanced statistical modeling methods to the GBD database offers the most comprehensive estimates of disease burden to including the burden of nonalcoholic stea to hepatitis (NASH)-associated liver cancer. This study utilized the most recent GBD dataset to evaluate the global burden of NASH-associated liver cancer to providing updated and in-depth assessments of this pressing public health issue.

**Age-standardized rate:** Rate per 100 to 000 population following standardization to the global age structure. The difference between age-standardized rates across geographies and over time is independent of population size and age structure.

**The Sociodemographic Index (SDI):** SDI is a composite measure that quantifies the level of sociodemographic development of a country or region. It is comparable across geographies and over time. The SDI is expressed on a scale from 0 to 1 and is calculated as the composite average of rankings for per capita income to average educational attainment to and to tal fertility rates across all areas included in the GBD study. A value of 0 represents the lowest income per capita to lowest educational attainment to and highest fertility rate observed across all GBD geographies from 1970 to 2021 to while a value of 1 represents the highest income per capita to highest educational attainment to and lowest fertility rate.

**Decomposition analysis:** An analysis approach invented by Das Gupta to which summarize the contribution of various fac to rs to the observed changes by algebraic isolation of the standardized impact of each contribution multiplication fac to r. Decomposing the epidemiological indica to rs of disease according to age structure to

population growth and epidemiologic changes can quantify the contribution of each of these fac to rs to the overall effect.

**Frontier analysis:** frontier analysis is an analytic approach used to identify the lowest potentially achievable burden of NASH-associated liver cancer based on a country or region's level of development to as measured by the SDI. The frontier represents those countries or terri to ries that are at the forefront of performance to achieving the lowest burden of NASH-associated liver cancer for their respective SDI. The distance from this frontier to termed the “effective difference to” reflects the gap between the observed burden and the theoretically achievable burden of disease for a country or region to given its SDI. This gap suggests an opportunity to reduce or eliminate the excess burden of disease based on the country or region’s sociodemographic resources. For instance, to if a country or terri to ry falls significantly below the frontier for its SDI to this indicates an unrealized potential for reducing NASH-associated liver cancer deaths to which could be addressed through better utilization of available resources aligned with its development status.

**Disability-adjusted life-years (DALYs)**: A measure that quantitates the overall burden of disease in terms of years of healthy life lost due to the disease. It represents the sum of years lost due to premature death and years living with disability due to the disease. The years of living with
disability is weighted in proportion to the severity of the underlying disease.

**Confidence interval (CI) and uncertainty intervals (UIs):** Because the mean value of the real population is unknown to the range with upper and lower limits is calculated according to the samples to describe the possible value of the mean value to which is the mean CI. We used the 25th and 975th ordinal 1000 draw values of the posterior distribution to generate UIs.

**Country/Region Groupings Used in This Study to Including SDI Countries and GBD Regions**

| Location ID | Location Name | 2021 SDI Index Value | SDI Quintile |
|---|---|---|---|
| 1 | Global | 0.666367819 | Middle SDI |
| 4 | Southeast Asia, East Asia, and Oceania | 0.696630914 | Middle SDI |
| 5 | East Asia | 0.722912119 | High-middle SDI |
| 6 | China | 0.71867919 | High-middle SDI |
| 7 | Democratic People's Republic of Korea | 0.56945513 | Low-middle SDI |
| 8 | Taiwan (Province of China) | 0.875139514 | High SDI |
| 9 | Southeast Asia | 0.64907177 | Middle SDI |
| 10 | Cambodia | 0.473999694 | Low-middle SDI |
| 11 | Indonesia | 0.657934796 | Middle SDI |
| 12 | Lao People's Democratic Republic | 0.489280726 | Low-middle SDI |
| 13 | Malaysia | 0.742552841 | High-middle SDI |
| 14 | Maldives | 0.657665453 | Middle SDI |
| 15 | Myanmar | 0.528492169 | Low-middle SDI |
| 16 | Philippines | 0.651920253 | Middle SDI |

| Location ID | Location Name | 2021 SDI Index Value | SDI Quintile |
|---|---|---|---|
| 17 | Sri Lanka | 0.701371778 | Middle SDI |
| 18 | Thailand | 0.682657272 | Middle SDI |
| 19 | Timor-Leste | 0.450689053 | Low SDI |
| 20 | Viet Nam | 0.621620778 | Middle SDI |
| 21 | Oceania | 0.467359461 | Low-middle SDI |
| 22 | Fiji | 0.669068631 | Middle SDI |
| 23 | Kiribati | 0.525957502 | Low-middle SDI |
| 24 | Marshall Islands | 0.573524783 | Low-middle SDI |
| 25 | Micronesia (Federated States of) | 0.588012508 | Low-middle SDI |
| 26 | Papua New Guinea | 0.418098053 | Low SDI |
| 27 | Samoa | 0.592340278 | Low-middle SDI |
| 28 | Solomon Islands | 0.429541799 | Low SDI |
| 29 | Tonga | 0.629100964 | Middle SDI |
| 30 | Vanuatu | 0.472796337 | Low-middle SDI |
| 31 | Central Europe, Eastern Europe, and Central Asia | 0.768649142 | High-middle SDI |
| 32 | Central Asia | 0.674963478 | Middle SDI |

| Location ID | Location Name | 2021 SDI Index Value | SDI Quintile |
|---|---|---|---|
| 33 | Armenia | 0.702496602 | Middle SDI |
| 34 | Azerbaijan | 0.695410598 | Middle SDI |
| 35 | Georgia | 0.733123642 | High-middle SDI |
| 36 | Kazakhstan | 0.718331647 | High-middle SDI |
| 37 | Kyrgyzstan | 0.609180728 | Low-middle SDI |
| 38 | Mongolia | 0.618744133 | Low-middle SDI |
| 39 | Tajikistan | 0.536613238 | Low-middle SDI |
| 40 | Turkmenistan | 0.683039569 | Middle SDI |
| 41 | Uzbekistan | 0.664964654 | Middle SDI |
| 42 | Central Europe | 0.795780357 | High-middle SDI |
| 43 | Albania | 0.706888685 | Middle SDI |
| 44 | Bosnia and Herzegovina | 0.72296408 | High-middle SDI |
| 45 | Bulgaria | 0.764641037 | High-middle SDI |
| 46 | Croatia | 0.799069214 | High-middle SDI |
| 47 | Czechia | 0.828510085 | High SDI |
| 48 | Hungary | 0.791024669 | High-middle SDI |

| Location ID | Location Name | 2021 SDI Index Value | SDI Quintile |
|---|---|---|---|
| 49 | North Macedonia | 0.750954677 | High-middle SDI |
| 50 | Montenegro | 0.796532951 | High-middle SDI |
| 51 | Poland | 0.812073312 | High SDI |
| 52 | Romania | 0.766321392 | High-middle SDI |
| 53 | Serbia | 0.79221264 | High-middle SDI |
| 54 | Slovakia | 0.808329132 | High-middle SDI |
| 55 | Slovenia | 0.842633141 | High SDI |
| 56 | Eastern Europe | 0.803414319 | High-middle SDI |
| 57 | Belarus | 0.784114127 | High-middle SDI |
| 58 | Estonia | 0.845787294 | High SDI |
| 59 | Latvia | 0.830715451 | High SDI |
| 60 | Lithuania | 0.857613278 | High SDI |
| 61 | Republic of Moldova | 0.732393345 | High-middle SDI |
| 62 | Russian Federation | 0.809111108 | High-middle SDI |
| 63 | Ukraine | 0.761045561 | High-middle SDI |
| 64 | High-income | 0.85286503 | High SDI |

| Location ID | Location Name | 2021 SDI Index Value | SDI Quintile |
|---|---|---|---|
| 65 | High-income Asia Pacific | 0.877157409 | High SDI |
| 66 | Brunei Darussalam | 0.810288851 | High SDI |
| 67 | Japan | 0.871459701 | High SDI |
| 68 | Republic of Korea | 0.887195638 | High SDI |
| 69 | Singapore | 0.856235308 | High SDI |
| 70 | Australasia | 0.845644432 | High SDI |
| 71 | Australia | 0.844269408 | High SDI |
| 72 | New Zealand | 0.850145187 | High SDI |
| 73 | Western Europe | 0.848728514 | High SDI |
| 74 | Andorra | 0.869895393 | High SDI |
| 75 | Austria | 0.854558286 | High SDI |
| 76 | Belgium | 0.853674059 | High SDI |
| 77 | Cyprus | 0.835648571 | High SDI |
| 78 | Denmark | 0.897314038 | High SDI |
| 79 | Finland | 0.860244219 | High SDI |
| 80 | France | 0.837816091 | High SDI |

| Location ID | Location Name | 2021 SDI Index Value | SDI Quintile |
|---|---|---|---|
| 81 | Germany | 0.903515704 | High SDI |
| 82 | Greece | 0.791882294 | High-middle SDI |
| 83 | Iceland | 0.874628639 | High SDI |
| 84 | Ireland | 0.873989853 | High SDI |
| 85 | Israel | 0.809091066 | High-middle SDI |
| 86 | Italy | 0.805537426 | High-middle SDI |
| 87 | Luxembourg | 0.884636327 | High SDI |
| 88 | Malta | 0.801853922 | High-middle SDI |
| 89 | Netherlands | 0.888375951 | High SDI |
| 90 | Norway | 0.916631633 | High SDI |
| 91 | Portugal | 0.745394909 | High-middle SDI |
| 92 | Spain | 0.76948336 | High-middle SDI |
| 93 | Sweden | 0.887384361 | High SDI |
| 94 | Switzerland | 0.933531726 | High SDI |
| 95 | United Kingdom | 0.858444983 | High SDI |
| 96 | Southern Latin America | 0.743029817 | High-middle SDI |

| Location ID | Location Name | 2021 SDI Index Value | SDI Quintile |
|---|---|---|---|
| 97 | Argentina | 0.733528396 | High-middle SDI |
| 98 | Chile | 0.770149297 | High-middle SDI |
| 99 | Uruguay | 0.721713499 | High-middle SDI |
| 100 | High-income North America | 0.86421664 | High SDI |
| 101 | Canada | 0.873181934 | High SDI |
| 102 | United States of America | 0.863243823 | High SDI |
| 103 | Latin America and Caribbean | 0.646195591 | Middle SDI |
| 104 | Caribbean | 0.6423146 | Middle SDI |
| 105 | Antigua and Barbuda | 0.749849952 | High-middle SDI |
| 106 | Bahamas | 0.805143711 | High-middle SDI |
| 107 | Barbados | 0.74706542 | High-middle SDI |
| 108 | Belize | 0.61055234 | Low-middle SDI |
| 109 | Cuba | 0.669331767 | Middle SDI |
| 110 | Dominica | 0.747381853 | High-middle SDI |
| 111 | Dominican Republic | 0.619170694 | Middle SDI |
| 112 | Grenada | 0.6693506 | Middle SDI |

| Location ID | Location Name | 2021 SDI Index Value | SDI Quintile |
|---|---|---|---|
| 113 | Guyana | 0.650902479 | Middle SDI |
| 114 | Haiti | 0.448751017 | Low SDI |
| 115 | Jamaica | 0.68306364 | Middle SDI |
| 116 | Saint Lucia | 0.672601687 | Middle SDI |
| 117 | Saint Vincent and the Grenadines | 0.640886762 | Middle SDI |
| 118 | Suriname | 0.641162711 | Middle SDI |
| 119 | Trinidad and Tobago | 0.769401094 | High-middle SDI |
| 120 | Andean Latin America | 0.654007956 | Middle SDI |
| 121 | Bolivia (Plurinational State of) | 0.604496662 | Low-middle SDI |
| 122 | Ecuador | 0.665675436 | Middle SDI |
| 123 | Peru | 0.662036006 | Middle SDI |
| 124 | Central Latin America | 0.641931122 | Middle SDI |
| 125 | Colombia | 0.65664043 | Middle SDI |
| 126 | Costa Rica | 0.704369665 | Middle SDI |
| 127 | El Salvador | 0.565569678 | Low-middle SDI |
| 128 | Guatemala | 0.540099007 | Low-middle SDI |

| Location ID | Location Name | 2021 SDI Index Value | SDI Quintile |
|---|---|---|---|
| 129 | Honduras | 0.513585699 | Low-middle SDI |
| 130 | Mexico | 0.66496867 | Middle SDI |
| 131 | Nicaragua | 0.52364671 | Low-middle SDI |
| 132 | Panama | 0.706659844 | Middle SDI |
| 133 | Venezuela (Bolivarian Republic of) | 0.596599587 | Low-middle SDI |
| 134 | Tropical Latin America | 0.648941531 | Middle SDI |
| 135 | Brazil | 0.648846512 | Middle SDI |
| 136 | Paraguay | 0.650487525 | Middle SDI |
| 137 | North Africa and Middle East | 0.658716072 | Middle SDI |
| 138 | North Africa and Middle East | 0.658716072 | Middle SDI |
| 139 | Algeria | 0.659720087 | Middle SDI |
| 140 | Bahrain | 0.752218099 | High-middle SDI |
| 141 | Egypt | 0.603962121 | Low-middle SDI |
| 142 | Iran (Islamic Republic of) | 0.69729326 | Middle SDI |
| 143 | Iraq | 0.662777495 | Middle SDI |
| 144 | Jordan | 0.725420238 | High-middle SDI |

| Location ID | Location Name | 2021 SDI Index Value | SDI Quintile |
|---|---|---|---|
| 145 | Kuwait | 0.846802486 | High SDI |
| 146 | Lebanon | 0.741226017 | High-middle SDI |
| 147 | Libya | 0.73508433 | High-middle SDI |
| 148 | Morocco | 0.561680434 | Low-middle SDI |
| 149 | Palestine | 0.629201641 | Middle SDI |
| 150 | Oman | 0.773801229 | High-middle SDI |
| 151 | Qatar | 0.846704498 | High SDI |
| 152 | Saudi Arabia | 0.814515567 | High SDI |
| 153 | Syrian Arab Republic | 0.622855859 | Middle SDI |
| 154 | Tunisia | 0.681701488 | Middle SDI |
| 155 | Turkey | 0.713246106 | High-middle SDI |
| 156 | United Arab Emirates | 0.849740335 | High SDI |
| 157 | Yemen | 0.453539967 | Low SDI |
| 158 | South Asia | 0.559642669 | Low-middle SDI |
| 159 | South Asia | 0.559642669 | Low-middle SDI |
| 160 | Afghanistan | 0.335068107 | Low SDI |

| Location ID | Location Name | 2021 SDI Index Value | SDI Quintile |
|---|---|---|---|
| 161 | Bangladesh | 0.493106236 | Low-middle SDI |
| 162 | Bhutan | 0.476724988 | Low-middle SDI |
| 163 | India | 0.5777383 | Low-middle SDI |
| 164 | Nepal | 0.433952916 | Low SDI |
| 165 | Pakistan | 0.504275856 | Low-middle SDI |
| 166 | Sub-Saharan Africa | 0.461224007 | Low SDI |
| 167 | Central Sub-Saharan Africa | 0.484517732 | Low-middle SDI |
| 168 | Angola | 0.482946052 | Low-middle SDI |
| 169 | Central African Republic | 0.311026626 | Low SDI |
| 170 | Congo | 0.586908906 | Low-middle SDI |
| 171 | Democratic Republic of the Congo | 0.390178166 | Low SDI |
| 172 | Equatorial Guinea | 0.663978286 | Middle SDI |
| 173 | Gabon | 0.639080604 | Middle SDI |
| 174 | Eastern Sub-Saharan Africa | 0.412187942 | Low SDI |
| 175 | Burundi | 0.291288817 | Low SDI |
| 176 | Comoros | 0.476955685 | Low-middle SDI |

| Location ID | Location Name | 2021 SDI Index Value | SDI Quintile |
|---|---|---|---|
| 177 | Djibouti | 0.489200321 | Low-middle SDI |
| 178 | Eritrea | 0.404572056 | Low SDI |
| 179 | Ethiopia | 0.360727644 | Low SDI |
| 180 | Kenya | 0.524783146 | Low-middle SDI |
| 181 | Madagascar | 0.401385119 | Low SDI |
| 182 | Malawi | 0.381985594 | Low SDI |
| 183 | Mauritius | 0.717977109 | High-middle SDI |
| 184 | Mozambique | 0.327475463 | Low SDI |
| 185 | Rwanda | 0.436140248 | Low SDI |
| 186 | Seychelles | 0.727579445 | High-middle SDI |
| 187 | Somalia | 0.077433678 | Low SDI |
| 189 | United Republic of Tanzania | 0.448565569 | Low SDI |
| 190 | Uganda | 0.426553554 | Low SDI |
| 191 | Zambia | 0.510230369 | Low-middle SDI |
| 192 | Southern Sub-Saharan Africa | 0.643347819 | Middle SDI |
| 193 | Botswana | 0.643077969 | Middle SDI |

| Location ID | Location Name | 2021 SDI Index Value | SDI Quintile |
|---|---|---|---|
| 194 | Lesotho | 0.51157061 | Low-middle SDI |
| 195 | Namibia | 0.618073651 | Low-middle SDI |
| 196 | South Africa | 0.681292244 | Middle SDI |
| 197 | Eswatini | 0.586216849 | Low-middle SDI |
| 198 | Zimbabwe | 0.475577138 | Low-middle SDI |
| 199 | Western Sub-Saharan Africa | 0.446420999 | Low SDI |
| 200 | Benin | 0.37452237 | Low SDI |
| 201 | Burkina Faso | 0.284470947 | Low SDI |
| 202 | Cameroon | 0.480364523 | Low-middle SDI |
| 203 | Cabo Verde | 0.533600978 | Low-middle SDI |
| 204 | Chad | 0.243516859 | Low SDI |
| 205 | Côte d'Ivoire | 0.424540566 | Low SDI |
| 206 | Gambia | 0.410077462 | Low SDI |
| 207 | Ghana | 0.563348184 | Low-middle SDI |
| 208 | Guinea | 0.336555329 | Low SDI |
| 209 | Guinea-Bissau | 0.353448423 | Low SDI |

| Location ID | Location Name | 2021 SDI Index Value | SDI Quintile |
|---|---|---|---|
| 210 | Liberia | 0.353229409 | Low SDI |
| 211 | Mali | 0.271175692 | Low SDI |
| 212 | Mauritania | 0.495266784 | Low-middle SDI |
| 213 | Niger | 0.170310328 | Low SDI |
| 214 | Nigeria | 0.503698612 | Low-middle SDI |
| 215 | Sao Tome and Principe | 0.503305577 | Low-middle SDI |
| 216 | Senegal | 0.409005254 | Low SDI |
| 217 | Sierra Leone | 0.35900867 | Low SDI |
| 218 | Togo | 0.410016394 | Low SDI |
| 298 | American Samoa | 0.726267628 | High-middle SDI |
| 305 | Bermuda | 0.821319794 | High SDI |
| 320 | Cook Islands | 0.778251758 | High-middle SDI |
| 349 | Greenland | 0.835640003 | High SDI |
| 351 | Guam | 0.80216771 | High-middle SDI |
| 354 | Hong Kong Special Administrative Region of China | 0.850450848 | High SDI |
| 361 | Macao Special Administrative Region of China | 0.874759481 | High SDI |

| Location ID | Location Name | 2021 SDI Index Value | SDI Quintile |
|---|---|---|---|
| 367 | Monaco | 0.909519124 | High SDI |
| 369 | Nauru | 0.627549782 | Middle SDI |
| 374 | Niue | 0.72621855 | High-middle SDI |
| 376 | Northern Mariana Islands | 0.777504838 | High-middle SDI |
| 380 | Palau | 0.754590186 | High-middle SDI |
| 385 | Puerto Rico | 0.824543903 | High SDI |
| 393 | Saint Kitts and Nevis | 0.756332641 | High-middle SDI |
| 396 | San Marino | 0.887883596 | High SDI |
| 413 | Tokelau | 0.68701842 | Middle SDI |
| 416 | Tuvalu | 0.578627145 | Low-middle SDI |
| 422 | United States Virgin Islands | 0.822988043 | High SDI |
| 433 | Northern Ireland | 0.841529955 | High SDI |
| 434 | Scotland | 0.853887319 | High SDI |
| 435 | South Sudan | 0.278377554 | Low SDI |
| 482 | Eastern Cape | 0.619101287 | Middle SDI |
| 483 | Free State | 0.678893967 | Middle SDI |

| Location ID | Location Name | 2021 SDI Index Value | SDI Quintile |
|---|---|---|---|
| 484 | Gauteng | 0.736905342 | High-middle SDI |
| 485 | KwaZulu-Natal | 0.662386215 | Middle SDI |
| 486 | Limpopo | 0.613431617 | Low-middle SDI |
| 487 | Mpumalanga | 0.648324523 | Middle SDI |
| 488 | North-West | 0.654616033 | Middle SDI |
| 489 | Northern Cape | 0.665813245 | Middle SDI |
| 490 | Western Cape | 0.719732052 | High-middle SDI |
| 491 | Anhui | 0.661241263 | Middle SDI |
| 492 | Beijing | 0.845876468 | High SDI |
| 493 | Chongqing | 0.713248108 | High-middle SDI |
| 494 | Fujian | 0.715426566 | High-middle SDI |
| 495 | Gansu | 0.629648423 | Middle SDI |
| 496 | Guangdong | 0.766590014 | High-middle SDI |
| 497 | Guangxi | 0.677747142 | Middle SDI |
| 498 | Guizhou | 0.622926981 | Middle SDI |
| 499 | Hainan | 0.70526301 | Middle SDI |

| Location ID | Location Name | 2021 SDI Index Value | SDI Quintile |
|---|---|---|---|
| 500 | Hebei | 0.703535825 | Middle SDI |
| 501 | Heilongjiang | 0.712062797 | High-middle SDI |
| 502 | Henan | 0.699479878 | Middle SDI |
| 503 | Hubei | 0.711832348 | Middle SDI |
| 504 | Hunan | 0.692120518 | Middle SDI |
| 505 | Inner Mongolia | 0.731684517 | High-middle SDI |
| 506 | Jiangsu | 0.755114988 | High-middle SDI |
| 507 | Jiangxi | 0.678215672 | Middle SDI |
| 508 | Jilin | 0.735628033 | High-middle SDI |
| 509 | Liaoning | 0.744555054 | High-middle SDI |
| 510 | Ningxia | 0.695319453 | Middle SDI |
| 511 | Qinghai | 0.648309483 | Middle SDI |
| 512 | Shaanxi | 0.724147628 | High-middle SDI |
| 513 | Shandong | 0.725623497 | High-middle SDI |
| 514 | Shanghai | 0.828661817 | High SDI |
| 515 | Shanxi | 0.711816196 | Middle SDI |

| Location ID | Location Name | 2021 SDI Index Value | SDI Quintile |
|---|---|---|---|
| 516 | Sichuan | 0.672696014 | Middle SDI |
| 517 | Tianjin | 0.833804693 | High SDI |
| 518 | Tibet | 0.483893412 | Low-middle SDI |
| 519 | Xinjiang | 0.70686651 | Middle SDI |
| 520 | Yunnan | 0.627205795 | Middle SDI |
| 521 | Zhejiang | 0.742041363 | High-middle SDI |
| 522 | Sudan | 0.542748299 | Low-middle SDI |
| 523 | Alabama | 0.826417909 | High SDI |
| 524 | Alaska | 0.857125567 | High SDI |
| 525 | Arizona | 0.847683473 | High SDI |
| 526 | Arkansas | 0.816754461 | High SDI |
| 527 | California | 0.871090459 | High SDI |
| 528 | Colorado | 0.875986347 | High SDI |
| 529 | Connecticut | 0.901972117 | High SDI |
| 530 | Delaware | 0.866079256 | High SDI |
| 531 | District of Columbia | 0.907426863 | High SDI |

| Location ID | Location Name | 2021 SDI Index Value | SDI Quintile |
|---|---|---|---|
| 532 | Florida | 0.861825164 | High SDI |
| 533 | Georgia | 0.847268118 | High SDI |
| 534 | Hawaii | 0.87084045 | High SDI |
| 535 | Idaho | 0.836495322 | High SDI |
| 536 | Illinois | 0.880611434 | High SDI |
| 537 | Indiana | 0.844050669 | High SDI |
| 538 | Iowa | 0.864342086 | High SDI |
| 539 | Kansas | 0.858890931 | High SDI |
| 540 | Kentucky | 0.821720983 | High SDI |
| 541 | Louisiana | 0.826669718 | High SDI |
| 542 | Maine | 0.866792716 | High SDI |
| 543 | Maryland | 0.891055635 | High SDI |
| 544 | Massachusetts | 0.90725037 | High SDI |
| 545 | Michigan | 0.864940748 | High SDI |
| 546 | Minnesota | 0.887884435 | High SDI |
| 547 | Mississippi | 0.811867151 | High SDI |

| Location ID | Location Name | 2021 SDI Index Value | SDI Quintile |
|---|---|---|---|
| 548 | Missouri | 0.849044295 | High SDI |
| 549 | Montana | 0.859517184 | High SDI |
| 550 | Nebraska | 0.865629234 | High SDI |
| 551 | Nevada | 0.847864111 | High SDI |
| 552 | New Hampshire | 0.898526447 | High SDI |
| 553 | New Jersey | 0.891850577 | High SDI |
| 554 | New Mexico | 0.832846305 | High SDI |
| 555 | New York | 0.88592619 | High SDI |
| 556 | North Carolina | 0.846173734 | High SDI |
| 557 | North Dakota | 0.876134627 | High SDI |
| 558 | Ohio | 0.851227042 | High SDI |
| 559 | Oklahoma | 0.82814491 | High SDI |
| 560 | Oregon | 0.870189511 | High SDI |
| 561 | Pennsylvania | 0.873950359 | High SDI |
| 562 | Rhode Island | 0.884283653 | High SDI |
| 563 | South Carolina | 0.838586487 | High SDI |

| Location ID | Location Name | 2021 SDI Index Value | SDI Quintile |
|---|---|---|---|
| 564 | South Dakota | 0.856263782 | High SDI |
| 565 | Tennessee | 0.831968835 | High SDI |
| 566 | Texas | 0.836777383 | High SDI |
| 567 | Utah | 0.854829295 | High SDI |
| 568 | Vermont | 0.89152237 | High SDI |
| 569 | Virginia | 0.881907315 | High SDI |
| 570 | Washington | 0.878013634 | High SDI |
| 571 | West Virginia | 0.82033351 | High SDI |
| 572 | Wisconsin | 0.873095963 | High SDI |
| 573 | Wyoming | 0.863142903 | High SDI |
| 4618 | North East England | 0.825842759 | High SDI |
| 4619 | North West England | 0.843295382 | High SDI |
| 4620 | Yorkshire and the Humber | 0.836407087 | High SDI |
| 4621 | East Midlands | 0.835023214 | High SDI |
| 4622 | West Midlands | 0.834648192 | High SDI |
| 4623 | East of England | 0.85577408 | High SDI |

| Location ID | Location Name | 2021 SDI Index Value | SDI Quintile |
|---|---|---|---|
| 4624 | Greater London | 0.9041535 | High SDI |
| 4625 | South East England | 0.875365568 | High SDI |
| 4626 | South West England | 0.860849638 | High SDI |
| 4636 | Wales | 0.833274667 | High SDI |
| 4643 | Aguascalientes | 0.682557435 | Middle SDI |
| 4644 | Baja California | 0.704776585 | Middle SDI |
| 4645 | Baja California Sur | 0.710175355 | Middle SDI |
| 4646 | Campeche | 0.665087938 | Middle SDI |
| 4647 | Coahuila | 0.678075116 | Middle SDI |
| 4648 | Colima | 0.699338436 | Middle SDI |
| 4649 | Chiapas | 0.569756592 | Low-middle SDI |
| 4650 | Chihuahua | 0.674472052 | Middle SDI |
| 4651 | Mexico City | 0.759378377 | High-middle SDI |
| 4652 | Durango | 0.640562517 | Middle SDI |
| 4653 | Guanajuato | 0.647044734 | Middle SDI |
| 4654 | Guerrero | 0.584126986 | Low-middle SDI |

| Location ID | Location Name | 2021 SDI Index Value | SDI Quintile |
|---|---|---|---|
| 4655 | Hidalgo | 0.633128071 | Middle SDI |
| 4656 | Jalisco | 0.677078025 | Middle SDI |
| 4657 | México | 0.681505383 | Middle SDI |
| 4658 | Michoacán de Ocampo | 0.613949206 | Low-middle SDI |
| 4659 | Morelos | 0.670104932 | Middle SDI |
| 4660 | Nayarit | 0.657928691 | Middle SDI |
| 4661 | Nuevo León | 0.712152517 | High-middle SDI |
| 4662 | Oaxaca | 0.588389144 | Low-middle SDI |
| 4663 | Puebla | 0.622884968 | Middle SDI |
| 4664 | Querétaro | 0.684048164 | Middle SDI |
| 4665 | Quintana Roo | 0.682591131 | Middle SDI |
| 4666 | San Luis Potosí | 0.647579585 | Middle SDI |
| 4667 | Sinaloa | 0.678348037 | Middle SDI |
| 4668 | Sonora | 0.709903997 | Middle SDI |
| 4669 | Tabasco | 0.649850519 | Middle SDI |
| 4670 | Tamaulipas | 0.682912198 | Middle SDI |

| Location ID | Location Name | 2021 SDI Index Value | SDI Quintile |
|---|---|---|---|
| 4671 | Tlaxcala | 0.64898896 | Middle SDI |
| 4672 | Veracruz de Ignacio de la Llave | 0.627366789 | Middle SDI |
| 4673 | Yucatán | 0.654459012 | Middle SDI |
| 4674 | Zacatecas | 0.636670528 | Middle SDI |
| 4709 | Aceh | 0.671759479 | Middle SDI |
| 4710 | North Sumatra | 0.669499052 | Middle SDI |
| 4711 | West Sumatra | 0.66784543 | Middle SDI |
| 4712 | Riau | 0.724215125 | High-middle SDI |
| 4713 | Jambi | 0.640779478 | Middle SDI |
| 4714 | South Sumatra | 0.646421244 | Middle SDI |
| 4715 | Bengkulu | 0.613885329 | Low-middle SDI |
| 4716 | Lampung | 0.60891275 | Low-middle SDI |
| 4717 | Bangka-Belitung Islands | 0.644388463 | Middle SDI |
| 4718 | Riau Islands | 0.749803315 | High-middle SDI |
| 4719 | North Kalimantan | 0.754016402 | High-middle SDI |
| 4720 | Jakarta | 0.801237549 | High-middle SDI |

| Location ID | Location Name | 2021 SDI Index Value | SDI Quintile |
|---|---|---|---|
| 4721 | West Java | 0.644279321 | Middle SDI |
| 4722 | Central Java | 0.613780247 | Low-middle SDI |
| 4723 | Yogyakarta | 0.676829859 | Middle SDI |
| 4724 | East Java | 0.646540022 | Middle SDI |
| 4725 | Banten | 0.641544087 | Middle SDI |
| 4726 | Bali | 0.652382779 | Middle SDI |
| 4727 | West Nusa Tenggara | 0.587663369 | Low-middle SDI |
| 4728 | East Nusa Tenggara | 0.550545868 | Low-middle SDI |
| 4729 | West Kalimantan | 0.587438981 | Low-middle SDI |
| 4730 | Central Kalimantan | 0.639931265 | Middle SDI |
| 4731 | South Kalimantan | 0.622221912 | Middle SDI |
| 4732 | East Kalimantan | 0.761652368 | High-middle SDI |
| 4733 | North Sulawesi | 0.652614588 | Middle SDI |
| 4734 | Central Sulawesi | 0.617544908 | Low-middle SDI |
| 4735 | South Sulawesi | 0.622994177 | Middle SDI |
| 4736 | Southeast Sulawesi | 0.618324728 | Low-middle SDI |

| Location ID | Location Name | 2021 SDI Index Value | SDI Quintile |
|---|---|---|---|
| 4737 | Gorontalo | 0.571050074 | Low-middle SDI |
| 4738 | West Sulawesi | 0.576458969 | Low-middle SDI |
| 4739 | Maluku | 0.581624279 | Low-middle SDI |
| 4740 | North Maluku | 0.563444978 | Low-middle SDI |
| 4741 | West Papua | 0.676555222 | Middle SDI |
| 4742 | Papua | 0.646553603 | Middle SDI |
| 4749 | England | 0.860552694 | High SDI |
| 4750 | Acre | 0.562074727 | Low-middle SDI |
| 4751 | Alagoas | 0.529742892 | Low-middle SDI |
| 4752 | Amazonas | 0.603585976 | Low-middle SDI |
| 4753 | Amapá | 0.629807813 | Middle SDI |
| 4754 | Bahia | 0.574142222 | Low-middle SDI |
| 4755 | Ceará | 0.563912693 | Low-middle SDI |
| 4756 | Distrito Federal | 0.776152007 | High-middle SDI |
| 4757 | Espírito Santo | 0.667428625 | Middle SDI |
| 4758 | Goiás | 0.639347711 | Middle SDI |

| Location ID | Location Name | 2021 SDI Index Value | SDI Quintile |
|---|---|---|---|
| 4759 | Maranhão | 0.49216193 | Low-middle SDI |
| 4760 | Minas Gerais | 0.648904701 | Middle SDI |
| 4761 | Mato Grosso do Sul | 0.642693307 | Middle SDI |
| 4762 | Mato Grosso | 0.647043759 | Middle SDI |
| 4763 | Pará | 0.577314362 | Low-middle SDI |
| 4764 | Paraíba | 0.557922296 | Low-middle SDI |
| 4765 | Paraná | 0.669860641 | Middle SDI |
| 4766 | Pernambuco | 0.583214758 | Low-middle SDI |
| 4767 | Piaui | 0.520291625 | Low-middle SDI |
| 4768 | Rio de Janeiro | 0.710470527 | Middle SDI |
| 4769 | Rio Grande do Norte | 0.585328241 | Low-middle SDI |
| 4770 | Rondônia | 0.618510473 | Low-middle SDI |
| 4771 | Roraima | 0.609883228 | Low-middle SDI |
| 4772 | Rio Grande do Sul | 0.689722837 | Middle SDI |
| 4773 | Santa Catarina | 0.694839624 | Middle SDI |
| 4774 | Sergipe | 0.590589987 | Low-middle SDI |

| Location ID | Location Name | 2021 SDI Index Value | SDI Quintile |
|---|---|---|---|
| 4775 | São Paulo | 0.711182598 | Middle SDI |
| 4776 | Tocantins | 0.601403 | Low-middle SDI |
| 4841 | Andhra Pradesh | 0.552472881 | Low-middle SDI |
| 4842 | Arunachal Pradesh | 0.585180701 | Low-middle SDI |
| 4843 | Assam | 0.573442541 | Low-middle SDI |
| 4844 | Bihar | 0.45805086 | Low SDI |
| 4846 | Chhattisgarh | 0.540359862 | Low-middle SDI |
| 4849 | Delhi | 0.728650031 | High-middle SDI |
| 4850 | Goa | 0.724320503 | High-middle SDI |
| 4851 | Gujarat | 0.622975241 | Middle SDI |
| 4852 | Haryana | 0.629602575 | Middle SDI |
| 4853 | Himachal Pradesh | 0.643033305 | Middle SDI |
| 4854 | Jammu & Kashmir and Ladakh | 0.609583681 | Low-middle SDI |
| 4855 | Jharkhand | 0.5242297 | Low-middle SDI |
| 4856 | Karnataka | 0.585910724 | Low-middle SDI |
| 4857 | Kerala | 0.666417241 | Middle SDI |

| Location ID | Location Name | 2021 SDI Index Value | SDI Quintile |
|---|---|---|---|
| 4859 | Madhya Pradesh | 0.531115567 | Low-middle SDI |
| 4860 | Maharashtra | 0.639494993 | Middle SDI |
| 4861 | Manipur | 0.584558179 | Low-middle SDI |
| 4862 | Meghalaya | 0.560405068 | Low-middle SDI |
| 4863 | Mizoram | 0.62843683 | Middle SDI |
| 4864 | Nagaland | 0.637648412 | Middle SDI |
| 4865 | Odisha | 0.551364782 | Low-middle SDI |
| 4867 | Punjab | 0.632901572 | Middle SDI |
| 4868 | Rajasthan | 0.536745684 | Low-middle SDI |
| 4869 | Sikkim | 0.635190296 | Middle SDI |
| 4870 | Tamil Nadu | 0.629914474 | Middle SDI |
| 4871 | Telangana | 0.567480596 | Low-middle SDI |
| 4872 | Tripura | 0.555782348 | Low-middle SDI |
| 4873 | Uttar Pradesh | 0.527579329 | Low-middle SDI |
| 4874 | Uttarakhand | 0.661804198 | Middle SDI |
| 4875 | West Bengal | 0.556791581 | Low-middle SDI |

| Location ID | Location Name | 2021 SDI Index Value | SDI Quintile |
|---|---|---|---|
| 4910 | Oslo | 0.947286104 | High SDI |
| 4920 | Rogaland | 0.916764681 | High SDI |
| 4923 | Møre og Romsdal | 0.908149197 | High SDI |
| 4926 | Nordland | 0.898213885 | High SDI |
| 4940 | Sweden except Stockholm | 0.875759008 | High SDI |
| 4944 | Stockholm | 0.916765403 | High SDI |
| 25318 | Abia | 0.629261465 | Middle SDI |
| 25319 | Adamawa | 0.474621913 | Low-middle SDI |
| 25320 | Akwa Ibom | 0.589407255 | Low-middle SDI |
| 25321 | Anambra | 0.62862687 | Middle SDI |
| 25322 | Bauchi | 0.294070246 | Low SDI |
| 25323 | Bayelsa | 0.558802657 | Low-middle SDI |
| 25324 | Benue | 0.49859261 | Low-middle SDI |
| 25325 | Borno | 0.398589587 | Low SDI |
| 25326 | Cross River | 0.562249026 | Low-middle SDI |
| 25327 | Delta | 0.614789164 | Low-middle SDI |

| Location ID | Location Name | 2021 SDI Index Value | SDI Quintile |
|---|---|---|---|
| 25328 | Ebonyi | 0.540874531 | Low-middle SDI |
| 25329 | Edo | 0.6270757 | Middle SDI |
| 25330 | Ekiti | 0.609689218 | Low-middle SDI |
| 25331 | Enugu | 0.60872942 | Low-middle SDI |
| 25332 | FCT (Abuja) | 0.621406693 | Middle SDI |
| 25333 | Gombe | 0.379904259 | Low SDI |
| 25334 | Imo | 0.629384576 | Middle SDI |
| 25335 | Jigawa | 0.287216991 | Low SDI |
| 25336 | Kaduna | 0.442437011 | Low SDI |
| 25337 | Kano | 0.408256207 | Low SDI |
| 25338 | Katsina | 0.297115378 | Low SDI |
| 25339 | Kebbi | 0.284501095 | Low SDI |
| 25340 | Kogi | 0.560608509 | Low-middle SDI |
| 25341 | Kwara | 0.536539828 | Low-middle SDI |
| 25342 | Lagos | 0.679573249 | Middle SDI |
| 25343 | Nasarawa | 0.518505244 | Low-middle SDI |

| Location ID | Location Name | 2021 SDI Index Value | SDI Quintile |
|---|---|---|---|
| 25344 | Niger | 0.42518919 | Low SDI |
| 25345 | Ogun | 0.569648186 | Low-middle SDI |
| 25346 | Ondo | 0.592384037 | Low-middle SDI |
| 25347 | Osun | 0.627939478 | Middle SDI |
| 25348 | Oyo | 0.584428907 | Low-middle SDI |
| 25349 | Plateau | 0.530865998 | Low-middle SDI |
| 25350 | Rivers | 0.638605802 | Middle SDI |
| 25351 | Sokoto | 0.254081326 | Low SDI |
| 25352 | Taraba | 0.438659637 | Low SDI |
| 25353 | Yobe | 0.312002634 | Low SDI |
| 25354 | Zamfara | 0.267800369 | Low SDI |
| 35424 | Hokkaidō | 0.844008657 | High SDI |
| 35425 | Aomori | 0.828693902 | High SDI |
| 35426 | Iwate | 0.835652812 | High SDI |
| 35427 | Miyagi | 0.860058983 | High SDI |
| 35428 | Akita | 0.832380775 | High SDI |

| Location ID | Location Name | 2021 SDI Index Value | SDI Quintile |
|---|---|---|---|
| 35429 | Yamagata | 0.838535343 | High SDI |
| 35430 | Fukushima | 0.841218485 | High SDI |
| 35431 | Ibaraki | 0.860523394 | High SDI |
| 35432 | Tochigi | 0.861256293 | High SDI |
| 35433 | Gunma | 0.861770439 | High SDI |
| 35434 | Saitama | 0.85655373 | High SDI |
| 35435 | Chiba | 0.861645419 | High SDI |
| 35436 | Tōkyō | 0.929043198 | High SDI |
| 35437 | Kanagawa | 0.882743596 | High SDI |
| 35438 | Niigata | 0.845233683 | High SDI |
| 35439 | Toyama | 0.865453766 | High SDI |
| 35440 | Ishikawa | 0.860214615 | High SDI |
| 35441 | Fukui | 0.856579697 | High SDI |
| 35442 | Yamanashi | 0.858173518 | High SDI |
| 35443 | Nagano | 0.858966672 | High SDI |
| 35444 | Gifu | 0.853447894 | High SDI |

| Location ID | Location Name | 2021 SDI Index Value | SDI Quintile |
|---|---|---|---|
| 35445 | Shizuoka | 0.865720709 | High SDI |
| 35446 | Aichi | 0.883329296 | High SDI |
| 35447 | Mie | 0.860795402 | High SDI |
| 35448 | Shiga | 0.874377981 | High SDI |
| 35449 | Kyōto | 0.876288854 | High SDI |
| 35450 | Ōsaka | 0.876433199 | High SDI |
| 35451 | Hyōgo | 0.868490782 | High SDI |
| 35452 | Nara | 0.851417382 | High SDI |
| 35453 | Wakayama | 0.847330156 | High SDI |
| 35454 | Tottori | 0.836091465 | High SDI |
| 35455 | Shimane | 0.838873648 | High SDI |
| 35456 | Okayama | 0.862350161 | High SDI |
| 35457 | Hiroshima | 0.870170469 | High SDI |
| 35458 | Yamaguchi | 0.856635725 | High SDI |
| 35459 | Tokushima | 0.85729815 | High SDI |
| 35460 | Kagawa | 0.857948449 | High SDI |

| Location ID | Location Name | 2021 SDI Index Value | SDI Quintile |
| --- | --- | --- | --- |
| 35461 | Ehime | 0.843779842 | High SDI |
| 35462 | Kōchi | 0.835890247 | High SDI |
| 35463 | Fukuoka | 0.858415047 | High SDI |
| 35464 | Saga | 0.835872528 | High SDI |
| 35465 | Nagasaki | 0.829106338 | High SDI |
| 35466 | Kumamoto | 0.834728198 | High SDI |
| 35467 | Ōita | 0.848715974 | High SDI |
| 35468 | Miyazaki | 0.826768586 | High SDI |
| 35469 | Kagoshima | 0.832472168 | High SDI |
| 35470 | Okinawa | 0.821640127 | High SDI |
| 35494 | Piemonte | 0.806813454 | High-middle SDI |
| 35495 | Valle d'Aosta | 0.812706016 | High SDI |
| 35496 | Liguria | 0.821521126 | High SDI |
| 35497 | Lombardia | 0.829091495 | High SDI |
| 35498 | Provincia autonoma di Bolzano | 0.838907386 | High SDI |
| 35499 | Provincia autonoma di Trento | 0.829531919 | High SDI |

| Location ID | Location Name | 2021 SDI Index Value | SDI Quintile |
|---|---|---|---|
| 35500 | Veneto | 0.808196606 | High-middle SDI |
| 35501 | Friuli-Venezia Giulia | 0.819858092 | High SDI |
| 35502 | Emilia-Romagna | 0.829695157 | High SDI |
| 35503 | Toscana | 0.811485035 | High SDI |
| 35504 | Umbria | 0.799092255 | High-middle SDI |
| 35505 | Marche | 0.803749028 | High-middle SDI |
| 35506 | Lazio | 0.827195546 | High SDI |
| 35507 | Abruzzo | 0.816496827 | High SDI |
| 35508 | Molise | 0.787791453 | High-middle SDI |
| 35509 | Campania | 0.766832076 | High-middle SDI |
| 35510 | Puglia | 0.763946911 | High-middle SDI |
| 35511 | Basilicata | 0.783798467 | High-middle SDI |
| 35512 | Calabria | 0.775304844 | High-middle SDI |
| 35513 | Sicilia | 0.76255912 | High-middle SDI |
| 35514 | Sardegna | 0.772644153 | High-middle SDI |
| 35617 | Baringo | 0.514621991 | Low-middle SDI |

| Location ID | Location Name | 2021 SDI Index Value | SDI Quintile |
|---|---|---|---|
| 35618 | Bomet | 0.530035509 | Low-middle SDI |
| 35619 | Bungoma | 0.488274636 | Low-middle SDI |
| 35620 | Busia | 0.478169739 | Low-middle SDI |
| 35621 | Elgeyo-Marakwet | 0.525285148 | Low-middle SDI |
| 35622 | Embu | 0.548377806 | Low-middle SDI |
| 35623 | Garissa | 0.32165019 | Low SDI |
| 35624 | Homa Bay | 0.507694781 | Low-middle SDI |
| 35625 | Isiolo | 0.435133975 | Low SDI |
| 35626 | Kajiado | 0.501634865 | Low-middle SDI |
| 35627 | Kakamega | 0.508518514 | Low-middle SDI |
| 35628 | Kericho | 0.520411743 | Low-middle SDI |
| 35629 | Kiambu | 0.593046232 | Low-middle SDI |
| 35630 | Kilifi | 0.486686815 | Low-middle SDI |
| 35631 | Kirinyaga | 0.546740689 | Low-middle SDI |
| 35632 | Kisii | 0.549398782 | Low-middle SDI |
| 35633 | Kisumu | 0.548720604 | Low-middle SDI |

| Location ID | Location Name | 2021 SDI Index Value | SDI Quintile |
|---|---|---|---|
| 35634 | Kitui | 0.474177297 | Low-middle SDI |
| 35635 | Kwale | 0.47965716 | Low-middle SDI |
| 35636 | Laikipia | 0.576686523 | Low-middle SDI |
| 35637 | Lamu | 0.505575183 | Low-middle SDI |
| 35638 | Machakos | 0.55015778 | Low-middle SDI |
| 35639 | Makueni | 0.514230695 | Low-middle SDI |
| 35640 | Mandera | 0.239926734 | Low SDI |
| 35641 | Marsabit | 0.399976958 | Low SDI |
| 35642 | Meru | 0.509152437 | Low-middle SDI |
| 35643 | Migori | 0.482207014 | Low-middle SDI |
| 35644 | Mombasa | 0.598166665 | Low-middle SDI |
| 35645 | Murang'a | 0.55292203 | Low-middle SDI |
| 35646 | Nairobi | 0.684188978 | Middle SDI |
| 35647 | Nakuru | 0.5721094 | Low-middle SDI |
| 35648 | Nandi | 0.516039708 | Low-middle SDI |
| 35649 | Narok | 0.458227005 | Low SDI |

| Location ID | Location Name | 2021 SDI Index Value | SDI Quintile |
|---|---|---|---|
| 35650 | Nyamira | 0.593029203 | Low-middle SDI |
| 35651 | Nyandarua | 0.577540201 | Low-middle SDI |
| 35652 | Nyeri | 0.579362636 | Low-middle SDI |
| 35653 | Samburu | 0.371475608 | Low SDI |
| 35654 | Siaya | 0.484421932 | Low-middle SDI |
| 35655 | Taita Taveta | 0.542579215 | Low-middle SDI |
| 35656 | Tana River | 0.389358757 | Low SDI |
| 35657 | Tharaka Nithi | 0.528624517 | Low-middle SDI |
| 35658 | Trans Nzoia | 0.549784994 | Low-middle SDI |
| 35659 | Turkana | 0.3683857 | Low SDI |
| 35660 | Uasin Gishu | 0.567922916 | Low-middle SDI |
| 35661 | Vihiga | 0.527054687 | Low-middle SDI |
| 35662 | Wajir | 0.258713923 | Low SDI |
| 35663 | West Pokot | 0.44769021 | Low SDI |
| 43872 | Andhra Pradesh, Urban | 0.647551072 | Middle SDI |
| 43873 | Arunachal Pradesh, Urban | 0.68419449 | Middle SDI |

| Location ID | Location Name | 2021 SDI Index Value | SDI Quintile |
|---|---|---|---|
| 43874 | Assam, Urban | 0.675446354 | Middle SDI |
| 43875 | Bihar, Urban | 0.591183966 | Low-middle SDI |
| 43877 | Chhattisgarh, Urban | 0.679763349 | Middle SDI |
| 43880 | Delhi, Urban | 0.730151189 | High-middle SDI |
| 43881 | Goa, Urban | 0.741223296 | High-middle SDI |
| 43882 | Gujarat, Urban | 0.703282867 | Middle SDI |
| 43883 | Haryana, Urban | 0.703932889 | Middle SDI |
| 43884 | Himachal Pradesh, Urban | 0.748161993 | High-middle SDI |
| 43885 | Jammu & Kashmir and Ladakh, Urban | 0.684343769 | Middle SDI |
| 43886 | Jharkhand, Urban | 0.670458961 | Middle SDI |
| 43887 | Karnataka, Urban | 0.658831954 | Middle SDI |
| 43888 | Kerala, Urban | 0.672698917 | Middle SDI |
| 43890 | Madhya Pradesh, Urban | 0.675329604 | Middle SDI |
| 43891 | Maharashtra, Urban | 0.705231591 | Middle SDI |
| 43892 | Manipur, Urban | 0.632756357 | Middle SDI |
| 43893 | Meghalaya, Urban | 0.685725867 | Middle SDI |

| Location ID | Location Name | 2021 SDI Index Value | SDI Quintile |
|---|---|---|---|
| 43894 | Mizoram, Urban | 0.670528706 | Middle SDI |
| 43895 | Nagaland, Urban | 0.691617405 | Middle SDI |
| 43896 | Odisha, Urban | 0.657514066 | Middle SDI |
| 43898 | Punjab, Urban | 0.698430939 | Middle SDI |
| 43899 | Rajasthan, Urban | 0.671757713 | Middle SDI |
| 43900 | Sikkim, Urban | 0.695740102 | Middle SDI |
| 43901 | Tamil Nadu, Urban | 0.676173806 | Middle SDI |
| 43902 | Telangana, Urban | 0.667241582 | Middle SDI |
| 43903 | Tripura, Urban | 0.633348097 | Middle SDI |
| 43904 | Uttar Pradesh, Urban | 0.63211101 | Middle SDI |
| 43905 | Uttarakhand, Urban | 0.746776963 | High-middle SDI |
| 43906 | West Bengal, Urban | 0.6493937 | Middle SDI |
| 43908 | Andhra Pradesh, Rural | 0.495920251 | Low-middle SDI |
| 43909 | Arunachal Pradesh, Rural | 0.549469207 | Low-middle SDI |
| 43910 | Assam, Rural | 0.551639812 | Low-middle SDI |
| 43911 | Bihar, Rural | 0.434277772 | Low SDI |

| Location ID | Location Name | 2021 SDI Index Value | SDI Quintile |
|---|---|---|---|
| 43913 | Chhattisgarh, Rural | 0.486779046 | Low-middle SDI |
| 43916 | Delhi, Rural | 0.655467765 | Middle SDI |
| 43917 | Goa, Rural | 0.687517157 | Middle SDI |
| 43918 | Gujarat, Rural | 0.549296846 | Low-middle SDI |
| 43919 | Haryana, Rural | 0.579178153 | Low-middle SDI |
| 43920 | Himachal Pradesh, Rural | 0.629512224 | Middle SDI |
| 43921 | Jammu & Kashmir and Ladakh, Rural | 0.572664356 | Low-middle SDI |
| 43922 | Jharkhand, Rural | 0.463971644 | Low SDI |
| 43923 | Karnataka, Rural | 0.526848679 | Low-middle SDI |
| 43924 | Kerala, Rural | 0.659487084 | Middle SDI |
| 43926 | Madhya Pradesh, Rural | 0.464482329 | Low SDI |
| 43927 | Maharashtra, Rural | 0.574895865 | Low-middle SDI |
| 43928 | Manipur, Rural | 0.560840633 | Low-middle SDI |
| 43929 | Meghalaya, Rural | 0.519625901 | Low-middle SDI |
| 43930 | Mizoram, Rural | 0.57456484 | Low-middle SDI |
| 43931 | Nagaland, Rural | 0.600219084 | Low-middle SDI |

| Location ID | Location Name | 2021 SDI Index Value | SDI Quintile |
|---|---|---|---|
| 43932 | Odisha, Rural | 0.520670909 | Low-middle SDI |
| 43934 | Punjab, Rural | 0.585512634 | Low-middle SDI |
| 43935 | Rajasthan, Rural | 0.478194737 | Low-middle SDI |
| 43936 | Sikkim, Rural | 0.593976905 | Low-middle SDI |
| 43937 | Tamil Nadu, Rural | 0.580536414 | Low-middle SDI |
| 43938 | Telangana, Rural | 0.483176983 | Low-middle SDI |
| 43939 | Tripura, Rural | 0.515999282 | Low-middle SDI |
| 43940 | Uttar Pradesh, Rural | 0.490024946 | Low-middle SDI |
| 43941 | Uttarakhand, Rural | 0.61464175 | Low-middle SDI |
| 43942 | West Bengal, Rural | 0.502428983 | Low-middle SDI |
| 44533 | China (without Hong Kong and Macao) | 0.717406624 | High-middle SDI |
| 44538 | Other Union Territories | 0.676062684 | Middle SDI |
| 44539 | Other Union Territories, Rural | 0.601933292 | Low-middle SDI |
| 44540 | Other Union Territories, Urban | 0.708204745 | Middle SDI |
| 44643 | Darlington | 0.835427723 | High SDI |
| 44644 | Northumberland | 0.822333819 | High SDI |

| Location ID | Location Name | 2021 SDI Index Value | SDI Quintile |
|---|---|---|---|
| 44645 | Stockton-on-Tees | 0.829517602 | High SDI |
| 44646 | Newcastle upon Tyne | 0.871588115 | High SDI |
| 44647 | North Tyneside | 0.835247504 | High SDI |
| 44648 | Redcar and Cleveland | 0.796360243 | High-middle SDI |
| 44649 | County Durham | 0.810754624 | High SDI |
| 44650 | Gateshead | 0.828579746 | High SDI |
| 44651 | Middlesbrough | 0.798976656 | High-middle SDI |
| 44652 | South Tyneside | 0.799489743 | High-middle SDI |
| 44653 | Sunderland | 0.817933435 | High SDI |
| 44654 | Hartlepool | 0.797297174 | High-middle SDI |
| 44655 | Cheshire East | 0.884278805 | High SDI |
| 44656 | Stockport | 0.861014705 | High SDI |
| 44657 | Trafford | 0.896679044 | High SDI |
| 44658 | Cheshire West and Chester | 0.870242062 | High SDI |
| 44659 | Sefton | 0.825438177 | High SDI |
| 44660 | Lancashire | 0.839626361 | High SDI |

| Location ID | Location Name | 2021 SDI Index Value | SDI Quintile |
|---|---|---|---|
| 44661 | Cumbria | 0.842325739 | High SDI |
| 44662 | Bolton | 0.812595954 | High SDI |
| 44663 | Wirral | 0.818111684 | High SDI |
| 44664 | Bury | 0.828816843 | High SDI |
| 44665 | St Helens | 0.810914124 | High SDI |
| 44666 | Warrington | 0.878129342 | High SDI |
| 44667 | Oldham | 0.796704511 | High-middle SDI |
| 44668 | Rochdale | 0.800322186 | High-middle SDI |
| 44669 | Wigan | 0.80548334 | High-middle SDI |
| 44670 | Halton | 0.835687566 | High SDI |
| 44671 | Liverpool | 0.847483043 | High SDI |
| 44672 | Tameside | 0.798557943 | High-middle SDI |
| 44673 | Salford | 0.837393179 | High SDI |
| 44674 | Blackburn with Darwen | 0.810134059 | High SDI |
| 44675 | Knowsley | 0.811985259 | High SDI |
| 44676 | Blackpool | 0.788904606 | High-middle SDI |

| Location ID | Location Name | 2021 SDI Index Value | SDI Quintile |
|---|---|---|---|
| 44677 | Manchester | 0.88057256 | High SDI |
| 44678 | North Yorkshire | 0.855520996 | High SDI |
| 44679 | East Riding of Yorkshire | 0.835193656 | High SDI |
| 44680 | York | 0.888110353 | High SDI |
| 44681 | North East Lincolnshire | 0.803563778 | High-middle SDI |
| 44682 | Calderdale | 0.836996359 | High SDI |
| 44683 | North Lincolnshire | 0.824582845 | High SDI |
| 44684 | Bradford | 0.814923693 | High SDI |
| 44685 | Kirklees | 0.823525465 | High SDI |
| 44686 | Leeds | 0.86787974 | High SDI |
| 44687 | Sheffield | 0.854247178 | High SDI |
| 44688 | Wakefield | 0.804506533 | High-middle SDI |
| 44689 | Rotherham | 0.803331209 | High-middle SDI |
| 44690 | Doncaster | 0.793628472 | High-middle SDI |
| 44691 | Kingston upon Hull, City of | 0.797611011 | High-middle SDI |
| 44692 | Barnsley | 0.788111758 | High-middle SDI |

| Location ID | Location Name | 2021 SDI Index Value | SDI Quintile |
|---|---|---|---|
| 44693 | Northamptonshire | 0.839033237 | High SDI |
| 44694 | Leicestershire | 0.851100665 | High SDI |
| 44695 | Lincolnshire | 0.820522815 | High SDI |
| 44696 | Rutland | 0.852195785 | High SDI |
| 44697 | Derby | 0.844076935 | High SDI |
| 44698 | Derbyshire | 0.823174583 | High SDI |
| 44699 | Nottinghamshire | 0.822432787 | High SDI |
| 44700 | Nottingham | 0.858455251 | High SDI |
| 44701 | Leicester | 0.828051202 | High SDI |
| 44702 | Warwickshire | 0.865693767 | High SDI |
| 44703 | Herefordshire, County of | 0.846194561 | High SDI |
| 44704 | Solihull | 0.871566638 | High SDI |
| 44705 | Shropshire | 0.842380445 | High SDI |
| 44706 | Worcestershire | 0.842681308 | High SDI |
| 44707 | Staffordshire | 0.828181727 | High SDI |
| 44708 | Dudley | 0.802585915 | High-middle SDI |

| Location ID | Location Name | 2021 SDI Index Value | SDI Quintile |
|---|---|---|---|
| 44709 | Coventry | 0.847335743 | High SDI |
| 44710 | Telford and Wrekin | 0.826093832 | High SDI |
| 44711 | Stoke-on-Trent | 0.796441727 | High-middle SDI |
| 44712 | Walsall | 0.790654736 | High-middle SDI |
| 44713 | Wolverhampton | 0.810887602 | High SDI |
| 44714 | Birmingham | 0.836949232 | High SDI |
| 44715 | Sandwell | 0.793668776 | High-middle SDI |
| 44716 | Bedford | 0.856962284 | High SDI |
| 44717 | Central Bedfordshire | 0.851318833 | High SDI |
| 44718 | Suffolk | 0.840388992 | High SDI |
| 44719 | Hertfordshire | 0.886963263 | High SDI |
| 44720 | Essex | 0.844953071 | High SDI |
| 44721 | Cambridgeshire | 0.887630336 | High SDI |
| 44722 | Thurrock | 0.818629818 | High SDI |
| 44723 | Norfolk | 0.836988602 | High SDI |
| 44724 | Southend-on-Sea | 0.825018651 | High SDI |

| Location ID | Location Name | 2021 SDI Index Value | SDI Quintile |
|---|---|---|---|
| 44725 | Peterborough | 0.837008458 | High SDI |
| 44726 | Luton | 0.838003231 | High SDI |
| 44727 | Richmond upon Thames | 0.932021729 | High SDI |
| 44728 | Kensington and Chelsea | 0.946366051 | High SDI |
| 44729 | Barnet | 0.885110546 | High SDI |
| 44730 | Westminster | 0.93701032 | High SDI |
| 44731 | Bromley | 0.869193749 | High SDI |
| 44732 | Bexley | 0.844097911 | High SDI |
| 44733 | Redbridge | 0.849278219 | High SDI |
| 44734 | Merton | 0.887252238 | High SDI |
| 44735 | Brent | 0.858683624 | High SDI |
| 44736 | Hillingdon | 0.892536477 | High SDI |
| 44737 | Havering | 0.834176853 | High SDI |
| 44738 | Kingston upon Thames | 0.908394132 | High SDI |
| 44739 | Sutton | 0.857530229 | High SDI |
| 44740 | Harrow | 0.858244679 | High SDI |

| Location ID | Location Name | 2021 SDI Index Value | SDI Quintile |
|---|---|---|---|
| 44741 | Enfield | 0.845516817 | High SDI |
| 44742 | Croydon | 0.851439949 | High SDI |
| 44743 | Hammersmith and Fulham | 0.934892187 | High SDI |
| 44744 | Ealing | 0.882007048 | High SDI |
| 44745 | Greenwich | 0.845443655 | High SDI |
| 44746 | Wandsworth | 0.924121015 | High SDI |
| 44747 | Waltham Forest | 0.840038834 | High SDI |
| 44748 | Camden | 0.936076172 | High SDI |
| 44749 | Lambeth | 0.916015975 | High SDI |
| 44750 | Lewisham | 0.85672931 | High SDI |
| 44751 | Hounslow | 0.896360928 | High SDI |
| 44752 | Southwark | 0.919165412 | High SDI |
| 44753 | Newham | 0.840477768 | High SDI |
| 44754 | Barking and Dagenham | 0.80642649 | High-middle SDI |
| 44755 | Haringey | 0.8714663 | High SDI |
| 44756 | Hackney | 0.891329222 | High SDI |

| Location ID | Location Name | 2021 SDI Index Value | SDI Quintile |
|---|---|---|---|
| 44757 | Islington | 0.924302624 | High SDI |
| 44758 | Tower Hamlets | 0.903700654 | High SDI |
| 44759 | Wokingham | 0.910821902 | High SDI |
| 44760 | Buckinghamshire | 0.888192339 | High SDI |
| 44761 | Surrey | 0.904483995 | High SDI |
| 44762 | Windsor and Maidenhead | 0.915543426 | High SDI |
| 44763 | West Berkshire | 0.897124589 | High SDI |
| 44764 | Hampshire | 0.87199888 | High SDI |
| 44765 | Bracknell Forest | 0.890716234 | High SDI |
| 44766 | West Sussex | 0.863885895 | High SDI |
| 44767 | Oxfordshire | 0.899135231 | High SDI |
| 44768 | Reading | 0.90527187 | High SDI |
| 44769 | Kent | 0.844500542 | High SDI |
| 44770 | Brighton and Hove | 0.897901251 | High SDI |
| 44771 | Medway | 0.819461594 | High SDI |
| 44772 | East Sussex | 0.83898657 | High SDI |

| Location ID | Location Name | 2021 SDI Index Value | SDI Quintile |
|---|---|---|---|
| 44773 | Portsmouth | 0.864778156 | High SDI |
| 44774 | Isle of Wight | 0.826023587 | High SDI |
| 44775 | Milton Keynes | 0.886756129 | High SDI |
| 44776 | Southampton | 0.860211008 | High SDI |
| 44777 | Slough | 0.877374496 | High SDI |
| 44778 | South Gloucestershire | 0.88411864 | High SDI |
| 44779 | Dorset | 0.8514167 | High SDI |
| 44780 | Wiltshire | 0.859020608 | High SDI |
| 44781 | North Somerset | 0.85864883 | High SDI |
| 44782 | Devon | 0.854622345 | High SDI |
| 44783 | Poole | 0.862990852 | High SDI |
| 44784 | Bath and North East Somerset | 0.895075988 | High SDI |
| 44785 | Gloucestershire | 0.870918382 | High SDI |
| 44786 | Somerset | 0.842820976 | High SDI |
| 44787 | Swindon | 0.86670182 | High SDI |
| 44788 | Torbay | 0.812459589 | High SDI |

| Location ID | Location Name | 2021 SDI Index Value | SDI Quintile |
|---|---|---|---|
| 44789 | Bristol, City of | 0.896565514 | High SDI |
| 44790 | Bournemouth | 0.870149525 | High SDI |
| 44791 | Cornwall | 0.839219489 | High SDI |
| 44792 | Plymouth | 0.84217698 | High SDI |
| 44793 | Central | 0.577789494 | Low-middle SDI |
| 44794 | Coast | 0.518014043 | Low-middle SDI |
| 44795 | Eastern | 0.508217906 | Low-middle SDI |
| 44796 | Nairobi | 0.684188971 | Middle SDI |
| 44797 | North Eastern | 0.264360533 | Low SDI |
| 44798 | Nyanza | 0.525077558 | Low-middle SDI |
| 44799 | Rift Valley | 0.51425552 | Low-middle SDI |
| 44800 | Western | 0.504497721 | Low-middle SDI |
| 44850 | New Zealand Maori population | 0.770791784 | High-middle SDI |
| 44851 | New Zealand non-Maori population | 0.870812225 | High SDI |
| 44852 | Tigray | 0.38400255 | Low SDI |
| 44853 | Afar | 0.286502778 | Low SDI |

| Location ID | Location Name | 2021 SDI Index Value | SDI Quintile |
|---|---|---|---|
| 44854 | Amhara | 0.322156172 | Low SDI |
| 44855 | Oromia | 0.337961203 | Low SDI |
| 44856 | Somali | 0.27014767 | Low SDI |
| 44857 | Benishangul-Gumuz | 0.323654895 | Low SDI |
| 44858 | Southern Nations, Nationalities, and Peoples | 0.357248469 | Low SDI |
| 44859 | Harari | 0.539739601 | Low-middle SDI |
| 44860 | Gambella | 0.460576186 | Low SDI |
| 44861 | Addis Ababa | 0.695287927 | Middle SDI |
| 44862 | Dire Dawa | 0.542617703 | Low-middle SDI |
| 44864 | Alborz | 0.748208954 | High-middle SDI |
| 44865 | Ardebil | 0.658777858 | Middle SDI |
| 44866 | East Azarbayejan | 0.667933993 | Middle SDI |
| 44867 | West Azarbayejan | 0.626918833 | Middle SDI |
| 44868 | Bushehr | 0.708677286 | Middle SDI |
| 44869 | Chahar Mahaal and Bakhtiari | 0.678339055 | Middle SDI |
| 44870 | Fars | 0.715109154 | High-middle SDI |

| Location ID | Location Name | 2021 SDI Index Value | SDI Quintile |
|---|---|---|---|
| 44871 | Gilan | 0.712361968 | High-middle SDI |
| 44872 | Golestan | 0.656422158 | Middle SDI |
| 44873 | Hamadan | 0.666968762 | Middle SDI |
| 44874 | Hormozgan | 0.670775004 | Middle SDI |
| 44875 | Ilam | 0.705185457 | Middle SDI |
| 44876 | Isfahan | 0.709893952 | Middle SDI |
| 44877 | Kerman | 0.66878396 | Middle SDI |
| 44878 | Kermanshah | 0.674511267 | Middle SDI |
| 44879 | North Khorasan | 0.651483141 | Middle SDI |
| 44880 | Khorasan-e-Razavi | 0.67053956 | Middle SDI |
| 44881 | South Khorasan | 0.653364832 | Middle SDI |
| 44882 | Khuzestan | 0.669816556 | Middle SDI |
| 44883 | Kohgiluyeh and Boyer-Ahmad | 0.694488035 | Middle SDI |
| 44884 | Kurdistan | 0.642334326 | Middle SDI |
| 44885 | Lorestan | 0.669421197 | Middle SDI |
| 44886 | Markazi | 0.682789151 | Middle SDI |

| Location ID | Location Name | 2021 SDI Index Value | SDI Quintile |
|---|---|---|---|
| 44887 | Mazandaran | 0.729935836 | High-middle SDI |
| 44888 | Qazvin | 0.687516004 | Middle SDI |
| 44889 | Qom | 0.694133773 | Middle SDI |
| 44890 | Semnan | 0.724001841 | High-middle SDI |
| 44891 | Sistan and Baluchistan | 0.549869409 | Low-middle SDI |
| 44892 | Tehran | 0.776102826 | High-middle SDI |
| 44893 | Yazd | 0.713637577 | High-middle SDI |
| 44894 | Zanjan | 0.661463751 | Middle SDI |
| 44903 | Belgorod oblast | 0.803695074 | High-middle SDI |
| 44904 | Bryansk oblast | 0.792329315 | High-middle SDI |
| 44905 | Vladimir oblast | 0.788797618 | High-middle SDI |
| 44906 | Voronezh oblast | 0.810406591 | High SDI |
| 44907 | Ivanovo oblast | 0.789631875 | High-middle SDI |
| 44908 | Kaluga oblast | 0.800527701 | High-middle SDI |
| 44909 | Kostroma oblast | 0.787756437 | High-middle SDI |
| 44910 | Kursk oblast | 0.793649987 | High-middle SDI |

| Location ID | Location Name | 2021 SDI Index Value | SDI Quintile |
|---|---|---|---|
| 44911 | Lipetzk oblast | 0.802916176 | High-middle SDI |
| 44912 | Moscow oblast | 0.828120859 | High SDI |
| 44913 | Oryol oblast | 0.79451294 | High-middle SDI |
| 44914 | Ryazan oblast | 0.793693116 | High-middle SDI |
| 44915 | Smolensk oblast | 0.800950484 | High-middle SDI |
| 44916 | Tambov oblast | 0.790982828 | High-middle SDI |
| 44917 | Tver oblast | 0.799102213 | High-middle SDI |
| 44918 | Tula oblast | 0.797660828 | High-middle SDI |
| 44919 | Yaroslavl oblast | 0.805364359 | High-middle SDI |
| 44920 | Moscow City | 0.887772609 | High SDI |
| 44921 | Republic of Karelia | 0.81238914 | High SDI |
| 44922 | Komi Republic | 0.790079817 | High-middle SDI |
| 44923 | Nenets autonomous district | 0.800997369 | High-middle SDI |
| 44924 | Arkhangelsk oblast without Nenets autonomous district | 0.812017693 | High SDI |
| 44925 | Vologda oblast | 0.786532281 | High-middle SDI |
| 44926 | Kaliningrad oblast | 0.803693646 | High-middle SDI |

| Location ID | Location Name | 2021 SDI Index Value | SDI Quintile |
|---|---|---|---|
| 44927 | Leningrad oblast | 0.814446452 | High SDI |
| 44928 | Murmansk oblast | 0.829529044 | High SDI |
| 44929 | Novgorod oblast | 0.779643289 | High-middle SDI |
| 44930 | Pskov oblast | 0.787746847 | High-middle SDI |
| 44931 | Saint Petersburg | 0.859435837 | High SDI |
| 44932 | Republic of Adygeya | 0.783355473 | High-middle SDI |
| 44933 | Republic of Kalmykia | 0.754503495 | High-middle SDI |
| 44934 | Republic of Crimea | 0.713851386 | High-middle SDI |
| 44935 | Krasnodar kray | 0.799215014 | High-middle SDI |
| 44936 | Astrakhan oblast | 0.777754786 | High-middle SDI |
| 44937 | Volgograd oblast | 0.792090776 | High-middle SDI |
| 44938 | Rostov oblast | 0.804560133 | High-middle SDI |
| 44939 | Sevastopol | 0.778562755 | High-middle SDI |
| 44940 | Republic of Dagestan | 0.75489971 | High-middle SDI |
| 44941 | Republic of Ingushetia | 0.723181052 | High-middle SDI |
| 44942 | Kabardino-Balkar Republic | 0.773368211 | High-middle SDI |

| Location ID | Location Name | 2021 SDI Index Value | SDI Quintile |
|---|---|---|---|
| 44943 | Karachay-Cherkess Republic | 0.756823567 | High-middle SDI |
| 44944 | Republic of North Ossetia-Alania | 0.792451998 | High-middle SDI |
| 44945 | Chechen Republic | 0.697000298 | Middle SDI |
| 44946 | Stavropol kray | 0.785308224 | High-middle SDI |
| 44947 | Republic of Bashkortostan | 0.807048582 | High-middle SDI |
| 44948 | Republic of Mari El | 0.777484633 | High-middle SDI |
| 44949 | Republic of Mordovia | 0.77934414 | High-middle SDI |
| 44950 | Republic of Tatarstan | 0.815624973 | High SDI |
| 44951 | Udmurt Republic | 0.786843627 | High-middle SDI |
| 44952 | Chuvash Republic | 0.779169152 | High-middle SDI |
| 44953 | Perm kray | 0.796955473 | High-middle SDI |
| 44954 | Kirov oblast | 0.785897475 | High-middle SDI |
| 44955 | Nizhny Novgorod oblast | 0.81057854 | High SDI |
| 44956 | Orenburg oblast | 0.78313786 | High-middle SDI |
| 44957 | Penza oblast | 0.790346819 | High-middle SDI |
| 44958 | Samara oblast | 0.814000588 | High SDI |

| Location ID | Location Name | 2021 SDI Index Value | SDI Quintile |
|---|---|---|---|
| 44959 | Saratov oblast | 0.785980203 | High-middle SDI |
| 44960 | Ulyanovsk oblast | 0.782641776 | High-middle SDI |
| 44961 | Kurgan oblast | 0.76552614 | High-middle SDI |
| 44962 | Sverdlovsk oblast | 0.816064746 | High SDI |
| 44963 | Khanty-Mansi autonomous area | 0.826709092 | High SDI |
| 44964 | Yamalo-Nenets autonomous area | 0.837700215 | High SDI |
| 44965 | Tyumen oblast without autonomous areas | 0.813017728 | High SDI |
| 44966 | Chelyabinsk oblast | 0.793022546 | High-middle SDI |
| 44967 | Republic of Altai | 0.749802645 | High-middle SDI |
| 44968 | Republic of Buryatia | 0.778616162 | High-middle SDI |
| 44969 | Republic of Tuva | 0.692513465 | Middle SDI |
| 44970 | Republic of Khakassia | 0.777318539 | High-middle SDI |
| 44971 | Altai kray | 0.777099855 | High-middle SDI |
| 44972 | Zabaikalsk kray | 0.740925147 | High-middle SDI |
| 44973 | Krasnoyarsk kray | 0.796852774 | High-middle SDI |
| 44974 | Irkutsk oblast | 0.77351672 | High-middle SDI |

| Location ID | Location Name | 2021 SDI Index Value | SDI Quintile |
|---|---|---|---|
| 44975 | Kemerovo oblast | 0.782797668 | High-middle SDI |
| 44976 | Novosibirsk oblast | 0.796528925 | High-middle SDI |
| 44977 | Omsk oblast | 0.791260764 | High-middle SDI |
| 44978 | Tomsk oblast | 0.802325795 | High-middle SDI |
| 44979 | Republic of Sakha (Yakutia) | 0.814649347 | High SDI |
| 44980 | Kamchatka kray | 0.798113769 | High-middle SDI |
| 44981 | Primorsky kray | 0.802659761 | High-middle SDI |
| 44982 | Khabarovsk kray | 0.818047393 | High SDI |
| 44983 | Amur oblast | 0.793881993 | High-middle SDI |
| 44984 | Magadan oblast | 0.830546268 | High SDI |
| 44985 | Sakhalin oblast | 0.822655388 | High SDI |
| 44986 | Jewish autonomous oblast | 0.765463494 | High-middle SDI |
| 44987 | Chukotka Autonomous Area | 0.800770455 | High-middle SDI |
| 50559 | Ukraine (without Crimea & Sevastopol) | 0.761743094 | High-middle SDI |
| 53432 | Trøndelag | 0.916773946 | High SDI |
| 53533 | Mountain Province | 0.51921863 | Low-middle SDI |

| Location ID | Location Name | 2021 SDI Index Value | SDI Quintile |
|---|---|---|---|
| 53534 | Ifugao | 0.597325624 | Low-middle SDI |
| 53535 | Benguet | 0.716293119 | High-middle SDI |
| 53536 | Abra | 0.654173651 | Middle SDI |
| 53537 | Apayao | 0.60770129 | Low-middle SDI |
| 53538 | Kalinga | 0.575239213 | Low-middle SDI |
| 53539 | La Union | 0.661006064 | Middle SDI |
| 53540 | Ilocos Norte | 0.687657015 | Middle SDI |
| 53541 | Ilocos Sur | 0.671836522 | Middle SDI |
| 53542 | Pangasinan | 0.666135554 | Middle SDI |
| 53543 | Nueva Vizcaya | 0.616609509 | Low-middle SDI |
| 53544 | Cagayan | 0.63433253 | Middle SDI |
| 53545 | Isabela | 0.636783935 | Middle SDI |
| 53546 | Quirino | 0.575885131 | Low-middle SDI |
| 53547 | Batanes | 0.682565947 | Middle SDI |
| 53548 | Bataan | 0.660374268 | Middle SDI |
| 53549 | Zambales | 0.65425423 | Middle SDI |

| Location ID | Location Name | 2021 SDI Index Value | SDI Quintile |
|---|---|---|---|
| 53550 | Tarlac | 0.650760386 | Middle SDI |
| 53551 | Pampanga | 0.697271285 | Middle SDI |
| 53552 | Bulacan | 0.708002625 | Middle SDI |
| 53553 | Nueva Ecija | 0.650591609 | Middle SDI |
| 53554 | Aurora | 0.614063114 | Low-middle SDI |
| 53555 | Rizal | 0.710524418 | Middle SDI |
| 53556 | Cavite | 0.72917856 | High-middle SDI |
| 53557 | Laguna | 0.701620417 | Middle SDI |
| 53558 | Batangas | 0.686055208 | Middle SDI |
| 53559 | Quezon | 0.630168626 | Middle SDI |
| 53560 | Occidental Mindoro | 0.46028876 | Low SDI |
| 53561 | Oriental Mindoro | 0.60555167 | Low-middle SDI |
| 53562 | Romblon | 0.527572654 | Low-middle SDI |
| 53563 | Palawan | 0.527211201 | Low-middle SDI |
| 53564 | Marinduque | 0.549650294 | Low-middle SDI |
| 53565 | Catanduanes | 0.60942324 | Low-middle SDI |

| Location ID | Location Name | 2021 SDI Index Value | SDI Quintile |
|---|---|---|---|
| 53566 | Camarines Norte | 0.594459098 | Low-middle SDI |
| 53567 | Sorsogon | 0.600776304 | Low-middle SDI |
| 53568 | Albay | 0.640151269 | Middle SDI |
| 53569 | Masbate | 0.458060424 | Low SDI |
| 53570 | Camarines Sur | 0.633743794 | Middle SDI |
| 53571 | Capiz | 0.5714003 | Low-middle SDI |
| 53572 | Aklan | 0.64042058 | Middle SDI |
| 53573 | Antique | 0.569823295 | Low-middle SDI |
| 53574 | Negros Occidental | 0.604235111 | Low-middle SDI |
| 53575 | Iloilo | 0.673508341 | Middle SDI |
| 53576 | Guimaras | 0.609860186 | Low-middle SDI |
| 53577 | Negros Oriental | 0.578181475 | Low-middle SDI |
| 53578 | Cebu | 0.658154663 | Middle SDI |
| 53579 | Bohol | 0.604933545 | Low-middle SDI |
| 53580 | Siquijor | 0.600945243 | Low-middle SDI |
| 53581 | Southern Leyte | 0.60149118 | Low-middle SDI |

| Location ID | Location Name | 2021 SDI Index Value | SDI Quintile |
|---|---|---|---|
| 53582 | Eastern Samar | 0.499800385 | Low-middle SDI |
| 53583 | Northern Samar | 0.52396754 | Low-middle SDI |
| 53584 | Samar (Western Samar) | 0.527590959 | Low-middle SDI |
| 53585 | Leyte | 0.611701754 | Low-middle SDI |
| 53586 | Biliran | 0.643027685 | Middle SDI |
| 53587 | Zamboanga Sibugay | 0.549420679 | Low-middle SDI |
| 53588 | Zamboanga Del Norte | 0.53803222 | Low-middle SDI |
| 53589 | Zamboanga Del Sur | 0.630995266 | Middle SDI |
| 53590 | Misamis Occidental | 0.588184738 | Low-middle SDI |
| 53591 | Bukidnon | 0.551058807 | Low-middle SDI |
| 53592 | Lanao Del Norte | 0.587617211 | Low-middle SDI |
| 53593 | Misamis Oriental | 0.662873429 | Middle SDI |
| 53594 | Camiguin | 0.632089767 | Middle SDI |
| 53595 | Davao Oriental | 0.547236917 | Low-middle SDI |
| 53596 | Davao de Oro | 0.533098715 | Low-middle SDI |
| 53597 | Davao Del Sur | 0.659917629 | Middle SDI |

| Location ID | Location Name | 2021 SDI Index Value | SDI Quintile |
|---|---|---|---|
| 53598 | Davao Occidental | 0.578314908 | Low-middle SDI |
| 53599 | Davao Del Norte | 0.636970822 | Middle SDI |
| 53600 | South Cotabato | 0.632536869 | Middle SDI |
| 53601 | Sultan Kudarat | 0.519757294 | Low-middle SDI |
| 53602 | Cotabato (North Cotabato) | 0.55211245 | Low-middle SDI |
| 53603 | Sarangani | 0.582418699 | Low-middle SDI |
| 53604 | Agusan Del Norte | 0.615707618 | Low-middle SDI |
| 53605 | Agusan Del Sur | 0.541666497 | Low-middle SDI |
| 53606 | Surigao Del Sur | 0.587486668 | Low-middle SDI |
| 53607 | Surigao Del Norte | 0.627545678 | Middle SDI |
| 53608 | Dinagat Islands | 0.622041414 | Middle SDI |
| 53609 | Tawi-Tawi | 0.535621341 | Low-middle SDI |
| 53610 | Basilan | 0.546108643 | Low-middle SDI |
| 53611 | Sulu | 0.48399862 | Low-middle SDI |
| 53612 | Maguindanao | 0.51070243 | Low-middle SDI |
| 53613 | Lanao Del Sur | 0.532909532 | Low-middle SDI |

| Location ID | Location Name | 2021 SDI Index Value | SDI Quintile |
|---|---|---|---|
| 53614 | National Capital Region | 0.751536473 | High-middle SDI |
| 53615 | Azad Jammu & Kashmir | 0.541342775 | Low-middle SDI |
| 53616 | Balochistan | 0.417109886 | Low SDI |
| 53617 | Gilgit-Baltistan | 0.399312068 | Low SDI |
| 53618 | Islamabad Capital Territory | 0.695559154 | Middle SDI |
| 53619 | Khyber Pakhtunkhwa | 0.451366327 | Low SDI |
| 53620 | Punjab | 0.520053339 | Low-middle SDI |
| 53621 | Sindh | 0.513737094 | Low-middle SDI |
| 53660 | Dolnośląskie | 0.817783922 | High SDI |
| 53661 | Kujawsko-Pomorskie | 0.790185071 | High-middle SDI |
| 53662 | Łódzkie | 0.805693677 | High-middle SDI |
| 53663 | Lubelskie | 0.789673817 | High-middle SDI |
| 53664 | Lubuskie | 0.791264292 | High-middle SDI |
| 53665 | Małopolskie | 0.812434112 | High SDI |
| 53666 | Mazowieckie | 0.849859302 | High SDI |
| 53667 | Opolskie | 0.794191353 | High-middle SDI |

| Location ID | Location Name | 2021 SDI Index Value | SDI Quintile |
|---|---|---|---|
| 53668 | Podkarpackie | 0.792282338 | High-middle SDI |
| 53669 | Podlaskie | 0.786166754 | High-middle SDI |
| 53670 | Pomorskie | 0.804282452 | High-middle SDI |
| 53671 | Śląskie | 0.81907325 | High SDI |
| 53672 | Świętokrzyskie | 0.796464119 | High-middle SDI |
| 53673 | Warmińsko-Mazurskie | 0.777449817 | High-middle SDI |
| 53674 | Wielkopolskie | 0.813257407 | High SDI |
| 53675 | Zachodniopomorskie | 0.792789122 | High-middle SDI |
| 60132 | Vestland | 0.917642172 | High SDI |
| 60133 | Agder | 0.907093137 | High SDI |
| 60134 | Vestfold og Telemark | 0.907351769 | High SDI |
| 60135 | Innlandet | 0.899977235 | High SDI |
| 60136 | Viken | 0.914602128 | High SDI |
| 60137 | Troms og Finnmark | 0.904453583 | High SDI |

**21GBD region and 5 SDI region specific distribution**

| Location |
| --- |
| **Global** |
| **Central Europe to Eastern Europe to and Central Asia** |
| Central Asia |
| Central Europe |
| Eastern Europe |
| **High-income** |
| Australasia |
| High-income Asia Pacific |
| High-income North America |
| Southern Latin America |
| Western Europe |
| **Latin America and Caribbean** |
| Andean Latin America |
| Caribbean |
| Central Latin America |

Tropical Latin America

**North Africa and Middle East**

North Africa and Middle East

**South Asia**

South Asia

**Southeast Asia to East Asia to and Oceania**

East Asia

Oceania

Southeast Asia

**Sub-Saharan Africa**

Central Sub-Saharan Africa

Eastern Sub-Saharan Africa

Southern Sub-Saharan Africa

Western Sub-Saharan Africa

**SDI region**

Low SDI

Low-middle SDI

| |
|---|
| Middle SDI |
| High-middle SDI |
| High SDI |

**Table S3 Absolute deaths and age-standardized death rate (ASDR) of NALC globally and regionally in 1990 and 2021, and their average annual percentage change (AAPC) from 1990 to 2021 across 204 countries and regions.**

| | Both | | | Men | | | Women | | |
|---|---|---|---|---|---|---|---|---|---|
| Location | Cases (95% UI) 2021 | ASDR (95% UI) 2021 | 1990-2021 AAPC (95% UI) | Cases (95% UI) 2021 | ASDR (95% UI) 2021 | 1990-2021AAPC (95% UI) | Cases (95% UI) 2021 | ASDR (95% UI) 2021 | 1990-2021 AAPC (95% UI) |
| Afghanistan | 62 (38 to 99) | 0.65 (0.38 to 1.11) | 0.69 (0.65 to 0.73) | 14 (8 to 24) | 0.36 (0.2 to 0.61) | 0.23 (0.03 to 0.43) | 47 (27 to 79) | 0.91 (0.49 to 1.63) | 0.68 (0.64 to 0.73) |
| Albania | 40 (23 to | 0.91 (0.53 | -0.91 | 21 (12 to | 1.02 (0.59 to | -0.67 | 19 (10 to | 0.8 (0.45 to | -1.59 |

| Location | Both Cases (95% UI) 2021 | Both ASDR (95% UI) 2021 | Both 1990-2021 AAPC (95% UI) | Men Cases (95% UI) 2021 | Men ASDR (95% UI) 2021 | Men 1990-2021AAPC (95% UI) | Women Cases (95% UI) 2021 | Women ASDR (95% UI) 2021 | Women 1990-2021 AAPC (95% UI) |
|---|---|---|---|---|---|---|---|---|---|
| | 65) | to 1.48) | (-1.36 to -0.47) | 35) | 1.68) | (-1.17 to -0.16) | 31) | 1.33) | (-2.15 to -1.03) |
| Algeria | 81 (51 to 118) | 0.24 (0.15 to 0.37) | 2.02 (1.71 to 2.32) | 40 (23 to 63) | 0.24 (0.14 to 0.38) | 2.25 (2.12 to 2.39) | 41 (25 to 61) | 0.26 (0.16 to 0.39) | 1.83 (1.58 to 2.07) |
| American Samoa | 0 (0 to 1) | 0.96 (0.63 to 1.4) | 1.87 (1.2 to 2.54) | 0 (0 to 0) | 0.83 (0.51 to 1.24) | 1.74 (1.08 to 2.41) | 0 (0 to 0) | 1.09 (0.69 to 1.63) | 1.93 (1.65 to 2.21) |
| Andorra | 1 (1 to 2) | 0.87 (0.51 to 1.39) | 0.31 (0.1 to 0.53) | 0 (0 to 1) | 0.52 (0.29 to 0.85) | 0.12 (-0.15 to 0.4) | 1 (1 to 2) | 1.22 (0.69 to 1.9) | 0.37 (0.16 to 0.58) |
| Angola | 76 (18 to 203) | 0.67 (0.16 to 1.78) | -0.85 (-1.03 to -0.67) | 40 (7 to 108) | 0.8 (0.14 to 2.08) | -0.87 (-1.18 to -0.56) | 35 (9 to 106) | 0.57 (0.13 to 1.78) | -0.7 (-0.99 to -0.41) |
| Antigua and Barbuda | 0 (0 to 0) | 0.28 (0.19 to 0.4) | 0.18 (-0.76 to 1.14) | 0 (0 to 0) | 0.33 (0.22 to 0.48) | 0.17 (-0.93 to 1.28) | 0 (0 to 0) | 0.24 (0.17 to 0.33) | 0.1 (-0.87 to 1.08) |
| Argentina | 63 (42 to 89) | 0.11 (0.07 to 0.16) | 3 (2.25 to 3.76) | 32 (21 to 48) | 0.13 (0.09 to 0.2) | 3.44 (2.8 to 4.08) | 30 (20 to 42) | 0.09 (0.06 to 0.13) | 2.6 (1.69 to 3.52) |

| Location | Both | | | Men | | | Women | | |
|---|---|---|---|---|---|---|---|---|---|
| | Cases (95% UI) 2021 | ASDR (95% UI) 2021 | 1990-2021 AAPC (95% UI) | Cases (95% UI) 2021 | ASDR (95% UI) 2021 | 1990-2021AAPC (95% UI) | Cases (95% UI) 2021 | ASDR (95% UI) 2021 | 1990-2021 AAPC (95% UI) |
| Armenia | 29 (19 to 42) | 0.66 (0.43 to 0.96) | -0.03 (-0.59 to 0.53) | 12 (8 to 18) | 0.65 (0.43 to 0.98) | 0.5 (-0.21 to 1.22) | 17 (11 to 26) | 0.66 (0.43 to 0.99) | -0.31 (-1.04 to 0.42) |
| Australia | 260 (179 to 365) | 0.57 (0.39 to 0.79) | 4.43 (3.94 to 4.92) | 137 (88 to 194) | 0.64 (0.42 to 0.91) | 4.21 (3.5 to 4.93) | 123 (81 to 175) | 0.5 (0.34 to 0.7) | 4.62 (4.13 to 5.11) |
| Austria | 58 (38 to 84) | 0.3 (0.2 to 0.43) | 2.22 (1.56 to 2.87) | 31 (20 to 46) | 0.38 (0.25 to 0.56) | 2.35 (2.15 to 2.56) | 26 (17 to 38) | 0.24 (0.16 to 0.34) | 2.01 (1.29 to 2.73) |
| Azerbaijan | 75 (38 to 137) | 0.8 (0.39 to 1.46) | 1.22 (0.94 to 1.5) | 34 (17 to 63) | 0.83 (0.4 to 1.51) | 1.41 (1.13 to 1.69) | 40 (20 to 77) | 0.77 (0.37 to 1.43) | 1.03 (0.65 to 1.4) |
| Bahamas | 1 (1 to 2) | 0.34 (0.23 to 0.49) | 0.13 (-0.35 to 0.61) | 1 (0 to 1) | 0.43 (0.29 to 0.63) | 1.01 (0.5 to 1.52) | 1 (0 to 1) | 0.26 (0.17 to 0.38) | -0.51 (-1.32 to 0.31) |
| Bahrain | 5 (3 to 8) | 0.83 (0.53 to 1.26) | -0.7 (-1.08 to -0.33) | 3 (2 to 5) | 0.94 (0.58 to 1.45) | -0.57 (-1.01 to -0.13) | 2 (1 to 3) | 0.75 (0.46 to 1.11) | -0.87 (-1.31 to -0.43) |
| Bangladesh | 280 (171 to | 0.21 (0.13 | 0.78 | 157 (90 to | 0.22 (0.13 to | 0.66 | 123 (71 to | 0.19 (0.11 to | 1.09 |

| Location | Both Cases (95% UI) 2021 | Both ASDR (95% UI) 2021 | Both 1990-2021 AAPC (95% UI) | Men Cases (95% UI) 2021 | Men ASDR (95% UI) 2021 | Men 1990-2021AAPC (95% UI) | Women Cases (95% UI) 2021 | Women ASDR (95% UI) 2021 | Women 1990-2021 AAPC (95% UI) |
|---|---|---|---|---|---|---|---|---|---|
| | 431) | to 0.32) | (0.42 to 1.14) | 257) | 0.36) | (0.37 to 0.95) | 192) | 0.29) | (0.64 to 1.55) |
| Barbados | 2 (1 to 2) | 0.31 (0.2 to 0.46) | 0.67 (-0.2 to 1.54) | 1 (0 to 1) | 0.26 (0.16 to 0.39) | 0.67 (-0.63 to 1.98) | 1 (1 to 2) | 0.36 (0.23 to 0.53) | 0.99 (0.38 to 1.6) |
| Belarus | 38 (24 to 55) | 0.24 (0.15 to 0.34) | 0.69 (0.07 to 1.3) | 19 (12 to 28) | 0.33 (0.21 to 0.49) | 1.43 (0.95 to 1.92) | 19 (12 to 28) | 0.18 (0.12 to 0.27) | 0.02 (-0.8 to 0.83) |
| Belgium | 66 (41 to 95) | 0.26 (0.17 to 0.38) | 1.2 (0.63 to 1.78) | 31 (20 to 47) | 0.29 (0.19 to 0.43) | 2.11 (1.51 to 2.71) | 34 (20 to 50) | 0.24 (0.15 to 0.34) | 0.65 (-0.08 to 1.4) |
| Belize | 1 (1 to 1) | 0.28 (0.18 to 0.39) | 1.29 (0.36 to 2.23) | 0 (0 to 0) | 0.23 (0.15 to 0.35) | 2.17 (1.23 to 3.12) | 0 (0 to 1) | 0.32 (0.21 to 0.45) | 0.89 (-0.32 to 2.11) |
| Benin | 86 (55 to 131) | 1.77 (1.12 to 2.74) | -0.51 (-0.64 to -0.37) | 36 (22 to 57) | 1.58 (0.98 to 2.51) | -0.9 (-1.11 to -0.68) | 50 (30 to 81) | 1.93 (1.14 to 3.18) | -0.2 (-0.34 to -0.05) |
| Bermuda | 0 (0 to 0) | 0.19 (0.12 to 0.28) | -1.92 (-2.5 to -1.34) | 0 (0 to 0) | 0.22 (0.15 to 0.33) | -1.11 (-1.79 to -0.43) | 0 (0 to 0) | 0.16 (0.1 to 0.24) | -2.42 (-3.17 to -1.66) |

| | Both | | | Men | | | Women | | |
|---|---|---|---|---|---|---|---|---|---|
| Location | Cases (95% UI) 2021 | ASDR (95% UI) 2021 | 1990-2021 AAPC (95% UI) | Cases (95% UI) 2021 | ASDR (95% UI) 2021 | 1990-2021AAPC (95% UI) | Cases (95% UI) 2021 | ASDR (95% UI) 2021 | 1990-2021 AAPC (95% UI) |
| Bhutan | 3 (2 to 4) | 0.47 (0.26 to 0.75) | 1.46 (1.39 to 1.54) | 2 (1 to 3) | 0.52 (0.27 to 0.83) | 1.35 (1.26 to 1.45) | 1 (1 to 2) | 0.41 (0.18 to 0.74) | 1.59 (1.54 to 1.64) |
| Bolivia (Plurinational State of) | 40 (23 to 66) | 0.48 (0.27 to 0.8) | 0.89 (0.7 to 1.09) | 9 (5 to 15) | 0.24 (0.13 to 0.4) | 1.63 (1.27 to 2) | 31 (16 to 53) | 0.7 (0.36 to 1.2) | 0.76 (0.57 to 0.96) |
| Bosnia and Herzegovina | 45 (28 to 68) | 0.7 (0.43 to 1.04) | 0.4 (0.13 to 0.66) | 19 (11 to 31) | 0.69 (0.42 to 1.12) | 0.4 (0.09 to 0.71) | 26 (16 to 39) | 0.71 (0.42 to 1.03) | 0.35 (0.12 to 0.59) |
| Botswana | 11 (6 to 23) | 0.83 (0.44 to 1.57) | 0.69 (0.27 to 1.11) | 6 (2 to 14) | 0.99 (0.39 to 2.32) | 1.75 (1.34 to 2.15) | 6 (3 to 10) | 0.72 (0.42 to 1.24) | -0.11 (-1.12 to 0.9) |
| Brazil | 409 (345 to 477) | 0.17 (0.14 to 0.19) | 0.71 (0.47 to 0.94) | 179 (152 to 210) | 0.16 (0.14 to 0.19) | 1.38 (0.96 to 1.79) | 230 (192 to 267) | 0.17 (0.14 to 0.19) | 0.33 (-0.26 to 0.92) |
| Brunei Darussalam | 2 (1 to 3) | 0.71 (0.44 to 1.08) | -0.73 (-1.24 to -0.21) | 1 (1 to 2) | 0.63 (0.38 to 0.97) | -1.04 (-2.08 to 0.01) | 1 (1 to 2) | 0.76 (0.45 to 1.23) | -0.63 (-1.03 to -0.22) |
| Bulgaria | 46 (28 to | 0.32 (0.21 | -2.84 | 19 (12 to | 0.32 (0.2 to | -2.14 | 27 (16 to | 0.32 (0.2 to | -3.39 |

| Location | Both Cases (95% UI) 2021 | Both ASDR (95% UI) 2021 | Both 1990-2021 AAPC (95% UI) | Men Cases (95% UI) 2021 | Men ASDR (95% UI) 2021 | Men 1990-2021AAPC (95% UI) | Women Cases (95% UI) 2021 | Women ASDR (95% UI) 2021 | Women 1990-2021 AAPC (95% UI) |
|---|---|---|---|---|---|---|---|---|---|
| | 70) | to 0.48) | (-3.7 to -1.97) | 30) | 0.51) | (-2.84 to -1.42) | 41) | 0.47) | (-4.61 to -2.16) |
| Burkina Faso | 177 (85 to 344) | 2 (0.97 to 3.97) | -0.48 (-0.81 to -0.15) | 80 (33 to 142) | 1.9 (0.77 to 3.35) | -1.11 (-1.39 to -0.82) | 97 (43 to 212) | 2.09 (0.91 to 4.7) | 0.09 (-0.27 to 0.46) |
| Burundi | 18 (11 to 30) | 0.39 (0.23 to 0.64) | -1.21 (-1.37 to -1.05) | 4 (1 to 12) | 0.18 (0.06 to 0.5) | -1.45 (-1.6 to -1.29) | 14 (8 to 22) | 0.61 (0.35 to 0.99) | -0.9 (-1.06 to -0.75) |
| Cabo Verde | 9 (5 to 13) | 2.11 (1.23 to 3.23) | 1.56 (1.28 to 1.84) | 3 (2 to 5) | 1.92 (1 to 3.11) | 2.73 (1.96 to 3.5) | 5 (3 to 9) | 2.22 (1.17 to 3.56) | 0.94 (0.78 to 1.1) |
| Cambodia | 75 (37 to 137) | 0.66 (0.33 to 1.28) | -0.38 (-0.51 to -0.26) | 25 (10 to 62) | 0.52 (0.22 to 1.31) | -0.34 (-0.44 to -0.25) | 50 (23 to 91) | 0.75 (0.34 to 1.35) | -0.47 (-0.57 to -0.37) |
| Cameroon | 214 (109 to 340) | 1.83 (0.91 to 2.94) | -0.73 (-0.92 to -0.54) | 90 (45 to 177) | 1.57 (0.78 to 3.06) | -0.76 (-0.99 to -0.52) | 123 (54 to 210) | 2.06 (0.91 to 3.62) | -0.73 (-0.85 to -0.61) |
| Canada | 502 (348 to 700) | 0.66 (0.47 to 0.91) | 3.19 (2.62 to 3.75) | 232 (149 to 332) | 0.68 (0.44 to 0.96) | 3.21 (2.86 to 3.55) | 270 (183 to 376) | 0.65 (0.44 to 0.89) | 3.42 (3.21 to 3.63) |

| Location | Both: Cases (95% UI) 2021 | Both: ASDR (95% UI) 2021 | Both: 1990-2021 AAPC (95% UI) | Men: Cases (95% UI) 2021 | Men: ASDR (95% UI) 2021 | Men: 1990-2021AAPC (95% UI) | Women: Cases (95% UI) 2021 | Women: ASDR (95% UI) 2021 | Women: 1990-2021 AAPC (95% UI) |
|---|---|---|---|---|---|---|---|---|---|
| Central African Republic | 12 (5 to 26) | 0.57 (0.21 to 1.34) | -1.29 (-1.5 to -1.08) | 6 (2 to 12) | 0.61 (0.23 to 1.29) | -1.65 (-1.93 to -1.37) | 6 (2 to 16) | 0.52 (0.15 to 1.49) | -0.91 (-1.14 to -0.68) |
| Chad | 76 (41 to 144) | 1.42 (0.74 to 2.73) | -0.08 (-0.29 to 0.14) | 36 (20 to 64) | 1.24 (0.69 to 2.22) | -0.91 (-1.41 to -0.41) | 40 (19 to 87) | 1.65 (0.74 to 3.62) | 0.76 (0.6 to 0.92) |
| Chile | 96 (62 to 137) | 0.37 (0.24 to 0.52) | 2.98 (2.46 to 3.5) | 43 (27 to 64) | 0.38 (0.24 to 0.56) | 3.19 (2.22 to 4.16) | 53 (34 to 75) | 0.36 (0.23 to 0.51) | 2.91 (2.44 to 3.38) |
| China | 10409 (8036 to 13180) | 0.51 (0.39 to 0.64) | 0.12 (-0.36 to 0.6) | 5514 (3895 to 7542) | 0.58 (0.41 to 0.78) | 0.34 (-0.09 to 0.78) | 4895 (3669 to 6558) | 0.45 (0.34 to 0.6) | -0.08 (-0.53 to 0.38) |
| Colombia | 163 (107 to 233) | 0.3 (0.2 to 0.43) | 0.16 (-0.85 to 1.17) | 62 (39 to 92) | 0.26 (0.16 to 0.38) | 1 (-0.03 to 2.03) | 101 (66 to 145) | 0.33 (0.21 to 0.47) | -0.54 (-2.04 to 0.97) |
| Comoros | 4 (2 to 6) | 0.85 (0.49 to 1.29) | -0.24 (-0.39 to -0.08) | 1 (0 to 2) | 0.47 (0.21 to 0.9) | -0.46 (-0.95 to 0.03) | 3 (2 to 5) | 1.17 (0.66 to 1.81) | -0.25 (-0.39 to -0.1) |

| Location | Both | | | Men | | | Women | | |
|---|---|---|---|---|---|---|---|---|---|
| | Cases (95% UI) 2021 | ASDR (95% UI) 2021 | 1990-2021 AAPC (95% UI) | Cases (95% UI) 2021 | ASDR (95% UI) 2021 | 1990-2021AAPC (95% UI) | Cases (95% UI) 2021 | ASDR (95% UI) 2021 | 1990-2021 AAPC (95% UI) |
| Congo | 19 (8 to 41) | 0.74 (0.3 to 1.72) | -1.17 (-1.37 to -0.96) | 7 (3 to 13) | 0.55 (0.28 to 1.05) | -1.89 (-2.11 to -1.66) | 12 (4 to 30) | 0.91 (0.3 to 2.3) | -0.7 (-1.04 to -0.35) |
| Cook Islands | 0 (0 to 1) | 1.33 (0.87 to 1.94) | 0.36 (0.13 to 0.6) | 0 (0 to 0) | 1.55 (0.96 to 2.29) | 1.19 (0.77 to 1.61) | 0 (0 to 0) | 1.13 (0.7 to 1.71) | -0.35 (-0.67 to -0.04) |
| Costa Rica | 38 (25 to 54) | 0.7 (0.46 to 0.99) | 1.58 (0.89 to 2.27) | 18 (12 to 26) | 0.74 (0.48 to 1.08) | 2.06 (0.66 to 3.48) | 20 (13 to 29) | 0.67 (0.44 to 0.96) | 1.39 (0.78 to 2.01) |
| Croatia | 33 (21 to 49) | 0.35 (0.22 to 0.5) | -0.84 (-0.97 to -0.7) | 15 (9 to 23) | 0.38 (0.23 to 0.57) | -1.68 (-1.97 to -1.38) | 18 (12 to 27) | 0.31 (0.2 to 0.45) | -0.12 (-0.42 to 0.18) |
| Cuba | 39 (26 to 53) | 0.19 (0.13 to 0.26) | 0.27 (-0.72 to 1.27) | 16 (10 to 23) | 0.17 (0.11 to 0.25) | 1.65 (0.43 to 2.89) | 23 (15 to 32) | 0.21 (0.14 to 0.3) | -0.52 (-1.82 to 0.8) |
| Cyprus | 5 (3 to 8) | 0.24 (0.15 to 0.37) | -0.56 (-1.19 to 0.08) | 2 (1 to 4) | 0.3 (0.17 to 0.47) | 0.11 (-1.08 to 1.3) | 2 (1 to 4) | 0.21 (0.13 to 0.33) | -0.94 (-2 to 0.12) |
| Czechia | 52 (33 to | 0.23 (0.15 | -0.01 | 25 (16 to | 0.26 (0.17 to | 0.36 | 27 (17 to | 0.2 (0.13 to | -0.15 |

| | Both | | | Men | | | Women | | |
|---|---|---|---|---|---|---|---|---|---|
| Location | Cases (95% UI) 2021 | ASDR (95% UI) 2021 | 1990-2021 AAPC (95% UI) | Cases (95% UI) 2021 | ASDR (95% UI) 2021 | 1990-2021AAPC (95% UI) | Cases (95% UI) 2021 | ASDR (95% UI) 2021 | 1990-2021 AAPC (95% UI) |
| | 79) | to 0.34) | (-0.26 to 0.23) | 38) | 0.38) | (0.04 to 0.67) | 42) | 0.3) | (-0.47 to 0.17) |
| Côte d'Ivoire | 52 (26 to 83) | 0.5 (0.26 to 0.81) | -1.53 (-2.38 to -0.67) | 22 (11 to 38) | 0.4 (0.19 to 0.69) | -0.91 (-1.79 to -0.03) | 30 (13 to 52) | 0.61 (0.26 to 1.07) | -2 (-3.01 to -0.98) |
| Democratic People's Republic of Korea | 149 (86 to 241) | 0.46 (0.26 to 0.72) | -0.73 (-0.83 to -0.62) | 59 (31 to 105) | 0.43 (0.23 to 0.72) | -1.23 (-1.32 to -1.14) | 89 (52 to 146) | 0.47 (0.28 to 0.74) | -0.37 (-0.57 to -0.17) |
| Democratic Republic of the Congo | 113 (48 to 275) | 0.34 (0.13 to 0.87) | -0.46 (-0.55 to -0.36) | 34 (12 to 89) | 0.22 (0.07 to 0.62) | -0.83 (-1.12 to -0.53) | 79 (32 to 194) | 0.42 (0.17 to 1.11) | -0.44 (-0.52 to -0.36) |
| Denmark | 25 (15 to 36) | 0.19 (0.12 to 0.28) | 2.57 (1.65 to 3.51) | 13 (8 to 19) | 0.22 (0.14 to 0.33) | 3.1 (2.08 to 4.12) | 12 (7 to 17) | 0.17 (0.11 to 0.24) | 2.14 (1.26 to 3.03) |
| Djibouti | 4 (2 to 7) | 0.71 (0.43 to 1.13) | 1.07 (0.93 to 1.21) | 2 (1 to 3) | 0.5 (0.25 to 0.91) | 1.54 (1.37 to 1.71) | 2 (1 to 4) | 0.94 (0.54 to 1.48) | 0.96 (0.81 to 1.11) |
| Dominica | 0 (0 to 1) | 0.46 (0.28 to 0.69) | 1.89 (1.38 to 2.4) | 0 (0 to 0) | 0.56 (0.34 to 0.87) | 2.11 (1.5 to 2.72) | 0 (0 to 0) | 0.4 (0.24 to 0.6) | 1.66 (1.18 to 2.14) |

| Location | Both | | | Men | | | Women | | |
|---|---|---|---|---|---|---|---|---|---|
| | Cases (95% UI) 2021 | ASDR (95% UI) 2021 | 1990-2021 AAPC (95% UI) | Cases (95% UI) 2021 | ASDR (95% UI) 2021 | 1990-2021AAPC (95% UI) | Cases (95% UI) 2021 | ASDR (95% UI) 2021 | 1990-2021 AAPC (95% UI) |
| Dominican Republic | 19 (12 to 29) | 0.19 (0.12 to 0.29) | 1.63 (1.28 to 1.98) | 9 (5 to 14) | 0.18 (0.11 to 0.29) | 2.4 (1.48 to 3.32) | 10 (6 to 16) | 0.2 (0.12 to 0.3) | 1.12 (0.88 to 1.36) |
| Ecuador | 73 (50 to 104) | 0.46 (0.32 to 0.65) | -0.37 (-1.54 to 0.8) | 19 (12 to 28) | 0.26 (0.16 to 0.38) | -0.16 (-1.55 to 1.25) | 54 (37 to 77) | 0.63 (0.43 to 0.9) | -0.49 (-1.75 to 0.78) |
| Egypt | 1427 (889 to 2052) | 2.42 (1.5 to 3.54) | 2.11 (1.37 to 2.86) | 777 (472 to 1181) | 2.35 (1.43 to 3.51) | 3.13 (2.59 to 3.66) | 651 (413 to 955) | 3.03 (1.93 to 4.44) | 2.07 (1.36 to 2.78) |
| El Salvador | 13 (8 to 18) | 0.2 (0.13 to 0.29) | 0.34 (-0.25 to 0.93) | 5 (3 to 7) | 0.18 (0.11 to 0.27) | 0.8 (-0.2 to 1.82) | 8 (5 to 12) | 0.22 (0.14 to 0.33) | 0.06 (-0.57 to 0.69) |
| Equatorial Guinea | 3 (2 to 5) | 0.69 (0.39 to 1.15) | 2.38 (2.12 to 2.64) | 1 (1 to 3) | 0.69 (0.37 to 1.38) | 4.47 (4.07 to 4.86) | 2 (1 to 3) | 0.69 (0.37 to 1.15) | 1.4 (1.09 to 1.71) |
| Eritrea | 15 (8 to 24) | 0.58 (0.33 to 0.95) | 0.17 (-0.04 to 0.39) | 3 (1 to 7) | 0.26 (0.11 to 0.63) | -0.47 (-0.76 to -0.17) | 11 (7 to 19) | 0.77 (0.43 to 1.27) | 0.35 (0.16 to 0.55) |
| Estonia | 11 (7 to 16) | 0.37 (0.25 | 1.29 | 4 (3 to 6) | 0.44 (0.3 to | 1.72 | 6 (4 to 9) | 0.33 (0.21 to | 1.06 |

| Location | Both | | | Men | | | Women | | |
|---|---|---|---|---|---|---|---|---|---|
| | Cases (95% UI) 2021 | ASDR (95% UI) 2021 | 1990-2021 AAPC (95% UI) | Cases (95% UI) 2021 | ASDR (95% UI) 2021 | 1990-2021AAPC (95% UI) | Cases (95% UI) 2021 | ASDR (95% UI) 2021 | 1990-2021 AAPC (95% UI) |
| | | to 0.54) | (0.26 to 2.32) | | 0.65) | (0.37 to 3.09) | | 0.46) | (0.51 to 1.61) |
| Eswatini | 15 (6 to 32) | 2.81 (1.23 to 5.97) | 2.56 (2.1 to 3.02) | 10 (3 to 26) | 5.01 (1.46 to 12.83) | 4.52 (4.01 to 5.03) | 5 (3 to 8) | 1.56 (0.91 to 2.75) | 0.59 (0.18 to 1) |
| Ethiopia | 145 (95 to 231) | 0.35 (0.23 to 0.55) | -0.86 (-1.06 to -0.66) | 33 (19 to 58) | 0.15 (0.09 to 0.27) | -0.8 (-1.01 to -0.59) | 112 (71 to 185) | 0.55 (0.34 to 0.9) | -0.85 (-0.99 to -0.72) |
| Fiji | 4 (3 to 7) | 0.58 (0.36 to 0.88) | 1.19 (0.73 to 1.66) | 2 (1 to 3) | 0.64 (0.37 to 1.03) | 1.39 (0.7 to 2.08) | 2 (1 to 3) | 0.55 (0.34 to 0.84) | 1 (0.83 to 1.18) |
| Finland | 43 (28 to 63) | 0.31 (0.2 to 0.44) | 1.22 (0.53 to 1.92) | 21 (13 to 31) | 0.34 (0.23 to 0.5) | 1.52 (0.76 to 2.29) | 23 (14 to 34) | 0.28 (0.18 to 0.42) | 0.88 (0.49 to 1.28) |
| France | 639 (406 to 924) | 0.43 (0.28 to 0.61) | 1.36 (1.09 to 1.63) | 347 (221 to 516) | 0.56 (0.36 to 0.83) | 0.87 (0.6 to 1.15) | 293 (171 to 432) | 0.33 (0.2 to 0.46) | 2.04 (1.71 to 2.37) |
| Gabon | 11 (6 to 18) | 1.08 (0.6 to 1.82) | 0.91 (0.7 to 1.12) | 5 (3 to 10) | 1.15 (0.59 to 2.15) | 1.35 (1.2 to 1.49) | 5 (2 to 10) | 1 (0.45 to 1.88) | 0.56 (0.36 to 0.77) |

| Location | Both | | | Men | | | Women | | |
|---|---|---|---|---|---|---|---|---|---|
| | Cases (95% UI) 2021 | ASDR (95% UI) 2021 | 1990-2021 AAPC (95% UI) | Cases (95% UI) 2021 | ASDR (95% UI) 2021 | 1990-2021AAPC (95% UI) | Cases (95% UI) 2021 | ASDR (95% UI) 2021 | 1990-2021 AAPC (95% UI) |
| Gambia | 38 (22 to 60) | 3.99 (2.18 to 6.4) | 1.25 (0.8 to 1.71) | 21 (11 to 36) | 4.52 (2.46 to 8.03) | 1.41 (1.01 to 1.82) | 18 (8 to 28) | 3.54 (1.67 to 5.84) | 1.17 (0.55 to 1.79) |
| Georgia | 21 (14 to 31) | 0.35 (0.23 to 0.51) | -0.24 (-1.95 to 1.51) | 11 (7 to 16) | 0.45 (0.3 to 0.66) | 0.13 (-2.51 to 2.83) | 10 (7 to 15) | 0.28 (0.19 to 0.4) | -0.82 (-2.59 to 0.98) |
| Germany | 676 (446 to 1019) | 0.33 (0.22 to 0.47) | 1.75 (1.54 to 1.97) | 337 (210 to 503) | 0.37 (0.23 to 0.54) | 2.08 (1.82 to 2.33) | 339 (218 to 507) | 0.29 (0.2 to 0.42) | 1.52 (1.2 to 1.84) |
| Ghana | 174 (97 to 273) | 1.16 (0.63 to 1.8) | -0.1 (-0.29 to 0.09) | 84 (50 to 145) | 1.32 (0.77 to 2.31) | 0.68 (0.46 to 0.91) | 91 (41 to 156) | 1.06 (0.47 to 1.85) | -0.7 (-0.88 to -0.52) |
| Greece | 81 (55 to 117) | 0.3 (0.21 to 0.43) | 2.31 (1.83 to 2.79) | 35 (23 to 52) | 0.3 (0.2 to 0.44) | 3.54 (2.92 to 4.16) | 47 (29 to 67) | 0.31 (0.21 to 0.43) | 1.67 (1.16 to 2.18) |
| Greenland | 0 (0 to 1) | 0.81 (0.48 to 1.21) | 0.72 (0.25 to 1.2) | 0 (0 to 0) | 0.78 (0.46 to 1.22) | 0.17 (-0.03 to 0.38) | 0 (0 to 0) | 0.84 (0.49 to 1.28) | 1.22 (0.53 to 1.91) |
| Grenada | 0 (0 to 1) | 0.42 (0.29 | 2.37 | 0 (0 to 0) | 1.11 (0.68 to | 6.35 | 0 (0 to 0) | 0.38 (0.26 to | 1.72 |

| Location | Both: Cases (95% UI) 2021 | Both: ASDR (95% UI) 2021 | Both: 1990-2021 AAPC (95% UI) | Men: Cases (95% UI) 2021 | Men: ASDR (95% UI) 2021 | Men: 1990-2021AAPC (95% UI) | Women: Cases (95% UI) 2021 | Women: ASDR (95% UI) 2021 | Women: 1990-2021 AAPC (95% UI) |
|---|---|---|---|---|---|---|---|---|---|
| | | to 0.6) | (0.92 to 3.85) | | 1.68) | (5.19 to 7.53) | | 0.53) | (0.23 to 3.23) |
| Guam | 1 (1 to 2) | 0.61 (0.42 to 0.87) | 1.98 (1.38 to 2.58) | 1 (1 to 1) | 0.85 (0.57 to 1.22) | 2.67 (2.31 to 3.03) | 0 (0 to 1) | 0.4 (0.26 to 0.6) | 0.92 (-0.1 to 1.94) |
| Guatemala | 60 (41 to 85) | 0.56 (0.37 to 0.8) | -0.67 (-2.68 to 1.39) | 24 (16 to 35) | 0.48 (0.32 to 0.71) | -0.66 (-2.79 to 1.52) | 36 (24 to 50) | 0.64 (0.42 to 0.9) | -0.39 (-1.98 to 1.23) |
| Guinea | 133 (76 to 198) | 2.42 (1.36 to 3.61) | -0.19 (-0.33 to -0.05) | 53 (29 to 92) | 1.91 (1.05 to 3.41) | -0.35 (-0.66 to -0.03) | 80 (41 to 129) | 2.93 (1.51 to 4.84) | -0.11 (-0.21 to -0.02) |
| Guinea-Bissau | 15 (10 to 23) | 2.31 (1.45 to 3.51) | -0.81 (-0.99 to -0.63) | 7 (4 to 12) | 2.43 (1.29 to 3.97) | -1.37 (-1.95 to -0.78) | 8 (5 to 12) | 2.23 (1.35 to 3.4) | -0.2 (-0.34 to -0.07) |
| Guyana | 2 (1 to 2) | 0.25 (0.16 to 0.36) | 0.33 (-0.69 to 1.35) | 1 (0 to 1) | 0.21 (0.13 to 0.32) | 1.03 (0.04 to 2.02) | 1 (1 to 1) | 0.27 (0.18 to 0.39) | -0.11 (-0.97 to 0.75) |
| Haiti | 9 (4 to 18) | 0.14 (0.06 to 0.29) | -0.33 (-0.4 to -0.25) | 2 (1 to 6) | 0.07 (0.03 to 0.19) | -0.13 (-0.22 to -0.04) | 7 (3 to 13) | 0.2 (0.09 to 0.42) | -0.33 (-0.41 to -0.24) |

| | Both | | | Men | | | Women | | |
|---|---|---|---|---|---|---|---|---|---|
| Location | Cases (95% UI) 2021 | ASDR (95% UI) 2021 | 1990-2021 AAPC (95% UI) | Cases (95% UI) 2021 | ASDR (95% UI) 2021 | 1990-2021AAPC (95% UI) | Cases (95% UI) 2021 | ASDR (95% UI) 2021 | 1990-2021 AAPC (95% UI) |
| Honduras | 26 (15 to 40) | 0.43 (0.25 to 0.69) | 2.61 (2.12 to 3.1) | 8 (4 to 13) | 0.28 (0.15 to 0.47) | 2.5 (2.29 to 2.71) | 18 (10 to 29) | 0.56 (0.32 to 0.92) | 2.5 (1.92 to 3.09) |
| Hungary | 35 (23 to 52) | 0.17 (0.11 to 0.26) | -1.38 (-2.48 to -0.27) | 16 (10 to 24) | 0.2 (0.13 to 0.29) | -0.52 (-1.72 to 0.69) | 20 (12 to 29) | 0.16 (0.1 to 0.23) | -1.91 (-2.78 to -1.04) |
| Iceland | 2 (1 to 3) | 0.33 (0.22 to 0.47) | 1.9 (1.4 to 2.4) | 1 (1 to 2) | 0.39 (0.26 to 0.57) | 2.22 (1.69 to 2.75) | 1 (1 to 1) | 0.26 (0.16 to 0.38) | 1.68 (1.08 to 2.29) |
| India | 4735 (3969 to 5648) | 0.41 (0.34 to 0.49) | 1.52 (1.4 to 1.65) | 2404 (1947 to 2948) | 0.43 (0.35 to 0.53) | 1.42 (1.3 to 1.54) | 2331 (1908 to 2805) | 0.39 (0.32 to 0.47) | 1.7 (1.55 to 1.85) |
| Indonesia | 1085 (576 to 1699) | 0.48 (0.25 to 0.75) | 1.31 (1.24 to 1.37) | 603 (378 to 1053) | 0.53 (0.33 to 0.92) | 1.61 (1.52 to 1.7) | 481 (149 to 804) | 0.42 (0.13 to 0.71) | 0.98 (0.9 to 1.07) |
| Iran (Islamic Republic of) | 338 (277 to 401) | 0.49 (0.4 to 0.58) | 1.83 (1.31 to 2.35) | 148 (123 to 178) | 0.42 (0.35 to 0.51) | 2.42 (1.29 to 3.57) | 189 (148 to 234) | 0.55 (0.43 to 0.68) | 1.58 (1.21 to 1.96) |
| Iraq | 136 (82 to | 0.64 (0.4 to | 1.32 | 67 (39 to | 0.67 (0.39 to | 1.62 | 69 (44 to | 0.62 (0.4 to | 1.04 |

| Location | Both: Cases (95% UI) 2021 | Both: ASDR (95% UI) 2021 | Both: 1990-2021 AAPC (95% UI) | Men: Cases (95% UI) 2021 | Men: ASDR (95% UI) 2021 | Men: 1990-2021AAPC (95% UI) | Women: Cases (95% UI) 2021 | Women: ASDR (95% UI) 2021 | Women: 1990-2021 AAPC (95% UI) |
|---|---|---|---|---|---|---|---|---|---|
| | 199) | 0.95) | (0.97 to 1.67) | 101) | 1) | (1.3 to 1.94) | 103) | 0.94) | (0.62 to 1.45) |
| Ireland | 22 (14 to 32) | 0.27 (0.18 to 0.38) | 1.98 (1.42 to 2.55) | 10 (6 to 14) | 0.25 (0.16 to 0.36) | 2.42 (2.02 to 2.81) | 12 (8 to 18) | 0.28 (0.18 to 0.4) | 1.75 (1.01 to 2.5) |
| Israel | 27 (18 to 39) | 0.21 (0.14 to 0.3) | 0.23 (0 to 0.47) | 13 (8 to 19) | 0.23 (0.15 to 0.33) | 0.8 (0.61 to 1) | 14 (9 to 21) | 0.2 (0.13 to 0.29) | -0.17 (-0.78 to 0.45) |
| Italy | 315 (248 to 386) | 0.2 (0.17 to 0.24) | -1.28 (-2.8 to 0.25) | 165 (134 to 200) | 0.25 (0.21 to 0.3) | -1.2 (-2.9 to 0.53) | 150 (113 to 188) | 0.16 (0.12 to 0.19) | -1.44 (-2.79 to -0.07) |
| Jamaica | 6 (4 to 9) | 0.2 (0.13 to 0.3) | 1.9 (0.59 to 3.23) | 3 (2 to 4) | 0.18 (0.11 to 0.28) | 2.17 (-0.05 to 4.44) | 4 (2 to 5) | 0.22 (0.14 to 0.32) | 1.76 (0.86 to 2.66) |
| Japan | 1470 (1105 to 1848) | 0.33 (0.26 to 0.4) | -1.28 (-1.51 to -1.04) | 706 (566 to 856) | 0.4 (0.33 to 0.47) | -1.49 (-1.64 to -1.34) | 764 (534 to 994) | 0.26 (0.2 to 0.33) | -0.98 (-1.41 to -0.56) |
| Jordan | 16 (9 to 23) | 0.23 (0.14 to 0.36) | 0.11 (-0.32 to 0.54) | 8 (5 to 12) | 0.23 (0.13 to 0.35) | 0.36 (0.05 to 0.67) | 8 (5 to 12) | 0.25 (0.15 to 0.38) | -0.08 (-0.63 to 0.47) |

| Location | Both | | | Men | | | Women | | |
|---|---|---|---|---|---|---|---|---|---|
| | Cases (95% UI) 2021 | ASDR (95% UI) 2021 | 1990-2021 AAPC (95% UI) | Cases (95% UI) 2021 | ASDR (95% UI) 2021 | 1990-2021AAPC (95% UI) | Cases (95% UI) 2021 | ASDR (95% UI) 2021 | 1990-2021 AAPC (95% UI) |
| Kazakhstan | 82 (54 to 117) | 0.5 (0.32 to 0.7) | -1.35 (-2.02 to -0.68) | 33 (21 to 49) | 0.51 (0.34 to 0.74) | -1.99 (-2.68 to -1.3) | 49 (31 to 70) | 0.48 (0.32 to 0.7) | -0.74 (-1.56 to 0.09) |
| Kenya | 170 (124 to 228) | 0.77 (0.57 to 1.02) | 2.07 (1.86 to 2.27) | 63 (44 to 92) | 0.59 (0.41 to 0.84) | 2.87 (2.64 to 3.1) | 107 (74 to 153) | 0.9 (0.63 to 1.26) | 1.5 (1.23 to 1.77) |
| Kiribati | 1 (0 to 1) | 0.72 (0.44 to 1.02) | 0.26 (0.11 to 0.41) | 0 (0 to 0) | 0.43 (0.24 to 0.71) | -0.12 (-0.68 to 0.45) | 0 (0 to 1) | 0.92 (0.55 to 1.37) | 0.36 (0.22 to 0.49) |
| Kuwait | 5 (3 to 7) | 0.21 (0.14 to 0.3) | -2.78 (-7.16 to 1.81) | 3 (2 to 4) | 0.21 (0.13 to 0.31) | -2.92 (-6.35 to 0.63) | 2 (1 to 3) | 0.21 (0.14 to 0.3) | -2.54 (-5.84 to 0.88) |
| Kyrgyzstan | 13 (8 to 19) | 0.29 (0.18 to 0.43) | -1.25 (-3.18 to 0.71) | 6 (4 to 10) | 0.32 (0.2 to 0.49) | -1.15 (-3.01 to 0.75) | 7 (4 to 10) | 0.26 (0.16 to 0.4) | -1.55 (-3.35 to 0.29) |
| Lao People's Democratic Republic | 26 (15 to 41) | 0.59 (0.33 to 0.94) | -0.9 (-0.99 to -0.81) | 14 (7 to 22) | 0.64 (0.34 to 1.03) | -0.98 (-1.11 to -0.86) | 12 (7 to 19) | 0.55 (0.32 to 0.86) | -0.79 (-0.94 to -0.64) |
| Latvia | 12 (8 to 17) | 0.29 (0.2 to | 1.19 | 5 (3 to 7) | 0.33 (0.22 to | 0.95 | 7 (5 to 10) | 0.26 (0.18 to | 1.1 |

| Location | Both | | | Men | | | Women | | |
|---|---|---|---|---|---|---|---|---|---|
| | Cases (95% UI) 2021 | ASDR (95% UI) 2021 | 1990-2021 AAPC (95% UI) | Cases (95% UI) 2021 | ASDR (95% UI) 2021 | 1990-2021AAPC (95% UI) | Cases (95% UI) 2021 | ASDR (95% UI) 2021 | 1990-2021 AAPC (95% UI) |
| | | 0.39) | (-0.28 to 2.67) | | 0.46) | (-0.56 to 2.48) | | 0.36) | (0.25 to 1.95) |
| Lebanon | 14 (9 to 21) | 0.23 (0.15 to 0.35) | -0.34 (-0.57 to -0.12) | 6 (4 to 9) | 0.22 (0.14 to 0.34) | 0.17 (-0.1 to 0.44) | 8 (5 to 13) | 0.24 (0.15 to 0.39) | -0.68 (-0.85 to -0.52) |
| Lesotho | 21 (9 to 47) | 2.1 (0.91 to 4.47) | 3.58 (3.23 to 3.93) | 12 (3 to 35) | 3.2 (0.83 to 10.16) | 4.77 (4.49 to 5.05) | 9 (6 to 15) | 1.54 (0.93 to 2.47) | 2.6 (2.23 to 2.97) |
| Liberia | 47 (30 to 70) | 2.38 (1.5 to 3.62) | -0.09 (-0.36 to 0.19) | 24 (13 to 39) | 2.31 (1.27 to 3.85) | -0.37 (-0.62 to -0.11) | 23 (14 to 34) | 2.45 (1.52 to 3.68) | 0.19 (-0.17 to 0.55) |
| Libya | 49 (31 to 74) | 1 (0.64 to 1.52) | 1.77 (1.5 to 2.04) | 22 (14 to 33) | 0.87 (0.54 to 1.32) | 2.29 (1.87 to 2.71) | 28 (17 to 42) | 1.13 (0.72 to 1.75) | 1.36 (0.93 to 1.79) |
| Lithuania | 17 (12 to 24) | 0.29 (0.2 to 0.41) | 1.63 (0.74 to 2.53) | 8 (5 to 11) | 0.37 (0.25 to 0.52) | 2.53 (1.92 to 3.13) | 10 (6 to 14) | 0.24 (0.16 to 0.35) | 0.93 (0.02 to 1.84) |
| Luxembourg | 3 (2 to 5) | 0.29 (0.18 to 0.41) | 0.9 (0.51 to 1.29) | 1 (1 to 2) | 0.3 (0.19 to 0.44) | 1.13 (0.58 to 1.68) | 2 (1 to 2) | 0.27 (0.17 to 0.39) | 0.58 (0.26 to 0.9) |

| Location | Both | | | Men | | | Women | | |
|---|---|---|---|---|---|---|---|---|---|
| | Cases (95% UI) 2021 | ASDR (95% UI) 2021 | 1990-2021 AAPC (95% UI) | Cases (95% UI) 2021 | ASDR (95% UI) 2021 | 1990-2021AAPC (95% UI) | Cases (95% UI) 2021 | ASDR (95% UI) 2021 | 1990-2021 AAPC (95% UI) |
| Madagascar | 45 (26 to 73) | 0.42 (0.24 to 0.66) | 0 (-0.15 to 0.15) | 11 (5 to 25) | 0.22 (0.1 to 0.47) | -0.49 (-0.95 to -0.03) | 34 (20 to 53) | 0.6 (0.35 to 0.97) | 0.1 (-0.04 to 0.24) |
| Malawi | 51 (32 to 72) | 0.71 (0.44 to 1.03) | 1.22 (1.03 to 1.41) | 24 (14 to 37) | 0.72 (0.43 to 1.13) | 1.85 (1.48 to 2.23) | 27 (16 to 40) | 0.68 (0.41 to 1.05) | 0.73 (0.62 to 0.85) |
| Malaysia | 182 (121 to 269) | 0.68 (0.45 to 1.01) | 1.72 (1.02 to 2.43) | 105 (66 to 161) | 0.78 (0.5 to 1.2) | 2.07 (1.14 to 3) | 78 (51 to 116) | 0.58 (0.38 to 0.85) | 1.13 (0.73 to 1.54) |
| Maldives | 1 (1 to 2) | 0.5 (0.3 to 0.76) | -0.4 (-0.78 to -0.01) | 1 (1 to 2) | 0.75 (0.44 to 1.19) | 0.03 (-0.32 to 0.37) | 0 (0 to 0) | 0.21 (0.13 to 0.31) | -1.06 (-1.45 to -0.68) |
| Mali | 235 (151 to 364) | 2.87 (1.83 to 4.47) | 0.58 (0.41 to 0.76) | 138 (84 to 214) | 3.31 (2.03 to 5.08) | 0.69 (0.56 to 0.82) | 97 (59 to 152) | 2.41 (1.46 to 3.93) | 0.42 (0.21 to 0.63) |
| Malta | 2 (1 to 3) | 0.2 (0.13 to 0.3) | 1.21 (0.54 to 1.89) | 1 (1 to 2) | 0.26 (0.16 to 0.4) | 1.7 (1.34 to 2.06) | 1 (1 to 1) | 0.15 (0.1 to 0.22) | 0.42 (-0.37 to 1.22) |
| Marshall Islands | 0 (0 to 0) | 0.4 (0.22 to | 0.95 | 0 (0 to 0) | 0.33 (0.14 to | 1.08 | 0 (0 to 0) | 0.48 (0.29 to | 0.87 |

| Location | Both: Cases (95% UI) 2021 | Both: ASDR (95% UI) 2021 | Both: 1990-2021 AAPC (95% UI) | Men: Cases (95% UI) 2021 | Men: ASDR (95% UI) 2021 | Men: 1990-2021AAPC (95% UI) | Women: Cases (95% UI) 2021 | Women: ASDR (95% UI) 2021 | Women: 1990-2021 AAPC (95% UI) |
|---|---|---|---|---|---|---|---|---|---|
| | | 0.67) | (0.77 to 1.14) | | 0.7) | (0.55 to 1.61) | | 0.79) | (0.68 to 1.06) |
| Mauritania | 63 (35 to 99) | 3.13 (1.66 to 4.95) | -0.99 (-1.11 to -0.86) | 32 (12 to 57) | 3.16 (1.19 to 5.77) | -1.41 (-1.58 to -1.25) | 31 (18 to 50) | 3.1 (1.73 to 4.92) | -0.58 (-0.73 to -0.43) |
| Mauritius | 2 (1 to 2) | 0.09 (0.07 to 0.13) | -4.52 (-7.49 to -1.46) | 1 (1 to 1) | 0.09 (0.06 to 0.13) | -4.27 (-7.94 to -0.46) | 1 (1 to 1) | 0.09 (0.07 to 0.13) | -4.37 (-7.35 to -1.3) |
| Mexico | 389 (324 to 458) | 0.32 (0.27 to 0.38) | 1.92 (1.45 to 2.39) | 169 (134 to 209) | 0.3 (0.24 to 0.37) | 2.41 (1.76 to 3.07) | 220 (176 to 268) | 0.34 (0.27 to 0.41) | 1.76 (1.5 to 2.03) |
| Micronesia (Federated States of) | 0 (0 to 1) | 0.59 (0.34 to 0.93) | 0.79 (0.65 to 0.92) | 0 (0 to 0) | 0.56 (0.27 to 1.15) | 0.93 (0.67 to 1.19) | 0 (0 to 0) | 0.6 (0.29 to 0.97) | 0.66 (0.61 to 0.72) |
| Monaco | 1 (0 to 1) | 0.69 (0.41 to 1.05) | 2.69 (2.53 to 2.85) | 0 (0 to 1) | 0.82 (0.5 to 1.24) | 2.6 (2.38 to 2.82) | 0 (0 to 1) | 0.57 (0.31 to 0.91) | 2.67 (2.53 to 2.82) |
| Mongolia | 118 (73 to 183) | 5.87 (3.61 to 9.12) | 1.86 (1.39 to 2.33) | 43 (25 to 68) | 4.68 (2.74 to 7.38) | 0.87 (0.08 to 1.66) | 74 (47 to 116) | 6.57 (3.99 to 10.25) | 2.29 (1.73 to 2.85) |

| Location | Both | | | Men | | | Women | | |
|---|---|---|---|---|---|---|---|---|---|
| | Cases (95% UI) 2021 | ASDR (95% UI) 2021 | 1990-2021 AAPC (95% UI) | Cases (95% UI) 2021 | ASDR (95% UI) 2021 | 1990-2021AAPC (95% UI) | Cases (95% UI) 2021 | ASDR (95% UI) 2021 | 1990-2021 AAPC (95% UI) |
| Montenegro | 8 (5 to 11) | 0.81 (0.51 to 1.2) | 1.16 (0.87 to 1.47) | 3 (2 to 5) | 0.75 (0.45 to 1.18) | 1.33 (0.88 to 1.79) | 5 (3 to 7) | 0.84 (0.52 to 1.21) | 0.98 (0.77 to 1.19) |
| Morocco | 21 (13 to 31) | 0.06 (0.04 to 0.09) | 1.88 (1.75 to 2.01) | 12 (7 to 20) | 0.07 (0.05 to 0.12) | 2.09 (1.87 to 2.3) | 9 (5 to 14) | 0.05 (0.03 to 0.08) | 1.62 (1.53 to 1.71) |
| Mozambique | 304 (154 to 564) | 3.43 (1.74 to 6.44) | 1.13 (1.05 to 1.22) | 139 (51 to 341) | 3.4 (1.3 to 8.53) | 1.82 (1.56 to 2.08) | 165 (77 to 294) | 3.34 (1.49 to 6.19) | 0.54 (0.4 to 0.69) |
| Myanmar | 140 (62 to 261) | 0.3 (0.13 to 0.56) | 0.23 (0.17 to 0.3) | 57 (25 to 139) | 0.28 (0.13 to 0.68) | 0.61 (0.45 to 0.78) | 83 (22 to 168) | 0.31 (0.08 to 0.64) | 0 (-0.08 to 0.08) |
| Namibia | 5 (3 to 7) | 0.41 (0.26 to 0.62) | 1.17 (0.84 to 1.49) | 2 (1 to 3) | 0.42 (0.25 to 0.63) | 2.82 (2.45 to 3.19) | 3 (2 to 5) | 0.4 (0.24 to 0.63) | 0.26 (0.11 to 0.42) |
| Nauru | 0 (0 to 0) | 0.52 (0.32 to 0.82) | -0.58 (-0.97 to -0.19) | 0 (0 to 0) | 0.6 (0.33 to 1.02) | -0.04 (-0.43 to 0.35) | 0 (0 to 0) | 0.45 (0.25 to 0.75) | -1.27 (-1.52 to -1.02) |
| Nepal | 77 (44 to | 0.34 (0.19 | 2.64 | 41 (22 to | 0.38 (0.2 to | 3.08 | 36 (19 to | 0.3 (0.16 to | 2.12 |

| | Both | | | Men | | | Women | | |
|---|---|---|---|---|---|---|---|---|---|
| Location | Cases (95% UI) 2021 | ASDR (95% UI) 2021 | 1990-2021 AAPC (95% UI) | Cases (95% UI) 2021 | ASDR (95% UI) 2021 | 1990-2021AAPC (95% UI) | Cases (95% UI) 2021 | ASDR (95% UI) 2021 | 1990-2021 AAPC (95% UI) |
| | 123) | to 0.54) | (2.51 to 2.76) | 65) | 0.59) | (2.88 to 3.29) | 60) | 0.5) | (1.98 to 2.26) |
| Netherlands | 87 (58 to 126) | 0.24 (0.16 to 0.33) | 2.51 (1.73 to 3.3) | 41 (26 to 61) | 0.24 (0.16 to 0.36) | 2.54 (1.68 to 3.42) | 46 (30 to 66) | 0.23 (0.15 to 0.32) | 2.64 (1.65 to 3.63) |
| New Zealand | 37 (30 to 44) | 0.43 (0.36 to 0.52) | 3.08 (2.13 to 4.03) | 18 (14 to 22) | 0.46 (0.37 to 0.55) | 3.08 (2.67 to 3.5) | 19 (15 to 23) | 0.41 (0.33 to 0.49) | 2.87 (1.07 to 4.7) |
| Nicaragua | 16 (10 to 23) | 0.33 (0.21 to 0.49) | 0.63 (0.12 to 1.14) | 6 (4 to 10) | 0.31 (0.19 to 0.47) | 0.76 (0.38 to 1.14) | 9 (6 to 14) | 0.35 (0.22 to 0.53) | 0.47 (0.01 to 0.93) |
| Niger | 92 (52 to 165) | 1.23 (0.68 to 2.16) | -1.33 (-1.44 to -1.23) | 35 (20 to 62) | 0.96 (0.54 to 1.68) | -2.31 (-2.44 to -2.17) | 58 (30 to 105) | 1.48 (0.76 to 2.68) | -0.58 (-0.74 to -0.42) |
| Nigeria | 614 (415 to 902) | 0.75 (0.52 to 1.07) | 0.17 (0.06 to 0.29) | 128 (83 to 190) | 0.33 (0.22 to 0.48) | 0.07 (-0.31 to 0.45) | 487 (316 to 752) | 1.11 (0.74 to 1.66) | 0.17 (0.02 to 0.32) |
| Niue | 0 (0 to 0) | 0.63 (0.38 to 0.99) | 1.17 (1.05 to 1.29) | 0 (0 to 0) | 0.78 (0.42 to 1.39) | 1.67 (1.56 to 1.79) | 0 (0 to 0) | 0.51 (0.28 to 0.8) | 0.68 (0.55 to 0.81) |

| Location | Both | | | Men | | | Women | | |
|---|---|---|---|---|---|---|---|---|---|
| | Cases (95% UI) 2021 | ASDR (95% UI) 2021 | 1990-2021 AAPC (95% UI) | Cases (95% UI) 2021 | ASDR (95% UI) 2021 | 1990-2021AAPC (95% UI) | Cases (95% UI) 2021 | ASDR (95% UI) 2021 | 1990-2021 AAPC (95% UI) |
| North Macedonia | 28 (17 to 43) | 1.02 (0.62 to 1.53) | 0.45 (0.12 to 0.78) | 13 (8 to 21) | 1.04 (0.62 to 1.64) | 1.43 (1.08 to 1.78) | 15 (9 to 23) | 1.01 (0.62 to 1.48) | -0.35 (-0.72 to 0.01) |
| Northern Mariana Islands | 0 (0 to 1) | 0.91 (0.59 to 1.33) | 0.9 (0.21 to 1.6) | 0 (0 to 0) | 1.05 (0.67 to 1.56) | 1.87 (0.83 to 2.93) | 0 (0 to 0) | 0.85 (0.54 to 1.26) | -0.01 (-0.51 to 0.5) |
| Norway | 26 (21 to 30) | 0.25 (0.21 to 0.29) | 2.87 (1.59 to 4.18) | 11 (9 to 13) | 0.24 (0.2 to 0.28) | 2.66 (1.74 to 3.58) | 15 (12 to 18) | 0.26 (0.21 to 0.31) | 3.02 (1.1 to 4.97) |
| Oman | 9 (6 to 14) | 0.5 (0.32 to 0.74) | 2.2 (1.96 to 2.45) | 6 (4 to 10) | 0.64 (0.39 to 0.98) | 2.81 (2.46 to 3.17) | 3 (2 to 5) | 0.37 (0.22 to 0.58) | 1.35 (1.08 to 1.63) |
| Pakistan | 375 (278 to 501) | 0.3 (0.22 to 0.39) | 1.14 (0.98 to 1.29) | 140 (96 to 199) | 0.21 (0.14 to 0.29) | 1.23 (1.02 to 1.44) | 235 (165 to 328) | 0.39 (0.27 to 0.55) | 0.91 (0.73 to 1.1) |
| Palau | 0 (0 to 0) | 0.71 (0.43 to 1.11) | 0.7 (0.49 to 0.91) | 0 (0 to 0) | 1.35 (0.81 to 2.14) | 0.52 (0.3 to 0.74) | 0 (0 to 0) | 0 (0 to 0) | 0.9 (0.78 to 1.01) |
| Palestine | 16 (10 to | 0.72 (0.47 | -0.14 | 7 (5 to 11) | 0.78 (0.5 to | 0.19 | 8 (6 to 12) | 0.72 (0.46 to | -0.36 |

| Location | Both: Cases (95% UI) 2021 | Both: ASDR (95% UI) 2021 | Both: 1990-2021 AAPC (95% UI) | Men: Cases (95% UI) 2021 | Men: ASDR (95% UI) 2021 | Men: 1990-2021AAPC (95% UI) | Women: Cases (95% UI) 2021 | Women: ASDR (95% UI) 2021 | Women: 1990-2021 AAPC (95% UI) |
|---|---|---|---|---|---|---|---|---|---|
| | 24) | to 1.07) | (-0.38 to 0.12) | | 1.16) | (-0.01 to 0.39) | | 1.05) | (-0.6 to -0.12) |
| Panama | 16 (10 to 23) | 0.36 (0.23 to 0.52) | 0.24 (-0.95 to 1.43) | 6 (4 to 10) | 0.31 (0.19 to 0.47) | 0.3 (-0.1 to 0.71) | 9 (6 to 14) | 0.4 (0.26 to 0.59) | 0.08 (-0.25 to 0.41) |
| Papua New Guinea | 11 (5 to 27) | 0.22 (0.09 to 0.55) | -1.02 (-1.16 to -0.88) | 5 (2 to 16) | 0.2 (0.07 to 0.62) | -1.08 (-1.19 to -0.96) | 6 (3 to 12) | 0.24 (0.1 to 0.55) | -0.91 (-1.04 to -0.77) |
| Paraguay | 14 (9 to 21) | 0.25 (0.16 to 0.37) | 1.94 (1.53 to 2.35) | 6 (4 to 10) | 0.23 (0.13 to 0.37) | 2.93 (2.38 to 3.49) | 8 (5 to 12) | 0.27 (0.16 to 0.4) | 1.31 (0.82 to 1.8) |
| Peru | 100 (60 to 152) | 0.3 (0.18 to 0.46) | 1.57 (0.2 to 2.96) | 27 (16 to 43) | 0.17 (0.1 to 0.28) | 2.41 (1.29 to 3.54) | 73 (44 to 108) | 0.42 (0.25 to 0.62) | 1.4 (0.46 to 2.35) |
| Philippines | 444 (353 to 559) | 0.56 (0.45 to 0.7) | 0.32 (0.02 to 0.63) | 246 (181 to 326) | 0.67 (0.5 to 0.88) | 0.27 (-0.09 to 0.63) | 198 (150 to 259) | 0.47 (0.36 to 0.61) | 0.2 (-0.01 to 0.4) |
| Poland | 132 (110 to 156) | 0.18 (0.15 to 0.21) | 3.06 (2.14 to 3.98) | 50 (41 to 60) | 0.17 (0.14 to 0.2) | 4.99 (3.79 to 6.21) | 82 (66 to 97) | 0.18 (0.15 to 0.22) | 2.3 (1.27 to 3.34) |

| Location | Both | | | Men | | | Women | | |
|---|---|---|---|---|---|---|---|---|---|
| | Cases (95% UI) 2021 | ASDR (95% UI) 2021 | 1990-2021 AAPC (95% UI) | Cases (95% UI) 2021 | ASDR (95% UI) 2021 | 1990-2021AAPC (95% UI) | Cases (95% UI) 2021 | ASDR (95% UI) 2021 | 1990-2021 AAPC (95% UI) |
| Portugal | 79 (52 to 115) | 0.31 (0.21 to 0.45) | 2.92 (2.39 to 3.46) | 45 (29 to 67) | 0.43 (0.28 to 0.63) | 3.73 (3.19 to 4.28) | 34 (21 to 51) | 0.22 (0.14 to 0.32) | 1.8 (1.43 to 2.18) |
| Puerto Rico | 29 (19 to 41) | 0.39 (0.26 to 0.55) | 0.8 (-0.12 to 1.73) | 16 (10 to 23) | 0.51 (0.34 to 0.73) | 2.03 (1.12 to 2.94) | 13 (8 to 18) | 0.29 (0.2 to 0.41) | -0.5 (-1.63 to 0.64) |
| Qatar | 16 (10 to 24) | 2.68 (1.65 to 4.21) | 1.84 (0.35 to 3.35) | 11 (7 to 18) | 3.19 (1.96 to 5.29) | 2.46 (0.11 to 4.87) | 5 (3 to 7) | 2.19 (1.26 to 3.43) | 0.51 (-0.45 to 1.48) |
| Republic of Korea | 990 (626 to 1485) | 1.04 (0.66 to 1.56) | -2.44 (-2.66 to -2.22) | 479 (287 to 765) | 1.18 (0.73 to 1.86) | -2.24 (-2.51 to -1.98) | 512 (320 to 796) | 0.93 (0.59 to 1.43) | -2.64 (-2.79 to -2.48) |
| Republic of Moldova | 15 (10 to 21) | 0.25 (0.17 to 0.35) | 0.42 (-1.62 to 2.51) | 7 (5 to 10) | 0.29 (0.2 to 0.42) | 0.72 (-1.5 to 3) | 8 (5 to 11) | 0.22 (0.15 to 0.3) | 0.67 (-1.68 to 3.08) |
| Romania | 110 (74 to 162) | 0.29 (0.2 to 0.42) | 3.15 (2.56 to 3.74) | 49 (32 to 73) | 0.32 (0.21 to 0.47) | 3.59 (3.01 to 4.19) | 61 (40 to 91) | 0.26 (0.18 to 0.38) | 2.88 (2.23 to 3.53) |
| Russian Federation | 614 (520 to | 0.26 (0.22 | 2.18 | 273 (227 to | 0.31 (0.26 to | 2.07 | 341 (281 to | 0.22 (0.18 to | 2.26 |

| Location | Both Cases (95% UI) 2021 | Both ASDR (95% UI) 2021 | Both 1990-2021 AAPC (95% UI) | Men Cases (95% UI) 2021 | Men ASDR (95% UI) 2021 | Men 1990-2021AAPC (95% UI) | Women Cases (95% UI) 2021 | Women ASDR (95% UI) 2021 | Women 1990-2021 AAPC (95% UI) |
|---|---|---|---|---|---|---|---|---|---|
| | 717) | to 0.3) | (1.35 to 3) | 328) | 0.37) | (0.83 to 3.32) | 404) | 0.26) | (1.18 to 3.35) |
| Rwanda | 39 (23 to 59) | 0.66 (0.4 to 1.01) | -0.82 (-1.14 to -0.51) | 9 (4 to 18) | 0.36 (0.17 to 0.73) | -1.07 (-1.38 to -0.75) | 29 (18 to 44) | 0.86 (0.51 to 1.31) | -0.85 (-1.17 to -0.53) |
| Saint Kitts and Nevis | 0 (0 to 0) | 0.39 (0.26 to 0.56) | 0.24 (-0.98 to 1.47) | 0 (0 to 0) | 0.53 (0.34 to 0.78) | 1.56 (0.83 to 2.31) | 0 (0 to 0) | 0.28 (0.19 to 0.41) | -1.2 (-2.68 to 0.31) |
| Saint Lucia | 0 (0 to 1) | 0.16 (0.11 to 0.23) | -0.92 (-1.32 to -0.52) | 0 (0 to 0) | 0.17 (0.11 to 0.25) | 0.03 (-0.77 to 0.83) | 0 (0 to 0) | 0.14 (0.1 to 0.2) | -1.63 (-2.66 to -0.59) |
| Saint Vincent and the Grenadines | 0 (0 to 1) | 0.27 (0.18 to 0.38) | -0.42 (-1.04 to 0.21) | 0 (0 to 0) | 0.32 (0.21 to 0.46) | -0.16 (-0.72 to 0.41) | 0 (0 to 0) | 0.22 (0.15 to 0.3) | -0.95 (-2.06 to 0.16) |
| Samoa | 1 (0 to 1) | 0.54 (0.34 to 0.79) | -0.05 (-0.18 to 0.08) | 0 (0 to 1) | 0.63 (0.38 to 1.02) | -0.36 (-0.92 to 0.2) | 0 (0 to 0) | 0.46 (0.27 to 0.69) | 0.32 (0.25 to 0.39) |
| San Marino | 0 (0 to 0) | 0.17 (0.09 to 0.29) | 0.74 (0.67 to 0.82) | 0 (0 to 0) | 0.21 (0.11 to 0.37) | 0.58 (0.47 to 0.68) | 0 (0 to 0) | 0.14 (0.08 to 0.23) | 0.89 (0.61 to 1.17) |

| Location | Both: Cases (95% UI) 2021 | Both: ASDR (95% UI) 2021 | Both: 1990-2021 AAPC (95% UI) | Men: Cases (95% UI) 2021 | Men: ASDR (95% UI) 2021 | Men: 1990-2021AAPC (95% UI) | Women: Cases (95% UI) 2021 | Women: ASDR (95% UI) 2021 | Women: 1990-2021 AAPC (95% UI) |
|---|---|---|---|---|---|---|---|---|---|
| Sao Tome and Principe | 0 (0 to 1) | 0.34 (0.17 to 0.54) | 0.27 (0.02 to 0.52) | 0 (0 to 0) | 0.37 (0.19 to 0.73) | -0.61 (-1.54 to 0.32) | 0 (0 to 0) | 0.31 (0.13 to 0.55) | 0.48 (0.34 to 0.62) |
| Saudi Arabia | 172 (112 to 252) | 1.14 (0.74 to 1.67) | 1.46 (1.23 to 1.68) | 114 (73 to 167) | 1.33 (0.85 to 1.97) | 1.58 (1.24 to 1.92) | 58 (34 to 88) | 0.85 (0.51 to 1.29) | 1.06 (0.89 to 1.22) |
| Senegal | 116 (73 to 175) | 1.61 (1.01 to 2.46) | -0.17 (-0.53 to 0.18) | 33 (20 to 52) | 0.98 (0.59 to 1.58) | -1.06 (-1.55 to -0.57) | 82 (49 to 130) | 2.18 (1.28 to 3.43) | 0.18 (-0.1 to 0.46) |
| Serbia | 72 (44 to 112) | 0.43 (0.26 to 0.65) | 0.13 (0.02 to 0.23) | 32 (19 to 50) | 0.43 (0.26 to 0.66) | 0.59 (0.42 to 0.75) | 40 (24 to 64) | 0.42 (0.26 to 0.65) | -0.17 (-0.48 to 0.14) |
| Seychelles | 1 (0 to 1) | 0.5 (0.33 to 0.73) | -1.77 (-2.07 to -1.47) | 0 (0 to 1) | 0.68 (0.43 to 0.98) | -1.33 (-1.65 to -1) | 0 (0 to 0) | 0.34 (0.23 to 0.52) | -2.5 (-2.7 to -2.31) |
| Sierra Leone | 44 (27 to 71) | 1.23 (0.74 to 1.95) | -1.13 (-1.26 to -0.99) | 17 (10 to 27) | 0.92 (0.55 to 1.49) | -2.34 (-2.52 to -2.16) | 27 (16 to 46) | 1.53 (0.87 to 2.57) | -0.06 (-0.2 to 0.08) |
| Singapore | 27 (18 to | 0.32 (0.21 | -0.56 | 15 (9 to 24) | 0.38 (0.23 to | -0.75 | 12 (8 to 18) | 0.27 (0.17 to | -0.21 |

| Location | Both | | | Men | | | Women | | |
|---|---|---|---|---|---|---|---|---|---|
| | Cases (95% UI) 2021 | ASDR (95% UI) 2021 | 1990-2021 AAPC (95% UI) | Cases (95% UI) 2021 | ASDR (95% UI) 2021 | 1990-2021AAPC (95% UI) | Cases (95% UI) 2021 | ASDR (95% UI) 2021 | 1990-2021 AAPC (95% UI) |
| | 41) | to 0.49) | (-1.71 to 0.6) | | 0.6) | (-1.28 to -0.22) | | 0.4) | (-2.56 to 2.21) |
| Slovakia | 34 (20 to 54) | 0.36 (0.21 to 0.56) | -0.65 (-1.13 to -0.17) | 15 (9 to 24) | 0.38 (0.22 to 0.6) | 0.02 (-0.34 to 0.38) | 19 (11 to 30) | 0.34 (0.2 to 0.51) | -1.14 (-1.51 to -0.76) |
| Slovenia | 23 (15 to 34) | 0.49 (0.32 to 0.72) | 0.88 (-0.24 to 2.02) | 12 (8 to 19) | 0.61 (0.38 to 0.92) | 1.84 (1.64 to 2.03) | 11 (7 to 17) | 0.39 (0.25 to 0.58) | 0.13 (-0.97 to 1.25) |
| Solomon Islands | 1 (1 to 3) | 0.41 (0.23 to 0.72) | -0.36 (-0.53 to -0.19) | 1 (0 to 2) | 0.45 (0.24 to 0.9) | -0.46 (-0.64 to -0.28) | 1 (0 to 1) | 0.36 (0.2 to 0.63) | 0.01 (-0.34 to 0.37) |
| Somalia | 64 (32 to 124) | 1.2 (0.58 to 2.29) | 0.07 (-0.04 to 0.17) | 21 (6 to 49) | 0.87 (0.24 to 2.05) | -0.51 (-0.69 to -0.33) | 43 (21 to 85) | 1.4 (0.65 to 2.86) | 0.22 (0.15 to 0.28) |
| South Africa | 445 (359 to 538) | 1.02 (0.84 to 1.23) | 2.09 (1.66 to 2.51) | 252 (193 to 323) | 1.45 (1.12 to 1.84) | 3.25 (2.96 to 3.54) | 193 (161 to 230) | 0.76 (0.63 to 0.9) | 0.96 (0.49 to 1.43) |
| South Sudan | 37 (21 to 58) | 1.01 (0.6 to 1.65) | 1.24 (1.1 to 1.38) | 15 (7 to 27) | 0.77 (0.38 to 1.38) | 1.48 (1.1 to 1.86) | 22 (12 to 35) | 1.28 (0.72 to 2.07) | 0.97 (0.86 to 1.09) |

| Location | Both | | | Men | | | Women | | |
|---|---|---|---|---|---|---|---|---|---|
| | Cases (95% UI) 2021 | ASDR (95% UI) 2021 | 1990-2021 AAPC (95% UI) | Cases (95% UI) 2021 | ASDR (95% UI) 2021 | 1990-2021AAPC (95% UI) | Cases (95% UI) 2021 | ASDR (95% UI) 2021 | 1990-2021 AAPC (95% UI) |
| Spain | 313 (200 to 466) | 0.31 (0.2 to 0.45) | 1.25 (0.99 to 1.52) | 169 (107 to 256) | 0.39 (0.25 to 0.59) | 1.74 (1.32 to 2.17) | 145 (86 to 222) | 0.23 (0.15 to 0.34) | 0.77 (0.38 to 1.16) |
| Sri Lanka | 39 (22 to 64) | 0.15 (0.08 to 0.24) | -0.79 (-1.32 to -0.26) | 23 (12 to 38) | 0.19 (0.11 to 0.32) | -0.09 (-0.81 to 0.64) | 17 (9 to 27) | 0.11 (0.06 to 0.18) | -1.58 (-2.09 to -1.06) |
| Sudan | 92 (53 to 145) | 0.5 (0.29 to 0.8) | 1.39 (1.29 to 1.48) | 50 (26 to 81) | 0.52 (0.27 to 0.83) | 1.7 (1.58 to 1.83) | 42 (23 to 70) | 0.48 (0.27 to 0.8) | 1.04 (0.96 to 1.12) |
| Suriname | 2 (1 to 3) | 0.28 (0.17 to 0.42) | 1.13 (0.58 to 1.69) | 1 (1 to 1) | 0.31 (0.18 to 0.49) | 2.04 (1.22 to 2.87) | 1 (1 to 1) | 0.25 (0.16 to 0.37) | 0.35 (-0.01 to 0.7) |
| Sweden | 53 (42 to 65) | 0.24 (0.19 to 0.29) | 0.67 (-0.87 to 2.23) | 27 (21 to 35) | 0.27 (0.2 to 0.34) | 1.1 (-0.71 to 2.94) | 26 (20 to 32) | 0.21 (0.16 to 0.26) | 0.38 (-1.41 to 2.2) |
| Switzerland | 49 (31 to 74) | 0.26 (0.17 to 0.37) | 0.45 (-0.03 to 0.93) | 28 (17 to 44) | 0.32 (0.2 to 0.5) | -0.17 (-0.58 to 0.25) | 22 (14 to 32) | 0.2 (0.13 to 0.29) | 0.91 (0.62 to 1.2) |
| Syrian Arab Republic | 78 (48 to | 0.65 (0.4 to | -0.05 | 38 (22 to | 0.6 (0.35 to | 0.22 | 40 (24 to | 0.72 (0.44 to | -0.22 |

| Location | Both: Cases (95% UI) 2021 | Both: ASDR (95% UI) 2021 | Both: 1990-2021 AAPC (95% UI) | Men: Cases (95% UI) 2021 | Men: ASDR (95% UI) 2021 | Men: 1990-2021AAPC (95% UI) | Women: Cases (95% UI) 2021 | Women: ASDR (95% UI) 2021 | Women: 1990-2021 AAPC (95% UI) |
|---|---|---|---|---|---|---|---|---|---|
| | 117) | 0.97) | (-0.44 to 0.34) | 57) | 0.92) | (-0.19 to 0.63) | 61) | 1.07) | (-0.45 to 0.01) |
| Taiwan (Province of China) | 378 (257 to 531) | 0.89 (0.61 to 1.25) | 1.62 (-0.33 to 3.61) | 193 (128 to 282) | 0.98 (0.65 to 1.41) | 1.36 (-0.45 to 3.2) | 185 (120 to 265) | 0.8 (0.53 to 1.15) | 2.13 (-0.27 to 4.58) |
| Tajikistan | 17 (9 to 29) | 0.32 (0.17 to 0.54) | -0.21 (-0.52 to 0.09) | 7 (4 to 12) | 0.27 (0.14 to 0.5) | -0.2 (-0.49 to 0.09) | 10 (6 to 16) | 0.37 (0.2 to 0.61) | -0.12 (-0.58 to 0.34) |
| Thailand | 1243 (803 to 1863) | 1.15 (0.74 to 1.71) | -0.38 (-0.67 to -0.09) | 701 (431 to 1097) | 1.43 (0.89 to 2.23) | 0.08 (-0.25 to 0.41) | 542 (340 to 828) | 0.9 (0.57 to 1.37) | -0.95 (-1.2 to -0.7) |
| Timor-Leste | 2 (1 to 4) | 0.3 (0.16 to 0.5) | -0.25 (-0.47 to -0.02) | 1 (0 to 2) | 0.22 (0.1 to 0.46) | -0.13 (-0.3 to 0.04) | 2 (1 to 3) | 0.37 (0.16 to 0.62) | -0.37 (-0.6 to -0.15) |
| Togo | 41 (25 to 65) | 1.22 (0.72 to 1.93) | 0.47 (0.28 to 0.66) | 12 (6 to 24) | 0.8 (0.42 to 1.57) | -0.04 (-0.4 to 0.32) | 29 (15 to 49) | 1.5 (0.75 to 2.53) | 0.51 (0.35 to 0.66) |
| Tokelau | 0 (0 to 0) | 0.51 (0.28 to 0.89) | 0.76 (0.44 to 1.07) | 0 (0 to 0) | 0.53 (0.28 to 0.95) | 1.85 (1.44 to 2.27) | 0 (0 to 0) | 0.5 (0.26 to 0.88) | -0.01 (-0.45 to 0.43) |

| Location | Both | | | Men | | | Women | | |
|---|---|---|---|---|---|---|---|---|---|
| | Cases (95% UI) 2021 | ASDR (95% UI) 2021 | 1990-2021 AAPC (95% UI) | Cases (95% UI) 2021 | ASDR (95% UI) 2021 | 1990-2021AAPC (95% UI) | Cases (95% UI) 2021 | ASDR (95% UI) 2021 | 1990-2021 AAPC (95% UI) |
| Tonga | 2 (1 to 3) | 2.43 (1.47 to 3.6) | 0.59 (0.12 to 1.07) | 1 (1 to 2) | 3.34 (1.94 to 5.41) | 0.87 (0.21 to 1.54) | 1 (0 to 1) | 1.65 (0.97 to 2.57) | 0.19 (0.02 to 0.36) |
| Trinidad and Tobago | 5 (3 to 8) | 0.28 (0.18 to 0.4) | 0.3 (-0.85 to 1.46) | 2 (1 to 3) | 0.23 (0.14 to 0.36) | 0.88 (-0.8 to 2.59) | 3 (2 to 5) | 0.32 (0.21 to 0.48) | -0.01 (-0.82 to 0.8) |
| Tunisia | 23 (13 to 38) | 0.18 (0.1 to 0.29) | 1.43 (1.28 to 1.58) | 13 (7 to 21) | 0.21 (0.12 to 0.34) | 2.12 (1.97 to 2.27) | 10 (6 to 18) | 0.15 (0.08 to 0.26) | 0.75 (0.6 to 0.9) |
| Turkey | 322 (205 to 466) | 0.36 (0.23 to 0.52) | 1 (0.81 to 1.19) | 177 (108 to 265) | 0.44 (0.27 to 0.64) | 1.35 (1 to 1.7) | 145 (91 to 213) | 0.3 (0.19 to 0.43) | 0.61 (0.37 to 0.85) |
| Turkmenistan | 20 (13 to 31) | 0.52 (0.33 to 0.77) | 0.94 (-0.05 to 1.94) | 9 (6 to 15) | 0.54 (0.32 to 0.83) | 0.01 (-0.55 to 0.57) | 11 (7 to 17) | 0.51 (0.33 to 0.75) | 1.91 (0.78 to 3.05) |
| Tuvalu | 0 (0 to 0) | 0.52 (0.31 to 0.84) | 0.59 (0.53 to 0.65) | 0 (0 to 0) | 0.54 (0.28 to 0.96) | 1.06 (0.8 to 1.32) | 0 (0 to 0) | 0.51 (0.3 to 0.85) | 0.32 (0.19 to 0.45) |
| Uganda | 137 (85 to | 0.96 (0.58 | 0.28 | 51 (30 to | 0.8 (0.46 to | -0.47 | 86 (48 to | 1.07 (0.58 to | 0.78 |

| Location | Both: Cases (95% UI) 2021 | Both: ASDR (95% UI) 2021 | Both: 1990-2021 AAPC (95% UI) | Men: Cases (95% UI) 2021 | Men: ASDR (95% UI) 2021 | Men: 1990-2021AAPC (95% UI) | Women: Cases (95% UI) 2021 | Women: ASDR (95% UI) 2021 | Women: 1990-2021 AAPC (95% UI) |
|---|---|---|---|---|---|---|---|---|---|
| | 211) | to 1.49) | (0.15 to 0.41) | 79) | 1.26) | (-0.65 to -0.28) | 138) | 1.77) | (0.6 to 0.96) |
| Ukraine | 90 (63 to 119) | 0.12 (0.08 to 0.15) | -0.69 (-1.87 to 0.51) | 46 (29 to 66) | 0.16 (0.1 to 0.23) | -0.41 (-1.58 to 0.77) | 44 (28 to 63) | 0.09 (0.06 to 0.13) | -1.14 (-2.33 to 0.06) |
| United Arab Emirates | 58 (35 to 92) | 1.91 (1.15 to 2.96) | 1.77 (1.29 to 2.25) | 41 (23 to 68) | 1.6 (0.93 to 2.65) | 1.91 (1.42 to 2.39) | 17 (11 to 25) | 3.24 (1.88 to 4.9) | 2.78 (1.75 to 3.82) |
| United Kingdom | 582 (474 to 699) | 0.42 (0.35 to 0.5) | 3.64 (3.3 to 3.98) | 247 (201 to 298) | 0.4 (0.33 to 0.48) | 3.56 (3.26 to 3.86) | 335 (268 to 406) | 0.44 (0.36 to 0.52) | 3.78 (3.38 to 4.18) |
| United Republic of Tanzania | 179 (112 to 274) | 0.73 (0.44 to 1.09) | -0.15 (-0.26 to -0.04) | 57 (30 to 102) | 0.47 (0.25 to 0.83) | -0.79 (-0.91 to -0.67) | 122 (77 to 181) | 0.95 (0.58 to 1.43) | 0.15 (0.04 to 0.26) |
| United States of America | 2799 (2341 to 3270) | 0.47 (0.4 to 0.55) | 2.71 (2.52 to 2.9) | 1561 (1308 to 1839) | 0.58 (0.49 to 0.68) | 2.74 (2.54 to 2.94) | 1238 (1021 to 1456) | 0.37 (0.31 to 0.43) | 2.55 (2.35 to 2.75) |
| United States Virgin Islands | 1 (0 to 1) | 0.32 (0.2 to 0.49) | 1.23 (1 to 1.46) | 0 (0 to 1) | 0.43 (0.25 to 0.71) | 2.88 (2.5 to 3.25) | 0 (0 to 0) | 0.21 (0.13 to 0.34) | -0.51 (-0.67 to -0.34) |

| Location | Both: Cases (95% UI) 2021 | Both: ASDR (95% UI) 2021 | Both: 1990-2021 AAPC (95% UI) | Men: Cases (95% UI) 2021 | Men: ASDR (95% UI) 2021 | Men: 1990-2021AAPC (95% UI) | Women: Cases (95% UI) 2021 | Women: ASDR (95% UI) 2021 | Women: 1990-2021 AAPC (95% UI) |
|---|---|---|---|---|---|---|---|---|---|
| Uruguay | 13 (8 to 18) | 0.23 (0.15 to 0.33) | 3.47 (2.56 to 4.38) | 8 (5 to 12) | 0.34 (0.21 to 0.5) | 4.19 (3.57 to 4.82) | 5 (3 to 7) | 0.15 (0.1 to 0.2) | 2.63 (1.67 to 3.6) |
| Uzbekistan | 99 (62 to 150) | 0.38 (0.24 to 0.58) | 1.62 (1.25 to 1.99) | 43 (27 to 68) | 0.36 (0.22 to 0.55) | 1.64 (1.13 to 2.15) | 56 (35 to 85) | 0.4 (0.25 to 0.6) | 1.61 (1.29 to 1.93) |
| Vanuatu | 1 (0 to 1) | 0.45 (0.25 to 0.77) | 0.24 (-0.12 to 0.6) | 0 (0 to 1) | 0.47 (0.23 to 0.91) | 0.43 (0.1 to 0.76) | 0 (0 to 1) | 0.43 (0.25 to 0.76) | 0.1 (-0.15 to 0.34) |
| Venezuela (Bolivarian Republic of) | 83 (52 to 124) | 0.29 (0.18 to 0.43) | -1.89 (-2.75 to -1.02) | 37 (23 to 57) | 0.29 (0.18 to 0.45) | -0.86 (-1.9 to 0.2) | 46 (28 to 69) | 0.29 (0.18 to 0.43) | -2.47 (-3.62 to -1.29) |
| Viet Nam | 1020 (646 to 1527) | 1.04 (0.67 to 1.57) | -0.05 (-0.18 to 0.09) | 587 (346 to 950) | 1.35 (0.85 to 2.14) | -0.2 (-0.38 to -0.02) | 433 (260 to 654) | 0.8 (0.48 to 1.2) | 0.17 (-0.04 to 0.37) |
| Yemen | 24 (12 to 47) | 0.18 (0.09 to 0.37) | -0.49 (-0.63 to -0.35) | 11 (6 to 22) | 0.18 (0.09 to 0.36) | -0.97 (-1.16 to -0.78) | 13 (6 to 27) | 0.19 (0.08 to 0.41) | -0.01 (-0.14 to 0.11) |

| Location | Both | | | Men | | | Women | | |
|---|---|---|---|---|---|---|---|---|---|
| | Cases (95% UI) 2021 | ASDR (95% UI) 2021 | 1990-2021 AAPC (95% UI) | Cases (95% UI) 2021 | ASDR (95% UI) 2021 | 1990-2021AAPC (95% UI) | Cases (95% UI) 2021 | ASDR (95% UI) 2021 | 1990-2021 AAPC (95% UI) |
| Zambia | 49 (20 to 115) | 0.78 (0.34 to 1.78) | -1.39 (-1.71 to -1.07) | 19 (7 to 53) | 0.62 (0.25 to 1.66) | -1.64 (-2.01 to -1.27) | 30 (12 to 69) | 0.91 (0.38 to 2.05) | -1.43 (-1.69 to -1.17) |
| Zimbabwe | 125 (71 to 191) | 2.06 (1.18 to 3.22) | 1.3 (0.99 to 1.61) | 34 (17 to 62) | 1.24 (0.63 to 2.34) | 0.26 (-0.29 to 0.82) | 92 (48 to 148) | 2.61 (1.38 to 4.33) | 1.69 (1.1 to 2.28) |

**Table S4 Absolute deaths and age-standardized death rate (ASDR) of NALC globally and regionally in 1990 and 2021 across 21 GBD regions and 5 SDI regions.**

| Location | 1990 Cases (95% UI) | | | 2019 Cases (95% UI) | | | 2021 Cases (95% UI) | | |
|---|---|---|---|---|---|---|---|---|---|
| | Both | Women | Men | Both | Women | Men | Both | Women | Men |
| Global | 14,675 (11,621-18,159) | 7,558 (5,870-9,620) | 7,118 (5,598-8,864) | 39074.57 (31718.58 - 47007.82) | 19703.52 (15921.83 - 23902.04) | 19371.04 (15316.59 - 24070.74) | 40,925 (32,961-49,610) | 20,456 (16,632-25,003) | 20,469 (16,180-25,363) |
| Andean Latin America | 56 (39-77) | 43 (30-59) | 13 (9-19) | 198.16 (134.98 - 281.57) | 146.54 (100.21 - 207.10) | 51.62 (33.63 - 77.47) | 213 (139-306) | 158 (104-228) | 55 (36-81) |
| Australasia | 36 (26-48) | 17 (12-22) | 19 (13-26) | 272.16 (196.05 - 368.17) | 131.55 (91.29 - 181.13) | 140.62 (97.57 - 197.83) | 297 (211-404) | 142 (98-196) | 155 (103-212) |
| Caribbean | 53 (37-74) | 34 (23-46) | 20 (13-28) | 113.76 (79.27 - 156.24) | 62.08 (42.41 - 85.80) | 51.68 (36.00 - 73.68) | 121 (82-167) | 67 (45-94) | 54 (37-77) |
| Central Asia | 248 (173-349) | 140 (95-192) | 108 (74-157) | 445.80 (304.20 - 623.10) | 258.50 (176.55 - 362.40) | 187.30 (126.01 - 271.44) | 474 (318-679) | 275 (187-389) | 199 (130-296) |
| Central Europe | 401 | 252 | 150 | 650.36 (467.40 - | 366.22 (264.96 - | 284.14 (201.57 - | 669 | 376 | 294 |

| Location | 1990 Cases (95% UI) | | | 2019 Cases (95% UI) | | | 2021 Cases (95% UI) | | |
|---|---|---|---|---|---|---|---|---|---|
| | **Both** | **Women** | **Men** | **Both** | **Women** | **Men** | **Both** | **Women** | **Men** |
| | (277-562) | (174-345) | (102-215) | 864.98) | 487.26) | 391.40) | (475-908) | (271-500) | (204-409) |
| Central Latin America | 207 (159-267) | 131 (100-169) | 76 (59-100) | 725.32 (570.25 - 903.39) | 420.38 (331.42 - 523.83) | 304.94 (240.46 - 381.75) | 803 (631-1014) | 468 (359-594) | 335 (257-422) |
| Central Sub-Saharan Africa | 114 (49-250) | 63 (25-152) | 51 (20-122) | 212.88 (86.39 - 492.58) | 127.51 (48.13 - 326.17) | 85.37 (32.63 - 213.08) | 233 (96-535) | 139 (54-360) | 94 (37-225) |
| East Asia | 4,292 (3,440-5,304) | 2,071 (1567-2,597) | 2,221 (1,709-2,857) | 10974.71 (8502.96 - 13774.87) | 5391.43 (4109.94 - 6866.18) | 5583.28 (4034.71 - 7518.27) | 10,936 (8458-13,798) | 5,169 (3,920-6,823) | 5,767 (4,113-7,793) |
| Eastern Europe | 387 (323-460) | 223 (187-266) | 164 (136-194) | 753.53 (631.78 - 884.04) | 412.38 (345.45 - 484.66) | 341.15 (284.59 - 400.28) | 796 (668-935) | 434 (356-518) | 362 (300-436) |
| Eastern Sub-Saharan Africa | 532 (371-751) | 338 (241-472) | 194 (119-312) | 1180.89 (807.38 - 1639.70) | 753.05 (536.41 - 1021.79) | 427.84 (259.10 - 684.85) | 1,261 (865-1,739) | 808 (572-1073) | 453 (270-727) |
| High SDI | 3,360 | 1,710 | 1,650 | 9078.80 (7096.75 | 4486.90 (3406.95 | 4591.90 (3662.89 | 9,585 | 4,700 | 4,885 |

| Location | 1990 Cases (95% UI) | | | 2019 Cases (95% UI) | | | 2021 Cases (95% UI) | | |
|---|---|---|---|---|---|---|---|---|---|
| | Both | Women | Men | Both | Women | Men | Both | Women | Men |
| | (2,663-4,196) | (1,337-2,146) | (1,316-2,101) | - 11328.93) | - 5700.33) | - 5797.04) | (7,410-11,975) | (3,555-5,978) | (3,841-6,150) |
| High-income Asia Pacific | 1,436 (1,132-1,835) | 686 (517-894) | 751 (602-959) | 2304.36 (1770.01 - 2986.55) | 1189.29 (860.50 - 1570.51) | 1115.08 (872.88 - 1438.52) | 2,490 (1,864-3,212) | 1,289 (903-1,729) | 1,201 (926-1,562) |
| High-income North America | 744 (630-873) | 383 (319-448) | 361 (304-426) | 3146.59 (2593.11 - 3732.03) | 1458.53 (1190.99 - 1726.24) | 1688.05 (1398.86 - 2015.60) | 3,302 (2,726-3,914) | 1,508 (1,218-1,805) | 1,793 (1,486-2,133) |
| High-middle SDI | 3,102 (2,540-3,748) | 1,523 (1,208-1,867) | 1,579 (1,249-1,964) | 7323.95 (5814.73 - 9014.42) | 3585.60 (2812.34 - 4425.41) | 3738.35 (2834.12 - 4821.64) | 7,438 (5,952-9,089) | 3,556 (2,826-4,423) | 3,882 (2,931-4,938) |
| Low SDI | 1,465 (942-2,243) | 825 (520-1342) | 639 (397-962) | 3019.38 (2148.21 - 4129.08) | 1811.37 (1294.30 - 2485.00) | 1208.01 (819.02 - 1731.43) | 3,222 (2,283-4,455) | 1,949 (1,396-2,661) | 1,273 (865-1,833) |
| Low-middle | 2,144 | 1,154 | 990 | 6561.92 (5263.20 | 3460.41 (2799.62 | 3101.51 (2394.87 | 7,040 | 3,733 | 3,308 |

| Location | 1990 Cases (95% UI) | | | 2019 Cases (95% UI) | | | 2021 Cases (95% UI) | | |
|---|---|---|---|---|---|---|---|---|---|
| | **Both** | **Women** | **Men** | **Both** | **Women** | **Men** | **Both** | **Women** | **Men** |
| SDI | (1,572-3,011 ) | (823-1,67 7) | (736-1,33 4) | - 8024.35) | - 4280.06) | - 3921.63) | (5,612-8,681 ) | (2,969-4,636 ) | (2,517-4,165 ) |
| Middle SDI | 4,592 (3,709-5,603 ) | 2,337 (1,844-2,8 56) | 2,255 (1,762-2,8 03) | 13064.15 (10541.50 - 15743.29) | 6345.49 (5071.88 - 7713.99) | 6718.66 (5200.09 - 8561.74) | 13,613 (10,818-16,6 83) | 6,504 (5,233-8,029 ) | 7,109 (5,434-9,006 ) |
| North Africa and Middle East | 694 (438-1,140) | 389 (229-701) | 305 (202-479) | 2749.13 (1982.89 - 3678.99) | 1299.01 (974.68 - 1727.75) | 1450.12 (1013.51 - 1990.54) | 2,966 (2,066-4,066 ) | 1,396 (1,020-1881 ) | 1,570 (1,066-2,214 ) |
| Oceania | 11 (6-22) | 5 (3-11) | 6 (3-12) | 23.50 (13.41 - 41.85) | 11.56 (6.87 - 19.41) | 11.94 (6.32 - 23.88) | 25 (14-46) | 12 (7-21) | 13 (7-27) |
| South Asia | 1,375 (1,108-1,649 ) | 615 (494-734) | 760 (600-934) | 5056.54 (4191.70 - 5929.69) | 2496.43 (2063.55 - 2924.40) | 2560.11 (2077.19 - 3048.06) | 5,469 (4,600-6,486 ) | 2,726 (2,223-3,236 ) | 2,744 (2,238-3,426 ) |
| Southeast Asia | 1,484 (1,070-2,019 | 707 (517-943) | 777 (541-1,08 | 3997.25 (2858.60 - 5427.95) | 1792.12 (1131.32 - 2488.42) | 2205.13 (1534.31 - 3173.11) | 4,267 (3,013-5,833 | 1,900 (1,187-2,704 | 2,366 (1,644-3,467 |

| Location | 1990 Cases (95% UI) | | | 2019 Cases (95% UI) | | | 2021 Cases (95% UI) | | |
|---|---|---|---|---|---|---|---|---|---|
| | **Both** | **Women** | **Men** | **Both** | **Women** | **Men** | **Both** | **Women** | **Men** |
| | ) | | 5) | | | | ) | ) | ) |
| Southern Latin America | 32 (22-46) | 18 (11-24) | 15 (9-22) | 157.33 (106.59 - 222.30) | 81.19 (54.83 - 112.54) | 76.14 (50.32 - 111.78) | 171 (113-242) | 88 (59-122) | 84 (53-124) |
| Southern Sub-Saharan Africa | 178 (108-268) | 107 (73-157) | 71 (30-133) | 584.41 (467.58 - 721.10) | 288.27 (227.99 - 374.30) | 296.14 (217.75 - 388.48) | 623 (492-769) | 307 (242-397) | 316 (235-417) |
| Tropical Latin America | 115 (99-132) | 72 (62-82) | 43 (37-50) | 392.69 (333.45 - 459.17) | 216.95 (183.78 - 254.10) | 175.74 (149.18 - 206.67) | 423 (356-493) | 239 (199-277) | 185 (157-217) |
| Western Europe | 1,217 (907-1,612) | 652 (474-877) | 565 (417-769) | 3067.47 (2193.32 - 4156.19) | 1542.47 (1067.93 - 2122.06) | 1525.00 (1097.21 - 2070.72) | 3,159 (2,255-4,272 ) | 1,580 (1,078-2,166 ) | 1,579 (1,136-2,155 ) |
| Western Sub-Saharan Africa | 1,062 (623-1,781) | 612 (336-1116 ) | 450 (252-706) | 2067.72 (1527.29 - 2771.48) | 1258.06 (920.04 - 1695.19) | 809.66 (577.30 - 1125.98) | 2,227 (1,654-2,944 ) | 1,376 (1,015-1,829 ) | 850 (598-1,176) |

## Table S5 Absolute and age-standardized death rate (ASDR) for NALC in men and women globally over 30 years from 1990 to 2021

| Year | Both | | Men | | Women | |
|---|---|---|---|---|---|---|
| | Cases (95% UI) | ASDR (95% UI) | Cases (95% UI) | ASDR (95% UI) | Cases (95% UI) | ASDR (95% UI) |
| 1990 | 14675 (11621,18159) | 0.38 (0.3,0.47) | 7118 (5598,8864) | 0.39 (0.31,0.49) | 7558 (5870,9620) | 0.36 (0.28,0.46) |
| 1991 | 15136 (12001,18903) | 0.38 (0.3,0.47) | 7369 (5816,9240) | 0.4 (0.32,0.5) | 7767 (6002,9896) | 0.36 (0.28,0.46) |
| 1992 | 15660 (12355,19555) | 0.38 (0.3,0.48) | 7641 (6030,9591) | 0.4 (0.32,0.51) | 8019 (6259,10147) | 0.36 (0.28,0.46) |
| 1993 | 16198 (12757,20083) | 0.39 (0.31,0.48) | 7945 (6296,10002) | 0.41 (0.33,0.52) | 8253 (6424,10464) | 0.36 (0.28,0.46) |
| 1994 | 16831 (13464,20804) | 0.39 (0.31,0.49) | 8274 (6599,10421) | 0.42 (0.33,0.52) | 8557 (6720,10751) | 0.37 (0.29,0.46) |
| 1995 | 17625 (14024,21785) | 0.4 (0.32,0.5) | 8689 (6967,10707) | 0.43 (0.35,0.54) | 8936 (7059,11081) | 0.38 (0.3,0.47) |
| 1996 | 18300 (14637,22589) | 0.41 (0.33,0.51) | 9047 (7221,11260) | 0.44 (0.35,0.54) | 9253 (7323,11433) | 0.38 (0.3,0.47) |
| 1997 | 18843 (15119,23353) | 0.41 (0.33,0.51) | 9322 (7441,11572) | 0.44 (0.35,0.55) | 9520 (7581,11816) | 0.39 (0.31,0.48) |
| 1998 | 19626 (15860,24194) | 0.42 (0.34,0.52) | 9763 (7734,12148) | 0.45 (0.36,0.57) | 9863 (7933,12216) | 0.39 (0.31,0.48) |
| 1999 | 20442 (16508,25065) | 0.43 (0.35,0.53) | 10173 (8195,12769) | 0.46 (0.37,0.58) | 10269 (8193,12663) | 0.4 (0.32,0.49) |
| 2000 | 21185 (17154,25852) | 0.43 (0.35,0.53) | 10581 (8437,13070) | 0.47 (0.37,0.58) | 10603 (8478,12968) | 0.4 (0.32,0.49) |
| 2001 | 21722 (17607,26679) | 0.43 (0.35,0.53) | 10854 (8736,13635) | 0.47 (0.38,0.59) | 10869 (8773,13240) | 0.4 (0.32,0.49) |
| 2002 | 22221 (18061,27355) | 0.43 (0.35,0.54) | 11090 (8900,13832) | 0.47 (0.38,0.59) | 11131 (9011,13643) | 0.4 (0.32,0.49) |
| 2003 | 22547 (18349,27521) | 0.43 (0.35,0.52) | 11258 (9081,13912) | 0.46 (0.37,0.57) | 11289 (9157,13675) | 0.4 (0.32,0.48) |

| Year | Both | | Men | | Women | |
|---|---|---|---|---|---|---|
| | Cases (95% UI) | ASDR (95% UI) | Cases (95% UI) | ASDR (95% UI) | Cases (95% UI) | ASDR (95% UI) |
| 2004 | 22831 (18796,27989) | 0.42 (0.35,0.52) | 11401 (9218,14125) | 0.46 (0.37,0.56) | 11430 (9293,13874) | 0.39 (0.32,0.48) |
| 2005 | 23380 (19169,28566) | 0.42 (0.35,0.52) | 11666 (9502,14318) | 0.46 (0.37,0.57) | 11714 (9522,14277) | 0.39 (0.32,0.48) |
| 2006 | 23983 (19639,29440) | 0.42 (0.35,0.52) | 11932 (9694,14869) | 0.46 (0.37,0.57) | 12051 (9764,14720) | 0.39 (0.32,0.48) |
| 2007 | 24862 (20374,30481) | 0.43 (0.35,0.53) | 12355 (9980,15370) | 0.46 (0.37,0.57) | 12506 (10091,15233) | 0.4 (0.32,0.48) |
| 2008 | 26104 (21476,31737) | 0.44 (0.36,0.53) | 12971 (10428,15904) | 0.47 (0.38,0.58) | 13132 (10646,15881) | 0.41 (0.33,0.49) |
| 2009 | 27181 (22336,33106) | 0.44 (0.36,0.54) | 13498 (11033,16618) | 0.48 (0.39,0.59) | 13683 (10996,16699) | 0.41 (0.33,0.5) |
| 2010 | 28259 (23212,34209) | 0.45 (0.37,0.54) | 14053 (11385,17259) | 0.48 (0.39,0.59) | 14207 (11555,17167) | 0.42 (0.34,0.5) |
| 2011 | 29164 (23853,35345) | 0.45 (0.37,0.55) | 14501 (11690,17762) | 0.48 (0.39,0.59) | 14663 (11868,17656) | 0.42 (0.34,0.5) |
| 2012 | 30276 (24953,36855) | 0.45 (0.37,0.55) | 15039 (12129,18514) | 0.49 (0.39,0.6) | 15237 (12254,18375) | 0.42 (0.34,0.51) |
| 2013 | 31821 (26133,38533) | 0.46 (0.38,0.56) | 15801 (12691,19419) | 0.5 (0.41,0.62) | 16020 (13109,19218) | 0.43 (0.35,0.52) |
| 2014 | 33497 (27635,40620) | 0.48 (0.39,0.58) | 16628 (13601,20403) | 0.51 (0.42,0.62) | 16868 (13725,20388) | 0.44 (0.36,0.53) |
| 2015 | 34802 (28636,42264) | 0.48 (0.39,0.58) | 17310 (13999,21225) | 0.52 (0.42,0.63) | 17492 (14259,21292) | 0.45 (0.36,0.54) |
| 2016 | 35977 (29481,43083) | 0.48 (0.4,0.58) | 17803 (14311,22063) | 0.52 (0.42,0.64) | 18174 (15011,21892) | 0.45 (0.37,0.54) |
| 2017 | 37019 (30390,45204) | 0.48 (0.4,0.59) | 18261 (14433,22592) | 0.51 (0.41,0.63) | 18758 (15306,22814) | 0.45 (0.37,0.55) |
| 2018 | 38188 (31086,46197) | 0.48 (0.39,0.58) | 18873 (15014,23594) | 0.52 (0.41,0.65) | 19315 (15691,23174) | 0.45 (0.37,0.54) |

| Year | Both | | Men | | Women | |
|---|---|---|---|---|---|---|
| | **Cases (95% UI)** | **ASDR (95% UI)** | **Cases (95% UI)** | **ASDR (95% UI)** | **Cases (95% UI)** | **ASDR (95% UI)** |
| 2019 | 39075 (31719,47008) | 0.48 (0.39,0.58) | 19371 (15317,24071) | 0.52 (0.41,0.64) | 19704 (15922,23902) | 0.45 (0.36,0.54) |
| 2020 | 39930 (31997,48130) | 0.48 (0.39,0.58) | 19999 (15540,24759) | 0.52 (0.41,0.64) | 19930 (16024,24218) | 0.44 (0.36,0.54) |
| 2021 | 40925 (32961,49610) | 0.48 (0.39,0.58) | 20469 (16180,25363) | 0.52 (0.41,0.64) | 20456 (16632,25003) | 0.44 (0.36,0.54) |

**Table S6 The annual percentage change (APC) of ASDR of NALC in 21 GBD regions and global, from 1990 to 2021**

| Location | Joinpoint number | Segment order | Segment Start year | Segment End year | APC (95%CI) | P Value |
|---|---|---|---|---|---|---|
| Global | 3 | 0 | 1990 | 2000 | 1.54 (1.42 to 1.66) | <0.001 |
| Global | 3 | 1 | 2000 | 2006 | -0.42 (-0.76 to -0.08) | 0.018 |
| Global | 3 | 2 | 2006 | 2016 | 1.36 (1.22 to 1.5) | <0.001 |
| Global | 3 | 3 | 2016 | 2021 | -0.21 (-0.55 to 0.13) | 0.210 |
| Andean Latin America | 5 | 0 | 1990 | 1996 | 1.53 (0.86 to 2.2) | <0.001 |
| Andean Latin America | 5 | 1 | 1996 | 1999 | -8.8 (-12.3 to -5.15) | <0.001 |
| Andean Latin America | 5 | 2 | 1999 | 2003 | 5.65 (3.6 to 7.74) | <0.001 |

| Location | Joinpoint number | Segment order | Segment Start year | Segment End year | APC (95%CI) | P Value |
|---|---|---|---|---|---|---|
| Andean Latin America | 5 | 3 | 2003 | 2007 | -1.74 (-3.65 to 0.2) | 0.075 |
| Andean Latin America | 5 | 4 | 2007 | 2013 | 0.97 (0.09 to 1.86) | 0.033 |
| Andean Latin America | 5 | 5 | 2013 | 2021 | 2.65 (2.21 to 3.09) | <0.001 |
| Australasia | 4 | 0 | 1990 | 1994 | 7.89 (6.68 to 9.12) | <0.001 |
| Australasia | 4 | 1 | 1994 | 2002 | 4.45 (3.95 to 4.95) | <0.001 |
| Australasia | 4 | 2 | 2002 | 2012 | 3.29 (2.96 to 3.63) | <0.001 |
| Australasia | 4 | 3 | 2012 | 2016 | 5.94 (4.07 to 7.86) | <0.001 |
| Australasia | 4 | 4 | 2016 | 2021 | 1.51 | 0.001 |

| Location | Joinpoint number | Segment order | Segment Start year | Segment End year | APC (95%CI) | P Value |
|---|---|---|---|---|---|---|
| | | | | | (0.7 to 2.32) | |
| Caribbean | 3 | 0 | 1990 | 1999 | 0.17 (-0.38 to 0.71) | 0.531 |
| Caribbean | 3 | 1 | 1999 | 2002 | -6.62 (-12.02 to -0.88) | 0.026 |
| Caribbean | 3 | 2 | 2002 | 2015 | 0.84 (0.48 to 1.2) | <0.001 |
| Caribbean | 3 | 3 | 2015 | 2021 | 2.3 (1.28 to 3.34) | <0.001 |
| Central Asia | 5 | 0 | 1990 | 1992 | 4.34 (2.26 to 6.46) | <0.001 |
| Central Asia | 5 | 1 | 1992 | 1999 | -0.63 (-0.97 to -0.29) | 0.001 |
| Central Asia | 5 | 2 | 1999 | 2006 | 0.31 (-0.03 to 0.65) | 0.072 |

| Location | Joinpoint number | Segment order | Segment Start year | Segment End year | APC (95%CI) | P Value |
|---|---|---|---|---|---|---|
| Central Asia | 5 | 3 | 2006 | 2010 | 2.34 (1.31 to 3.37) | <0.001 |
| Central Asia | 5 | 4 | 2010 | 2017 | -0.63 (-0.97 to -0.3) | 0.001 |
| Central Asia | 5 | 5 | 2017 | 2021 | 0.54 (-0.1 to 1.18) | 0.092 |
| Central Europe | 4 | 0 | 1990 | 2004 | -0.96 (-1.13 to -0.8) | <0.001 |
| Central Europe | 4 | 1 | 2004 | 2009 | 2.37 (1.24 to 3.51) | <0.001 |
| Central Europe | 4 | 2 | 2009 | 2013 | -2.4 (-4.09 to -0.67) | 0.009 |
| Central Europe | 4 | 3 | 2013 | 2017 | 4.15 (2.34 to 5.99) | <0.001 |
| Central Europe | 4 | 4 | 2017 | 2021 | 0.04 | 0.938 |

| Location | Joinpoint number | Segment order | Segment Start year | Segment End year | APC (95%CI) | P Value |
|---|---|---|---|---|---|---|
| | | | | | (-1.06 to 1.16) | |
| Central Latin America | 2 | 0 | 1990 | 1992 | 2.16 (-4.91 to 9.74) | 0.545 |
| Central Latin America | 2 | 1 | 1992 | 2000 | -2.22 (-3.15 to -1.28) | <0.001 |
| Central Latin America | 2 | 2 | 2000 | 2021 | 1.85 (1.66 to 2.03) | <0.001 |
| Central Sub-Saharan Africa | 3 | 0 | 1990 | 1996 | -0.06 (-0.28 to 0.16) | 0.551 |
| Central Sub-Saharan Africa | 3 | 1 | 1996 | 2008 | -1.82 (-1.91 to -1.74) | <0.001 |
| Central Sub-Saharan Africa | 3 | 2 | 2008 | 2017 | -0.17 (-0.32 to -0.03) | 0.018 |
| Central Sub-Saharan Africa | 3 | 3 | 2017 | 2021 | 1.72 (1.31 to 2.14) | <0.001 |

| Location | Joinpoint number | Segment order | Segment Start year | Segment End year | APC (95%CI) | P Value |
|---|---|---|---|---|---|---|
| East Asia | 4 | 0 | 1990 | 1995 | -0.54 (-1.76 to 0.7) | 0.373 |
| East Asia | 4 | 1 | 1995 | 2001 | 2.12 (0.86 to 3.39) | 0.002 |
| East Asia | 4 | 2 | 2001 | 2005 | -3.89 (-6.51 to -1.18) | 0.008 |
| East Asia | 4 | 3 | 2005 | 2015 | 2.17 (1.66 to 2.69) | <0.001 |
| East Asia | 4 | 4 | 2015 | 2021 | -1.76 (-2.67 to -0.83) | 0.001 |
| Eastern Europe | 5 | 0 | 1990 | 1994 | 4.06 (2.12 to 6.05) | <0.001 |
| Eastern Europe | 5 | 1 | 1994 | 2003 | -1.24 (-1.88 to -0.59) | 0.001 |
| Eastern Europe | 5 | 2 | 2003 | 2008 | 6.45 | <0.001 |

| Location | Joinpoint number | Segment order | Segment Start year | Segment End year | APC (95%CI) | P Value |
|---|---|---|---|---|---|---|
| | | | | | (4.46 to 8.48) | |
| Eastern Europe | 5 | 3 | 2008 | 2015 | 2.12 (1.09 to 3.16) | <0.001 |
| Eastern Europe | 5 | 4 | 2015 | 2018 | -2.79 (-8.43 to 3.19) | 0.328 |
| Eastern Europe | 5 | 5 | 2018 | 2021 | 2.53 (-0.49 to 5.64) | 0.095 |
| Eastern Sub-Saharan Africa | 5 | 0 | 1990 | 1996 | 1.61 (1.5 to 1.73) | <0.001 |
| Eastern Sub-Saharan Africa | 5 | 1 | 1996 | 2000 | 0.29 (-0.04 to 0.62) | 0.084 |
| Eastern Sub-Saharan Africa | 5 | 2 | 2000 | 2005 | -0.49 (-0.7 to -0.28) | <0.001 |
| Eastern Sub-Saharan Africa | 5 | 3 | 2005 | 2009 | -1.04 (-1.37 to -0.72) | <0.001 |

| Location | Joinpoint number | Segment order | Segment Start year | Segment End year | APC (95%CI) | P Value |
|---|---|---|---|---|---|---|
| Eastern Sub-Saharan Africa | 5 | 4 | 2009 | 2019 | -0.13 (-0.19 to -0.07) | <0.001 |
| Eastern Sub-Saharan Africa | 5 | 5 | 2019 | 2021 | 1.05 (0.39 to 1.72) | 0.004 |
| High-income Asia Pacific | 5 | 0 | 1990 | 1993 | -0.29 (-1.08 to 0.5) | 0.442 |
| High-income Asia Pacific | 5 | 1 | 1993 | 1996 | 6.51 (4.83 to 8.22) | <0.001 |
| High-income Asia Pacific | 5 | 2 | 1996 | 1999 | -0.17 (-1.75 to 1.43) | 0.824 |
| High-income Asia Pacific | 5 | 3 | 1999 | 2016 | -2.69 (-2.75 to -2.63) | <0.001 |
| High-income Asia Pacific | 5 | 4 | 2016 | 2019 | -4.96 (-6.46 to -3.44) | <0.001 |
| High-income Asia Pacific | 5 | 5 | 2019 | 2021 | 2.31 | 0.008 |

| Location | Joinpoint number | Segment order | Segment Start year | Segment End year | APC (95%CI) | P Value |
|---|---|---|---|---|---|---|
| | | | | | (0.7 to 3.95) | |
| High-income North America | 3 | 0 | 1990 | 2001 | 4.03 (3.86 to 4.2) | <0.001 |
| High-income North America | 3 | 1 | 2001 | 2007 | 2.33 (1.78 to 2.89) | <0.001 |
| High-income North America | 3 | 2 | 2007 | 2017 | 2.97 (2.75 to 3.2) | <0.001 |
| High-income North America | 3 | 3 | 2017 | 2021 | -0.19 (-0.95 to 0.57) | 0.602 |
| North Africa and Middle East | 4 | 0 | 1990 | 2002 | 0.84 (0.68 to 0.99) | <0.001 |
| North Africa and Middle East | 4 | 1 | 2002 | 2007 | -0.81 (-1.63 to 0.03) | 0.057 |
| North Africa and Middle East | 4 | 2 | 2007 | 2013 | 2.88 (2.27 to 3.49) | <0.001 |

| Location | Joinpoint number | Segment order | Segment Start year | Segment End year | APC (95%CI) | P Value |
|---|---|---|---|---|---|---|
| North Africa and Middle East | 4 | 3 | 2013 | 2018 | 3.91 (3.05 to 4.79) | <0.001 |
| North Africa and Middle East | 4 | 4 | 2018 | 2021 | 1.2 (-0.13 to 2.54) | 0.075 |
| Oceania | 3 | 0 | 1990 | 2005 | 0.59 (0.53 to 0.64) | <0.001 |
| Oceania | 3 | 1 | 2005 | 2009 | -2.67 (-3.31 to -2.02) | <0.001 |
| Oceania | 3 | 2 | 2009 | 2014 | -1.34 (-1.76 to -0.93) | <0.001 |
| Oceania | 3 | 3 | 2014 | 2021 | -0.37 (-0.54 to -0.19) | <0.001 |
| South Asia | 1 | 0 | 1990 | 2004 | 0.9 (0.72 to 1.07) | <0.001 |
| South Asia | 1 | 1 | 2004 | 2021 | 1.96 | <0.001 |

| Location | Joinpoint number | Segment order | Segment Start year | Segment End year | APC (95%CI) | P Value |
|---|---|---|---|---|---|---|
| | | | | | (1.83 to 2.09) | |
| Southeast Asia | 4 | 0 | 1990 | 1996 | 1.25 (0.99 to 1.52) | <0.001 |
| Southeast Asia | 4 | 1 | 1996 | 2005 | 0.8 (0.63 to 0.97) | <0.001 |
| Southeast Asia | 4 | 2 | 2005 | 2010 | 0.18 (-0.31 to 0.66) | 0.458 |
| Southeast Asia | 4 | 3 | 2010 | 2017 | -0.46 (-0.72 to -0.2) | 0.002 |
| Southeast Asia | 4 | 4 | 2017 | 2021 | 0.2 (-0.29 to 0.69) | 0.400 |
| Southern Latin America | 4 | 0 | 1990 | 1998 | 1.88 (1.24 to 2.52) | <0.001 |
| Southern Latin America | 4 | 1 | 1998 | 2002 | 6.12 (3.11 to 9.21) | <0.001 |

| Location | Joinpoint number | Segment order | Segment Start year | Segment End year | APC (95%CI) | P Value |
|---|---|---|---|---|---|---|
| Southern Latin America | 4 | 2 | 2002 | 2009 | 4.13 (3.12 to 5.15) | <0.001 |
| Southern Latin America | 4 | 3 | 2009 | 2012 | 8.01 (1.97 to 14.4) | 0.011 |
| Southern Latin America | 4 | 4 | 2012 | 2021 | 1.17 (0.64 to 1.71) | <0.001 |
| Southern Sub-Saharan Africa | 4 | 0 | 1990 | 1995 | 4.99 (4.3 to 5.67) | <0.001 |
| Southern Sub-Saharan Africa | 4 | 1 | 1995 | 1998 | 10.09 (6.92 to 13.35) | <0.001 |
| Southern Sub-Saharan Africa | 4 | 2 | 1998 | 2004 | 3.09 (2.42 to 3.76) | <0.001 |
| Southern Sub-Saharan Africa | 4 | 3 | 2004 | 2013 | -2.18 (-2.49 to -1.86) | <0.001 |
| Southern Sub-Saharan Africa | 4 | 4 | 2013 | 2021 | 0.26 | 0.101 |

| Location | Joinpoint number | Segment order | Segment Start year | Segment End year | APC (95%CI) | P Value |
|---|---|---|---|---|---|---|
| | | | | | (-0.06 to 0.58) | |
| Tropical Latin America | 2 | 0 | 1990 | 1998 | -2.41 (-2.93 to -1.89) | <0.001 |
| Tropical Latin America | 2 | 1 | 1998 | 2015 | 2.84 (2.65 to 3.04) | <0.001 |
| Tropical Latin America | 2 | 2 | 2015 | 2021 | -0.88 (-1.69 to -0.05) | 0.038 |
| Western Europe | 5 | 0 | 1990 | 1994 | 2.67 (2.08 to 3.26) | <0.001 |
| Western Europe | 5 | 1 | 1994 | 1997 | 0.81 (-1.02 to 2.66) | 0.363 |
| Western Europe | 5 | 2 | 1997 | 2000 | 3.31 (1.44 to 5.21) | 0.002 |
| Western Europe | 5 | 3 | 2000 | 2005 | 0.22 (-0.36 to 0.8) | 0.436 |

| Location | Joinpoint number | Segment order | Segment Start year | Segment End year | APC (95%CI) | P Value |
|---|---|---|---|---|---|---|
| Western Europe | 5 | 4 | 2005 | 2013 | 2.58 (2.33 to 2.83) | <0.001 |
| Western Europe | 5 | 5 | 2013 | 2021 | 0.04 (-0.16 to 0.24) | 0.691 |
| Western Sub-Saharan Africa | 5 | 0 | 1990 | 1994 | 1.22 (1.01 to 1.44) | <0.001 |
| Western Sub-Saharan Africa | 5 | 1 | 1994 | 1999 | 0.14 (-0.08 to 0.35) | 0.194 |
| Western Sub-Saharan Africa | 5 | 2 | 1999 | 2007 | -1.37 (-1.46 to -1.28) | <0.001 |
| Western Sub-Saharan Africa | 5 | 3 | 2007 | 2014 | 0.26 (0.14 to 0.37) | <0.001 |
| Western Sub-Saharan Africa | 5 | 4 | 2014 | 2019 | -0.46 (-0.67 to -0.25) | <0.001 |
| Western Sub-Saharan Africa | 5 | 5 | 2019 | 2021 | 1.15 | 0.003 |

| Location | Joinpoint number | Segment order | Segment Start year | Segment End year | APC (95%CI) | P Value |
|---|---|---|---|---|---|---|
| | | | | | (0.46 to 1.84) | |

**Table S7 Changes in NALC deaths in men and Women at global and regional levels from 1990 to 2021, decomposed by three population-level determinants: population aging, population growth, and epidemiological changes.**

| | Overall difference | | | Aging | | | Population | | | Epidemiological change | | |
|---|---|---|---|---|---|---|---|---|---|---|---|---|
| location | Both | Women | Men | Both | Women | Men | Both | Women | Men | Both | Women | Men |
| Andean Latin America | 156.09 | 114.7 | 41.39 | 44.35 (28.41%) | 35.46 (30.92%) | 10.08 (24.36%) | 84.09 (53.87%) | 62.44 (54.44%) | 21.15 (51.1%) | 27.66 (17.72%) | 16.8 (14.65%) | 10.16 (24.54%) |
| Australasia | 261.09 | 125.13 | 135.96 | 49.12 (18.81%) | 20.38 (16.29%) | 29.61 (21.78%) | 62.71 (24.02%) | 30.23 (24.16%) | 32.46 (23.88%) | 149.26 (57.17%) | 74.52 (59.55%) | 73.88 (54.34%) |
| Caribbean | 67.79 | 33.27 | 34.52 | 28.35 (41.82%) | 18.56 (55.78%) | 10.17 (29.45%) | 33.72 (49.74%) | 19.84 (59.64%) | 13.89 (40.24%) | 5.72 (8.44%) | -5.13 (-15.42%) | 10.46 (30.3%) |
| Central Asia | 225.89 | 135.09 | 90.8 | 38.52 (17.05%) | 9.88 (7.31%) | 29.57 (32.56%) | 149.88 (66.35%) | 82.94 (61.4%) | 66.57 (73.31%) | 37.48 (16.59%) | 42.27 (31.29%) | -5.34 (-5.88%) |
| Central Europe | 268.13 | 123.93 | 144.2 | 222.54 | 134.34 | 88.05 | 10.75 | 7.07 | 3.86 | 34.84 | -17.48 | 52.29 |

| | Overall difference | | | Aging | | | Population | | | Epidemiological change | | |
|---|---|---|---|---|---|---|---|---|---|---|---|---|
| location | Both | Women | Men | Both | Women | Men | Both | Women | Men | Both | Women | Men |
| | | | | (83%) | (108.4%) | (61.06%) | (4.01%) | (5.71%) | (2.67%) | (13%) | (-14.11%) | (36.26%) |
| Central Latin America | 595.86 | 337.14 | 258.72 | 216.25 (36.29%) | 139.51 (41.38%) | 79.1 (30.57%) | 277.26 (46.53%) | 170.33 (50.52%) | 108.17 (41.81%) | 102.36 (17.18%) | 27.3 (8.1%) | 71.45 (27.62%) |
| Central Sub-Saharan Africa | 118.37 | 75.52 | 42.85 | -8.96 (-7.57%) | -0.59 (-0.78%) | -7.13 (-16.64%) | 165.51 (139.83%) | 93.62 (123.97%) | 71.79 (167.55%) | -38.18 (-32.26%) | -17.52 (-23.19%) | -21.81 (-50.91%) |
| East Asia | 6643.61 | 3098.04 | 3545.57 | 4545 (68.41%) | 2208.69 (71.29%) | 2329.81 (65.71%) | 2195.39 (33.05%) | 1091.12 (35.22%) | 1102.21 (31.09%) | -96.78 (-1.46%) | -201.77 (-6.51%) | 113.54 (3.2%) |
| Eastern Europe | 409.08 | 210.62 | 198.46 | 151.35 (37%) | 72.86 (34.59%) | 87.21 (43.94%) | -12.44 (-3.04%) | -6.96 (-3.3%) | -5.48 (-2.76%) | 270.18 (66.04%) | 144.72 (68.71%) | 116.73 (58.82%) |
| Eastern Sub-Saharan Africa | 729.07 | 470.06 | 259.02 | -62.68 (-8.6%) | -9.09 (-1.93%) | -40.96 (-15.81% | 766.07 (105.07 | 486.07 (103.41 | 279.06 (107.74 | 25.69 (3.52%) | -6.92 (-1.47%) | 20.92 (8.08%) |

|  | Overall difference |  |  | Aging |  |  | Population |  |  | Epidemiological change |  |  |
|---|---|---|---|---|---|---|---|---|---|---|---|---|
| location | Both | Women | Men | Both | Women | Men | Both | Women | Men | Both | Women | Men |
|  |  |  |  |  |  | ) | %) | %) | %) |  |  |  |
| Global | 26249.76 | 12898.66 | 13351.16 | 7911.1 (30.14%) | 3745.16 (29.04%) | 4170.45 (31.24%) | 12436.23 (47.38%) | 6340.5 (49.16%) | 6094.42 (45.65%) | 5902.43 (22.49%) | 2812.94 (21.81%) | 3086.3 (23.12%) |
| High SDI | 6224.84 | 2989.59 | 3235.25 | 2182.93 (35.07%) | 981.87 (32.84%) | 1256.04 (38.82%) | 1687.41 (27.11%) | 790.41 (26.44%) | 897.45 (27.74%) | 2354.5 (37.82%) | 1217.31 (40.72%) | 1081.76 (33.44%) |
| High-income Asia Pacific | 1053.35 | 603.02 | 450.33 | 1318.35 (125.16%) | 693.72 (115.04%) | 641.56 (142.46%) | 320.4 (30.42%) | 160.32 (26.59%) | 160.25 (35.58%) | -585.4 (-55.58%) | -251.01 (-41.63%) | -351.48 (-78.05%) |
| High-income North America | 2557.22 | 1125.17 | 1432.04 | 532.56 (20.83%) | 197.25 (17.53%) | 354.6 (24.76%) | 579.97 (22.68%) | 267.94 (23.81%) | 312.65 (21.83%) | 1444.68 (56.49%) | 659.98 (58.66%) | 764.79 (53.41%) |
| High-middle SDI | 4336.18 | 2033.38 | 2302.8 | 1967.3 (45.37%) | 895.13 (44.02%) | 1105.4 (48%) | 1513.52 (34.9%) | 712.83 (35.06%) | 802.73 (34.86%) | 855.36 (19.73% | 425.42 (20.92% | 394.67 (17.14% |

| | Overall difference | | | Aging | | | Population | | | Epidemiological change | | |
|---|---|---|---|---|---|---|---|---|---|---|---|---|
| location | Both | Women | Men | Both | Women | Men | Both | Women | Men | Both | Women | Men |
| | | | | | | | | | | ) | ) | ) |
| Low SDI | 1757.28 | 1123.94 | 633.34 | -109.7 (-6.24%) | -23.65 (-2.1%) | -74.82 (-11.81%) | 1975.8 (112.43%) | 1163.87 (103.55%) | 814.25 (128.56%) | -108.81 (-6.19%) | -16.28 (-1.45%) | -106.08 (-16.75%) |
| Low-middle SDI | 4896.26 | 2578.25 | 2318.01 | 878.79 (17.95%) | 555.29 (21.54%) | 330.04 (14.24%) | 2694.72 (55.04%) | 1473.79 (57.16%) | 1227.34 (52.95%) | 1322.75 (27.02%) | 549.17 (21.3%) | 760.63 (32.81%) |
| Middle SDI | 9020.42 | 4166.61 | 4853.81 | 4063.99 (45.05%) | 2033.5 (48.8%) | 2014.45 (41.5%) | 4087.2 (45.31%) | 2071.18 (49.71%) | 2015.52 (41.52%) | 869.23 (9.64%) | 61.93 (1.49%) | 823.84 (16.97%) |
| North Africa and Middle East | 2272.2 | 1006.58 | 1265.62 | 346.43 (15.25%) | 186.46 (18.52%) | 158.95 (12.56%) | 1215.61 (53.5%) | 599.38 (59.55%) | 614.93 (48.59%) | 710.17 (31.25%) | 220.74 (21.93%) | 491.74 (38.85%) |
| Oceania | 14.21 | 6.92 | 7.29 | 2.18 (15.32%) | 1 (14.5%) | 1.19 (16.28%) | 14.08 (99.09%) | 6.95 (100.46 | 7.13 (97.83%) | -2.05 (-14.42 | -1.03 (-14.96 | -1.03 (-14.11 |

| | Overall difference | | | Aging | | | Population | | | Epidemiological change | | |
|---|---|---|---|---|---|---|---|---|---|---|---|---|
| location | Both | Women | Men | Both | Women | Men | Both | Women | Men | Both | Women | Men |
| | | | | | | | | %) | | %) | %) | %) |
| South Asia | 4094.41 | 2110.33 | 1984.09 | 800.65 (19.55%) | 440.02 (20.85%) | 353.78 (17.83%) | 2053.14 (50.14%) | 1022.01 (48.43%) | 1028.39 (51.83%) | 1240.62 (30.3%) | 648.29 (30.72%) | 601.92 (30.34%) |
| Southeast Asia | 2782.72 | 1192.98 | 1589.74 | 1024.21 (36.81%) | 504.25 (42.27%) | 517.7 (32.56%) | 1507.67 (54.18%) | 674.73 (56.56%) | 836.77 (52.64%) | 250.85 (9.01%) | 14.01 (1.17%) | 235.27 (14.8%) |
| Southern Latin America | 139.14 | 70.21 | 68.93 | 20.61 (14.81%) | 10.98 (15.64%) | 9.71 (14.08%) | 37.28 (26.79%) | 19.36 (27.58%) | 17.91 (25.98%) | 81.25 (58.4%) | 39.87 (56.79%) | 41.32 (59.94%) |
| Southern Sub-Saharan Africa | 445.37 | 200.61 | 244.76 | 62.29 (13.99%) | 38.02 (18.95%) | 22.58 (9.22%) | 201.51 (45.24%) | 105.09 (52.39%) | 96.63 (39.48%) | 181.58 (40.77%) | 57.49 (28.66%) | 125.56 (51.3%) |
| Tropical Latin America | 308.87 | 166.81 | 142.06 | 115.31 (37.33%) | 71.56 (42.9%) | 43.97 (30.95%) | 136.26 (44.12%) | 81.79 (49.03%) | 54.97 (38.69%) | 57.3 (18.55%) | 13.45 (8.06%) | 43.12 (30.35%) |

| | Overall difference | | | Aging | | | Population | | | Epidemiological change | | |
|---|---|---|---|---|---|---|---|---|---|---|---|---|
| location | Both | Women | Men | Both | Women | Men | Both | Women | Men | Both | Women | Men |
| Western Europe | 1942.26 | 928.04 | 1014.22 | 676.55 (34.83%) | 299.54 (32.28%) | 397.49 (39.19%) | 338.37 (17.42%) | 161.14 (17.36%) | 176.75 (17.43%) | 927.34 (47.75%) | 467.36 (50.36%) | 439.99 (43.38%) |
| Western Sub-Saharan Africa | 1165.02 | 764.43 | 400.59 | -263.72 (-22.64%) | -163.21 (-21.35%) | -104.19 (-26.01%) | 1542.13 (132.37%) | 964.93 (126.23%) | 590.09 (147.31%) | -113.39 (-9.73%) | -37.28 (-4.88%) | -85.31 (-21.3%) |

**Table S8 Changes in NALC deaths according to population-level determinants of population growth, aging, and epidemiological change from 1990 to 2021 in 203 countries**

| | Overll difference | | | Aging | | | Population | | | Epidemiological change | | |
|---|---|---|---|---|---|---|---|---|---|---|---|---|
| location | Both | Women | Men | Both | Women | Men | Both | Women | Men | Both | Women | Men |
| Afghanistan | 27.05 | 24.29 | 2.76 | -40.63 (-150.19%) | -19.71 (-81.15%) | -16.6 (-601.83%) | 57.25 (211.64%) | 36.91 (151.94%) | 18.23 (660.95%) | 10.43 (38.55%) | 7.1 (29.21%) | 1.13 (40.88%) |
| Albania | 16.52 | 5.45 | 11.08 | 27.45 (166.09%) | 13.07 (239.82%) | 14.46 (130.51%) | 0.54 (3.29%) | 0.68 (12.56%) | -0.12 (-1.07%) | -11.46 (-69.37%) | -8.3 (-152.38%) | -3.26 (-29.44%) |
| Algeria | 65.25 | 32.04 | 33.21 | 13.79 (21.14%) | 6.7 (20.9%) | 7.13 (21.48%) | 28.83 (44.19%) | 15.01 (46.84%) | 13.81 (41.58%) | 22.63 (34.67%) | 10.34 (32.26%) | 12.27 (36.94%) |
| American Samoa | 0.33 | 0.18 | 0.14 | 0.14 (43.74%) | 0.08 (43.17%) | 0.06 (44.45%) | 0.05 (14.73%) | 0.03 (14.34%) | 0.02 (15.2%) | 0.14 (41.52%) | 0.08 (42.48%) | 0.06 (40.35%) |
| Andorra | 0.93 | 0.69 | 0.25 | 0.43 (45.76%) | 0.29 (42.42%) | 0.13 (50.5%) | 0.43 (45.65%) | 0.32 (46.06%) | 0.12 (49.43%) | 0.08 (8.59%) | 0.08 (11.52%) | 0 (0.06%) |
| Angola | 42.87 | 22.48 | 20.39 | -0.28 | 0.74 | -1.55 | 60.61 | 27.56 | 32.27 | -17.46 | -5.82 | -10.33 |

| | Overll difference | | | Aging | | | Population | | | Epidemiological change | | |
|---|---|---|---|---|---|---|---|---|---|---|---|---|
| location | Both | Women | Men | Both | Women | Men | Both | Women | Men | Both | Women | Men |
| | | | | (-0.66%) | (3.28%) | (-7.63%) | (141.4%) | (122.61%) | (158.3%) | (-40.74%) | (-25.89%) | (-50.67%) |
| Antigua and Barbuda | 0.14 | 0.06 | 0.08 | 0.01 (9.31%) | 0 (-3.6%) | 0.02 (20.39%) | 0.11 (82.71%) | 0.05 (92.56%) | 0.06 (76.67%) | 0.01 (7.97%) | 0.01 (11.04%) | 0 (2.94%) |
| Argentina | 48.42 | 22.55 | 25.87 | 5.16 (10.66%) | 2.77 (12.29%) | 2.45 (9.45%) | 14.53 (30%) | 7.29 (32.35%) | 7.22 (27.93%) | 28.73 (59.34%) | 12.48 (55.37%) | 16.2 (62.62%) |
| Armenia | 11.64 | 5.61 | 6.03 | 12.14 (104.28%) | 7.33 (130.66%) | 4.61 (76.56%) | 0.25 (2.11%) | 0.42 (7.51%) | -0.09 (-1.47%) | -0.74 (-6.4%) | -2.14 (-38.17%) | 1.5 (24.91%) |
| Australia | 231.15 | 109.95 | 121.2 | 43.81 (18.95%) | 17.96 (16.34%) | 26.85 (22.16%) | 54.22 (23.46%) | 25.99 (23.64%) | 28.19 (23.26%) | 133.12 (57.59%) | 65.99 (60.02%) | 66.16 (54.59%) |
| Austria | 39.24 | 16.35 | 22.89 | 8.48 (21.61%) | 2.96 (18.12%) | 6.6 (28.81%) | 6.39 (16.29%) | 2.6 (15.88%) | 3.81 (16.65%) | 24.37 (62.1%) | 10.79 (66%) | 12.49 (54.54%) |
| Azerbaijan | 49.5 | 24.23 | 25.27 | 8.54 (17.26%) | 3.19 (13.16%) | 5.55 (21.98%) | 23.06 (46.59%) | 12.56 (51.82%) | 10.28 (40.67%) | 17.9 (36.15%) | 8.49 (35.02%) | 9.44 (37.35%) |

| | Overll difference | | | Aging | | | Population | | | Epidemiological change | | |
|---|---|---|---|---|---|---|---|---|---|---|---|---|
| location | Both | Women | Men | Both | Women | Men | Both | Women | Men | Both | Women | Men |
| Bahamas | 0.82 | 0.29 | 0.53 | 0.34 (41.15%) | 0.14 (50.02%) | 0.2 (36.93%) | 0.46 (56.43%) | 0.23 (78.72%) | 0.23 (44.2%) | 0.02 (2.43%) | -0.08 (-28.74%) | 0.1 (18.88%) |
| Bahrain | 3.8 | 1.45 | 2.35 | 1.18 (30.93%) | 0.55 (37.99%) | 0.65 (27.53%) | 3.73 (98.08%) | 1.48 (101.81%) | 2.19 (93.39%) | -1.1 (-29.01%) | -0.58 (-39.8%) | -0.49 (-20.92%) |
| Bangladesh | 201.96 | 94.09 | 107.86 | 63.61 (31.5%) | 26.7 (28.37%) | 36.91 (34.22%) | 105.49 (52.23%) | 46.82 (49.75%) | 56.86 (52.71%) | 32.86 (16.27%) | 20.58 (21.87%) | 14.1 (13.07%) |
| Barbados | 0.89 | 0.56 | 0.33 | 0.26 (29.63%) | 0.12 (22.05%) | 0.13 (38.9%) | 0.32 (35.46%) | 0.2 (35.22%) | 0.12 (35.21%) | 0.31 (34.91%) | 0.24 (42.73%) | 0.09 (25.9%) |
| Belarus | 12.78 | 3.22 | 9.56 | 7.75 (60.66%) | 4.05 (126.04%) | 3.95 (41.36%) | -1.16 (-9.09%) | -0.59 (-18.42%) | -0.57 (-5.93%) | 6.19 (48.43%) | -0.25 (-7.62%) | 6.17 (64.57%) |
| Belgium | 35.79 | 14.62 | 21.17 | 13.18 (36.82%) | 6.17 (42.23%) | 6.89 (32.54%) | 7.15 (19.98%) | 3.91 (26.75%) | 3.16 (14.94%) | 15.46 (43.2%) | 4.54 (31.03%) | 11.11 (52.51%) |

| | Overll difference | | | Aging | | | Population | | | Epidemiological change | | |
|---|---|---|---|---|---|---|---|---|---|---|---|---|
| location | Both | Women | Men | Both | Women | Men | Both | Women | Men | Both | Women | Men |
| Belize | 0.62 | 0.34 | 0.28 | 0.02 (3.33%) | 0 (-0.81%) | 0.02 (6.32%) | 0.43 (68.42%) | 0.27 (78.54%) | 0.16 (56.86%) | 0.18 (28.25%) | 0.08 (22.27%) | 0.1 (36.82%) |
| Benin | 45.76 | 29.6 | 16.16 | -12.25 (-26.77%) | -3.76 (-12.69%) | -7.99 (-49.46%) | 70.43 (153.91%) | 36.55 (123.48%) | 33.83 (209.34%) | -12.42 (-27.14%) | -3.2 (-10.79%) | -9.68 (-59.88%) |
| Bermuda | 0.07 | 0.01 | 0.06 | 0.18 (247.19%) | 0.1 (650.83%) | 0.08 (140.28%) | 0.04 (52.37%) | 0.02 (142.73%) | 0.02 (28.65%) | -0.14 (-199.56%) | -0.1 (-693.56%) | -0.04 (-68.93%) |
| Bhutan | 2.08 | 0.92 | 1.16 | 0.77 (37.34%) | 0.31 (33.45%) | 0.47 (40.89%) | 0.67 (32.12%) | 0.3 (32.99%) | 0.36 (31.15%) | 0.63 (30.54%) | 0.31 (33.56%) | 0.32 (27.96%) |
| Bolivia (Plurinational State of) | 29.84 | 22.68 | 7.16 | 6.22 (20.84%) | 5.11 (22.51%) | 1.25 (17.41%) | 17.57 (58.87%) | 13.55 (59.74%) | 3.76 (52.52%) | 6.05 (20.29%) | 4.03 (17.75%) | 2.15 (30.07%) |
| Bosnia and Herzegovina | 21.91 | 12.7 | 9.21 | 23.86 (108.9%) | 13.48 (106.12%) | 10.37 (112.6%) | -6.66 (-30.38%) | -3.37 (-26.54%) | -3.18 (-34.54%) | 4.71 (21.48%) | 2.59 (20.42%) | 2.02 (21.94%) |

| | Overll difference | | | Aging | | | Population | | | Epidemiological change | | |
|---|---|---|---|---|---|---|---|---|---|---|---|---|
| location | Both | Women | Men | Both | Women | Men | Both | Women | Men | Both | Women | Men |
| Botswana | 8.1 | 3.63 | 4.46 | 1.02 (12.6%) | 0.8 (21.9%) | 0.22 (4.98%) | 5.38 (66.41%) | 2.77 (76.33%) | 2.59 (58.09%) | 1.7 (20.99%) | 0.06 (1.77%) | 1.65 (36.93%) |
| Brazil | 297.69 | 160.8 | 136.89 | 113.38 (38.09%) | 70.27 (43.7%) | 43.3 (31.63%) | 130.91 (43.98%) | 78.59 (48.87%) | 52.83 (38.59%) | 53.4 (17.94%) | 11.95 (7.43%) | 40.76 (29.77%) |
| Brunei Darussalam | 1.33 | 0.72 | 0.6 | 0.7 (52.74%) | 0.37 (51.43%) | 0.33 (55.27%) | 1.04 (78.5%) | 0.54 (74.82%) | 0.5 (83.17%) | -0.41 (-31.24%) | -0.19 (-26.25%) | -0.23 (-38.44%) |
| Bulgaria | -39.09 | -25.08 | -14.01 | 29.82 (-76.27%) | 23.61 (-94.13%) | 7.71 (-55.02%) | -12.16 (31.1%) | -6.88 (27.42%) | -5.13 (36.59%) | -56.75 (145.17%) | -41.81 (166.71%) | -16.6 (118.43%) |
| Burkina Faso | 82.3 | 53.5 | 28.8 | -22.69 (-27.57%) | -10.08 (-18.84%) | -12.38 (-42.98%) | 127.85 (155.34%) | 62.78 (117.34%) | 65.25 (226.57%) | -22.86 (-27.77%) | 0.8 (1.5%) | -24.07 (-83.59%) |
| Burundi | 5.29 | 3.75 | 1.54 | -2.97 (-56.02%) | -3.18 (-84.85% | -0.33 (-21.51% | 15.35 (289.86% | 11 (293.27% | 3.78 (245%) | -7.09 (-133.84 | -4.07 (-108.42 | -1.91 (-123.49 |

| | Overll difference | | | Aging | | | Population | | | Epidemiological change | | |
|---|---|---|---|---|---|---|---|---|---|---|---|---|
| location | Both | Women | Men | Both | Women | Men | Both | Women | Men | Both | Women | Men |
| | | | | | ) | ) | ) | ) | | %) | %) | %) |
| Cabo Verde | 5.68 | 3.21 | 2.47 | -1.2 (-21.19%) | -0.28 (-8.7%) | -0.68 (-27.56%) | 4.13 (72.64%) | 2.36 (73.32%) | 1.61 (65.21%) | 2.76 (48.54%) | 1.14 (35.38%) | 1.54 (62.35%) |
| Cambodia | 43.23 | 28.64 | 14.59 | 14.72 (34.06%) | 12.28 (42.88%) | 3.45 (23.62%) | 38.35 (88.72%) | 23.54 (82.19%) | 13.99 (95.91%) | -9.85 (-22.78%) | -7.18 (-25.07%) | -2.85 (-19.54%) |
| Cameroon | 119.03 | 69.08 | 49.95 | -22.09 (-18.55%) | -9.17 (-13.27%) | -11.97 (-23.96%) | 181.92 (152.84%) | 101.88 (147.48%) | 79.47 (159.1%) | -40.81 (-34.29%) | -23.63 (-34.21%) | -17.55 (-35.14%) |
| Canada | 418.12 | 223.5 | 194.62 | 111.01 (26.55%) | 54.09 (24.2%) | 56.8 (29.18%) | 91.56 (21.9%) | 49.61 (22.2%) | 41.9 (21.53%) | 215.55 (51.55%) | 119.8 (53.6%) | 95.93 (49.29%) |
| Central African Republic | 2.63 | 1.81 | 0.82 | -0.76 (-28.92%) | -0.3 (-16.45%) | -0.47 (-56.81%) | 8.15 (309.23%) | 3.71 (204.73%) | 4.44 (538.05%) | -4.75 (-180.31%) | -1.6 (-88.28%) | -3.14 (-381.24%) |

| | Overll difference | | | Aging | | | Population | | | Epidemiological change | | |
|---|---|---|---|---|---|---|---|---|---|---|---|---|
| location | Both | Women | Men | Both | Women | Men | Both | Women | Men | Both | Women | Men |
| Chad | 36.65 | 21.86 | 14.79 | -21.64 (-59.03%) | -13.78 (-63.03%) | -8.35 (-56.44%) | 60.76 (165.77%) | 29.1 (133.11%) | 32.1 (217.04%) | -2.47 (-6.74%) | 6.54 (29.92%) | -8.96 (-60.6%) |
| Chile | 80.98 | 44.27 | 36.72 | 22.27 (27.5%) | 12.51 (28.26%) | 9.82 (26.75%) | 21.59 (26.66%) | 11.74 (26.53%) | 9.83 (26.76%) | 37.12 (45.83%) | 20.01 (45.21%) | 17.07 (46.49%) |
| China | 6281.15 | 2899.84 | 3381.31 | 4379.44 (69.72%) | 2119.31 (73.08%) | 2253.76 (66.65%) | 2087.99 (33.24%) | 1038.43 (35.81%) | 1047.43 (30.98%) | -186.29 (-2.97%) | -257.9 (-8.89%) | 80.11 (2.37%) |
| Colombia | 116.85 | 69.28 | 47.57 | 55.68 (47.65%) | 39.95 (57.66%) | 17.17 (36.09%) | 55.99 (47.92%) | 37.17 (53.65%) | 19.19 (40.34%) | 5.18 (4.43%) | -7.84 (-11.31%) | 11.21 (23.56%) |
| Comoros | 2.29 | 1.75 | 0.54 | 0.63 (27.66%) | 0.59 (33.44%) | 0.12 (22.63%) | 1.83 (79.83%) | 1.31 (74.53%) | 0.51 (94.43%) | -0.17 (-7.49%) | -0.14 (-7.97%) | -0.09 (-17.06%) |
| Congo | 8.14 | 5.87 | 2.27 | 0.28 (3.49%) | -0.34 (-5.83%) | 0.57 (24.92%) | 14.26 (175.07% | 8.3 (141.45% | 5.92 (260.44 | -6.4 (-78.56% | -2.09 (-35.62% | -4.21 (-185.35 |

| | Overll difference | | | Aging | | | Population | | | Epidemiological change | | |
|---|---|---|---|---|---|---|---|---|---|---|---|---|
| location | Both | Women | Men | Both | Women | Men | Both | Women | Men | Both | Women | Men |
| | | | | | | | ) | ) | %) | ) | ) | %) |
| Cook Islands | 0.21 | 0.08 | 0.13 | 0.15 (70.09%) | 0.07 (86.53%) | 0.08 (59.22%) | 0.03 (13.99%) | 0.02 (28.49%) | 0 (3.9%) | 0.03 (15.93%) | -0.01 (-15.02%) | 0.05 (36.88%) |
| Costa Rica | 30.81 | 15.88 | 14.93 | 9.43 (30.6%) | 5.24 (32.98%) | 4.19 (28.04%) | 12.38 (40.2%) | 6.95 (43.76%) | 5.47 (36.66%) | 8.99 (29.2%) | 3.69 (23.26%) | 5.27 (35.3%) |
| Croatia | 16.21 | 6.19 | 10.02 | 14.53 (89.68%) | 8.76 (141.63%) | 5.62 (56.06%) | -1.78 (-10.98%) | -1.13 (-18.21%) | -0.65 (-6.51%) | 3.45 (21.3%) | -1.45 (-23.42%) | 5.06 (50.45%) |
| Cuba | 15.26 | 8.03 | 7.23 | 16.32 (106.99%) | 11.17 (139.23%) | 5.54 (76.58%) | 3.99 (26.18%) | 2.75 (34.23%) | 1.36 (18.81%) | -5.06 (-33.17%) | -5.9 (-73.46%) | 0.33 (4.61%) |
| Cyprus | 3.07 | 1.39 | 1.67 | 1.14 (37.15%) | 0.54 (38.93%) | 0.6 (35.92%) | 1.96 (64.07%) | 1.01 (72.55%) | 0.95 (56.85%) | -0.04 (-1.21%) | -0.16 (-11.48%) | 0.12 (7.23%) |

| | Overll difference | | | Aging | | | Population | | | Epidemiological change | | |
|---|---|---|---|---|---|---|---|---|---|---|---|---|
| location | Both | Women | Men | Both | Women | Men | Both | Women | Men | Both | Women | Men |
| Czechia | -1.35 | -5.78 | 4.43 | 20.41 (-1511.92%) | 10.63 (-184.04%) | 10 (225.94%) | 5.24 (-388.48%) | 2.37 (-40.95%) | 2.74 (61.79%) | -27.01 (2000.4%) | -18.77 (324.99%) | -8.31 (-187.73%) |
| Democratic People's Republic of Korea | 58.41 | 40.22 | 18.18 | 39.86 (68.24%) | 26.17 (65.06%) | 15.17 (83.41%) | 45.95 (78.67%) | 21.53 (53.52%) | 23.76 (130.7%) | -27.4 (-46.91%) | -7.47 (-18.58%) | -20.75 (-114.11%) |
| Democratic Republic of the Congo | 55.96 | 41.3 | 14.66 | -4.93 (-8.8%) | 0.21 (0.52%) | -3.42 (-23.3%) | 77.76 (138.97%) | 50.5 (122.3%) | 26.31 (179.43%) | -16.88 (-30.16%) | -9.42 (-22.82%) | -8.23 (-56.12%) |
| Denmark | 17.21 | 7.25 | 9.96 | 3.64 (21.15%) | 1.35 (18.59%) | 2.42 (24.27%) | 2.08 (12.11%) | 0.96 (13.3%) | 1.11 (11.17%) | 11.49 (66.74%) | 4.94 (68.12%) | 6.43 (64.56%) |
| Djibouti | 3.38 | 2.01 | 1.37 | 0.61 (17.95%) | 0.33 (16.41%) | 0.25 (18.25%) | 2.24 (66.24%) | 1.39 (69.24%) | 0.83 (60.6%) | 0.54 (15.82%) | 0.29 (14.35%) | 0.29 (21.15%) |
| Dominica | 0.21 | 0.09 | 0.12 | 0.04 (21.27%) | 0.02 (18.37%) | 0.03 (27%) | 0.03 (14.01%) | 0.01 (10.12%) | 0.02 (16.73%) | 0.13 (64.72%) | 0.07 (71.52%) | 0.07 (56.27%) |

| | Overll difference | | | Aging | | | Population | | | Epidemiological change | | |
|---|---|---|---|---|---|---|---|---|---|---|---|---|
| location | Both | Women | Men | Both | Women | Men | Both | Women | Men | Both | Women | Men |
| Dominican Republic | 15.02 | 7.9 | 7.12 | 4.21 (28.06%) | 2.84 (35.96%) | 1.47 (20.68%) | 5.84 (38.9%) | 3.15 (39.89%) | 2.66 (37.37%) | 4.96 (33.05%) | 1.91 (24.15%) | 2.99 (41.95%) |
| Ecuador | 48.51 | 36.19 | 12.32 | 18.74 (38.62%) | 15.13 (41.81%) | 4.26 (34.54%) | 32.89 (67.8%) | 24.39 (67.39%) | 8.49 (68.92%) | -3.12 (-6.42%) | -3.33 (-9.2%) | -0.43 (-3.45%) |
| Egypt | 1115.93 | 465.1 | 650.83 | 106.05 (9.5%) | 30.45 (6.55%) | 66.72 (10.25%) | 528.91 (47.4%) | 261.99 (56.33%) | 266.78 (40.99%) | 480.96 (43.1%) | 172.66 (37.12%) | 317.33 (48.76%) |
| El Salvador | 7.29 | 4.56 | 2.73 | 3.09 (42.34%) | 2.17 (47.6%) | 0.96 (35.01%) | 3.22 (44.19%) | 2.29 (50.29%) | 1.01 (37.1%) | 0.98 (13.48%) | 0.1 (2.11%) | 0.76 (27.89%) |
| Equatorial Guinea | 2.71 | 1.42 | 1.29 | -0.75 (-27.6%) | -0.23 (-16.24%) | -0.48 (-37.18%) | 2.27 (83.75%) | 1.24 (86.88%) | 0.97 (74.96%) | 1.19 (43.86%) | 0.42 (29.36%) | 0.8 (62.22%) |
| Eritrea | 9.1 | 7.52 | 1.58 | 1.4 (15.38%) | 1.46 (19.36%) | 0.13 (8.46%) | 7.39 (81.19%) | 5.36 (71.33%) | 1.84 (116.32%) | 0.31 (3.43%) | 0.7 (9.31%) | -0.39 (-24.78%) |
| Estonia | 5.25 | 2.84 | 2.41 | 3.17 | 1.93 | 1.33 | -0.84 | -0.58 | -0.28 | 2.92 | 1.49 | 1.37 |

| | Overll difference | | | Aging | | | Population | | | Epidemiological change | | |
|---|---|---|---|---|---|---|---|---|---|---|---|---|
| location | Both | Women | Men | Both | Women | Men | Both | Women | Men | Both | Women | Men |
| | | | | (60.43%) | (67.84%) | (55.03%) | (-16.09%) | (-20.31%) | (-11.69%) | (55.66%) | (52.47%) | (56.66%) |
| Eswatini | 11.41 | 2.62 | 8.8 | 0.73 (6.36%) | 0.53 (20.09%) | -0.16 (-1.81%) | 4.44 (38.87%) | 1.61 (61.59%) | 2.96 (33.62%) | 6.25 (54.77%) | 0.48 (18.32%) | 6 (68.19%) |
| Ethiopia | 61.77 | 48.62 | 13.15 | -5.77 (-9.35%) | 0.39 (0.8%) | -3.56 (-27.08%) | 103.32 (167.28%) | 77.25 (158.88%) | 24.61 (187.19%) | -35.78 (-57.93%) | -29.02 (-59.68%) | -7.9 (-60.11%) |
| Fiji | 2.9 | 1.47 | 1.43 | 1.18 (40.72%) | 0.66 (44.68%) | 0.52 (36.51%) | 0.81 (27.93%) | 0.42 (28.79%) | 0.39 (27.09%) | 0.91 (31.35%) | 0.39 (26.53%) | 0.52 (36.4%) |
| Finland | 27.5 | 12.61 | 14.9 | 12.66 (46.04%) | 5.79 (45.95%) | 7.14 (47.91%) | 4.08 (14.85%) | 1.94 (15.38%) | 2.06 (13.85%) | 10.76 (39.11%) | 4.87 (38.66%) | 5.7 (38.24%) |
| France | 401.13 | 197.84 | 203.29 | 150.65 (37.56%) | 62.61 (31.65%) | 96.64 (47.54%) | 71.48 (17.82%) | 31.9 (16.12%) | 39.33 (19.35%) | 179 (44.62%) | 103.33 (52.23%) | 67.31 (33.11%) |
| Gabon | 6.05 | 2.64 | 3.41 | -0.8 (-13.28%) | -0.71 (-26.71% | -0.05 (-1.33%) | 5.09 (84.14%) | 2.79 (105.48% | 2.31 (67.76%) | 1.76 (29.14%) | 0.56 (21.23%) | 1.14 (33.57%) |

| | Overll difference | | | Aging | | | Population | | | Epidemiological change | | |
|---|---|---|---|---|---|---|---|---|---|---|---|---|
| location | Both | Women | Men | Both | Women | Men | Both | Women | Men | Both | Women | Men |
| | | | | | ) | | | ) | | | | |
| Gambia | 29.11 | 13.63 | 15.48 | 1.28 (4.4%) | 1.12 (8.19%) | 0.05 (0.34%) | 19.75 (67.83%) | 9.02 (66.16%) | 10.66 (68.86%) | 8.08 (27.77%) | 3.5 (25.65%) | 4.77 (30.8%) |
| Georgia | -2.64 | -3.09 | 0.45 | 7.49 (-283.91 %) | 4.06 (-131.43 %) | 3.53 (785.3%) | -8.38 (317.98% ) | -4.58 (148.38% ) | -3.8 (-844.15 %) | -1.74 (65.93%) | -2.56 (83.05%) | 0.71 (158.85 %) |
| Germany | 420.3 7 | 176.82 | 243.55 | 153.75 (36.57%) | 62.34 (35.26%) | 97.55 (40.05%) | 40.7 (9.68%) | 13.6 (7.69%) | 25.54 (10.48%) | 225.93 (53.74%) | 100.88 (57.05%) | 120.46 (49.46%) |
| Ghana | 105.5 8 | 51.14 | 54.44 | 6.64 (6.29%) | 7.55 (14.75%) | -1.01 (-1.86%) | 108.28 (102.55% ) | 61.03 (119.33% ) | 47.74 (87.7%) | -9.33 (-8.84%) | -17.43 (-34.08% ) | 7.71 (14.16%) |
| Greece | 58.58 | 31 | 27.58 | 25.39 (43.33%) | 15.94 (51.43%) | 9.17 (33.25%) | 2.36 (4.03%) | 1.79 (5.77%) | 0.68 (2.47%) | 30.84 (52.64%) | 13.27 (42.8%) | 17.73 (64.28%) |
| Greenland | 0.3 | 0.15 | 0.15 | 0.23 (77.32%) | 0.09 (57.1%) | 0.14 (97.33%) | 0.02 (7.93%) | 0.02 (12.95%) | 0 (2.99%) | 0.04 (14.75%) | 0.05 (29.95%) | 0 (-0.32%) |

| | Overll difference | | | Aging | | | Population | | | Epidemiological change | | |
|---|---|---|---|---|---|---|---|---|---|---|---|---|
| location | Both | Women | Men | Both | Women | Men | Both | Women | Men | Both | Women | Men |
| Grenada | 0.29 | 0.13 | 0.16 | -0.03 (-11.04%) | -0.02 (-12.15%) | -0.13 (-80.53%) | 0.12 (39.98%) | 0.06 (44.61%) | 0.07 (45.29%) | 0.21 (71.06%) | 0.08 (67.54%) | 0.22 (135.24%) |
| Guam | 1.04 | 0.33 | 0.71 | 0.47 (45.74%) | 0.18 (55.73%) | 0.3 (41.7%) | 0.17 (16.22%) | 0.08 (24.74%) | 0.08 (10.78%) | 0.39 (38.03%) | 0.06 (19.54%) | 0.34 (47.52%) |
| Guatemala | 40 | 24.17 | 15.84 | 11.58 (28.96%) | 7.65 (31.64%) | 4.18 (26.42%) | 33.31 (83.26%) | 20.48 (84.76%) | 12.96 (81.85%) | -4.89 (-12.22%) | -3.96 (-16.4%) | -1.31 (-8.28%) |
| Guinea | 50.27 | 31.38 | 18.89 | -31.65 (-62.95%) | -19.5 (-62.13%) | -12.57 (-66.56%) | 89.41 (177.84%) | 53.5 (170.47%) | 35.99 (190.51%) | -7.49 (-14.89%) | -2.62 (-8.34%) | -4.52 (-23.95%) |
| Guinea-Bissau | 3.87 | 3.23 | 0.64 | -2.52 (-65.13%) | -0.76 (-23.4%) | -1.8 (-280.8%) | 10.93 (282.08%) | 4.82 (149.09%) | 6.11 (952.9%) | -4.53 (-116.95%) | -0.83 (-25.69%) | -3.67 (-572.1%) |
| Guyana | 0.69 | 0.35 | 0.34 | 0.44 | 0.29 | 0.16 | 0.15 | 0.1 | 0.05 | 0.1 | -0.04 | 0.13 |

| | Overll difference | | | Aging | | | Population | | | Epidemiological change | | |
|---|---|---|---|---|---|---|---|---|---|---|---|---|
| location | Both | Women | Men | Both | Women | Men | Both | Women | Men | Both | Women | Men |
| | | | | (64.67%) | (82.99%) | (47.48%) | (21.22%) | (27.67%) | (15.22%) | (14.11%) | (-10.66%) | (37.29%) |
| Haiti | 4.35 | 3.2 | 1.15 | -0.21 (-4.87%) | -0.1 (-3.22%) | -0.09 (-7.65%) | 5.43 (124.72%) | 4.06 (126.75%) | 1.35 (117.57%) | -0.86 (-19.86%) | -0.75 (-23.53%) | -0.11 (-9.91%) |
| Honduras | 21.77 | 15.31 | 6.46 | 1.72 (7.92%) | 1.37 (8.93%) | 0.46 (7.1%) | 11.15 (51.22%) | 8.02 (52.39%) | 3.22 (49.81%) | 8.9 (40.86%) | 5.92 (38.69%) | 2.78 (43.09%) |
| Hungary | -3.49 | -5.57 | 2.07 | 12.36 (-353.74%) | 8.11 (-145.59%) | 4.25 (204.98%) | -0.25 (7.21%) | -0.07 (1.26%) | -0.16 (-7.58%) | -15.6 (446.52%) | -13.61 (244.33%) | -2.02 (-97.4%) |
| Iceland | 1.45 | 0.59 | 0.85 | 0.35 (24.3%) | 0.14 (24.1%) | 0.22 (25.34%) | 0.44 (30.38%) | 0.2 (32.88%) | 0.24 (28.68%) | 0.66 (45.32%) | 0.26 (43.02%) | 0.39 (45.98%) |
| India | 3571.74 | 1825.22 | 1746.52 | 773.03 (21.64%) | 414.75 (22.72%) | 347.85 (19.92%) | 1712.98 (47.96%) | 832.3 (45.6%) | 877.63 (50.25%) | 1085.73 (30.4%) | 578.18 (31.68%) | 521.04 (29.83%) |
| Indonesia | 770.7 | 322.63 | 448.16 | 196.88 | 95.42 | 101.64 | 361.89 | 161.97 | 200.78 | 212.02 | 65.24 | 145.74 |

| | Overll difference | | | Aging | | | Population | | | Epidemiological change | | |
|---|---|---|---|---|---|---|---|---|---|---|---|---|
| location | Both | Women | Men | Both | Women | Men | Both | Women | Men | Both | Women | Men |
| | 9 | | | (25.54%) | (29.57%) | (22.68%) | (46.95%) | (50.2%) | (44.8%) | (27.51%) | (20.22%) | (32.52%) |
| Iran (Islamic Republic of) | 278.93 | 154.13 | 124.8 | 81.55 (29.24%) | 48.93 (31.75%) | 31.72 (25.42%) | 112.41 (40.3%) | 65.15 (42.27%) | 47.3 (37.9%) | 84.97 (30.46%) | 40.04 (25.98%) | 45.78 (36.68%) |
| Iraq | 103.09 | 51.2 | 51.89 | 4.73 (4.59%) | 2.79 (5.45%) | 1.85 (3.56%) | 72.12 (69.96%) | 37.55 (73.33%) | 34.54 (66.56%) | 26.24 (25.45%) | 10.87 (21.23%) | 15.5 (29.87%) |
| Ireland | 16.18 | 8.66 | 7.52 | 3.22 (19.91%) | 1.42 (16.45%) | 1.66 (22.11%) | 5.02 (31.04%) | 3 (34.62%) | 2.03 (27.03%) | 7.94 (49.05%) | 4.24 (48.93%) | 3.83 (50.86%) |
| Israel | 17.7 | 8.63 | 9.07 | 4.18 (23.61%) | 2.65 (30.69%) | 1.64 (18.1%) | 11.84 (66.93%) | 6.33 (73.37%) | 5.51 (60.76%) | 1.67 (9.46%) | -0.35 (-4.06%) | 1.92 (21.14%) |
| Italy | 49.88 | 18.91 | 30.97 | 119.32 (239.2%) | 59.05 (312.22%) | 62.78 (202.73%) | 27.33 (54.78%) | 12.45 (65.84%) | 14.98 (48.38%) | -96.76 (-193.98%) | -52.59 (-278.06%) | -46.8 (-151.11%) |
| Jamaica | 4.17 | 2.36 | 1.8 | 0.61 (14.74%) | 0.37 (15.73%) | 0.25 (13.65%) | 1.43 (34.44%) | 0.83 (35.15%) | 0.59 (32.72%) | 2.12 (50.82%) | 1.16 (49.12%) | 0.97 (53.63%) |
| Japan | 632.7 | 409.39 | 223.37 | 782.17 | 412.8 | 384.09 | 99.09 | 50.77 | 47.87 | -248.5 | -54.18 | -208.6 |

| | Overll difference | | | Aging | | | Population | | | Epidemiological change | | |
|---|---|---|---|---|---|---|---|---|---|---|---|---|
| location | Both | Women | Men | Both | Women | Men | Both | Women | Men | Both | Women | Men |
| | 6 | | | (123.61%) | (100.83%) | (171.95%) | (15.66%) | (12.4%) | (21.43%) | (-39.27%) | (-13.23%) | (-93.38%) |
| Jordan | 12.74 | 6.11 | 6.63 | 2.79 (21.87%) | 1.37 (22.42%) | 1.42 (21.36%) | 10.22 (80.21%) | 5.17 (84.52%) | 5.02 (75.74%) | -0.26 (-2.08%) | -0.42 (-6.95%) | 0.19 (2.9%) |
| Kazakhstan | -9.39 | 1.57 | -10.96 | 13.88 (-147.73%) | 4.53 (288.56%) | 10.33 (-94.26%) | 16.85 (-179.34%) | 9.25 (589.49%) | 7.65 (-69.82%) | -40.12 (427.07%) | -12.21 (-778.05%) | -28.95 (264.08%) |
| Kenya | 136.39 | 83.2 | 53.19 | 4.76 (3.49%) | 6 (7.21%) | 0.6 (1.12%) | 79.41 (58.22%) | 51.77 (62.22%) | 27.56 (51.82%) | 52.22 (38.29%) | 25.43 (30.57%) | 25.03 (47.05%) |
| Kiribati | 0.27 | 0.21 | 0.06 | 0.04 (16.28%) | 0.03 (16.13%) | 0.01 (17.87%) | 0.21 (77.31%) | 0.15 (74.02%) | 0.06 (91.76%) | 0.02 (6.41%) | 0.02 (9.85%) | -0.01 (-9.63%) |
| Kuwait | 2.31 | 1.09 | 1.22 | 1.88 (81.1%) | 0.55 (50.74%) | 1.34 (109.73%) | 5.06 (218.81%) | 2.25 (206.33%) | 2.75 (225.59%) | -4.62 (-199.91%) | -1.72 (-157.07%) | -2.87 (-235.31%) |
| Kyrgyzstan | -0.4 | -1.36 | 0.96 | -0.59 | -0.95 | 0.39 | 6.79 | 3.75 | 3.01 | -6.6 | -4.15 | -2.45 |

| | Overll difference | | | Aging | | | Population | | | Epidemiological change | | |
|---|---|---|---|---|---|---|---|---|---|---|---|---|
| location | Both | Women | Men | Both | Women | Men | Both | Women | Men | Both | Women | Men |
| | | | | (147.12%) | (70.09%) | (40.79%) | (-1698.72%) | (-275.95%) | (314.73%) | (1651.6%) | (305.85%) | (-255.52%) |
| Lao People's Democratic Republic | 10.48 | 5.14 | 5.34 | 0.96 (9.19%) | 0.74 (14.37%) | 0.2 (3.7%) | 16.31 (155.59%) | 7.28 (141.73%) | 9.11 (170.46%) | -6.79 (-64.78%) | -2.88 (-56.1%) | -3.96 (-74.16%) |
| Latvia | 4.12 | 2.57 | 1.55 | 3.93 (95.32%) | 2.41 (93.91%) | 1.59 (102.24%) | -2.83 (-68.63%) | -1.68 (-65.4%) | -1.14 (-73.6%) | 3.02 (73.31%) | 1.84 (71.49%) | 1.11 (71.36%) |
| Lebanon | 8.79 | 4.86 | 3.93 | 2.8 (31.88%) | 2.04 (41.93%) | 0.76 (19.26%) | 6.99 (79.59%) | 4.05 (83.27%) | 2.92 (74.44%) | -1.01 (-11.47%) | -1.23 (-25.21%) | 0.25 (6.29%) |
| Lesotho | 15.68 | 5.91 | 9.77 | -2.12 (-13.54%) | -0.67 (-11.28%) | -1.59 (-16.29%) | 4.84 (30.89%) | 1.71 (28.97%) | 3.29 (33.69%) | 12.96 (82.65%) | 4.87 (82.31%) | 8.07 (82.59%) |
| Liberia | 19.94 | 11.27 | 8.66 | -12.19 | -4.89 | -7.17 | 33.36 | 14.98 | 18.39 | -1.23 | 1.18 | -2.56 |

| | Overll difference | | | Aging | | | Population | | | Epidemiological change | | |
|---|---|---|---|---|---|---|---|---|---|---|---|---|
| location | Both | Women | Men | Both | Women | Men | Both | Women | Men | Both | Women | Men |
| | | | | (-61.15%) | (-43.35%) | (-82.74%) | (167.33%) | (132.9%) | (212.28%) | (-6.18%) | (10.45%) | (-29.53%) |
| Libya | 38.73 | 21.18 | 17.55 | 5.15 (13.3%) | 2.86 (13.5%) | 2.28 (12.97%) | 19.79 (51.1%) | 12.14 (57.31%) | 7.92 (45.11%) | 13.79 (35.6%) | 6.18 (29.19%) | 7.36 (41.92%) |
| Lithuania | 9.18 | 4.27 | 4.91 | 5.86 (63.87%) | 3.71 (86.89%) | 2.22 (45.28%) | -2.63 (-28.6%) | -1.37 (-32.13%) | -1.23 (-25.16%) | 5.94 (64.73%) | 1.93 (45.24%) | 3.92 (79.88%) |
| Luxembourg | 1.96 | 0.98 | 0.98 | 0.26 (13.28%) | 0.06 (6.41%) | 0.21 (20.99%) | 1.09 (55.49%) | 0.59 (60.19%) | 0.49 (49.74%) | 0.61 (31.24%) | 0.33 (33.4%) | 0.29 (29.27%) |
| Madagascar | 23.68 | 18.83 | 4.84 | -5.68 (-24%) | -3.25 (-17.28%) | -1.85 (-38.28%) | 31.06 (131.2%) | 22.63 (120.17%) | 8.58 (177.11%) | -1.7 (-7.2%) | -0.54 (-2.89%) | -1.88 (-38.83%) |
| Malawi | 32.6 | 15.93 | 16.67 | -3.1 (-9.5%) | -0.98 (-6.16%) | -1.97 (-11.84%) | 24.09 (73.91%) | 13.63 (85.55%) | 10.51 (63.08%) | 11.6 (35.59%) | 3.28 (20.61%) | 8.13 (48.76%) |

| | Overll difference | | | Aging | | | Population | | | Epidemiological change | | |
|---|---|---|---|---|---|---|---|---|---|---|---|---|
| location | Both | Women | Men | Both | Women | Men | Both | Women | Men | Both | Women | Men |
| Malaysia | 146.01 | 58.93 | 87.08 | 33.43 (22.89%) | 15.61 (26.49%) | 17.81 (20.45%) | 69.25 (47.43%) | 30.29 (51.39%) | 39.09 (44.89%) | 43.33 (29.68%) | 13.04 (22.12%) | 30.18 (34.66%) |
| Maldives | 1 | 0.18 | 0.82 | 0.14 (14.48%) | 0.1 (54.02%) | -0.13 (-16.43%) | 1.07 (107.15%) | 0.16 (87.29%) | 1.04 (127.45%) | -0.22 (-21.63%) | -0.08 (-41.3%) | -0.09 (-11.02%) |
| Mali | 144.6 | 57.68 | 86.92 | -31.93 (-22.08%) | -13.39 (-23.22%) | -18.66 (-21.47%) | 154.73 (107.01%) | 64.32 (111.51%) | 91 (104.7%) | 21.8 (15.08%) | 6.75 (11.7%) | 14.58 (16.77%) |
| Malta | 1.49 | 0.54 | 0.95 | 0.7 (46.83%) | 0.32 (57.97%) | 0.4 (41.67%) | 0.35 (23.41%) | 0.15 (26.93%) | 0.21 (21.6%) | 0.44 (29.76%) | 0.08 (15.11%) | 0.35 (36.74%) |
| Marshall Islands | 0.09 | 0.05 | 0.04 | 0.02 (23.21%) | 0.01 (20.06%) | 0.01 (26.2%) | 0.04 (47.58%) | 0.03 (51.65%) | 0.02 (42.43%) | 0.03 (29.21%) | 0.01 (28.29%) | 0.01 (31.38%) |
| Mauritania | 21.99 | 12.41 | 9.58 | -2.12 (-9.63%) | -3.08 (-24.78%) | 1.15 (12.01%) | 43 (195.54%) | 20.64 (166.25%) | 22.39 (233.71%) | -18.89 (-85.91%) | -5.15 (-41.47%) | -13.96 (-145.72%) |

| | Overll difference | | | Aging | | | Population | | | Epidemiological change | | |
|---|---|---|---|---|---|---|---|---|---|---|---|---|
| location | Both | Women | Men | Both | Women | Men | Both | Women | Men | Both | Women | Men |
| Mauritius | -1.43 | -1.03 | -0.41 | 2.03 (-142.22 %) | 1.19 (-116.1% ) | 0.81 (-199.61 %) | 1 (-70.22%) | 0.64 (-62.02%) | 0.37 (-92.32% ) | -4.47 (312.44 %) | -2.85 (278.12% ) | -1.59 (391.93 %) |
| Mexico | 318.9 7 | 178.03 | 140.94 | 90.67 (28.43%) | 56.69 (31.84%) | 35.42 (25.13%) | 116 (36.37%) | 67.71 (38.03%) | 48.62 (34.5%) | 112.3 (35.21%) | 53.63 (30.13%) | 56.89 (40.37%) |
| Micronesia (Federated States of) | 0.2 | 0.1 | 0.1 | 0.06 (31.06%) | 0.03 (33.71%) | 0.03 (28.42%) | 0.07 (35.19%) | 0.04 (37.51%) | 0.03 (32.79%) | 0.07 (33.75%) | 0.03 (28.78%) | 0.04 (38.8%) |
| Monaco | 0.48 | 0.22 | 0.26 | 0.05 (11.03%) | 0.01 (6.06%) | 0.04 (16.82%) | 0.09 (18.42%) | 0.04 (17.19%) | 0.05 (19.73%) | 0.34 (70.55%) | 0.17 (76.75%) | 0.17 (63.46%) |
| Mongolia | 83.55 | 56.44 | 27.1 | 11.11 (13.3%) | 6.05 (10.72%) | 4.75 (17.54%) | 39.33 (47.07%) | 24.28 (43.02%) | 15.24 (56.23%) | 33.11 (39.63%) | 26.11 (46.26%) | 7.11 (26.23%) |
| Montenegro | 4.15 | 2.37 | 1.77 | 1.98 (47.64%) | 1.14 (47.97%) | 0.83 (46.89%) | 0.46 (11.19%) | 0.31 (13.08%) | 0.16 (9.18%) | 1.71 (41.17%) | 0.92 (38.95%) | 0.78 (43.93%) |
| Morocco | 16.17 | 6.63 | 9.55 | 3.67 (22.68%) | 1.66 (24.98%) | 1.97 (20.66%) | 6.27 (38.76%) | 2.62 (39.57%) | 3.67 (38.44%) | 6.24 (38.55%) | 2.35 (35.44%) | 3.9 (40.9%) |

| | Overll difference | | | Aging | | | Population | | | Epidemiological change | | |
|---|---|---|---|---|---|---|---|---|---|---|---|---|
| location | Both | Women | Men | Both | Women | Men | Both | Women | Men | Both | Women | Men |
| Mozambique | 183.59 | 91.95 | 91.64 | -53.1 (-28.93%) | -18.33 (-19.93%) | -33.25 (-36.29%) | 170.43 (92.83%) | 95.69 (104.06%) | 74.91 (81.75%) | 66.26 (36.09%) | 14.6 (15.87%) | 49.98 (54.54%) |
| Myanmar | 77.28 | 44.77 | 32.52 | 29.21 (37.8%) | 19.44 (43.43%) | 9.89 (30.4%) | 44.49 (57.57%) | 28.05 (62.67%) | 16.87 (51.89%) | 3.58 (4.63%) | -2.73 (-6.1%) | 5.76 (17.71%) |
| Namibia | 3.46 | 1.71 | 1.75 | 0.3 (8.53%) | 0.27 (16.08%) | 0.03 (1.93%) | 2.09 (60.46%) | 1.34 (78.32%) | 0.77 (43.81%) | 1.07 (31%) | 0.1 (5.61%) | 0.95 (54.26%) |
| Nauru | 0.01 | 0 | 0 | 0 (37.22%) | 0 (82.03%) | 0 (-90.69%) | 0.01 (89.86%) | 0 (75.49%) | 0 (123.05%) | 0 (-27.08%) | 0 (-57.52%) | 0 (67.64%) |
| Nepal | 63.1 | 29.06 | 34.05 | 10.22 (16.19%) | 5.14 (17.7%) | 5.05 (14.85%) | 25.01 (39.63%) | 12.85 (44.21%) | 11.96 (35.14%) | 27.88 (44.18%) | 11.07 (38.09%) | 17.03 (50.01%) |
| Netherlands | 65.65 | 33.54 | 32.1 | 19.55 (29.78%) | 8.41 (25.08%) | 11.32 (35.26%) | 8.67 (13.21%) | 4.58 (13.66%) | 4.06 (12.63%) | 37.42 (57.01%) | 20.55 (61.27%) | 16.73 (52.11%) |
| New Zealand | 29.94 | 15.19 | 14.75 | 5.49 | 2.41 | 2.96 | 8.47 | 4.25 | 4.23 | 15.98 | 8.53 | 7.57 |

| | Overll difference | | | Aging | | | Population | | | Epidemiological change | | |
|---|---|---|---|---|---|---|---|---|---|---|---|---|
| location | Both | Women | Men | Both | Women | Men | Both | Women | Men | Both | Women | Men |
| | | | | (18.34%) | (15.87%) | (20.06%) | (28.29%) | (27.98%) | (28.65%) | (53.37%) | (56.15%) | (51.29%) |
| Nicaragua | 11.69 | 6.84 | 4.85 | 3.03 (25.93%) | 2.01 (29.43%) | 1.08 (22.34%) | 6.8 (58.16%) | 4.04 (59.04%) | 2.74 (56.56%) | 1.86 (15.92%) | 0.79 (11.53%) | 1.02 (21.09%) |
| Niger | 44.52 | 35.9 | 8.62 | -1.03 (-2.32%) | 2.29 (6.38%) | -2.75 (-31.89%) | 81.07 (182.1%) | 42.6 (118.69%) | 38.21 (443.19%) | -35.52 (-79.78%) | -9 (-25.07%) | -26.84 (-311.31%) |
| Nigeria | 325.07 | 266.14 | 58.93 | -108.07 (-33.25%) | -103.13 (-38.75%) | -18.07 (-30.66%) | 411.02 (126.44%) | 358.22 (134.6%) | 80.35 (136.36%) | 22.11 (6.8%) | 11.04 (4.15%) | -3.36 (-5.7%) |
| Niue | 0 | 0 | 0 | 0 (34.3%) | 0 (24.23%) | 0 (37.02%) | 0 (-48.38%) | 0 (-171.87%) | 0 (-27.48%) | 0 (114.08%) | 0 (247.64%) | 0 (90.46%) |
| North Macedonia | 12.56 | 5.26 | 7.3 | 7.36 (58.56%) | 4.76 (90.49%) | 2.78 (38.08%) | 5.09 (40.54%) | 2.82 (53.69%) | 2.22 (30.33%) | 0.11 (0.9%) | -2.32 (-44.19%) | 2.31 (31.59%) |

| | Overll difference | | | Aging | | | Population | | | Epidemiological change | | |
|---|---|---|---|---|---|---|---|---|---|---|---|---|
| location | Both | Women | Men | Both | Women | Men | Both | Women | Men | Both | Women | Men |
| Northern Mariana Islands | 0.33 | 0.13 | 0.2 | 0.23 (69.27%) | 0.12 (88.68%) | 0.11 (54.75%) | 0.03 (9.24%) | 0.02 (12.73%) | 0.01 (7.09%) | 0.07 (21.49%) | 0 (-1.42%) | 0.08 (38.16%) |
| Norway | 18.21 | 10.09 | 8.12 | 1.64 (9.01%) | 0.41 (4.06%) | 1.2 (14.74%) | 4.11 (22.55%) | 2.16 (21.43%) | 1.88 (23.12%) | 12.47 (68.45%) | 7.52 (74.51%) | 5.05 (62.14%) |
| Oman | 7.56 | 2.3 | 5.26 | -0.13 (-1.67%) | -0.09 (-3.81%) | -0.02 (-0.37%) | 4.82 (63.72%) | 1.71 (74.68%) | 3.1 (58.87%) | 2.87 (37.95%) | 0.67 (29.13%) | 2.18 (41.5%) |
| Pakistan | 255.54 | 161.03 | 94.5 | -26.78 (-10.48%) | -13.25 (-8.23%) | -10.05 (-10.63%) | 196.7 (76.98%) | 128.05 (79.52%) | 70.89 (75.01%) | 85.61 (33.5%) | 46.23 (28.71%) | 33.66 (35.62%) |
| Palau | 0.11 | 0 | 0.11 | 0.05 (47%) | 0 (51.53%) | 0.05 (44.19%) | 0.03 (30.1%) | 0 (27.44%) | 0.04 (36.9%) | 0.03 (22.9%) | 0 (21.03%) | 0.02 (18.9%) |
| Palestine | 9.76 | 4.87 | 4.9 | -0.96 (-9.79%) | -0.54 (-11%) | -0.44 (-8.9%) | 11.39 (116.68%) | 6.22 (127.83%) | 5.11 (104.3%) | -0.67 (-6.89%) | -0.82 (-16.83%) | 0.23 (4.6%) |
| Panama | 10.92 | 6.42 | 4.5 | 3.94 | 2.48 | 1.49 | 6.38 | 3.8 | 2.6 | 0.6 | 0.13 | 0.41 |

| | Overll difference | | | Aging | | | Population | | | Epidemiological change | | |
|---|---|---|---|---|---|---|---|---|---|---|---|---|
| location | Both | Women | Men | Both | Women | Men | Both | Women | Men | Both | Women | Men |
| | | | | (36.04%) | (38.68%) | (33.17%) | (58.45%) | (59.25%) | (57.66%) | (5.52%) | (2.08%) | (9.17%) |
| Papua New Guinea | 5.78 | 3.03 | 2.74 | 0.4 (6.85%) | 0.11 (3.67%) | 0.28 (10.17%) | 7.88 (136.47%) | 4.11 (135.57%) | 3.77 (137.59%) | -2.5 (-43.32%) | -1.19 (-39.25%) | -1.31 (-47.76%) |
| Paraguay | 11.17 | 6 | 5.17 | 1.46 (13.03%) | 0.93 (15.47%) | 0.55 (10.58%) | 5.57 (49.83%) | 3.37 (56.17%) | 2.19 (42.28%) | 4.15 (37.14%) | 1.7 (28.36%) | 2.44 (47.14%) |
| Peru | 77.74 | 55.83 | 21.91 | 19.66 (25.29%) | 15.49 (27.75%) | 4.64 (21.19%) | 34.85 (44.83%) | 25.54 (45.74%) | 9.09 (41.5%) | 23.23 (29.88%) | 14.8 (26.5%) | 8.17 (37.31%) |
| Philippines | 293.42 | 138.43 | 155 | 77.01 (26.25%) | 41.88 (30.25%) | 34.78 (22.44%) | 197.8 (67.41%) | 83.25 (60.14%) | 115.18 (74.31%) | 18.61 (6.34%) | 13.29 (9.6%) | 5.04 (3.25%) |
| Poland | 103.83 | 59.37 | 44.46 | 31 (29.86%) | 20.02 (33.72%) | 10.5 (23.62%) | 9.02 (8.69%) | 5.98 (10.07%) | 3.03 (6.82%) | 63.81 (61.45%) | 33.37 (56.21%) | 30.92 (69.56%) |
| Portugal | 61.39 | 23.85 | 37.55 | 19.99 (32.56%) | 9.88 (41.43%) | 10.4 (27.7%) | 6.29 (10.25%) | 3.12 (13.08%) | 3.09 (8.24%) | 35.11 (57.19%) | 10.85 (45.49%) | 24.05 (64.06%) |
| Puerto Rico | 17.49 | 6.11 | 11.39 | 11.73 | 6.43 | 5.3 | 1.66 | 0.95 | 0.7 | 4.1 | -1.27 | 5.39 |

| | Overll difference | | | Aging | | | Population | | | Epidemiological change | | |
|---|---|---|---|---|---|---|---|---|---|---|---|---|
| location | Both | Women | Men | Both | Women | Men | Both | Women | Men | Both | Women | Men |
| | | | | (67.06%) | (105.32%) | (46.51%) | (9.48%) | (15.5%) | (6.14%) | (23.46%) | (-20.82%) | (47.35%) |
| Qatar | 14.93 | 4.35 | 10.58 | 0.87 (5.82%) | 0.45 (10.32%) | 0.44 (4.14%) | 11.17 (74.83%) | 3.72 (85.58%) | 7.41 (70.02%) | 2.89 (19.35%) | 0.18 (4.1%) | 2.73 (25.84%) |
| Republic of Korea | 400.13 | 184.25 | 215.88 | 812.7 (203.11%) | 420.6 (228.27%) | 399.7 (185.15%) | 280.52 (70.11%) | 145.69 (79.07%) | 134.65 (62.37%) | -693.09 (-173.22%) | -382.04 (-207.34%) | -318.48 (-147.52%) |
| Republic of Moldova | 6.23 | 3.25 | 2.97 | 4.34 (69.67%) | 2.44 (75.06%) | 1.92 (64.61%) | -0.51 (-8.23%) | -0.33 (-10.04%) | -0.19 (-6.29%) | 2.4 (38.56%) | 1.14 (34.97%) | 1.24 (41.68%) |
| Romania | 80.6 | 43.95 | 36.65 | 28.12 (34.89%) | 17.58 (40.01%) | 10.64 (29.04%) | -7.62 (-9.45%) | -3.77 (-8.57%) | -3.8 (-10.36%) | 60.1 (74.56%) | 30.13 (68.56%) | 29.8 (81.32%) |
| Russian Federation | 385.22 | 209.76 | 175.46 | 111.69 (28.99%) | 51.95 (24.76%) | 67.09 (38.24%) | 8.6 (2.23%) | 5.34 (2.54%) | 3.3 (1.88%) | 264.93 (68.77%) | 152.48 (72.69%) | 105.07 (59.88%) |
| Rwanda | 16.56 | 13.28 | 3.28 | 1.47 | 2.02 | -0.01 | 23.55 | 17.64 | 5.99 | -8.46 | -6.38 | -2.7 |

| | Overll difference | | | Aging | | | Population | | | Epidemiological change | | |
|---|---|---|---|---|---|---|---|---|---|---|---|---|
| location | Both | Women | Men | Both | Women | Men | Both | Women | Men | Both | Women | Men |
| | | | | (8.86%) | (15.21%) | (-0.32%) | (142.23%) | (132.84%) | (182.67%) | (-51.09%) | (-48.05%) | (-82.35%) |
| Saint Kitts and Nevis | 0.1 | 0 | 0.1 | -0.02 (-23.49%) | -0.01 (638.22%) | -0.01 (-9.51%) | 0.12 (120.08%) | 0.06 (-2743.05%) | 0.06 (60.39%) | 0 (3.41%) | -0.04 (2204.83%) | 0.05 (49.12%) |
| Saint Lucia | 0.2 | 0.07 | 0.13 | 0.13 (63.05%) | 0.07 (95.57%) | 0.06 (44.4%) | 0.15 (72.9%) | 0.08 (105.18%) | 0.07 (53.38%) | -0.07 (-35.95%) | -0.07 (-100.75%) | 0 (2.22%) |
| Saint Vincent and the Grenadines | 0.16 | 0.03 | 0.13 | 0.12 (72.67%) | 0.04 (135.11%) | 0.08 (60.97%) | 0.08 (46.84%) | 0.03 (95.49%) | 0.05 (34.81%) | -0.03 (-19.52%) | -0.04 (-130.6%) | 0.01 (4.22%) |
| Samoa | 0.31 | 0.16 | 0.16 | 0.13 (42.34%) | 0.05 (30.29%) | 0.09 (55.83%) | 0.18 (58.58%) | 0.08 (52.16%) | 0.1 (63.77%) | 0 (-0.92%) | 0.03 (17.55%) | -0.03 (-19.6%) |
| San Marino | 0.08 | 0.04 | 0.05 | 0.03 (39.62%) | 0.01 (34.16%) | 0.02 (46.08%) | 0.03 (37.17%) | 0.01 (39.05%) | 0.02 (35.13%) | 0.02 (23.21%) | 0.01 (26.79%) | 0.01 (18.79%) |

| | Overll difference | | | Aging | | | Population | | | Epidemiological change | | |
|---|---|---|---|---|---|---|---|---|---|---|---|---|
| location | Both | Women | Men | Both | Women | Men | Both | Women | Men | Both | Women | Men |
| Sao Tome and Principe | 0.15 | 0.08 | 0.07 | -0.07 (-44.93%) | -0.04 (-45.94%) | -0.02 (-32.19%) | 0.2 (129.98%) | 0.1 (121.35%) | 0.1 (139.83%) | 0.02 (14.95%) | 0.02 (24.59%) | -0.01 (-7.63%) |
| Saudi Arabia | 131.77 | 43.12 | 88.65 | -6.16 (-4.67%) | -2.19 (-5.08%) | -2.21 (-2.49%) | 105.86 (80.33%) | 35.98 (83.44%) | 69.59 (78.5%) | 32.07 (24.34%) | 9.33 (21.64%) | 21.26 (23.99%) |
| Senegal | 64.22 | 51.31 | 12.91 | 1.15 (1.79%) | 5.21 (10.15%) | -1.73 (-13.42%) | 69.42 (108.1%) | 43.91 (85.57%) | 24.82 (192.32%) | -6.35 (-9.89%) | 2.2 (4.28%) | -10.18 (-78.9%) |
| Serbia | 30.67 | 15.71 | 14.96 | 25.9 (84.46%) | 15.91 (101.28%) | 9.96 (66.59%) | 0.99 (3.23%) | 0.61 (3.91%) | 0.39 (2.59%) | 3.77 (12.31%) | -0.82 (-5.19%) | 4.61 (30.83%) |
| Seychelles | 0.07 | -0.05 | 0.11 | 0.1 (145.18%) | 0.03 (-73.19%) | 0.07 (65.18%) | 0.28 (408.75%) | 0.1 (-231.97%) | 0.18 (160.22%) | -0.32 (-453.92%) | -0.18 (405.16%) | -0.14 (-125.4%) |
| Sierra Leone | 9.41 | 12.31 | -2.9 | -9.21 | -3.79 | -5.18 | 33.8 | 16.6 | 17.18 | -15.18 | -0.5 | -14.9 |

| | Overll difference | | | Aging | | | Population | | | Epidemiological change | | |
|---|---|---|---|---|---|---|---|---|---|---|---|---|
| location | Both | Women | Men | Both | Women | Men | Both | Women | Men | Both | Women | Men |
| | | | | (-97.94%) | (-30.81%) | (178.69%) | (359.26%) | (134.92%) | (-593.18%) | (-161.33%) | (-4.1%) | (514.49%) |
| Singapore | 19.13 | 8.65 | 10.48 | 11.39 (59.55%) | 4.91 (56.74%) | 6.7 (63.94%) | 11.26 (58.86%) | 4.83 (55.82%) | 6.5 (62.01%) | -3.52 (-18.42%) | -1.09 (-12.57%) | -2.72 (-25.95%) |
| Slovakia | 8.09 | 2.48 | 5.61 | 9.91 (122.55%) | 6.24 (251.67%) | 3.8 (67.79%) | 4.38 (54.22%) | 2.57 (103.73%) | 1.78 (31.83%) | -6.21 (-76.77%) | -6.33 (-255.4%) | 0.02 (0.38%) |
| Slovenia | 14.21 | 5.43 | 8.77 | 7.52 (52.92%) | 3.8 (69.98%) | 3.98 (45.42%) | 1.83 (12.89%) | 0.73 (13.49%) | 1.06 (12.14%) | 4.86 (34.19%) | 0.9 (16.53%) | 3.72 (42.44%) |
| Solomon Islands | 0.85 | 0.43 | 0.42 | 0.1 (11.77%) | 0.07 (17.12%) | 0.02 (3.7%) | 0.83 (98%) | 0.34 (79.9%) | 0.48 (114.77%) | -0.08 (-9.76%) | 0.01 (2.98%) | -0.08 (-18.46%) |
| Somalia | 37.96 | 27.7 | 10.27 | -2.55 (-6.72%) | 0.77 (2.77%) | -2.59 (-25.28% | 43.98 (115.85% | 27.43 (99.05%) | 16.58 (161.49 | -3.47 (-9.13%) | -0.5 (-1.82%) | -3.72 (-36.21% |

| | Overll difference | | | Aging | | | Population | | | Epidemiological change | | |
|---|---|---|---|---|---|---|---|---|---|---|---|---|
| location | Both | Women | Men | Both | Women | Men | Both | Women | Men | Both | Women | Men |
| | | | | | | ) | ) | | %) | | | ) |
| South Africa | 332.06 | 125.49 | 206.57 | 51.98 (15.65%) | 27.52 (21.93%) | 22.91 (11.09%) | 138.51 (41.71%) | 66.07 (52.65%) | 72.81 (35.25%) | 141.57 (42.63%) | 31.89 (25.41%) | 110.85 (53.66%) |
| South Sudan | 19.67 | 12.04 | 7.64 | -3.12 (-15.85%) | -1.36 (-11.26%) | -1.35 (-17.72%) | 12.97 (65.9%) | 9.08 (75.44%) | 4.35 (56.94%) | 9.83 (49.95%) | 4.31 (35.82%) | 4.64 (60.78%) |
| Spain | 198.98 | 84.49 | 114.49 | 73.89 (37.13%) | 37.85 (44.8%) | 37.57 (32.81%) | 46.34 (23.29%) | 22.36 (26.46%) | 23.91 (20.88%) | 78.76 (39.58%) | 24.28 (28.74%) | 53.02 (46.3%) |
| Sri Lanka | 20.51 | 7.9 | 12.61 | 16.79 (81.88%) | 8.89 (112.59%) | 7.66 (60.73%) | 11.2 (54.6%) | 5.82 (73.66%) | 5.26 (41.7%) | -7.48 (-36.48%) | -6.81 (-86.25%) | -0.31 (-2.43%) |
| Sudan | 62.79 | 26.63 | 36.16 | -7.75 (-12.34%) | -3.88 (-14.57%) | -3.87 (-10.69%) | 47.98 (76.42%) | 22.63 (84.98%) | 25.36 (70.12%) | 22.55 (35.92%) | 7.88 (29.59%) | 14.67 (40.57%) |
| Suriname | 1.27 | 0.58 | 0.69 | 0.4 | 0.23 | 0.18 | 0.52 | 0.29 | 0.23 | 0.34 | 0.06 | 0.28 |

| | Overll difference | | | Aging | | | Population | | | Epidemiological change | | |
|---|---|---|---|---|---|---|---|---|---|---|---|---|
| location | Both | Women | Men | Both | Women | Men | Both | Women | Men | Both | Women | Men |
| | | | | (31.53%) | (39.05%) | (25.41%) | (41.34%) | (51.07%) | (33.67%) | (27.14%) | (9.89%) | (40.93%) |
| Sweden | 20.91 | 6.74 | 14.17 | 6.91 (33.03%) | 2.56 (37.97%) | 4.55 (32.1%) | 8.17 (39.09%) | 3.84 (56.91%) | 4.26 (30.09%) | 5.83 (27.89%) | 0.35 (5.12%) | 5.36 (37.81%) |
| Switzerland | 25.31 | 11.97 | 13.35 | 9.22 (36.43%) | 2.99 (25.01%) | 7.37 (55.23%) | 9.91 (39.14%) | 3.99 (33.31%) | 6.04 (45.26%) | 6.18 (24.43%) | 4.99 (41.69%) | -0.07 (-0.49%) |
| Syrian Arab Republic | 46.27 | 22.66 | 23.61 | 25.81 (55.79%) | 12.01 (52.99%) | 13.63 (57.72%) | 21.81 (47.13%) | 13.81 (60.93%) | 8.23 (34.86%) | -1.35 (-2.91%) | -3.15 (-13.92%) | 1.75 (7.42%) |
| Taiwan (Province of China) | 304.05 | 157.97 | 146.08 | 122.39 (40.25%) | 61.57 (38.98%) | 59.07 (40.44%) | 62.48 (20.55%) | 32.09 (20.32%) | 29.32 (20.07%) | 119.18 (39.2%) | 64.31 (40.71%) | 57.69 (39.49%) |
| Tajikistan | 7.71 | 4.07 | 3.64 | -0.96 (-12.39%) | -1.29 (-31.6%) | 0.2 (5.53%) | 10.04 (130.22%) | 6.12 (150.24%) | 3.85 (105.92%) | -1.38 (-17.83%) | -0.76 (-18.64%) | -0.42 (-11.45%) |
| Thailand | 812.2 | 326.12 | 486.08 | 624.74 (76.92%) | 301.33 (92.4%) | 325.81 (67.03%) | 281.65 (34.68%) | 140.36 (43.04%) | 139.63 (28.73%) | -94.19 (-11.6%) | -115.56 (-35.44% | 20.63 (4.25%) |

| | Overll difference | | | Aging | | | Population | | | Epidemiological change | | |
|---|---|---|---|---|---|---|---|---|---|---|---|---|
| location | Both | Women | Men | Both | Women | Men | Both | Women | Men | Both | Women | Men |
| | | | | | | | | | | | ) | |
| Timor-Leste | 1.67 | 1.06 | 0.61 | 0.8 (47.98%) | 0.52 (49.3%) | 0.28 (45.95%) | 1.01 (60.59%) | 0.67 (62.68%) | 0.36 (58.99%) | -0.14 (-8.57%) | -0.13 (-11.98% ) | -0.03 (-4.94%) |
| Togo | 29.04 | 21.43 | 7.6 | 3.19 (10.98%) | 3.86 (18.02%) | 0.28 (3.64%) | 23.32 (80.31%) | 15.52 (72.4%) | 7.69 (101.15 %) | 2.53 (8.71%) | 2.05 (9.58%) | -0.36 (-4.79%) |
| Tokelau | 0 | 0 | 0 | 0 (38.15%) | 0 (135.69 %) | 0 (15.92%) | 0 (-3.66%) | 0 (-29.53%) | 0 (1.53%) | 0 (65.51%) | 0 (-6.15%) | 0 (82.56%) |
| Tonga | 0.84 | 0.27 | 0.57 | 0.33 (39.45%) | 0.14 (51.05%) | 0.19 (32.88%) | 0.25 (29.4%) | 0.1 (38.11%) | 0.14 (24.03%) | 0.26 (31.15%) | 0.03 (10.84%) | 0.24 (43.09%) |
| Trinidad and Tobago | 3.32 | 1.91 | 1.41 | 1.77 (53.3%) | 1.13 (59.21%) | 0.63 (44.52%) | 1.18 (35.68%) | 0.75 (39.34%) | 0.43 (30.56%) | 0.37 (11.02%) | 0.03 (1.45%) | 0.35 (24.92%) |
| Tunisia | 17.79 | 7.55 | 10.24 | 5.57 | 2.9 | 2.66 | 6.68 | 3.29 | 3.39 | 5.53 | 1.35 | 4.19 |

| | Overll difference | | | Aging | | | Population | | | Epidemiological change | | |
|---|---|---|---|---|---|---|---|---|---|---|---|---|
| location | Both | Women | Men | Both | Women | Men | Both | Women | Men | Both | Women | Men |
| | | | | (31.33%) | (38.47%) | (25.97%) | (37.58%) | (43.59%) | (33.12%) | (31.09%) | (17.94%) | (40.9%) |
| Turkey | 237.9 | 102.8 | 135.1 | 88.38 (37.15%) | 40.92 (39.81%) | 47.79 (35.38%) | 99.3 (41.74%) | 47.31 (46.03%) | 51.94 (38.45%) | 50.22 (21.11%) | 14.56 (14.17%) | 35.37 (26.18%) |
| Turkmenistan | 13.62 | 8.07 | 5.56 | 2.95 (21.65%) | 1.43 (17.78%) | 1.58 (28.5%) | 6.2 (45.52%) | 2.88 (35.7%) | 3.33 (59.9%) | 4.47 (32.83%) | 3.75 (46.51%) | 0.64 (11.6%) |
| Tuvalu | 0.03 | 0.01 | 0.01 | 0 (18.25%) | 0 (35.46%) | 0 (5.35%) | 0.01 (53.45%) | 0.01 (44.62%) | 0.01 (57.04%) | 0.01 (28.3%) | 0 (19.92%) | 0.01 (37.61%) |
| Uganda | 82.47 | 59.33 | 23.14 | -13.4 (-16.25%) | -3.31 (-5.58%) | -9.36 (-40.44%) | 87.93 (106.63%) | 49.29 (83.08%) | 38.4 (165.95%) | 7.93 (9.62%) | 13.35 (22.5%) | -5.9 (-25.51%) |
| Ukraine | -13.69 | -15.29 | 1.6 | 18.53 (-135.33%) | 8.51 (-55.69%) | 11.36 (710.84%) | -11.49 (83.9%) | -6.44 (42.12%) | -5.01 (-313.65%) | -20.73 (151.43%) | -17.36 (113.57%) | -4.75 (-297.2%) |
| United Arab Emirates | 53.98 | 14.98 | 39 | 8.9 (16.5%) | -1.4 (-9.34%) | 8.45 (21.67%) | 36.15 (66.96%) | 12.88 (86.01%) | 22.93 (58.79%) | 8.93 (16.54%) | 3.49 (23.33%) | 7.62 (19.54%) |

| | Overll difference | | | Aging | | | Population | | | Epidemiological change | | |
|---|---|---|---|---|---|---|---|---|---|---|---|---|
| location | Both | Women | Men | Both | Women | Men | Both | Women | Men | Both | Women | Men |
| United Kingdom | 456.97 | 259.38 | 197.6 | 60.72 (13.29%) | 18.3 (7.05%) | 40.73 (20.61%) | 61.12 (13.37%) | 33.72 (13%) | 26.84 (13.58%) | 335.14 (73.34%) | 207.36 (79.94%) | 130.03 (65.8%) |
| United Republic of Tanzania | 98.78 | 72.95 | 25.82 | -4.76 (-4.82%) | -1.25 (-1.71%) | -2.75 (-10.66%) | 112.6 (113.99%) | 72.29 (99.09%) | 40.15 (155.49%) | -9.06 (-9.17%) | 1.91 (2.62%) | -11.57 (-44.83%) |
| United States of America | 2138.76 | 901.51 | 1237.25 | 433.16 (20.25%) | 152.61 (16.93%) | 299.78 (24.23%) | 490.79 (22.95%) | 220.47 (24.46%) | 271.21 (21.92%) | 1214.81 (56.8%) | 528.43 (58.62%) | 666.27 (53.85%) |
| United States Virgin Islands | 0.44 | 0.13 | 0.31 | 0.31 (69.79%) | 0.16 (124.92%) | 0.15 (47.64%) | -0.01 (-1.84%) | -0.01 (-4.09%) | 0 (-0.82%) | 0.14 (32.05%) | -0.03 (-20.83%) | 0.17 (53.18%) |
| Uruguay | 9.73 | 3.39 | 6.34 | 1.26 (12.91%) | 0.59 (17.48%) | 0.67 (10.57%) | 1.26 (12.9%) | 0.54 (15.83%) | 0.69 (10.92%) | 7.22 (74.19%) | 2.26 (66.69%) | 4.98 (78.51%) |
| Uzbekistan | 72.29 | 39.54 | 32.76 | 8.96 (12.39%) | 2.81 (7.11%) | 6.1 (18.62%) | 36.65 (50.7%) | 21.21 (53.65%) | 15.37 (46.92%) | 26.69 (36.92%) | 15.52 (39.24%) | 11.29 (34.46%) |
| Vanuatu | 0.5 | 0.25 | 0.25 | 0.09 | 0.06 | 0.03 | 0.37 | 0.18 | 0.19 | 0.04 | 0.01 | 0.03 |

| | Overll difference | | | Aging | | | Population | | | Epidemiological change | | |
|---|---|---|---|---|---|---|---|---|---|---|---|---|
| location | Both | Women | Men | Both | Women | Men | Both | Women | Men | Both | Women | Men |
| | | | | (17.68%) | (23.74%) | (11.15%) | (73.61%) | (71.78%) | (75.39%) | (8.71%) | (4.47%) | (13.46%) |
| Venezuela (Bolivarian Republic of) | 37.56 | 16.65 | 20.9 | 40.1 (106.78%) | 24.41 (146.6%) | 15.6 (74.65%) | 35.71 (95.07%) | 22.99 (138.03%) | 13.21 (63.18%) | -38.25 (-101.85%) | -30.75 (-184.63%) | -7.91 (-37.83%) |
| Viet Nam | 603.68 | 258.63 | 345.06 | 215.71 (35.73%) | 90.25 (34.89%) | 127.35 (36.91%) | 396.87 (65.74%) | 155.39 (60.08%) | 247.77 (71.81%) | -8.9 (-1.47%) | 12.99 (5.02%) | -30.06 (-8.71%) |
| Yemen | 14.27 | 8.15 | 6.12 | -0.41 (-2.86%) | -0.42 (-5.17%) | 0.03 (0.53%) | 17.98 (126.02%) | 8.83 (108.27%) | 9.17 (149.94%) | -3.3 (-23.16%) | -0.25 (-3.1%) | -3.09 (-50.48%) |
| Zambia | 14.83 | 10.74 | 4.1 | -3.84 (-25.91%) | 0.72 (6.7%) | -3.56 (-86.77%) | 43.46 (292.96%) | 25.38 (236.38%) | 18.03 (439.78%) | -24.78 (-167.05%) | -15.36 (-143.08%) | -10.37 (-253.02%) |
| Zimbabwe | 74.66 | 61.25 | 13.41 | -1.86 (-2.49%) | 2.57 (4.19%) | -2.92 (-21.81%) | 42.88 (57.43%) | 30.42 (49.67%) | 12.96 (96.66%) | 33.64 (45.06%) | 28.26 (46.14%) | 3.37 (25.15%) |

## Table S9 Frontier deaths, and effective difference by country or territory in 2021

| Location | SDI | Rate of Deaths | Frontier Deaths | Effective difference | Effective difference rank (Age-standardized Deaths rank) |
|---|---|---|---|---|---|
| Mongolia | 0.617621565 | 5.87(3.61 to 9.12) | 0.03 | 5.84 | 204 (204) |
| Gambia | 0.40971416 | 3.99(2.18 to 6.4) | 0.04 | 3.95 | 203 (203) |
| Mozambique | 0.326462614 | 3.43(1.74 to 6.44) | 0.06 | 3.36 | 202 (202) |
| Mauritania | 0.4989451 | 3.13(1.66 to 4.95) | 0.04 | 3.09 | 201 (201) |
| Eswatini | 0.585459713 | 2.81(1.23 to 5.97) | 0.03 | 2.77 | 200 (199) |
| Mali | 0.268579941 | 2.87(1.83 to 4.47) | 0.15 | 2.72 | 199 (200) |
| Qatar | 0.846860584 | 2.68(1.65 to 4.21) | 0.03 | 2.64 | 198 (198) |
| Tonga | 0.626349936 | 2.43(1.47 to 3.6) | 0.03 | 2.4 | 197 (197) |
| Egypt | 0.606787094 | 2.42(1.5 to 3.54) | 0.03 | 2.38 | 196 (196) |
| Guinea | 0.336401293 | 2.42(1.36 to 3.61) | 0.04 | 2.37 | 195 (195) |
| Liberia | 0.352442452 | 2.38(1.5 to 3.62) | 0.04 | 2.35 | 194 (194) |
| Guinea-Bissau | 0.353109621 | 2.31(1.45 to 3.51) | 0.04 | 2.28 | 193 (193) |
| Cabo Verde | 0.533534539 | 2.11(1.23 to 3.23) | 0.04 | 2.07 | 192 (192) |
| Lesotho | 0.510393066 | 2.1(0.91 to 4.47) | 0.04 | 2.07 | 191 (191) |
| Zimbabwe | 0.473819486 | 2.06(1.18 to 3.22) | 0.04 | 2.02 | 190 (190) |

| Location | SDI | Rate of Deaths | Frontier Deaths | Effective difference | Effective difference rank (Age-standardized Deaths rank) |
|---|---|---|---|---|---|
| Burkina Faso | 0.285118402 | 2(0.97 to 3.97) | 0.1 | 1.9 | 189 (189) |
| United Arab Emirates | 0.849317734 | 1.91(1.15 to 2.96) | 0.03 | 1.88 | 188 (188) |
| Cameroon | 0.479691223 | 1.83(0.91 to 2.94) | 0.04 | 1.79 | 187 (187) |
| Benin | 0.373486574 | 1.77(1.12 to 2.74) | 0.04 | 1.73 | 186 (186) |
| Senegal | 0.408054193 | 1.61(1.01 to 2.46) | 0.04 | 1.58 | 185 (185) |
| Cook Islands | 0.779109955 | 1.33(0.87 to 1.94) | 0.03 | 1.3 | 184 (183) |
| Chad | 0.240436019 | 1.42(0.74 to 2.73) | 0.15 | 1.27 | 183 (184) |
| Sierra Leone | 0.358665881 | 1.23(0.74 to 1.95) | 0.04 | 1.19 | 182 (182) |
| Togo | 0.408533695 | 1.22(0.72 to 1.93) | 0.04 | 1.19 | 181 (180) |
| Ghana | 0.56493039 | 1.16(0.63 to 1.8) | 0.04 | 1.13 | 180 (178) |
| Saudi Arabia | 0.815143493 | 1.14(0.74 to 1.67) | 0.03 | 1.11 | 178 (176) |
| Thailand | 0.682547933 | 1.15(0.74 to 1.71) | 0.03 | 1.11 | 179 (177) |
| Gabon | 0.634691393 | 1.08(0.6 to 1.82) | 0.03 | 1.05 | 177 (175) |
| Niger | 0.168072774 | 1.23(0.68 to 2.16) | 0.21 | 1.02 | 176 (181) |
| Republic of Korea | 0.886675267 | 1.04(0.66 to 1.56) | 0.03 | 1.01 | 175 (174) |
| Viet Nam | 0.627933721 | 1.04(0.67 to 1.57) | 0.03 | 1.01 | 174 (173) |

| Location | SDI | Rate of Deaths | Frontier Deaths | Effective difference | Effective difference rank (Age-standardized Deaths rank) |
|---|---|---|---|---|---|
| South Africa | 0.679626598 | 1.02(0.84 to 1.23) | 0.03 | 0.99 | 173 (172) |
| North Macedonia | 0.750629703 | 1.02(0.62 to 1.53) | 0.03 | 0.98 | 172 (171) |
| Libya | 0.725771399 | 1(0.64 to 1.52) | 0.03 | 0.97 | 171 (169) |
| American Samoa | 0.723727533 | 0.96(0.63 to 1.4) | 0.03 | 0.93 | 170 (168) |
| Uganda | 0.423261181 | 0.96(0.58 to 1.49) | 0.04 | 0.93 | 169 (167) |
| Albania | 0.706849791 | 0.91(0.53 to 1.48) | 0.03 | 0.88 | 168 (166) |
| Northern Mariana Islands | 0.771535213 | 0.91(0.59 to 1.33) | 0.03 | 0.87 | 167 (165) |
| South Sudan | 0.278371125 | 1.01(0.6 to 1.65) | 0.15 | 0.86 | 166 (170) |
| Taiwan (Province of China) | 0.874747053 | 0.89(0.61 to 1.25) | 0.03 | 0.85 | 165 (164) |
| Andorra | 0.869444113 | 0.87(0.51 to 1.39) | 0.03 | 0.84 | 164 (163) |
| Comoros | 0.475978688 | 0.85(0.49 to 1.29) | 0.04 | 0.82 | 163 (162) |
| Bahrain | 0.753043204 | 0.83(0.53 to 1.26) | 0.03 | 0.8 | 161 (160) |
| Botswana | 0.642721629 | 0.83(0.44 to 1.57) | 0.04 | 0.8 | 162 (161) |
| Greenland | 0.826210336 | 0.81(0.48 to 1.21) | 0.04 | 0.77 | 159 (159) |
| Montenegro | 0.795800584 | 0.81(0.51 to 1.2) | 0.03 | 0.77 | 160 (158) |
| Azerbaijan | 0.694851274 | 0.8(0.39 to 1.46) | 0.03 | 0.76 | 158 (157) |

| Location | SDI | Rate of Deaths | Frontier Deaths | Effective difference | Effective difference rank (Age-standardized Deaths rank) |
|---|---|---|---|---|---|
| Zambia | 0.505948954 | 0.78(0.34 to 1.78) | 0.04 | 0.75 | 157 (156) |
| Kenya | 0.523768077 | 0.77(0.57 to 1.02) | 0.04 | 0.73 | 156 (155) |
| Congo | 0.583075236 | 0.74(0.3 to 1.72) | 0.03 | 0.71 | 154 (153) |
| Nigeria | 0.503390833 | 0.75(0.52 to 1.07) | 0.04 | 0.71 | 155 (154) |
| Kiribati | 0.527186583 | 0.72(0.44 to 1.02) | 0.04 | 0.69 | 151 (150) |
| Palestine | 0.631011665 | 0.72(0.47 to 1.07) | 0.03 | 0.69 | 152 (151) |
| United Republic of Tanzania | 0.446568273 | 0.73(0.44 to 1.09) | 0.04 | 0.69 | 153 (152) |
| Djibouti | 0.487958371 | 0.71(0.43 to 1.13) | 0.04 | 0.68 | 150 (149) |
| Palau | 0.754046931 | 0.71(0.43 to 1.11) | 0.03 | 0.68 | 149 (147) |
| Bosnia and Herzegovina | 0.723077893 | 0.7(0.43 to 1.04) | 0.03 | 0.67 | 145 (145) |
| Brunei Darussalam | 0.810234367 | 0.71(0.44 to 1.08) | 0.03 | 0.67 | 148 (148) |
| Costa Rica | 0.700340477 | 0.7(0.46 to 0.99) | 0.03 | 0.67 | 146 (144) |
| Malawi | 0.384553634 | 0.71(0.44 to 1.03) | 0.04 | 0.67 | 147 (146) |
| Monaco | 0.908262831 | 0.69(0.41 to 1.05) | 0.03 | 0.66 | 144 (143) |
| Equatorial Guinea | 0.657857456 | 0.69(0.39 to 1.15) | 0.03 | 0.65 | 143 (142) |
| Angola | 0.453721949 | 0.67(0.16 to 1.78) | 0.04 | 0.64 | 141 (140) |

| Location | SDI | Rate of Deaths | Frontier Deaths | Effective difference | Effective difference rank (Age-standardized Deaths rank) |
|---|---|---|---|---|---|
| Malaysia | 0.742523828 | 0.68(0.45 to 1.01) | 0.03 | 0.64 | 142 (141) |
| Armenia | 0.701833194 | 0.66(0.43 to 0.96) | 0.03 | 0.63 | 138 (136) |
| Cambodia | 0.473621491 | 0.66(0.33 to 1.28) | 0.04 | 0.63 | 139 (138) |
| Canada | 0.87317068 | 0.66(0.47 to 0.91) | 0.03 | 0.63 | 140 (139) |
| Rwanda | 0.435588706 | 0.66(0.4 to 1.01) | 0.04 | 0.63 | 137 (137) |
| Syrian Arab Republic | 0.623004075 | 0.65(0.4 to 0.97) | 0.03 | 0.62 | 136 (134) |
| Afghanistan | 0.337199998 | 0.65(0.38 to 1.11) | 0.05 | 0.6 | 135 (135) |
| Iraq | 0.662626231 | 0.64(0.4 to 0.95) | 0.03 | 0.6 | 134 (133) |
| Niue | 0.72622205 | 0.63(0.38 to 0.99) | 0.03 | 0.6 | 133 (132) |
| Guam | 0.803982203 | 0.61(0.42 to 0.87) | 0.03 | 0.58 | 132 (131) |
| Lao People's Democratic Republic | 0.489136091 | 0.59(0.33 to 0.94) | 0.04 | 0.56 | 131 (130) |
| Fiji | 0.675051631 | 0.58(0.36 to 0.88) | 0.03 | 0.55 | 129 (128) |
| Micronesia (Federated States of) | 0.587534967 | 0.59(0.34 to 0.93) | 0.03 | 0.55 | 130 (129) |
| Eritrea | 0.403863943 | 0.58(0.33 to 0.95) | 0.04 | 0.54 | 128 (127) |
| Australia | 0.844252814 | 0.57(0.39 to 0.79) | 0.03 | 0.53 | 127 (125) |
| Guatemala | 0.539972424 | 0.56(0.37 to 0.8) | 0.04 | 0.53 | 126 (124) |

| Location | SDI | Rate of Deaths | Frontier Deaths | Effective difference | Effective difference rank (Age-standardized Deaths rank) |
|---|---|---|---|---|---|
| Philippines | 0.651219329 | 0.56(0.45 to 0.7) | 0.03 | 0.53 | 125 (123) |
| Central African Republic | 0.30916769 | 0.57(0.21 to 1.34) | 0.04 | 0.52 | 124 (126) |
| Samoa | 0.593392769 | 0.54(0.34 to 0.79) | 0.03 | 0.51 | 123 (122) |
| Nauru | 0.625177834 | 0.52(0.32 to 0.82) | 0.03 | 0.49 | 121 (120) |
| Tuvalu | 0.576620529 | 0.52(0.31 to 0.84) | 0.03 | 0.49 | 122 (121) |
| Tokelau | 0.686425621 | 0.51(0.28 to 0.89) | 0.03 | 0.48 | 119 (118) |
| Turkmenistan | 0.682160776 | 0.52(0.33 to 0.77) | 0.03 | 0.48 | 120 (119) |
| China | 0.72162976 | 0.51(0.39 to 0.64) | 0.03 | 0.47 | 118 (117) |
| Coted'Ivoire | 0.425941883 | 0.5(0.26 to 0.81) | 0.04 | 0.47 | 117 (116) |
| Oman | 0.773391602 | 0.5(0.32 to 0.74) | 0.03 | 0.47 | 114 (113) |
| Seychelles | 0.730150775 | 0.5(0.33 to 0.73) | 0.03 | 0.47 | 115 (114) |
| Sudan | 0.541949735 | 0.5(0.29 to 0.8) | 0.04 | 0.47 | 116 (115) |
| Kazakhstan | 0.725144495 | 0.5(0.32 to 0.7) | 0.03 | 0.46 | 113 (111) |
| Maldives | 0.650886627 | 0.5(0.3 to 0.76) | 0.03 | 0.46 | 112 (112) |
| Bolivia (Plurinational State of) | 0.599010799 | 0.48(0.27 to 0.8) | 0.03 | 0.45 | 109 (108) |
| Iran (Islamic Republic of) | 0.697207398 | 0.49(0.4 to 0.58) | 0.03 | 0.45 | 110 (109) |

| Location | SDI | Rate of Deaths | Frontier Deaths | Effective difference | Effective difference rank (Age-standardized Deaths rank) |
|---|---|---|---|---|---|
| Slovenia | 0.842430731 | 0.49(0.32 to 0.72) | 0.03 | 0.45 | 111 (110) |
| Indonesia | 0.656868336 | 0.48(0.25 to 0.75) | 0.03 | 0.44 | 108 (107) |
| Bhutan | 0.473062378 | 0.47(0.26 to 0.75) | 0.04 | 0.43 | 106 (105) |
| Ecuador | 0.661017053 | 0.46(0.32 to 0.65) | 0.03 | 0.43 | 105 (103) |
| United States of America | 0.862448354 | 0.47(0.4 to 0.55) | 0.03 | 0.43 | 107 (106) |
| Democratic People's Republic of Korea | 0.569854634 | 0.46(0.26 to 0.72) | 0.03 | 0.42 | 103 (102) |
| Dominica | 0.746967185 | 0.46(0.28 to 0.69) | 0.03 | 0.42 | 104 (104) |
| Vanuatu | 0.473100706 | 0.45(0.25 to 0.77) | 0.04 | 0.42 | 102 (101) |
| France | 0.838364875 | 0.43(0.28 to 0.61) | 0.03 | 0.4 | 99 (98) |
| Honduras | 0.513037248 | 0.43(0.25 to 0.69) | 0.04 | 0.4 | 100 (99) |
| New Zealand | 0.849442499 | 0.43(0.36 to 0.52) | 0.03 | 0.4 | 101 (100) |
| Grenada | 0.668993028 | 0.42(0.29 to 0.6) | 0.03 | 0.39 | 95 (94) |
| Madagascar | 0.400246943 | 0.42(0.24 to 0.66) | 0.04 | 0.39 | 97 (96) |
| Serbia | 0.792416294 | 0.43(0.26 to 0.65) | 0.03 | 0.39 | 98 (97) |
| United Kingdom | 0.859000182 | 0.42(0.35 to 0.5) | 0.03 | 0.39 | 96 (95) |
| India | 0.575401649 | 0.41(0.34 to 0.49) | 0.03 | 0.38 | 94 (93) |

| Location | SDI | Rate of Deaths | Frontier Deaths | Effective difference | Effective difference rank (Age-standardized Deaths rank) |
|---|---|---|---|---|---|
| Marshall Islands | 0.574091128 | 0.4(0.22 to 0.67) | 0.03 | 0.37 | 92 (90) |
| Namibia | 0.617564872 | 0.41(0.26 to 0.62) | 0.03 | 0.37 | 93 (92) |
| Solomon Islands | 0.429360316 | 0.41(0.23 to 0.72) | 0.04 | 0.37 | 91 (91) |
| Puerto Rico | 0.825525847 | 0.39(0.26 to 0.55) | 0.03 | 0.36 | 89 (88) |
| Saint Kitts and Nevis | 0.754987055 | 0.39(0.26 to 0.56) | 0.03 | 0.36 | 90 (89) |
| Uzbekistan | 0.662621694 | 0.38(0.24 to 0.58) | 0.03 | 0.35 | 88 (86) |
| Estonia | 0.844917787 | 0.37(0.25 to 0.54) | 0.03 | 0.34 | 87 (85) |
| Chile | 0.771514716 | 0.37(0.24 to 0.52) | 0.03 | 0.33 | 86 (84) |
| Turkey | 0.712692673 | 0.36(0.23 to 0.52) | 0.03 | 0.33 | 85 (83) |
| Georgia | 0.732473604 | 0.35(0.23 to 0.51) | 0.03 | 0.32 | 82 (80) |
| Panama | 0.708864828 | 0.36(0.23 to 0.52) | 0.03 | 0.32 | 84 (81) |
| Slovakia | 0.81061053 | 0.36(0.21 to 0.56) | 0.03 | 0.32 | 83 (82) |
| Croatia | 0.798341027 | 0.35(0.22 to 0.5) | 0.03 | 0.31 | 81 (79) |
| Ethiopia | 0.358823295 | 0.35(0.23 to 0.55) | 0.04 | 0.31 | 80 (78) |
| Bahamas | 0.805020668 | 0.34(0.23 to 0.49) | 0.03 | 0.3 | 78 (75) |
| Democratic Republic of the Congo | 0.383179849 | 0.34(0.13 to 0.87) | 0.04 | 0.3 | 77 (76) |

| Location | SDI | Rate of Deaths | Frontier Deaths | Effective difference | Effective difference rank (Age-standardized Deaths rank) |
|---|---|---|---|---|---|
| Nepal | 0.433174635 | 0.34(0.19 to 0.54) | 0.04 | 0.3 | 79 (77) |
| Nicaragua | 0.523958472 | 0.33(0.21 to 0.49) | 0.04 | 0.3 | 75 (73) |
| Sao Tome and Principe | 0.505413747 | 0.34(0.17 to 0.54) | 0.04 | 0.3 | 76 (74) |
| Bulgaria | 0.768150939 | 0.32(0.21 to 0.48) | 0.03 | 0.29 | 69 (67) |
| Germany | 0.902957091 | 0.33(0.22 to 0.47) | 0.03 | 0.29 | 74 (72) |
| Iceland | 0.87636168 | 0.33(0.22 to 0.47) | 0.03 | 0.29 | 73 (71) |
| Japan | 0.871241813 | 0.33(0.26 to 0.4) | 0.03 | 0.29 | 72 (70) |
| Singapore | 0.856097766 | 0.32(0.21 to 0.49) | 0.03 | 0.29 | 71 (68) |
| Tajikistan | 0.541511187 | 0.32(0.17 to 0.54) | 0.04 | 0.29 | 70 (69) |
| Barbados | 0.746748764 | 0.31(0.2 to 0.46) | 0.03 | 0.28 | 67 (63) |
| Burundi | 0.289374365 | 0.39(0.23 to 0.64) | 0.11 | 0.28 | 64 (87) |
| Finland | 0.859831368 | 0.31(0.2 to 0.44) | 0.03 | 0.28 | 63 (62) |
| Mexico | 0.664575304 | 0.32(0.27 to 0.38) | 0.03 | 0.28 | 68 (66) |
| Portugal | 0.744151851 | 0.31(0.21 to 0.45) | 0.03 | 0.28 | 65 (64) |
| United States Virgin Islands | 0.821830853 | 0.32(0.2 to 0.49) | 0.04 | 0.28 | 66 (65) |
| Austria | 0.853837004 | 0.3(0.2 to 0.43) | 0.03 | 0.27 | 61 (60) |

| Location | SDI | Rate of Deaths | Frontier Deaths | Effective difference | Effective difference rank (Age-standardized Deaths rank) |
|---|---|---|---|---|---|
| Greece | 0.791854408 | 0.3(0.21 to 0.43) | 0.03 | 0.27 | 60 (59) |
| Peru | 0.662054037 | 0.3(0.18 to 0.46) | 0.03 | 0.27 | 59 (58) |
| Spain | 0.769283698 | 0.31(0.2 to 0.45) | 0.03 | 0.27 | 62 (61) |
| Timor-Leste | 0.444667619 | 0.3(0.16 to 0.5) | 0.04 | 0.27 | 58 (57) |
| Colombia | 0.655442913 | 0.3(0.2 to 0.43) | 0.03 | 0.26 | 56 (55) |
| Lithuania | 0.856484049 | 0.29(0.2 to 0.41) | 0.03 | 0.26 | 54 (53) |
| Myanmar | 0.53390084 | 0.3(0.13 to 0.56) | 0.04 | 0.26 | 57 (56) |
| Pakistan | 0.504028689 | 0.3(0.22 to 0.39) | 0.04 | 0.26 | 55 (54) |
| Venezuela (Bolivarian Republic of) | 0.596513059 | 0.29(0.18 to 0.43) | 0.03 | 0.26 | 53 (52) |
| Antigua and Barbuda | 0.749886887 | 0.28(0.19 to 0.4) | 0.03 | 0.25 | 46 (46) |
| Kyrgyzstan | 0.603979328 | 0.29(0.18 to 0.43) | 0.03 | 0.25 | 49 (48) |
| Latvia | 0.830663516 | 0.29(0.2 to 0.39) | 0.03 | 0.25 | 50 (50) |
| Luxembourg | 0.884428955 | 0.29(0.18 to 0.41) | 0.03 | 0.25 | 52 (49) |
| Romania | 0.768453864 | 0.29(0.2 to 0.42) | 0.03 | 0.25 | 51 (51) |
| Suriname | 0.633665739 | 0.28(0.17 to 0.42) | 0.03 | 0.25 | 48 (47) |
| Trinidad and Tobago | 0.768763254 | 0.28(0.18 to 0.4) | 0.03 | 0.25 | 47 (45) |

| Location | SDI | Rate of Deaths | Frontier Deaths | Effective difference | Effective difference rank (Age-standardized Deaths rank) |
|---|---|---|---|---|---|
| Belize | 0.610229002 | 0.28(0.18 to 0.39) | 0.03 | 0.24 | 45 (44) |
| Saint Vincent and the Grenadines | 0.637195963 | 0.27(0.18 to 0.38) | 0.03 | 0.24 | 43 (43) |
| Switzerland | 0.933059111 | 0.26(0.17 to 0.37) | 0.02 | 0.24 | 44 (39) |
| Belgium | 0.853654016 | 0.26(0.17 to 0.38) | 0.03 | 0.23 | 41 (41) |
| Ireland | 0.87375385 | 0.27(0.18 to 0.38) | 0.04 | 0.23 | 42 (42) |
| Russian Federation | 0.808536005 | 0.26(0.22 to 0.3) | 0.03 | 0.23 | 40 (40) |
| Norway | 0.91613281 | 0.25(0.21 to 0.29) | 0.03 | 0.22 | 38 (37) |
| Paraguay | 0.635718099 | 0.25(0.16 to 0.37) | 0.03 | 0.22 | 39 (38) |
| Algeria | 0.659500924 | 0.24(0.15 to 0.37) | 0.04 | 0.21 | 35 (34) |
| Cyprus | 0.835630545 | 0.24(0.15 to 0.37) | 0.04 | 0.21 | 33 (33) |
| Guyana | 0.650812335 | 0.25(0.16 to 0.36) | 0.03 | 0.21 | 36 (35) |
| Netherlands | 0.888464256 | 0.24(0.16 to 0.33) | 0.03 | 0.21 | 34 (32) |
| Republic of Moldova | 0.732214875 | 0.25(0.17 to 0.35) | 0.04 | 0.21 | 37 (36) |
| Belarus | 0.784484711 | 0.24(0.15 to 0.34) | 0.03 | 0.2 | 31 (30) |
| Jordan | 0.725307227 | 0.23(0.14 to 0.36) | 0.03 | 0.2 | 30 (29) |
| Lebanon | 0.744746351 | 0.23(0.15 to 0.35) | 0.03 | 0.2 | 29 (28) |

| Location | SDI | Rate of Deaths | Frontier Deaths | Effective difference | Effective difference rank (Age-standardized Deaths rank) |
|---|---|---|---|---|---|
| Sweden | 0.886880299 | 0.24(0.19 to 0.29) | 0.03 | 0.2 | 32 (31) |
| Uruguay | 0.719283445 | 0.23(0.15 to 0.33) | 0.04 | 0.2 | 28 (27) |
| Czechia | 0.828450433 | 0.23(0.15 to 0.34) | 0.03 | 0.19 | 27 (26) |
| Papua New Guinea | 0.417797443 | 0.22(0.09 to 0.55) | 0.04 | 0.19 | 26 (25) |
| Israel | 0.809011652 | 0.21(0.14 to 0.3) | 0.03 | 0.18 | 25 (24) |
| Kuwait | 0.846651055 | 0.21(0.14 to 0.3) | 0.03 | 0.18 | 24 (23) |
| Bangladesh | 0.492420885 | 0.21(0.13 to 0.32) | 0.04 | 0.17 | 23 (22) |
| Italy | 0.805773534 | 0.2(0.17 to 0.24) | 0.03 | 0.17 | 21 (19) |
| Jamaica | 0.683263064 | 0.2(0.13 to 0.3) | 0.03 | 0.17 | 22 (21) |
| Malta | 0.801585034 | 0.2(0.13 to 0.3) | 0.04 | 0.17 | 20 (20) |
| Bermuda | 0.821365422 | 0.19(0.12 to 0.28) | 0.03 | 0.16 | 15 (14) |
| Cuba | 0.668729864 | 0.19(0.13 to 0.26) | 0.03 | 0.16 | 17 (16) |
| Denmark | 0.896424204 | 0.19(0.12 to 0.28) | 0.03 | 0.16 | 16 (15) |
| Dominican Republic | 0.619388201 | 0.19(0.12 to 0.29) | 0.03 | 0.16 | 18 (17) |
| El Salvador | 0.563775188 | 0.2(0.13 to 0.29) | 0.04 | 0.16 | 19 (18) |
| Tunisia | 0.682432216 | 0.18(0.1 to 0.29) | 0.03 | 0.15 | 13 (12) |

| Location | SDI | Rate of Deaths | Frontier Deaths | Effective difference | Effective difference rank (Age-standardized Deaths rank) |
|---|---|---|---|---|---|
| Yemen | 0.450376375 | 0.18(0.09 to 0.37) | 0.04 | 0.15 | 14 (13) |
| Hungary | 0.790754768 | 0.17(0.11 to 0.26) | 0.03 | 0.14 | 11 (10) |
| Poland | 0.812042809 | 0.18(0.15 to 0.21) | 0.03 | 0.14 | 12 (11) |
| San Marino | 0.888005474 | 0.17(0.09 to 0.29) | 0.03 | 0.14 | 10 (9) |
| Brazil | 0.653043887 | 0.17(0.14 to 0.19) | 0.03 | 0.13 | 9 (8) |
| Saint Lucia | 0.672509735 | 0.16(0.11 to 0.23) | 0.03 | 0.12 | 8 (7) |
| Sri Lanka | 0.701534935 | 0.15(0.08 to 0.24) | 0.03 | 0.11 | 7 (6) |
| Haiti | 0.448278285 | 0.14(0.06 to 0.29) | 0.04 | 0.1 | 6 (5) |
| Argentina | 0.723122973 | 0.11(0.07 to 0.16) | 0.03 | 0.08 | 4 (3) |
| Ukraine | 0.760773913 | 0.12(0.08 to 0.15) | 0.03 | 0.08 | 5 (4) |
| Mauritius | 0.718260446 | 0.09(0.07 to 0.13) | 0.03 | 0.06 | 3 (2) |
| Somalia | 0.077688109 | 1.2(0.58 to 2.29) | 1.16 | 0.04 | 2 (179) |
| Morocco | 0.562698301 | 0.06(0.04 to 0.09) | 0.04 | 0.03 | 1 (1) |

## Table S10 Projected Absolute and Age-Standardized Death Rates (ASDR) for NALC in Men and Women Globally through 2045

| year | Deaths | | | ASDR | | |
|---|---|---|---|---|---|---|
| | Both | Women | Men | Both | Women | Women |
| 1992 | 15660 | 8019 | 7641 | 0.38 | 0.36 | 0.40 |
| 1993 | 16198 | 8253 | 7945 | 0.39 | 0.36 | 0.41 |
| 1994 | 16831 | 8557 | 8274 | 0.39 | 0.37 | 0.42 |
| 1995 | 17625 | 8936 | 8689 | 0.40 | 0.38 | 0.43 |
| 1996 | 18300 | 9253 | 9047 | 0.41 | 0.38 | 0.44 |
| 1997 | 18843 | 9520 | 9322 | 0.41 | 0.39 | 0.44 |
| 1998 | 19626 | 9863 | 9763 | 0.42 | 0.39 | 0.45 |
| 1999 | 20442 | 10269 | 10173 | 0.43 | 0.40 | 0.46 |
| 2000 | 21185 | 10603 | 10581 | 0.43 | 0.40 | 0.47 |
| 2001 | 21722 | 10869 | 10854 | 0.43 | 0.40 | 0.47 |
| 2002 | 22221 | 11131 | 11090 | 0.43 | 0.40 | 0.47 |
| 2003 | 22547 | 11289 | 11258 | 0.43 | 0.40 | 0.46 |
| 2004 | 22831 | 11430 | 11401 | 0.42 | 0.39 | 0.46 |
| 2005 | 23380 | 11714 | 11666 | 0.42 | 0.39 | 0.46 |

| year | Deaths | | | ASDR | | |
|---|---|---|---|---|---|---|
| | Both | Women | Men | Both | Women | Women |
| 2006 | 23983 | 12051 | 11932 | 0.42 | 0.39 | 0.46 |
| 2007 | 24862 | 12506 | 12355 | 0.43 | 0.40 | 0.46 |
| 2008 | 26104 | 13132 | 12971 | 0.44 | 0.41 | 0.47 |
| 2009 | 27181 | 13683 | 13498 | 0.44 | 0.41 | 0.48 |
| 2010 | 28259 | 14207 | 14053 | 0.45 | 0.42 | 0.48 |
| 2011 | 29164 | 14663 | 14501 | 0.45 | 0.42 | 0.48 |
| 2012 | 30276 | 15237 | 15039 | 0.45 | 0.42 | 0.49 |
| 2013 | 31821 | 16020 | 15801 | 0.46 | 0.43 | 0.50 |
| 2014 | 33497 | 16868 | 16628 | 0.48 | 0.44 | 0.51 |
| 2015 | 34802 | 17492 | 17310 | 0.48 | 0.45 | 0.52 |
| 2016 | 35977 | 18174 | 17803 | 0.48 | 0.45 | 0.52 |
| 2017 | 37019 | 18758 | 18261 | 0.48 | 0.45 | 0.51 |
| 2018 | 38188 | 19315 | 18873 | 0.48 | 0.45 | 0.52 |
| 2019 | 39075 | 19704 | 19371 | 0.48 | 0.45 | 0.52 |
| 2020 | 39930 | 19930 | 19999 | 0.48 | 0.44 | 0.52 |

| year | Deaths | | | ASDR | | |
|---|---|---|---|---|---|---|
| | Both | Women | Men | Both | Women | Women |
| 2021 | 40925 | 20456 | 20469 | 0.48 | 0.44 | 0.52 |
| 2022 | 42202 | 21605 | 20596 | 0.49 | 0.45 | 0.52 |
| 2023 | 43510 | 22297 | 21214 | 0.49 | 0.46 | 0.52 |
| 2024 | 44961 | 23059 | 21903 | 0.49 | 0.46 | 0.52 |
| 2025 | 46284 | 23755 | 22529 | 0.49 | 0.46 | 0.52 |
| 2026 | 47632 | 24467 | 23165 | 0.49 | 0.46 | 0.52 |
| 2027 | 49034 | 25209 | 23825 | 0.49 | 0.46 | 0.52 |
| 2028 | 50505 | 25990 | 24514 | 0.49 | 0.46 | 0.52 |
| 2029 | 51993 | 26783 | 25211 | 0.49 | 0.46 | 0.52 |
| 2030 | 53317 | 27497 | 25821 | 0.49 | 0.46 | 0.52 |
| 2031 | 54639 | 28211 | 26428 | 0.49 | 0.46 | 0.52 |
| 2032 | 55987 | 28942 | 27046 | 0.49 | 0.46 | 0.52 |
| 2033 | 57385 | 29700 | 27685 | 0.49 | 0.46 | 0.52 |
| 2034 | 58774 | 30454 | 28320 | 0.49 | 0.46 | 0.52 |
| 2035 | 59959 | 31107 | 28852 | 0.48 | 0.45 | 0.51 |

| year | Deaths | | | ASDR | | |
|---|---|---|---|---|---|---|
| | Both | Women | Men | Both | Women | Women |
| 2036 | 61113 | 31743 | 29371 | 0.48 | 0.45 | 0.51 |
| 2037 | 62269 | 32380 | 29890 | 0.48 | 0.45 | 0.51 |
| 2038 | 63463 | 33038 | 30426 | 0.47 | 0.45 | 0.50 |
| 2039 | 64634 | 33682 | 30952 | 0.47 | 0.45 | 0.50 |
| 2040 | 65741 | 34293 | 31448 | 0.47 | 0.44 | 0.50 |
| 2041 | 66804 | 34878 | 31926 | 0.47 | 0.44 | 0.49 |
| 2042 | 67849 | 35454 | 32395 | 0.46 | 0.44 | 0.49 |
| 2043 | 68923 | 36044 | 32879 | 0.46 | 0.44 | 0.49 |
| 2044 | 69981 | 36626 | 33355 | 0.46 | 0.44 | 0.48 |
| 2045 | 71001 | 37186 | 33816 | 0.46 | 0.43 | 0.48 |

## Table S11 Projected Absolute Death Numbers for NALC in Women Globally by Age Group through 2045

| | Deaths-number | | | | | | | | | | | | | | | | |
|---|---|---|---|---|---|---|---|---|---|---|---|---|---|---|---|---|---|
| **year** | **15 to 19** | **20 to 24** | **25 to 29** | **30 to 34** | **35 to 39** | **40 to 44** | **45 to 49** | **50 to 54** | **55 to 59** | **60 to 64** | **65 to 69** | **70 to 74** | **75 to 79** | **80 to 84** | **85 to 89** | **90 to 94** | **95 plus** |
| 1992 | 66.57 | 73.78 | 86.74 | 100.48 | 144.38 | 215.94 | 301.70 | 475.65 | 694.96 | 985.34 | 1192.94 | 1207.29 | 1111.68 | 784.16 | 413.97 | 134.76 | 28.43 |
| 1993 | 67.73 | 75.26 | 90.34 | 105.66 | 144.98 | 226.81 | 312.91 | 477.72 | 704.55 | 1008.41 | 1230.14 | 1268.29 | 1122.88 | 807.94 | 435.49 | 143.72 | 30.60 |
| 1994 | 68.34 | 77.25 | 93.83 | 113.54 | 147.49 | 239.64 | 334.32 | 486.78 | 714.93 | 1038.23 | 1272.31 | 1339.82 | 1146.22 | 835.78 | 462.57 | 153.79 | 32.29 |
| 1995 | 69.76 | 79.66 | 96.15 | 120.26 | 147.52 | 249.19 | 350.30 | 491.15 | 726.60 | 1074.13 | 1328.47 | 1414.85 | 1218.84 | 872.74 | 494.96 | 165.89 | 35.24 |
| 1996 | 71.36 | 80.17 | 97.83 | 125.82 | 147.85 | 253.83 | 369.07 | 502.84 | 732.95 | 1097.44 | 1374.82 | 1466.85 | 1289.28 | 905.47 | 522.20 | 176.69 | 38.26 |
| 1997 | 73.42 | 79.77 | 98.86 | 130.56 | 150.41 | 257.80 | 384.56 | 521.61 | 735.50 | 1110.11 | 1409.46 | 1513.10 | 1355.31 | 928.48 | 542.22 | 187.63 | 41.60 |
| 1998 | 74.66 | 79.90 | 98.57 | 134.09 | 158.80 | 262.24 | 407.78 | 542.81 | 740.92 | 1127.59 | 1446.41 | 1574.34 | 1437.57 | 959.77 | 571.92 | 200.89 | 44.97 |
| 1999 | 76.45 | 81.90 | 99.83 | 135.90 | 168.66 | 260.28 | 429.58 | 575.61 | 754.88 | 1142.47 | 1487.34 | 1649.85 | 1536.88 | 998.33 | 605.27 | 217.19 | 48.83 |
| 2000 | 78.32 | 82.72 | 100.15 | 136.42 | 178.95 | 256.04 | 443.72 | 607.20 | 769.10 | 1153.95 | 1514.65 | 1704.03 | 1613.14 | 1054.88 | 627.23 | 230.10 | 52.76 |
| 2001 | 80.04 | 83.38 | 100.07 | 135.31 | 186.74 | 254.63 | 443.59 | 638.88 | 785.73 | 1158.60 | 1539.66 | 1743.73 | 1665.71 | 1113.26 | 641.82 | 240.62 | 56.74 |
| 2002 | 81.73 | 83.46 | 96.40 | 129.09 | 187.96 | 257.23 | 426.96 | 658.71 | 822.49 | 1170.63 | 1566.82 | 1771.35 | 1717.11 | 1187.26 | 660.69 | 252.70 | 60.45 |
| 2003 | 84.24 | 83.27 | 91.80 | 122.95 | 185.06 | 259.06 | 406.65 | 677.81 | 851.34 | 1167.40 | 1577.58 | 1795.08 | 1751.20 | 1252.65 | 659.57 | 259.69 | 64.11 |
| 2004 | 85.29 | 84.09 | 90.32 | 121.05 | 182.04 | 263.26 | 396.95 | 685.10 | 874.76 | 1160.65 | 1564.08 | 1806.67 | 1793.31 | 1320.13 | 668.18 | 266.34 | 68.01 |
| 2005 | 85.92 | 85.39 | 89.64 | 118.13 | 181.47 | 271.96 | 392.28 | 705.32 | 909.32 | 1169.62 | 1576.91 | 1841.13 | 1857.77 | 1380.37 | 701.02 | 274.64 | 72.80 |

| | Deaths-number | | | | | | | | | | | | | | | | |
|---|---|---|---|---|---|---|---|---|---|---|---|---|---|---|---|---|---|
| year | 15 to 19 | 20 to 24 | 25 to 29 | 30 to 34 | 35 to 39 | 40 to 44 | 45 to 49 | 50 to 54 | 55 to 59 | 60 to 64 | 65 to 69 | 70 to 74 | 75 to 79 | 80 to 84 | 85 to 89 | 90 to 94 | 95 plus |
| 2006 | 85.09 | 86.81 | 89.02 | 116.12 | 181.90 | 284.31 | 390.71 | 720.73 | 957.47 | 1188.86 | 1586.28 | 1884.84 | 1917.79 | 1437.83 | 754.42 | 290.57 | 78.62 |
| 2007 | 84.80 | 88.83 | 89.31 | 115.36 | 182.16 | 296.62 | 400.08 | 732.89 | 1003.37 | 1241.10 | 1610.95 | 1952.68 | 1986.58 | 1517.65 | 817.75 | 302.20 | 83.96 |
| 2008 | 85.42 | 91.10 | 92.43 | 117.55 | 184.59 | 305.19 | 427.29 | 742.84 | 1056.73 | 1311.18 | 1652.29 | 2023.32 | 2092.90 | 1635.28 | 901.89 | 320.25 | 92.20 |
| 2009 | 86.11 | 92.90 | 96.68 | 119.57 | 186.03 | 302.99 | 456.82 | 739.84 | 1084.91 | 1381.06 | 1698.87 | 2073.38 | 2166.26 | 1761.59 | 992.96 | 342.19 | 100.78 |
| 2010 | 86.96 | 94.32 | 99.31 | 120.52 | 184.46 | 302.04 | 483.42 | 742.86 | 1118.97 | 1452.04 | 1735.63 | 2119.23 | 2234.17 | 1878.53 | 1069.28 | 375.28 | 109.59 |
| 2011 | 87.07 | 93.07 | 100.02 | 119.82 | 180.10 | 299.69 | 501.94 | 754.61 | 1144.61 | 1541.86 | 1786.13 | 2157.89 | 2312.48 | 1941.04 | 1118.03 | 407.67 | 117.27 |
| 2012 | 86.14 | 91.96 | 102.89 | 121.97 | 180.24 | 301.86 | 524.22 | 779.76 | 1174.57 | 1631.28 | 1883.95 | 2209.12 | 2390.86 | 2018.83 | 1173.07 | 443.44 | 122.64 |
| 2013 | 87.87 | 91.92 | 107.07 | 127.38 | 183.45 | 308.79 | 541.22 | 833.21 | 1190.55 | 1727.78 | 1997.20 | 2252.38 | 2461.32 | 2198.80 | 1284.32 | 494.58 | 132.01 |
| 2014 | 89.01 | 90.27 | 109.98 | 132.07 | 185.78 | 314.28 | 557.45 | 910.04 | 1211.81 | 1840.14 | 2148.38 | 2304.56 | 2538.05 | 2362.90 | 1389.42 | 542.83 | 141.49 |
| 2015 | 89.95 | 90.18 | 111.76 | 136.25 | 188.55 | 316.96 | 569.84 | 980.07 | 1238.65 | 1959.62 | 2311.80 | 2365.14 | 2608.43 | 2380.92 | 1416.51 | 570.74 | 156.21 |
| 2016 | 91.21 | 91.53 | 114.22 | 141.92 | 193.98 | 321.72 | 580.19 | 1026.82 | 1276.30 | 2033.77 | 2493.83 | 2462.84 | 2673.68 | 2426.44 | 1468.20 | 605.68 | 172.05 |
| 2017 | 92.45 | 93.38 | 115.13 | 147.32 | 200.17 | 328.00 | 584.42 | 1035.06 | 1325.09 | 2068.30 | 2591.55 | 2569.64 | 2707.20 | 2511.51 | 1552.38 | 646.64 | 190.00 |
| 2018 | 92.91 | 94.20 | 114.71 | 151.44 | 204.35 | 330.93 | 588.07 | 1046.68 | 1389.78 | 2097.09 | 2716.75 | 2683.66 | 2757.14 | 2561.36 | 1597.06 | 681.82 | 207.55 |
| 2019 | 93.83 | 95.18 | 115.41 | 153.59 | 209.27 | 331.34 | 586.28 | 1038.90 | 1452.42 | 2102.16 | 2803.91 | 2804.55 | 2808.99 | 2553.90 | 1614.94 | 715.46 | 223.39 |
| 2020 | 95.78 | 97.32 | 115.84 | 154.89 | 215.51 | 332.93 | 578.47 | 1021.15 | 1512.28 | 2086.51 | 2842.00 | 2861.96 | 2825.15 | 2562.86 | 1645.00 | 746.35 | 236.44 |

| | Deaths-number | | | | | | | | | | | | | | | | |
|---|---|---|---|---|---|---|---|---|---|---|---|---|---|---|---|---|---|
| year | 15 to 19 | 20 to 24 | 25 to 29 | 30 to 34 | 35 to 39 | 40 to 44 | 45 to 49 | 50 to 54 | 55 to 59 | 60 to 64 | 65 to 69 | 70 to 74 | 75 to 79 | 80 to 84 | 85 to 89 | 90 to 94 | 95 plus |
| 2021 | 99.66 | 101.64 | 117.76 | 157.94 | 222.64 | 336.58 | 573.09 | 1028.13 | 1552.58 | 2110.24 | 2919.52 | 2984.28 | 2891.10 | 2633.47 | 1694.07 | 781.51 | 251.90 |
| 2022 | 103.42 | 98.90 | 112.88 | 153.35 | 221.63 | 343.57 | 581.73 | 1087.37 | 1625.96 | 2206.99 | 3051.50 | 3316.79 | 3038.13 | 2754.25 | 1814.78 | 839.77 | 254.26 |
| 2023 | 106.77 | 100.46 | 112.74 | 152.51 | 225.58 | 351.10 | 579.11 | 1098.14 | 1650.83 | 2280.56 | 3109.08 | 3491.26 | 3212.59 | 2797.54 | 1879.11 | 879.82 | 269.48 |
| 2024 | 110.26 | 102.13 | 112.77 | 151.40 | 229.59 | 357.54 | 579.53 | 1103.07 | 1677.75 | 2380.62 | 3142.46 | 3667.86 | 3420.89 | 2858.57 | 1952.15 | 925.08 | 286.86 |
| 2025 | 113.33 | 104.51 | 113.66 | 149.94 | 232.54 | 363.53 | 585.97 | 1099.38 | 1696.70 | 2480.19 | 3166.10 | 3793.20 | 3656.72 | 2918.73 | 2016.45 | 958.40 | 305.38 |
| 2026 | 116.37 | 106.94 | 114.79 | 148.81 | 233.91 | 369.59 | 595.87 | 1092.55 | 1718.25 | 2576.63 | 3188.55 | 3898.86 | 3890.83 | 3014.49 | 2080.08 | 995.89 | 324.14 |
| 2027 | 119.43 | 109.63 | 116.02 | 148.35 | 233.21 | 375.51 | 608.83 | 1086.34 | 1739.27 | 2644.16 | 3240.82 | 3992.64 | 4108.94 | 3173.11 | 2134.47 | 1035.69 | 343.06 |
| 2028 | 122.34 | 112.58 | 117.35 | 148.17 | 231.73 | 381.36 | 622.14 | 1081.58 | 1751.01 | 2679.52 | 3359.16 | 4074.20 | 4317.73 | 3370.29 | 2182.83 | 1075.73 | 362.76 |
| 2029 | 125.02 | 115.66 | 118.81 | 148.21 | 229.80 | 387.21 | 633.38 | 1082.23 | 1752.47 | 2716.42 | 3513.63 | 4116.82 | 4518.56 | 3591.70 | 2235.21 | 1114.21 | 383.31 |
| 2030 | 126.96 | 118.22 | 120.87 | 148.59 | 226.56 | 390.70 | 641.80 | 1091.07 | 1742.45 | 2742.48 | 3655.39 | 4139.99 | 4670.35 | 3840.84 | 2286.61 | 1153.78 | 399.87 |
| 2031 | 128.78 | 120.72 | 122.97 | 149.29 | 223.83 | 391.50 | 650.26 | 1106.29 | 1727.41 | 2772.50 | 3791.47 | 4161.55 | 4797.06 | 4086.90 | 2368.31 | 1194.36 | 417.94 |
| 2032 | 130.36 | 123.24 | 125.36 | 150.10 | 222.13 | 388.79 | 658.42 | 1127.08 | 1713.35 | 2801.35 | 3883.70 | 4224.20 | 4909.08 | 4314.40 | 2503.60 | 1230.01 | 436.54 |
| 2033 | 131.48 | 125.59 | 128.03 | 151.05 | 220.85 | 384.79 | 666.36 | 1148.37 | 1701.58 | 2814.84 | 3928.14 | 4376.32 | 5005.78 | 4529.36 | 2669.10 | 1263.02 | 455.10 |
| 2034 | 132.20 | 127.69 | 130.84 | 152.13 | 219.89 | 380.06 | 674.24 | 1165.66 | 1698.40 | 2811.52 | 3974.87 | 4575.35 | 5052.53 | 4735.37 | 2851.09 | 1298.53 | 473.20 |
| 2035 | 131.87 | 128.98 | 133.00 | 153.91 | 219.47 | 373.35 | 678.36 | 1178.21 | 1708.32 | 2790.43 | 4008.38 | 4756.68 | 5074.16 | 4894.28 | 3052.28 | 1332.35 | 492.74 |

| year | Deaths-number | | | | | | | | | | | | | | | | |
|---|---|---|---|---|---|---|---|---|---|---|---|---|---|---|---|---|---|
| | 15 to 19 | 20 to 24 | 25 to 29 | 30 to 34 | 35 to 39 | 40 to 44 | 45 to 49 | 50 to 54 | 55 to 59 | 60 to 64 | 65 to 69 | 70 to 74 | 75 to 79 | 80 to 84 | 85 to 89 | 90 to 94 | 95 plus |
| 2036 | 131.17 | 130.15 | 135.08 | 155.73 | 219.49 | 367.51 | 677.73 | 1190.72 | 1728.18 | 2761.16 | 4047.39 | 4928.73 | 5093.96 | 5026.12 | 3249.73 | 1386.09 | 513.65 |
| 2037 | 130.32 | 131.06 | 137.16 | 157.90 | 219.69 | 363.38 | 670.99 | 1202.60 | 1756.65 | 2733.46 | 4084.14 | 5041.77 | 5167.91 | 5143.09 | 3431.22 | 1475.32 | 532.88 |
| 2038 | 129.83 | 131.51 | 139.05 | 160.42 | 220.06 | 359.95 | 662.01 | 1213.98 | 1785.69 | 2709.44 | 4097.84 | 5092.64 | 5357.33 | 5244.02 | 3600.17 | 1582.34 | 551.24 |
| 2039 | 129.82 | 131.56 | 140.65 | 163.09 | 220.62 | 357.04 | 651.80 | 1225.15 | 1808.26 | 2699.23 | 4086.50 | 5146.86 | 5603.83 | 5289.43 | 3762.07 | 1696.17 | 570.25 |
| 2040 | 130.29 | 131.24 | 142.06 | 165.74 | 223.13 | 356.52 | 640.82 | 1234.02 | 1829.22 | 2717.35 | 4059.40 | 5197.74 | 5838.34 | 5318.27 | 3897.58 | 1821.38 | 589.46 |
| 2041 | 131.19 | 130.54 | 143.34 | 168.32 | 225.69 | 356.72 | 631.33 | 1234.21 | 1850.21 | 2751.52 | 4020.11 | 5255.72 | 6059.24 | 5346.03 | 4011.78 | 1944.10 | 618.01 |
| 2042 | 132.25 | 129.69 | 144.34 | 170.88 | 228.77 | 357.19 | 624.77 | 1223.19 | 1870.29 | 2799.64 | 3983.04 | 5310.10 | 6205.54 | 5437.99 | 4115.66 | 2057.87 | 662.27 |
| 2043 | 133.33 | 129.21 | 144.82 | 173.20 | 232.34 | 357.97 | 619.43 | 1208.05 | 1889.64 | 2848.70 | 3951.36 | 5333.76 | 6276.67 | 5661.94 | 4207.68 | 2162.99 | 713.31 |
| 2044 | 134.41 | 129.20 | 144.86 | 175.16 | 236.14 | 359.05 | 615.00 | 1190.63 | 1908.72 | 2887.35 | 3940.20 | 5324.25 | 6353.42 | 5944.96 | 4252.60 | 2264.60 | 765.17 |
| 2045 | 135.51 | 129.66 | 144.49 | 176.87 | 239.90 | 360.01 | 611.99 | 1170.10 | 1924.82 | 2922.19 | 3960.23 | 5288.78 | 6454.47 | 6211.55 | 4267.91 | 2369.52 | 817.66 |

## Table S12 Projected age-standardized death rate (ASDR) for NALC in Women Globally by Age Group through 2045

| | ASDR | | | | | | | | | | | | | | | | |
|---|---|---|---|---|---|---|---|---|---|---|---|---|---|---|---|---|---|
| year | 15 to 19 | 20 to 24 | 25 to 29 | 30 to 34 | 35 to 39 | 40 to 44 | 45 to 49 | 50 to 54 | 55 to 59 | 60 to 64 | 65 to 69 | 70 to 74 | 75 to 79 | 80 to 84 | 85 to 89 | 90 to 94 | 95 plus |
| 1992 | 0.03 | 0.03 | 0.04 | 0.05 | 0.08 | 0.14 | 0.25 | 0.45 | 0.73 | 1.17 | 1.71 | 2.38 | 3.07 | 3.35 | 3.77 | 3.94 | 3.45 |
| 1993 | 0.03 | 0.03 | 0.04 | 0.05 | 0.08 | 0.14 | 0.25 | 0.45 | 0.73 | 1.18 | 1.72 | 2.39 | 3.12 | 3.36 | 3.82 | 3.95 | 3.56 |
| 1994 | 0.03 | 0.03 | 0.04 | 0.05 | 0.08 | 0.15 | 0.26 | 0.45 | 0.73 | 1.20 | 1.74 | 2.43 | 3.17 | 3.40 | 3.91 | 3.99 | 3.59 |
| 1995 | 0.03 | 0.03 | 0.04 | 0.06 | 0.08 | 0.15 | 0.26 | 0.46 | 0.73 | 1.23 | 1.78 | 2.49 | 3.29 | 3.50 | 4.06 | 4.09 | 3.71 |
| 1996 | 0.03 | 0.03 | 0.04 | 0.06 | 0.08 | 0.15 | 0.26 | 0.46 | 0.73 | 1.24 | 1.82 | 2.50 | 3.35 | 3.63 | 4.14 | 4.15 | 3.77 |
| 1997 | 0.03 | 0.03 | 0.04 | 0.06 | 0.08 | 0.15 | 0.26 | 0.46 | 0.72 | 1.23 | 1.84 | 2.52 | 3.37 | 3.73 | 4.17 | 4.19 | 3.87 |
| 1998 | 0.03 | 0.03 | 0.04 | 0.06 | 0.08 | 0.15 | 0.27 | 0.46 | 0.73 | 1.22 | 1.86 | 2.55 | 3.41 | 3.86 | 4.25 | 4.28 | 3.93 |
| 1999 | 0.03 | 0.03 | 0.04 | 0.06 | 0.08 | 0.15 | 0.28 | 0.46 | 0.74 | 1.22 | 1.89 | 2.61 | 3.50 | 3.97 | 4.38 | 4.43 | 4.00 |
| 2000 | 0.03 | 0.03 | 0.04 | 0.06 | 0.08 | 0.14 | 0.27 | 0.47 | 0.75 | 1.21 | 1.90 | 2.64 | 3.54 | 4.07 | 4.44 | 4.51 | 4.05 |
| 2001 | 0.03 | 0.03 | 0.04 | 0.06 | 0.08 | 0.14 | 0.27 | 0.47 | 0.75 | 1.20 | 1.90 | 2.65 | 3.55 | 4.10 | 4.51 | 4.52 | 4.08 |
| 2002 | 0.03 | 0.03 | 0.04 | 0.05 | 0.08 | 0.14 | 0.25 | 0.47 | 0.76 | 1.21 | 1.89 | 2.65 | 3.55 | 4.16 | 4.62 | 4.57 | 4.07 |
| 2003 | 0.03 | 0.03 | 0.04 | 0.05 | 0.08 | 0.13 | 0.24 | 0.46 | 0.75 | 1.20 | 1.86 | 2.65 | 3.52 | 4.17 | 4.60 | 4.53 | 4.08 |
| 2004 | 0.03 | 0.03 | 0.04 | 0.05 | 0.08 | 0.13 | 0.23 | 0.46 | 0.73 | 1.18 | 1.81 | 2.63 | 3.51 | 4.20 | 4.57 | 4.48 | 4.09 |
| 2005 | 0.03 | 0.03 | 0.04 | 0.05 | 0.08 | 0.13 | 0.22 | 0.45 | 0.73 | 1.18 | 1.79 | 2.64 | 3.55 | 4.22 | 4.61 | 4.49 | 4.14 |

| | ASDR | | | | | | | | | | | | | | | | |
|---|---|---|---|---|---|---|---|---|---|---|---|---|---|---|---|---|---|
| year | 15 to 19 | 20 to 24 | 25 to 29 | 30 to 34 | 35 to 39 | 40 to 44 | 45 to 49 | 50 to 54 | 55 to 59 | 60 to 64 | 65 to 69 | 70 to 74 | 75 to 79 | 80 to 84 | 85 to 89 | 90 to 94 | 95 plus |
| 2006 | 0.03 | 0.03 | 0.03 | 0.05 | 0.08 | 0.13 | 0.22 | 0.45 | 0.73 | 1.18 | 1.78 | 2.64 | 3.58 | 4.24 | 4.69 | 4.67 | 4.23 |
| 2007 | 0.03 | 0.03 | 0.03 | 0.05 | 0.08 | 0.13 | 0.22 | 0.45 | 0.74 | 1.18 | 1.79 | 2.67 | 3.64 | 4.32 | 4.79 | 4.79 | 4.29 |
| 2008 | 0.03 | 0.03 | 0.03 | 0.05 | 0.08 | 0.14 | 0.23 | 0.45 | 0.74 | 1.20 | 1.82 | 2.70 | 3.76 | 4.51 | 4.98 | 4.99 | 4.48 |
| 2009 | 0.03 | 0.03 | 0.04 | 0.05 | 0.08 | 0.13 | 0.23 | 0.44 | 0.74 | 1.19 | 1.86 | 2.70 | 3.82 | 4.70 | 5.20 | 5.16 | 4.65 |
| 2010 | 0.03 | 0.03 | 0.04 | 0.05 | 0.08 | 0.13 | 0.24 | 0.44 | 0.73 | 1.19 | 1.88 | 2.70 | 3.86 | 4.88 | 5.35 | 5.33 | 4.82 |
| 2011 | 0.03 | 0.03 | 0.04 | 0.05 | 0.07 | 0.13 | 0.24 | 0.43 | 0.73 | 1.20 | 1.90 | 2.71 | 3.89 | 4.91 | 5.37 | 5.40 | 4.98 |
| 2012 | 0.03 | 0.03 | 0.04 | 0.05 | 0.07 | 0.13 | 0.24 | 0.44 | 0.73 | 1.22 | 1.91 | 2.74 | 3.91 | 4.99 | 5.42 | 5.49 | 5.04 |
| 2013 | 0.03 | 0.03 | 0.04 | 0.05 | 0.07 | 0.13 | 0.25 | 0.45 | 0.73 | 1.25 | 1.94 | 2.77 | 3.91 | 5.31 | 5.71 | 5.74 | 5.25 |
| 2014 | 0.03 | 0.03 | 0.04 | 0.05 | 0.07 | 0.13 | 0.25 | 0.47 | 0.74 | 1.29 | 1.97 | 2.80 | 3.93 | 5.58 | 5.97 | 5.96 | 5.39 |
| 2015 | 0.03 | 0.03 | 0.04 | 0.05 | 0.08 | 0.13 | 0.25 | 0.49 | 0.74 | 1.31 | 2.02 | 2.84 | 3.95 | 5.48 | 5.89 | 5.97 | 5.59 |
| 2016 | 0.03 | 0.03 | 0.04 | 0.05 | 0.08 | 0.13 | 0.25 | 0.50 | 0.75 | 1.33 | 2.06 | 2.90 | 3.97 | 5.42 | 5.92 | 6.06 | 5.76 |
| 2017 | 0.03 | 0.03 | 0.04 | 0.05 | 0.08 | 0.14 | 0.25 | 0.49 | 0.76 | 1.32 | 2.06 | 2.88 | 3.97 | 5.43 | 6.09 | 6.19 | 5.97 |
| 2018 | 0.03 | 0.03 | 0.04 | 0.05 | 0.08 | 0.14 | 0.25 | 0.49 | 0.77 | 1.32 | 2.08 | 2.87 | 3.99 | 5.37 | 6.10 | 6.25 | 6.15 |
| 2019 | 0.03 | 0.03 | 0.04 | 0.05 | 0.08 | 0.14 | 0.25 | 0.48 | 0.77 | 1.31 | 2.08 | 2.83 | 4.01 | 5.21 | 5.99 | 6.29 | 6.25 |
| 2020 | 0.03 | 0.03 | 0.04 | 0.05 | 0.08 | 0.14 | 0.25 | 0.46 | 0.78 | 1.29 | 2.02 | 2.75 | 3.99 | 5.11 | 5.93 | 6.35 | 6.27 |

| | ASDR | | | | | | | | | | | | | | | | |
|---|---|---|---|---|---|---|---|---|---|---|---|---|---|---|---|---|---|
| **year** | **15 to 19** | **20 to 24** | **25 to 29** | **30 to 34** | **35 to 39** | **40 to 44** | **45 to 49** | **50 to 54** | **55 to 59** | **60 to 64** | **65 to 69** | **70 to 74** | **75 to 79** | **80 to 84** | **85 to 89** | **90 to 94** | **95 plus** |
| 2021 | 0.03 | 0.03 | 0.04 | 0.05 | 0.08 | 0.14 | 0.24 | 0.46 | 0.77 | 1.28 | 2.03 | 2.73 | 4.01 | 5.17 | 5.95 | 6.48 | 6.40 |
| 2022 | 0.03 | 0.03 | 0.04 | 0.05 | 0.08 | 0.14 | 0.25 | 0.47 | 0.78 | 1.31 | 2.04 | 2.90 | 4.06 | 5.33 | 6.15 | 6.68 | 6.54 |
| 2023 | 0.03 | 0.03 | 0.04 | 0.05 | 0.08 | 0.14 | 0.25 | 0.47 | 0.79 | 1.31 | 2.04 | 2.93 | 4.09 | 5.36 | 6.20 | 6.80 | 6.64 |
| 2024 | 0.04 | 0.03 | 0.04 | 0.05 | 0.08 | 0.14 | 0.24 | 0.47 | 0.79 | 1.31 | 2.03 | 2.96 | 4.11 | 5.39 | 6.25 | 6.92 | 6.75 |
| 2025 | 0.04 | 0.04 | 0.04 | 0.05 | 0.08 | 0.14 | 0.24 | 0.47 | 0.79 | 1.32 | 2.04 | 2.95 | 4.13 | 5.41 | 6.26 | 6.92 | 6.84 |
| 2026 | 0.04 | 0.04 | 0.04 | 0.05 | 0.08 | 0.14 | 0.24 | 0.47 | 0.78 | 1.32 | 2.04 | 2.94 | 4.15 | 5.42 | 6.28 | 6.93 | 6.94 |
| 2027 | 0.04 | 0.04 | 0.04 | 0.05 | 0.08 | 0.14 | 0.24 | 0.47 | 0.78 | 1.32 | 2.05 | 2.94 | 4.17 | 5.44 | 6.29 | 6.93 | 7.04 |
| 2028 | 0.04 | 0.04 | 0.04 | 0.05 | 0.08 | 0.14 | 0.24 | 0.47 | 0.78 | 1.32 | 2.05 | 2.93 | 4.19 | 5.45 | 6.30 | 6.94 | 7.13 |
| 2029 | 0.04 | 0.04 | 0.04 | 0.05 | 0.08 | 0.14 | 0.24 | 0.47 | 0.77 | 1.32 | 2.05 | 2.92 | 4.21 | 5.47 | 6.32 | 6.94 | 7.23 |
| 2030 | 0.04 | 0.04 | 0.04 | 0.05 | 0.08 | 0.13 | 0.24 | 0.46 | 0.77 | 1.32 | 2.05 | 2.92 | 4.20 | 5.48 | 6.32 | 6.95 | 7.22 |
| 2031 | 0.04 | 0.04 | 0.04 | 0.05 | 0.08 | 0.13 | 0.24 | 0.46 | 0.76 | 1.31 | 2.05 | 2.92 | 4.18 | 5.50 | 6.33 | 6.95 | 7.21 |
| 2032 | 0.04 | 0.04 | 0.04 | 0.05 | 0.08 | 0.13 | 0.24 | 0.46 | 0.76 | 1.30 | 2.05 | 2.92 | 4.16 | 5.52 | 6.34 | 6.95 | 7.20 |
| 2033 | 0.04 | 0.04 | 0.04 | 0.05 | 0.08 | 0.13 | 0.24 | 0.46 | 0.75 | 1.29 | 2.05 | 2.91 | 4.14 | 5.53 | 6.34 | 6.95 | 7.20 |
| 2034 | 0.04 | 0.04 | 0.04 | 0.05 | 0.08 | 0.13 | 0.24 | 0.46 | 0.75 | 1.28 | 2.04 | 2.91 | 4.12 | 5.55 | 6.35 | 6.96 | 7.19 |
| 2035 | 0.04 | 0.04 | 0.04 | 0.05 | 0.08 | 0.13 | 0.24 | 0.45 | 0.74 | 1.27 | 2.03 | 2.90 | 4.11 | 5.51 | 6.35 | 6.95 | 7.18 |

| | ASDR | | | | | | | | | | | | | | | | |
|---|---|---|---|---|---|---|---|---|---|---|---|---|---|---|---|---|---|
| **year** | **15 to 19** | **20 to 24** | **25 to 29** | **30 to 34** | **35 to 39** | **40 to 44** | **45 to 49** | **50 to 54** | **55 to 59** | **60 to 64** | **65 to 69** | **70 to 74** | **75 to 79** | **80 to 84** | **85 to 89** | **90 to 94** | **95 plus** |
| 2036 | 0.04 | 0.04 | 0.04 | 0.05 | 0.08 | 0.13 | 0.23 | 0.45 | 0.74 | 1.26 | 2.01 | 2.90 | 4.10 | 5.48 | 6.36 | 6.94 | 7.17 |
| 2037 | 0.04 | 0.04 | 0.04 | 0.05 | 0.08 | 0.13 | 0.23 | 0.45 | 0.73 | 1.25 | 1.99 | 2.89 | 4.09 | 5.44 | 6.36 | 6.94 | 7.16 |
| 2038 | 0.04 | 0.04 | 0.04 | 0.05 | 0.08 | 0.13 | 0.23 | 0.44 | 0.73 | 1.24 | 1.98 | 2.88 | 4.08 | 5.41 | 6.37 | 6.93 | 7.15 |
| 2039 | 0.04 | 0.04 | 0.04 | 0.05 | 0.08 | 0.13 | 0.23 | 0.44 | 0.72 | 1.23 | 1.96 | 2.87 | 4.07 | 5.38 | 6.38 | 6.92 | 7.13 |
| 2040 | 0.04 | 0.04 | 0.04 | 0.05 | 0.08 | 0.13 | 0.23 | 0.44 | 0.72 | 1.22 | 1.95 | 2.85 | 4.06 | 5.36 | 6.34 | 6.93 | 7.13 |
| 2041 | 0.04 | 0.04 | 0.04 | 0.05 | 0.08 | 0.13 | 0.23 | 0.43 | 0.72 | 1.22 | 1.93 | 2.82 | 4.04 | 5.35 | 6.30 | 6.93 | 7.12 |
| 2042 | 0.04 | 0.04 | 0.04 | 0.05 | 0.08 | 0.13 | 0.22 | 0.43 | 0.71 | 1.21 | 1.92 | 2.80 | 4.03 | 5.34 | 6.26 | 6.94 | 7.11 |
| 2043 | 0.04 | 0.04 | 0.04 | 0.05 | 0.08 | 0.12 | 0.22 | 0.43 | 0.71 | 1.20 | 1.90 | 2.78 | 4.02 | 5.32 | 6.22 | 6.95 | 7.11 |
| 2044 | 0.04 | 0.04 | 0.04 | 0.05 | 0.08 | 0.12 | 0.22 | 0.42 | 0.70 | 1.19 | 1.89 | 2.76 | 4.01 | 5.31 | 6.18 | 6.95 | 7.10 |
| 2045 | 0.04 | 0.04 | 0.04 | 0.05 | 0.08 | 0.12 | 0.22 | 0.42 | 0.70 | 1.19 | 1.87 | 2.74 | 4.00 | 5.30 | 6.15 | 6.96 | 7.09 |

## Table S13 Projected Absolute Death Numbers for NALC in Men Globally by Age Group through 2045

| | Deaths-number | | | | | | | | | | | | | | | | |
|---|---|---|---|---|---|---|---|---|---|---|---|---|---|---|---|---|---|
| **year** | **15 to 19** | **20 to 24** | **25 to 29** | **30 to 34** | **35 to 39** | **40 to 44** | **45 to 49** | **50 to 54** | **55 to 59** | **60 to 64** | **65 to 69** | **70 to 74** | **75 to 79** | **80 to 84** | **85 to 89** | **90 to 94** | **95 plus** |
| 1992 | 34.97 | 42.19 | 62.60 | 101.16 | 179.13 | 303.70 | 420.49 | 623.54 | 932.40 | 1180.57 | 1188.66 | 1044.46 | 786.68 | 480.38 | 201.80 | 50.59 | 7.52 |
| 1993 | 35.70 | 43.38 | 65.90 | 107.76 | 182.57 | 324.06 | 440.29 | 629.26 | 945.77 | 1218.36 | 1250.79 | 1113.41 | 806.24 | 502.93 | 214.70 | 55.71 | 8.22 |
| 1994 | 36.55 | 44.61 | 69.81 | 116.79 | 185.12 | 341.16 | 466.42 | 640.15 | 959.63 | 1249.66 | 1319.53 | 1185.23 | 833.96 | 527.32 | 228.21 | 60.72 | 8.82 |
| 1995 | 37.20 | 46.11 | 74.13 | 128.23 | 188.31 | 362.86 | 492.01 | 651.32 | 982.37 | 1294.10 | 1397.76 | 1266.81 | 893.46 | 555.30 | 243.66 | 66.08 | 9.64 |
| 1996 | 37.81 | 46.73 | 75.87 | 135.78 | 189.97 | 377.49 | 524.28 | 670.24 | 992.92 | 1329.34 | 1457.88 | 1335.50 | 954.40 | 576.37 | 260.26 | 71.60 | 10.66 |
| 1997 | 38.54 | 46.72 | 76.11 | 140.48 | 192.25 | 386.40 | 551.19 | 701.90 | 992.83 | 1341.93 | 1497.95 | 1387.32 | 1015.13 | 590.99 | 273.75 | 76.91 | 11.74 |
| 1998 | 40.46 | 47.19 | 77.68 | 145.82 | 205.36 | 385.60 | 586.12 | 747.98 | 1009.15 | 1367.86 | 1563.72 | 1471.84 | 1107.62 | 620.32 | 290.90 | 82.65 | 12.83 |
| 1999 | 41.83 | 47.53 | 78.12 | 149.15 | 226.36 | 380.82 | 623.81 | 799.94 | 1035.06 | 1382.66 | 1605.10 | 1551.79 | 1191.57 | 649.37 | 307.17 | 88.23 | 14.11 |
| 2000 | 42.86 | 48.17 | 76.68 | 151.01 | 245.16 | 377.41 | 661.83 | 849.30 | 1058.49 | 1399.24 | 1637.12 | 1629.74 | 1266.95 | 701.63 | 325.04 | 95.19 | 15.55 |
| 2001 | 43.56 | 48.52 | 75.31 | 151.09 | 256.14 | 375.91 | 669.42 | 893.49 | 1075.62 | 1400.38 | 1651.83 | 1680.70 | 1322.59 | 749.86 | 340.41 | 101.72 | 17.01 |
| 2002 | 44.49 | 48.39 | 72.21 | 142.66 | 251.39 | 379.55 | 652.34 | 927.98 | 1128.89 | 1399.77 | 1661.28 | 1708.53 | 1382.38 | 810.16 | 353.64 | 107.83 | 18.42 |
| 2003 | 45.04 | 48.48 | 68.15 | 133.36 | 244.10 | 380.57 | 631.01 | 953.97 | 1171.37 | 1397.24 | 1657.61 | 1725.46 | 1440.53 | 863.88 | 364.18 | 112.63 | 20.31 |
| 2004 | 45.66 | 49.08 | 67.29 | 129.22 | 239.75 | 388.77 | 617.09 | 971.38 | 1214.71 | 1397.61 | 1642.02 | 1729.91 | 1489.82 | 910.96 | 370.79 | 115.77 | 21.36 |
| 2005 | 46.05 | 49.75 | 67.12 | 124.92 | 238.68 | 403.21 | 609.54 | 1009.70 | 1272.69 | 1404.21 | 1647.29 | 1744.57 | 1555.04 | 958.45 | 392.46 | 120.41 | 22.31 |

| year | Deaths-number | | | | | | | | | | | | | | | | |
|---|---|---|---|---|---|---|---|---|---|---|---|---|---|---|---|---|---|
| | 15 to 19 | 20 to 24 | 25 to 29 | 30 to 34 | 35 to 39 | 40 to 44 | 45 to 49 | 50 to 54 | 55 to 59 | 60 to 64 | 65 to 69 | 70 to 74 | 75 to 79 | 80 to 84 | 85 to 89 | 90 to 94 | 95 plus |
| 2006 | 45.89 | 50.98 | 66.78 | 121.22 | 239.55 | 424.68 | 603.02 | 1038.81 | 1345.94 | 1422.98 | 1644.93 | 1749.46 | 1604.49 | 1005.12 | 419.41 | 125.19 | 23.56 |
| 2007 | 45.64 | 52.85 | 68.09 | 120.95 | 243.86 | 445.42 | 613.72 | 1064.92 | 1404.82 | 1495.04 | 1660.51 | 1801.75 | 1661.43 | 1068.17 | 452.63 | 130.37 | 25.16 |
| 2008 | 45.78 | 54.88 | 70.92 | 124.33 | 249.51 | 451.20 | 652.00 | 1085.84 | 1477.63 | 1597.37 | 1713.63 | 1866.23 | 1737.13 | 1169.32 | 503.86 | 143.62 | 27.93 |
| 2009 | 45.38 | 56.04 | 73.89 | 128.63 | 247.04 | 442.43 | 697.29 | 1078.64 | 1523.55 | 1700.28 | 1760.33 | 1901.19 | 1800.64 | 1287.31 | 565.11 | 159.48 | 30.73 |
| 2010 | 45.06 | 57.25 | 76.51 | 130.09 | 243.08 | 441.82 | 730.52 | 1074.44 | 1588.33 | 1801.40 | 1821.08 | 1946.30 | 1857.66 | 1402.63 | 621.40 | 180.73 | 34.29 |
| 2011 | 44.94 | 57.15 | 78.50 | 128.61 | 233.95 | 437.93 | 759.03 | 1091.25 | 1645.83 | 1923.80 | 1876.60 | 1983.44 | 1912.38 | 1446.40 | 650.47 | 193.99 | 36.84 |
| 2012 | 44.83 | 56.32 | 80.97 | 128.46 | 228.02 | 435.07 | 795.22 | 1125.83 | 1704.13 | 2032.19 | 1977.59 | 2023.23 | 1968.31 | 1494.36 | 692.69 | 211.19 | 40.42 |
| 2013 | 44.96 | 56.22 | 84.90 | 133.08 | 229.17 | 441.52 | 827.58 | 1188.04 | 1738.03 | 2151.59 | 2111.95 | 2069.68 | 2026.20 | 1633.11 | 782.97 | 237.33 | 44.88 |
| 2014 | 45.41 | 55.68 | 88.42 | 138.75 | 231.43 | 445.92 | 854.47 | 1281.69 | 1766.73 | 2291.25 | 2303.78 | 2129.09 | 2091.83 | 1735.60 | 857.39 | 261.65 | 49.02 |
| 2015 | 45.56 | 55.72 | 90.66 | 143.52 | 232.57 | 444.46 | 872.38 | 1366.96 | 1801.07 | 2452.37 | 2495.47 | 2201.78 | 2162.92 | 1741.24 | 873.18 | 275.67 | 54.49 |
| 2016 | 45.49 | 56.35 | 91.07 | 147.97 | 234.30 | 443.76 | 873.57 | 1400.15 | 1824.12 | 2515.88 | 2653.32 | 2277.06 | 2213.01 | 1769.47 | 903.21 | 294.79 | 59.47 |
| 2017 | 46.07 | 57.14 | 90.91 | 152.80 | 240.77 | 448.54 | 862.78 | 1392.64 | 1853.39 | 2524.13 | 2719.31 | 2390.10 | 2254.05 | 1861.30 | 978.70 | 324.66 | 63.94 |
| 2018 | 46.75 | 58.52 | 91.15 | 158.48 | 246.13 | 449.08 | 861.00 | 1410.68 | 1912.29 | 2572.45 | 2854.09 | 2530.37 | 2319.28 | 1918.47 | 1019.83 | 353.40 | 70.98 |
| 2019 | 46.74 | 58.95 | 89.96 | 163.15 | 252.86 | 450.34 | 856.47 | 1419.11 | 1980.82 | 2591.18 | 2954.08 | 2680.09 | 2381.67 | 1946.16 | 1044.27 | 378.18 | 77.00 |
| 2020 | 46.84 | 59.41 | 88.62 | 165.61 | 262.07 | 455.76 | 854.10 | 1430.68 | 2090.76 | 2624.83 | 3061.21 | 2813.74 | 2451.67 | 2005.24 | 1096.97 | 409.55 | 82.07 |

| | Deaths-number | | | | | | | | | | | | | | | | |
|---|---|---|---|---|---|---|---|---|---|---|---|---|---|---|---|---|---|
| **year** | **15 to 19** | **20 to 24** | **25 to 29** | **30 to 34** | **35 to 39** | **40 to 44** | **45 to 49** | **50 to 54** | **55 to 59** | **60 to 64** | **65 to 69** | **70 to 74** | **75 to 79** | **80 to 84** | **85 to 89** | **90 to 94** | **95 plus** |
| 2021 | 47.92 | 60.45 | 89.59 | 167.11 | 271.70 | 456.13 | 842.87 | 1437.22 | 2159.91 | 2658.77 | 3129.00 | 2932.76 | 2504.34 | 2050.26 | 1136.62 | 435.00 | 89.15 |
| 2022 | 52.26 | 60.13 | 90.85 | 168.04 | 280.30 | 471.37 | 839.93 | 1482.33 | 2245.72 | 2664.51 | 3163.68 | 3034.27 | 2483.50 | 1968.87 | 1100.93 | 416.05 | 73.49 |
| 2023 | 54.15 | 60.30 | 90.77 | 167.95 | 288.07 | 481.49 | 831.29 | 1493.48 | 2282.16 | 2749.31 | 3240.02 | 3198.46 | 2626.45 | 2001.06 | 1136.29 | 433.31 | 79.15 |
| 2024 | 56.11 | 60.53 | 90.79 | 167.60 | 296.09 | 490.06 | 827.17 | 1496.64 | 2322.64 | 2867.55 | 3293.29 | 3370.83 | 2795.45 | 2049.03 | 1178.85 | 454.04 | 85.87 |
| 2025 | 57.84 | 62.07 | 90.60 | 165.48 | 299.79 | 500.91 | 835.53 | 1484.41 | 2342.17 | 2991.91 | 3310.07 | 3496.20 | 2990.64 | 2113.37 | 1224.19 | 471.30 | 92.31 |
| 2026 | 59.55 | 63.63 | 90.56 | 163.74 | 301.47 | 512.00 | 848.73 | 1467.90 | 2364.79 | 3112.22 | 3325.12 | 3606.92 | 3185.44 | 2202.82 | 1269.36 | 492.55 | 98.53 |
| 2027 | 61.27 | 65.37 | 90.54 | 162.75 | 300.40 | 523.19 | 866.11 | 1452.29 | 2385.94 | 3196.76 | 3372.97 | 3709.78 | 3370.59 | 2336.25 | 1309.88 | 516.30 | 104.39 |
| 2028 | 62.90 | 67.28 | 90.57 | 162.05 | 298.34 | 534.54 | 883.79 | 1438.66 | 2393.60 | 3242.56 | 3492.79 | 3803.85 | 3551.54 | 2490.67 | 1350.60 | 540.13 | 110.55 |
| 2029 | 64.39 | 69.26 | 90.66 | 161.53 | 295.79 | 546.17 | 898.27 | 1432.30 | 2386.58 | 3290.93 | 3649.76 | 3860.91 | 3730.49 | 2659.29 | 1393.79 | 563.41 | 117.04 |
| 2030 | 65.45 | 70.90 | 92.28 | 160.51 | 290.94 | 551.20 | 914.70 | 1443.24 | 2363.58 | 3315.61 | 3805.22 | 3877.30 | 3870.07 | 2847.44 | 1441.64 | 588.09 | 122.40 |
| 2031 | 66.45 | 72.50 | 93.94 | 159.74 | 286.77 | 552.44 | 931.45 | 1462.50 | 2333.63 | 3344.29 | 3954.18 | 3891.57 | 3992.87 | 3034.42 | 1509.21 | 613.08 | 128.86 |
| 2032 | 67.31 | 74.09 | 95.84 | 159.02 | 283.94 | 548.59 | 948.26 | 1488.85 | 2305.08 | 3370.34 | 4056.40 | 3946.85 | 4107.28 | 3211.78 | 1610.56 | 635.78 | 135.82 |
| 2033 | 67.93 | 75.57 | 97.97 | 158.36 | 281.62 | 542.93 | 965.27 | 1515.50 | 2279.66 | 3376.81 | 4109.46 | 4090.18 | 4212.07 | 3383.64 | 1726.19 | 659.24 | 142.77 |
| 2034 | 68.32 | 76.88 | 100.21 | 157.81 | 279.60 | 536.38 | 982.66 | 1536.44 | 2265.89 | 3362.20 | 4165.92 | 4276.58 | 4273.90 | 3553.98 | 1849.76 | 683.81 | 149.71 |
| 2035 | 68.11 | 77.65 | 101.91 | 159.62 | 276.93 | 526.11 | 989.38 | 1560.10 | 2279.05 | 3325.82 | 4195.32 | 4458.91 | 4291.36 | 3690.94 | 1984.26 | 709.83 | 157.20 |

| year | Deaths-number | | | | | | | | | | | | | | | | |
|---|---|---|---|---|---|---|---|---|---|---|---|---|---|---|---|---|---|
| | 15 to 19 | 20 to 24 | 25 to 29 | 30 to 34 | 35 to 39 | 40 to 44 | 45 to 49 | 50 to 54 | 55 to 59 | 60 to 64 | 65 to 69 | 70 to 74 | 75 to 79 | 80 to 84 | 85 to 89 | 90 to 94 | 95 plus |
| 2036 | 67.68 | 78.33 | 103.53 | 161.48 | 274.71 | 517.09 | 989.18 | 1584.19 | 2305.35 | 3279.41 | 4229.35 | 4631.52 | 4306.62 | 3811.59 | 2117.53 | 747.92 | 165.03 |
| 2037 | 67.18 | 78.86 | 105.15 | 163.74 | 272.55 | 510.52 | 979.75 | 1608.27 | 2342.78 | 3234.91 | 4259.26 | 4747.56 | 4371.44 | 3925.15 | 2244.52 | 805.67 | 172.30 |
| 2038 | 66.86 | 79.08 | 106.59 | 166.38 | 270.49 | 504.86 | 967.08 | 1632.53 | 2380.35 | 3194.82 | 4263.67 | 4806.72 | 4539.87 | 4029.95 | 2366.55 | 870.23 | 179.90 |
| 2039 | 66.81 | 79.06 | 107.78 | 169.18 | 268.59 | 499.74 | 952.84 | 1657.29 | 2408.61 | 3171.34 | 4240.85 | 4870.45 | 4755.35 | 4090.95 | 2488.26 | 936.96 | 187.70 |
| 2040 | 67.01 | 78.80 | 108.81 | 171.96 | 271.55 | 495.74 | 935.80 | 1670.77 | 2447.30 | 3193.38 | 4200.65 | 4915.81 | 4973.03 | 4118.14 | 2594.55 | 1008.62 | 196.21 |
| 2041 | 67.45 | 78.29 | 109.73 | 174.62 | 274.58 | 492.54 | 920.99 | 1672.47 | 2486.77 | 3234.18 | 4147.37 | 4966.44 | 5177.54 | 4144.04 | 2689.61 | 1080.22 | 208.67 |
| 2042 | 67.97 | 77.69 | 110.43 | 177.25 | 278.31 | 489.44 | 910.58 | 1658.42 | 2526.33 | 3290.97 | 4096.35 | 5011.37 | 5317.24 | 4224.69 | 2781.28 | 1150.04 | 226.97 |
| 2043 | 68.51 | 77.31 | 110.71 | 179.59 | 282.68 | 486.54 | 901.78 | 1638.82 | 2566.29 | 3347.89 | 4050.93 | 5025.46 | 5395.64 | 4415.07 | 2867.83 | 1216.88 | 246.87 |
| 2044 | 69.05 | 77.24 | 110.63 | 181.51 | 287.33 | 483.94 | 893.94 | 1616.51 | 2607.10 | 3391.55 | 4027.03 | 5006.77 | 5480.87 | 4649.50 | 2921.11 | 1284.46 | 266.66 |
| 2045 | 69.61 | 77.45 | 110.22 | 183.14 | 291.90 | 481.09 | 887.86 | 1590.03 | 2645.29 | 3430.45 | 4035.42 | 4962.06 | 5586.93 | 4866.39 | 2954.55 | 1357.05 | 286.10 |

## Table S14 Projected age-standardized death rate (ASDR) for NALC in Men Globally by Age Group through 2045

| | ASDR | | | | | | | | | | | | | | | | |
|---|---|---|---|---|---|---|---|---|---|---|---|---|---|---|---|---|---|
| year | 15 to 19 | 20 to 24 | 25 to 29 | 30 to 34 | 35 to 39 | 40 to 44 | 45 to 49 | 50 to 54 | 55 to 59 | 60 to 64 | 65 to 69 | 70 to 74 | 75 to 79 | 80 to 84 | 85 to 89 | 90 to 94 | 95 plus |
| 1992 | 0.01 | 0.02 | 0.03 | 0.05 | 0.10 | 0.19 | 0.34 | 0.57 | 0.97 | 1.44 | 1.94 | 2.57 | 3.08 | 3.41 | 3.62 | 3.56 | 2.65 |
| 1993 | 0.01 | 0.02 | 0.03 | 0.05 | 0.10 | 0.20 | 0.34 | 0.57 | 0.97 | 1.47 | 1.97 | 2.63 | 3.14 | 3.47 | 3.70 | 3.67 | 2.77 |
| 1994 | 0.01 | 0.02 | 0.03 | 0.05 | 0.10 | 0.20 | 0.34 | 0.58 | 0.98 | 1.49 | 2.01 | 2.69 | 3.20 | 3.55 | 3.79 | 3.75 | 2.83 |
| 1995 | 0.01 | 0.02 | 0.03 | 0.06 | 0.10 | 0.21 | 0.35 | 0.59 | 0.99 | 1.53 | 2.07 | 2.77 | 3.33 | 3.67 | 3.91 | 3.83 | 2.92 |
| 1996 | 0.01 | 0.02 | 0.03 | 0.06 | 0.10 | 0.21 | 0.36 | 0.60 | 0.99 | 1.55 | 2.11 | 2.82 | 3.42 | 3.77 | 4.04 | 3.92 | 3.00 |
| 1997 | 0.01 | 0.02 | 0.03 | 0.06 | 0.10 | 0.21 | 0.36 | 0.60 | 0.98 | 1.54 | 2.13 | 2.82 | 3.48 | 3.83 | 4.11 | 3.99 | 3.10 |
| 1998 | 0.01 | 0.02 | 0.03 | 0.06 | 0.10 | 0.21 | 0.37 | 0.62 | 1.00 | 1.55 | 2.20 | 2.88 | 3.63 | 3.98 | 4.23 | 4.10 | 3.14 |
| 1999 | 0.01 | 0.02 | 0.03 | 0.06 | 0.11 | 0.20 | 0.39 | 0.63 | 1.02 | 1.54 | 2.23 | 2.93 | 3.74 | 4.08 | 4.33 | 4.20 | 3.19 |
| 2000 | 0.01 | 0.02 | 0.03 | 0.06 | 0.11 | 0.20 | 0.40 | 0.64 | 1.03 | 1.54 | 2.24 | 2.98 | 3.81 | 4.25 | 4.47 | 4.36 | 3.24 |
| 2001 | 0.01 | 0.02 | 0.03 | 0.06 | 0.11 | 0.20 | 0.39 | 0.65 | 1.03 | 1.52 | 2.23 | 3.00 | 3.82 | 4.33 | 4.60 | 4.49 | 3.29 |
| 2002 | 0.01 | 0.02 | 0.03 | 0.06 | 0.11 | 0.20 | 0.37 | 0.65 | 1.04 | 1.51 | 2.20 | 2.98 | 3.83 | 4.45 | 4.70 | 4.60 | 3.32 |
| 2003 | 0.01 | 0.02 | 0.03 | 0.05 | 0.10 | 0.19 | 0.36 | 0.65 | 1.04 | 1.49 | 2.15 | 2.97 | 3.83 | 4.52 | 4.77 | 4.64 | 3.45 |
| 2004 | 0.01 | 0.02 | 0.03 | 0.05 | 0.10 | 0.19 | 0.35 | 0.64 | 1.02 | 1.48 | 2.09 | 2.93 | 3.81 | 4.54 | 4.74 | 4.61 | 3.44 |
| 2005 | 0.01 | 0.02 | 0.03 | 0.05 | 0.10 | 0.19 | 0.34 | 0.64 | 1.03 | 1.47 | 2.06 | 2.90 | 3.84 | 4.56 | 4.79 | 4.63 | 3.41 |

| | ASDR | | | | | | | | | | | | | | | | |
|---|---|---|---|---|---|---|---|---|---|---|---|---|---|---|---|---|---|
| year | 15 to 19 | 20 to 24 | 25 to 29 | 30 to 34 | 35 to 39 | 40 to 44 | 45 to 49 | 50 to 54 | 55 to 59 | 60 to 64 | 65 to 69 | 70 to 74 | 75 to 79 | 80 to 84 | 85 to 89 | 90 to 94 | 95 plus |
| 2006 | 0.01 | 0.02 | 0.03 | 0.05 | 0.10 | 0.19 | 0.33 | 0.64 | 1.03 | 1.46 | 2.03 | 2.85 | 3.84 | 4.58 | 4.83 | 4.68 | 3.43 |
| 2007 | 0.01 | 0.02 | 0.03 | 0.05 | 0.10 | 0.20 | 0.33 | 0.64 | 1.04 | 1.47 | 2.02 | 2.86 | 3.87 | 4.64 | 4.91 | 4.73 | 3.47 |
| 2008 | 0.01 | 0.02 | 0.03 | 0.05 | 0.10 | 0.20 | 0.34 | 0.65 | 1.06 | 1.51 | 2.06 | 2.89 | 3.95 | 4.84 | 5.15 | 5.04 | 3.65 |
| 2009 | 0.01 | 0.02 | 0.03 | 0.05 | 0.10 | 0.19 | 0.35 | 0.64 | 1.06 | 1.52 | 2.08 | 2.88 | 4.00 | 5.09 | 5.46 | 5.37 | 3.79 |
| 2010 | 0.01 | 0.02 | 0.03 | 0.05 | 0.10 | 0.19 | 0.35 | 0.63 | 1.06 | 1.54 | 2.12 | 2.89 | 4.03 | 5.33 | 5.68 | 5.71 | 4.01 |
| 2011 | 0.01 | 0.02 | 0.03 | 0.05 | 0.09 | 0.18 | 0.36 | 0.63 | 1.07 | 1.56 | 2.14 | 2.89 | 4.04 | 5.29 | 5.66 | 5.72 | 4.10 |
| 2012 | 0.01 | 0.02 | 0.03 | 0.05 | 0.09 | 0.18 | 0.36 | 0.63 | 1.09 | 1.60 | 2.15 | 2.90 | 4.04 | 5.29 | 5.71 | 5.81 | 4.30 |
| 2013 | 0.01 | 0.02 | 0.03 | 0.05 | 0.09 | 0.18 | 0.37 | 0.65 | 1.09 | 1.63 | 2.20 | 2.91 | 4.04 | 5.62 | 6.11 | 6.12 | 4.57 |
| 2014 | 0.01 | 0.02 | 0.03 | 0.05 | 0.09 | 0.18 | 0.38 | 0.67 | 1.10 | 1.69 | 2.26 | 2.94 | 4.07 | 5.81 | 6.37 | 6.38 | 4.80 |
| 2015 | 0.01 | 0.02 | 0.03 | 0.05 | 0.09 | 0.18 | 0.38 | 0.69 | 1.11 | 1.73 | 2.34 | 2.99 | 4.10 | 5.65 | 6.22 | 6.37 | 5.05 |
| 2016 | 0.01 | 0.02 | 0.03 | 0.05 | 0.09 | 0.18 | 0.38 | 0.69 | 1.10 | 1.73 | 2.37 | 3.01 | 4.10 | 5.56 | 6.17 | 6.45 | 5.20 |
| 2017 | 0.01 | 0.02 | 0.03 | 0.05 | 0.09 | 0.18 | 0.37 | 0.66 | 1.09 | 1.70 | 2.34 | 3.01 | 4.09 | 5.65 | 6.44 | 6.70 | 5.25 |
| 2018 | 0.01 | 0.02 | 0.03 | 0.05 | 0.09 | 0.18 | 0.37 | 0.66 | 1.09 | 1.71 | 2.37 | 3.04 | 4.11 | 5.63 | 6.48 | 6.87 | 5.49 |
| 2019 | 0.01 | 0.02 | 0.03 | 0.05 | 0.09 | 0.18 | 0.36 | 0.66 | 1.09 | 1.70 | 2.38 | 3.03 | 4.13 | 5.54 | 6.40 | 6.95 | 5.59 |
| 2020 | 0.01 | 0.02 | 0.03 | 0.05 | 0.09 | 0.18 | 0.36 | 0.65 | 1.11 | 1.71 | 2.37 | 3.05 | 4.18 | 5.56 | 6.51 | 7.21 | 5.63 |

| | ASDR | | | | | | | | | | | | | | | | |
|---|---|---|---|---|---|---|---|---|---|---|---|---|---|---|---|---|---|
| year | 15 to 19 | 20 to 24 | 25 to 29 | 30 to 34 | 35 to 39 | 40 to 44 | 45 to 49 | 50 to 54 | 55 to 59 | 60 to 64 | 65 to 69 | 70 to 74 | 75 to 79 | 80 to 84 | 85 to 89 | 90 to 94 | 95 plus |
| 2021 | 0.01 | 0.02 | 0.03 | 0.05 | 0.10 | 0.18 | 0.35 | 0.65 | 1.11 | 1.71 | 2.37 | 3.04 | 4.19 | 5.59 | 6.59 | 7.46 | 5.90 |
| 2022 | 0.02 | 0.02 | 0.03 | 0.05 | 0.10 | 0.18 | 0.35 | 0.65 | 1.11 | 1.70 | 2.38 | 3.17 | 4.23 | 5.55 | 6.47 | 7.22 | 5.89 |
| 2023 | 0.02 | 0.02 | 0.03 | 0.05 | 0.10 | 0.18 | 0.35 | 0.65 | 1.12 | 1.69 | 2.39 | 3.21 | 4.26 | 5.54 | 6.47 | 7.27 | 6.00 |
| 2024 | 0.02 | 0.02 | 0.03 | 0.05 | 0.10 | 0.18 | 0.34 | 0.64 | 1.12 | 1.69 | 2.39 | 3.25 | 4.29 | 5.53 | 6.47 | 7.33 | 6.10 |
| 2025 | 0.02 | 0.02 | 0.03 | 0.05 | 0.10 | 0.18 | 0.34 | 0.64 | 1.11 | 1.69 | 2.39 | 3.25 | 4.32 | 5.56 | 6.46 | 7.31 | 6.15 |
| 2026 | 0.02 | 0.02 | 0.03 | 0.05 | 0.10 | 0.18 | 0.34 | 0.63 | 1.10 | 1.69 | 2.38 | 3.25 | 4.36 | 5.59 | 6.45 | 7.30 | 6.20 |
| 2027 | 0.02 | 0.02 | 0.03 | 0.05 | 0.10 | 0.18 | 0.34 | 0.63 | 1.09 | 1.69 | 2.37 | 3.24 | 4.40 | 5.62 | 6.45 | 7.28 | 6.25 |
| 2028 | 0.02 | 0.02 | 0.03 | 0.05 | 0.10 | 0.18 | 0.34 | 0.62 | 1.08 | 1.69 | 2.37 | 3.24 | 4.43 | 5.65 | 6.44 | 7.26 | 6.30 |
| 2029 | 0.02 | 0.02 | 0.03 | 0.05 | 0.10 | 0.19 | 0.34 | 0.61 | 1.07 | 1.69 | 2.36 | 3.24 | 4.47 | 5.68 | 6.43 | 7.25 | 6.35 |
| 2030 | 0.02 | 0.02 | 0.03 | 0.05 | 0.10 | 0.18 | 0.34 | 0.61 | 1.06 | 1.67 | 2.36 | 3.22 | 4.45 | 5.72 | 6.45 | 7.23 | 6.32 |
| 2031 | 0.02 | 0.02 | 0.03 | 0.05 | 0.10 | 0.18 | 0.34 | 0.61 | 1.05 | 1.66 | 2.35 | 3.21 | 4.44 | 5.75 | 6.48 | 7.21 | 6.30 |
| 2032 | 0.02 | 0.02 | 0.03 | 0.05 | 0.10 | 0.18 | 0.34 | 0.60 | 1.04 | 1.64 | 2.35 | 3.19 | 4.43 | 5.78 | 6.50 | 7.19 | 6.27 |
| 2033 | 0.02 | 0.02 | 0.03 | 0.05 | 0.09 | 0.18 | 0.34 | 0.60 | 1.02 | 1.62 | 2.35 | 3.18 | 4.41 | 5.81 | 6.52 | 7.17 | 6.24 |
| 2034 | 0.02 | 0.02 | 0.03 | 0.05 | 0.09 | 0.18 | 0.34 | 0.60 | 1.01 | 1.60 | 2.34 | 3.16 | 4.40 | 5.85 | 6.54 | 7.14 | 6.22 |
| 2035 | 0.02 | 0.02 | 0.03 | 0.05 | 0.09 | 0.18 | 0.34 | 0.60 | 1.01 | 1.58 | 2.31 | 3.15 | 4.37 | 5.82 | 6.57 | 7.16 | 6.19 |

| | ASDR | | | | | | | | | | | | | | | | |
|---|---|---|---|---|---|---|---|---|---|---|---|---|---|---|---|---|---|
| year | 15 to 19 | 20 to 24 | 25 to 29 | 30 to 34 | 35 to 39 | 40 to 44 | 45 to 49 | 50 to 54 | 55 to 59 | 60 to 64 | 65 to 69 | 70 to 74 | 75 to 79 | 80 to 84 | 85 to 89 | 90 to 94 | 95 plus |
| 2036 | 0.02 | 0.02 | 0.03 | 0.05 | 0.09 | 0.18 | 0.33 | 0.59 | 1.00 | 1.56 | 2.28 | 3.14 | 4.35 | 5.79 | 6.59 | 7.17 | 6.16 |
| 2037 | 0.02 | 0.02 | 0.03 | 0.05 | 0.09 | 0.17 | 0.33 | 0.59 | 0.99 | 1.54 | 2.26 | 3.13 | 4.32 | 5.77 | 6.62 | 7.18 | 6.13 |
| 2038 | 0.02 | 0.02 | 0.03 | 0.05 | 0.09 | 0.17 | 0.33 | 0.59 | 0.98 | 1.52 | 2.23 | 3.11 | 4.29 | 5.74 | 6.64 | 7.19 | 6.10 |
| 2039 | 0.02 | 0.02 | 0.03 | 0.05 | 0.09 | 0.17 | 0.33 | 0.59 | 0.97 | 1.50 | 2.20 | 3.10 | 4.26 | 5.71 | 6.66 | 7.20 | 6.07 |
| 2040 | 0.02 | 0.02 | 0.03 | 0.05 | 0.09 | 0.17 | 0.32 | 0.59 | 0.97 | 1.49 | 2.17 | 3.07 | 4.25 | 5.68 | 6.63 | 7.22 | 6.08 |
| 2041 | 0.02 | 0.02 | 0.03 | 0.05 | 0.09 | 0.17 | 0.32 | 0.58 | 0.97 | 1.48 | 2.15 | 3.03 | 4.23 | 5.64 | 6.60 | 7.25 | 6.09 |
| 2042 | 0.02 | 0.02 | 0.03 | 0.05 | 0.09 | 0.16 | 0.32 | 0.58 | 0.97 | 1.47 | 2.12 | 3.00 | 4.22 | 5.61 | 6.57 | 7.28 | 6.10 |
| 2043 | 0.02 | 0.02 | 0.03 | 0.05 | 0.09 | 0.16 | 0.31 | 0.57 | 0.97 | 1.46 | 2.10 | 2.96 | 4.20 | 5.57 | 6.54 | 7.30 | 6.11 |
| 2044 | 0.02 | 0.02 | 0.03 | 0.05 | 0.09 | 0.16 | 0.31 | 0.57 | 0.96 | 1.45 | 2.07 | 2.93 | 4.18 | 5.54 | 6.51 | 7.33 | 6.11 |
| 2045 | 0.02 | 0.02 | 0.03 | 0.05 | 0.09 | 0.16 | 0.31 | 0.56 | 0.96 | 1.44 | 2.04 | 2.89 | 4.17 | 5.50 | 6.48 | 7.35 | 6.12 |
